# Multiyear tropical warm pool warming drives slowdown in Antarctic mass loss

**DOI:** 10.1038/s41586-026-10912-x

**Published:** 2026-08-19

**Authors:** Yunhe Wang, Qinghua Ding, Xiaofeng Li, Thomas J. Ballinger, Yoshihiro Nakayama, Dániel Topál, Eric J. Steig

**Affiliations:** 1 Key Laboratory of Ocean Observation and Forecasting and Laboratory of Ocean Circulation and Waves, Institute of Oceanology, Chinese Academy of Sciences, Qingdao, China; 2 Department of Geography, and Earth Research Institute, University of California, Santa Barbara, Santa Barbara, CA USA; 3 International Arctic Research Center, University of Alaska Fairbanks, Fairbanks, AK USA; 4 Thayer School of Engineering, Dartmouth College, Hanover, NH USA; 5 Institute of Low Temperature Science, Hokkaido University, Sapporo, Japan; 6 Earth and Life Institute, Université catholique de Louvain, Louvain-la-Neuve, Belgium; 7 HUN-REN Research Centre for Astronomy and Earth Sciences (MTA-Centre of Excellence), Institute for Geological and Geochemical Research, Budapest, Hungary; 8 Department of Earth and Space Sciences and Quaternary Research Center, University of Washington, Seattle, WA USA

**Keywords:** Cryospheric science, Atmospheric dynamics, Hydrology

## Abstract

Antarctic mass loss has been a major contributor to global sea-level rise for most of the last few decades, mainly driven by West Antarctica^1^. During 2021–2023, however, a sharp increase in surface mass balance over Queen Mary Land and Wilkes Land in East Antarctica offset West Antarctic loss and slowed the rate of total ice mass loss^2,3^. Although this slowdown is consistent with the expected long-term precipitation response to global warming through poleward-shifted storm tracks and Antarctic moistening^4^, our results point to a different mechanism. Here we show that the recent ice mass gain was linked to a recurrent atmospheric teleconnection driven by sea surface temperature anomalies in the tropical warm pool, which experienced unusually persistent warming from 2021 to 2023 relative to the previous two decades. On the basis of observations and model experiments, we find that tropical warm pool warming excites a poleward-propagating Rossby-wave train that induces a high-pressure anomaly over East Antarctica, enhancing Queen Mary Land and Wilkes Land precipitation and driving the observed mass gain, with moisture primarily sourced from the mid-latitude Indian Ocean. Similar multiyear warming in the tropical warm pool recurs about once per decade in observations and historical simulations, and its influence on precipitation is distinct from the effects of global warming. Therefore, the recent Antarctic Ice Sheet mass gain is probably temporary and does not yet reflect a sustained, global-warming-driven moistening of Antarctica.

## Abstract (web summary)

Recent Antarctic ice mass gain was linked to a recurrent atmospheric teleconnection, but this recurs about once per decade in observations and historical simulations so does not yet reflect a global-warming-driven moistening of Antarctica.

## Main

Future global sea-level rise is contingent on the stability of the Antarctic Ice Sheet (AIS), which remains the largest source of uncertainty in long-term sea-level projections^1,5^. Over the past three decades, the AIS has shown pronounced regional contrasts in mass changes. West Antarctica has experienced sustained mass loss, estimated at 82 ± 9 Gt yr^−1^ during 1992–2020 (ref. ^6^), driven primarily by oceanic processes, specifically ocean-forced grounding-line retreat and the resulting enhancement of dynamic ice discharge over the Amundsen Sea sector^7,8^. By contrast, the current mass balance of the East AIS (EAIS), which contains nearly 80% of Earth’s land ice, is more sensitive to precipitation-driven variability^9–12^, so snowfall fluctuations can offset or even reverse mass loss^6,11^. Consequently, the East AIS was quasi-balanced over the same period (3 ± 15 Gt yr^−1^), although individual estimates diverge from zero beyond their uncertainties^6^. Regional exceptions to this rough equilibrium occur in Wilkes Land and eastern Queen Mary Land, particularly at the Totten and Denman Glaciers^13–16^. In these regions, similar to West Antarctica, enhanced intrusions of modified circumpolar deep water onto the continental shelf have driven ice-shelf basal melt and acceleration of outlet glacier ice flow since the late 20th century^15,17^.

Coastal precipitation around East Antarctica (EA) is primarily regulated by large-scale atmospheric circulation variability^9,18,19^, with episodic, high-intensity synoptic storms and atmospheric rivers (ARs)^20,21^. As a result, surface mass balance (SMB) in EA shows substantial natural variability across a wide range of timescales^22^. During 2021–2023, EA experienced a large mass gain, leading to a slowdown in integrated Antarctic-wide mass loss over the past two decades, despite continuing mass loss in West Antarctica. Previous studies have linked the EA mass-gain event to several factors, including enhanced precipitation associated with clustered AR intrusions^23^, the prolonged triple-dip La Niña that displaced Southern Hemisphere storm tracks poleward^24^, cyclonic circulation anomalies^2^ and reduced sea ice extent^3^.

Under global warming, both the subtropical and mid-latitude jets show a pronounced southward shift, as supported by CMIP6 model experiments^4^. The associated synoptic storm tracks and AR activity also tend to shift poleward, particularly in the extratropics adjacent to Antarctica^25^. The mechanisms underlying the poleward shifting storm track have been associated with warmer sea surface temperatures (SSTs) on a global scale, the rising trend of the tropopause height^26^ and intensified tropospheric heating in the tropics as well as strengthened upper-level meridional temperature gradients, enhanced static stability^25^ and stronger upper-level winds^27^ over the Southern Hemisphere subtropics. Together with the anthropogenically driven increase in atmospheric moisture constrained by the Clausius–Clapeyron relationship and enhanced precipitation associated with individual storms^27,28^, these processes favour increased Antarctic precipitation and can partially offset ice-sheet mass loss^4,28,29^ primarily driven by oceanic processes. However, it remains unclear whether these primarily anthropogenically driven changes in the Southern Hemisphere storm track and high-latitude precipitation responses have contributed to the 2021–2023 mass-gain event. Storm-track and AR activity around Antarctica is strongly modulated by large-scale circulation variability arising from both anthropogenic forcing and internal climate variability, especially those related to tropical-SST-driven teleconnections^23,30,31^. Isolating the internally versus externally forced climate mechanisms that shape anomalous East AIS mass variations is a critical part of process-based understanding of current and future Antarctic mass-balance change.

Unlike in West Antarctica, where climate variability is unambiguously linked to teleconnections driven by tropical Pacific and Atlantic SSTs^30,32,33^, the large-scale circulation drivers of EA climate remain less understood. Precipitation and ARs over EA have been statistically linked to the El Niño–Southern Oscillation (ENSO) and Indian Ocean Dipole (IOD)^34^, but the underlying dynamics governing EA precipitation variability remain less known. To address these questions, we combine a wide array of observations and simulations to understand possible forcing mechanisms and moisture pathways driving EA precipitation anomalies, with a particular focus on the 2021–2023 mass-gain event.

## East Antarctic precipitation drives the mass gain

**Fig. 1:**
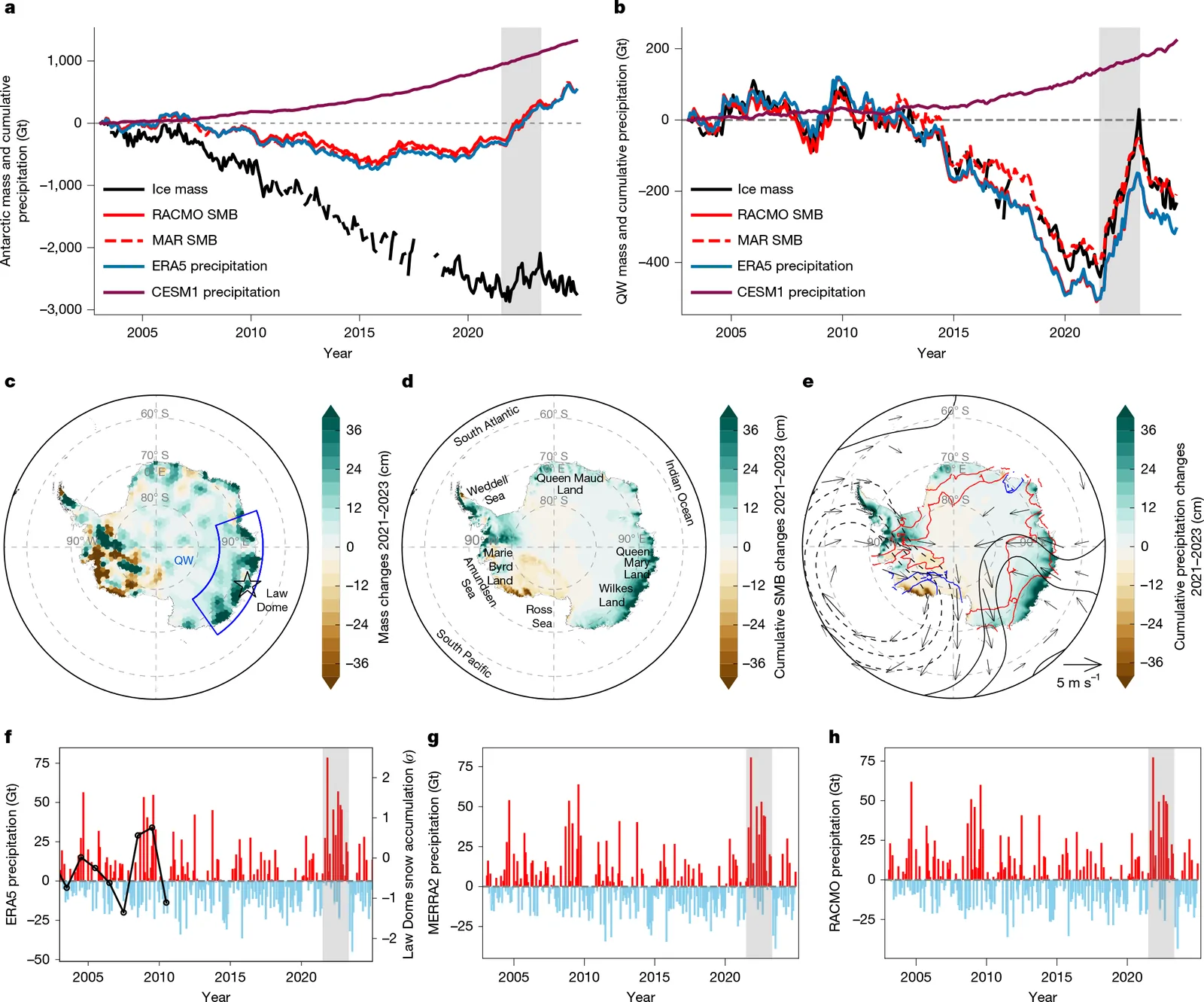
Antarctic mass change and associated precipitation anomalies in the past two decades. **a**, Deseasonalized Antarctic ice mass changes from GRACE/GRACE-FO, compared with cumulative SMB anomalies from RACMO2.4p1 and MARv3.14, ERA5 precipitation and CESM1 40-member ensemble-mean precipitation, relative to January 2003. The grey shading marks the mass reversal period (July 2021–April 2023). Missing GRACE/GRACE-FO values reflect satellite data gaps. **b**, As in **a**, but for the QW sector (70–145° E, 65–75° S), defined by the blue polygon in **c**. **c**, Antarctic mass changes from GRACE/GRACE-FO during the event (April 2023 minus July 2021). **d**, As in **c**, but for cumulative SMB changes from RACMO2.4p1. **e**, As in **c**, but for cumulative ERA5 precipitation changes. Black contours and vectors represent the mean 300-hPa geopotential height (Z300; contour interval, 20 m) and wind anomalies for the 10 months during the event when area-weighted average QW precipitation exceeded the 80th percentile of the raw monthly distribution for 1979–2024. Red and blue contours indicate positive and negative AR-frequency anomalies during the event, respectively; contour labels are scaled by 10^3^, so 1 and 5 correspond to 0.001 and 0.005 counts per day. Counts per day (ranging from 0 to 1) represents the AR frequency. **f**–**h**, Monthly precipitation anomaly over QW from ERA5 (**f**), MERRA-2 (**g**) and RACMO2.4p1 (**h**): **f** also overlays the Law Dome ice-core snow-accumulation record from ref. ^49^, shown as standardized annual anomalies relative to 1979–2010 and referenced to the right *y* axis. *σ*, standard deviation. Law Dome is located at 112.81° E, 66.77° S and is marked with the black star in **c**. As GRACE/GRACE-FO provides cumulative mass changes, SMB and precipitation anomalies are also shown cumulatively in **a**–**e** and Extended Data Fig. 1a. This representation highlights prolonged same-sign anomalies as step-like changes, whereas short-lived fluctuations largely cancel out.

**Extended Data Fig. 1:**
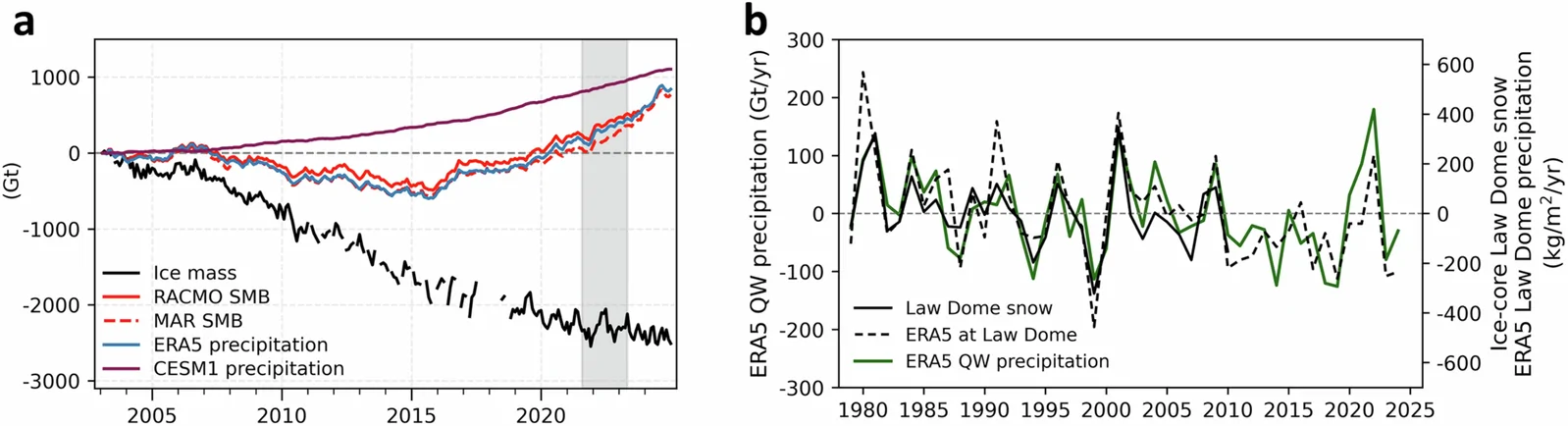
Antarctic mass change excluding the QW sector and Law Dome ice-core-derived snow accumulation. **a**, Time series of deseasonalized Antarctic ice mass changes from GRACE/GRACE-FO, compared with cumulative SMB anomalies from RACMO2.4p1 and MARv3.14, ERA5 precipitation, and CESM1 40-member ensemble-mean precipitation, after excluding the QW sector from the Antarctic total. All series are relative to January 2003. The grey shading marks the mass reversal period (July 2021–April 2023). **b**, Annual ERA5 QW precipitation anomalies, Law Dome ice-core snow-accumulation anomalies, and ERA5 precipitation anomalies at the Law Dome grid point. Law Dome anomalies are relative to the 1979–2010 mean. The left axis shows ERA5 QW precipitation in Gt yr^−¹^, and the right axis shows Law Dome snow accumulation and ERA5 Law Dome precipitation in kg m^−²^ yr^−¹^. The Law Dome record is highly correlated with ERA5 QW precipitation and ERA5 Law Dome precipitation anomalies, with r = 0.83 (p < 0.05) for both comparisons.

Consistent with previous studies^35^, Gravity Recovery and Climate Experiment (GRACE) observations indicate a sustained AIS mass loss rate of 140.5 ± 2.0 Gt yr^−1^ over the period 2003–2024 (Fig. 1a). This long-term decline was abruptly interrupted by pronounced mass gain between July 2021 and April 2023, during which the AIS gained roughly 695.0 Gt of mass compared with that in July 2021, marking the largest 22-month mass gain in the past two decades. As a result, the net Antarctic mass balance between 2021 and 2024 remained close to zero. Spatially, this mass reversal is dominated by an extreme mass gain over EA (Fig. 1c,d) and the Antarctic Peninsula, with the greatest accumulation concentrated in the Queen Mary Land and Wilkes Land sector (QW; 70–145° E, 65–75° S). By contrast, the integrated mass changes in other Antarctic regions remained negative during the event (Extended Data Fig. 1a). The Marie Byrd Land sector continued its long-term mass loss through 2021–2023, while the QW sector experienced a reversal of the previous decade-long decline, gaining 470.3 ± 29.1 Gt, accounting for roughly 68% of the continent-wide mass increase (Fig. 1b). Cumulative ERA5 precipitation (blue curve, 351.2 ± 26.2 Gt) and SMB (red curve; RACMO2.4p1: 348.3 ± 25.3 Gt; MARv3.14: 346.3 ± 24.8 Gt) changes show a close correspondence with GRACE mass changes (black curve) in QW, including a 10-year decline followed by a pronounced step-like increase in 2021 (Fig. 1b). This synchronous change may indicate the strong sensitivity of the QW mass balance to year-to-year variability in large-scale circulation and related precipitation.

**Extended Data Fig. 2:**
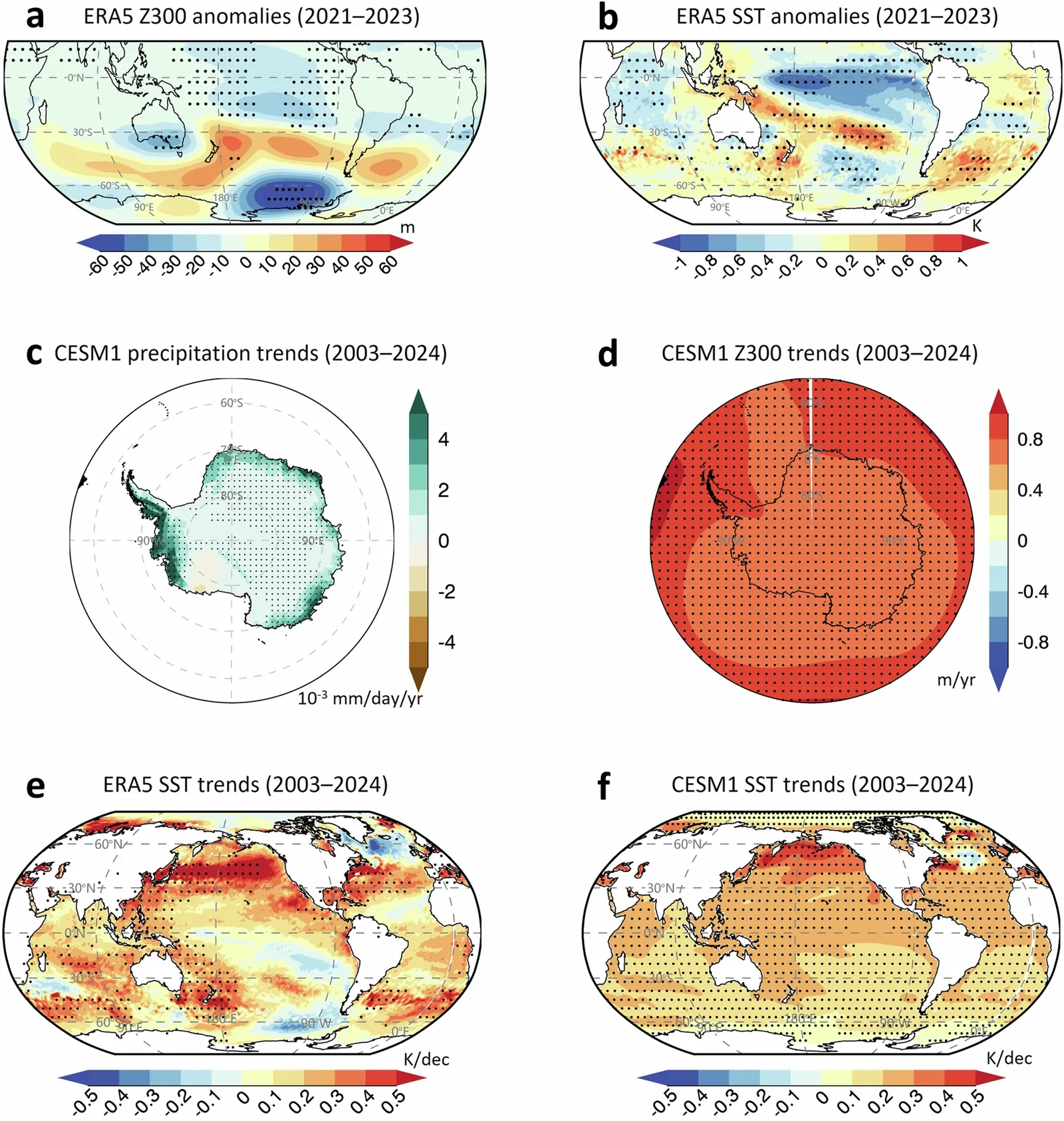
Trends and anomalies of SST, Z300, and precipitation. **a**, Mean ERA5 Z300 anomalies during July 2021 to April 2023. **b**, Mean ERA5 SST anomalies during July 2021 to April 2023. **c**, CESM1 Antarctic precipitation trends for 2003–2024. **d**, As in **c**, but for Z300. **e**, As in **c**, but for global SST trends from ERA5. **f**, As in **e**, but for CESM1 SST. All CESM1 results represent the ensemble mean of 40 members. Anomalies in **a** and **b** are computed after removing the climatological seasonal cycle and linear trend over 1979–2024. Stippling indicates regions significant at the 95% level based on an FDR-corrected Student’s t test.

The step-like jump in the cumulative precipitation was induced by the prolonged positive precipitation anomaly during July 2021–April 2023, which is evident in all three precipitation datasets (Fig. 1f–h). The snow-accumulation record from the Law Dome ice core supports ERA5 precipitation variability over the overlapping period (Fig. 1f and Extended Data Fig. 1b). This anomaly is clearly distinct from the predominantly negative precipitation anomalies seen over the previous 20 years. The recent mass gain over QW was the most pronounced in the 20-year GRACE observational record. The 2021–2023 precipitation anomaly is accompanied by a quasi-barotropic high-pressure anomaly near EA (Extended Data Fig. 2a), embedded within an Antarctic-wide low-pressure anomaly associated with the mega-La Niña^36^. The high-pressure pattern becomes more pronounced during the 10 months (Fig. 1e, black solid contour) that show the strongest positive precipitation over QW (Fig. 1e, green shading), favouring enhanced moisture transport onto the continent by means of large-scale advection (Fig. 1e, vectors) and ARs (Fig. 1e, red contours)^20,37,38^ from the Indian Ocean. In the tropics, 2021–2023 SST anomalies feature warming over the tropical warm pool (TWP), along with a La Niña-like pattern and IOD-like cooling over the Indian Ocean (Extended Data Fig. 2b), maximizing the effect of TWP warming that may favour Rossby-wave excitation and downstream effects on Antarctic atmospheric circulation^31,36^.

The ensemble mean of the CESM1 Large Ensemble historical + RCP8.5 simulations, representing the forced response to anthropogenic forcing, shows a near-linear increase in cumulative Antarctic precipitation (Fig. 1a, magenta curve), with positive precipitation trends mainly along the Antarctic coast (Extended Data Fig. 2c). However, over QW, forced precipitation rose by only 32.3 ± 2.4 Gt during the event, about 9% of the observed ERA5 cumulative precipitation anomaly over the same region and period (351.2 ± 26.2 Gt). These anthropogenically driven moistening trends, associated with widespread Southern Ocean warming (Extended Data Fig. 2e) and a poleward shift of the Southern Hemisphere storm track^4,25,29,39^, are expected to partially offset mass loss over West Antarctica driven by oceanic processes. Notably, the observed high-pressure anomalies over EA (Extended Data Fig. 2a) and SST anomalies in the tropics during the 2021–2023 event (Extended Data Fig. 2b) differ substantially from the long-term circulation and SST trends associated with CO_2_ forcing (Extended Data Fig. 2d,f) in the ensemble mean. This contrast indicates that the observed precipitation increase during this event may arise mainly from the distinct large-scale high-pressure anomaly over EA rather than from the forced response to global warming.

## A dipole drives EA precipitation extremes

**Fig. 2:**
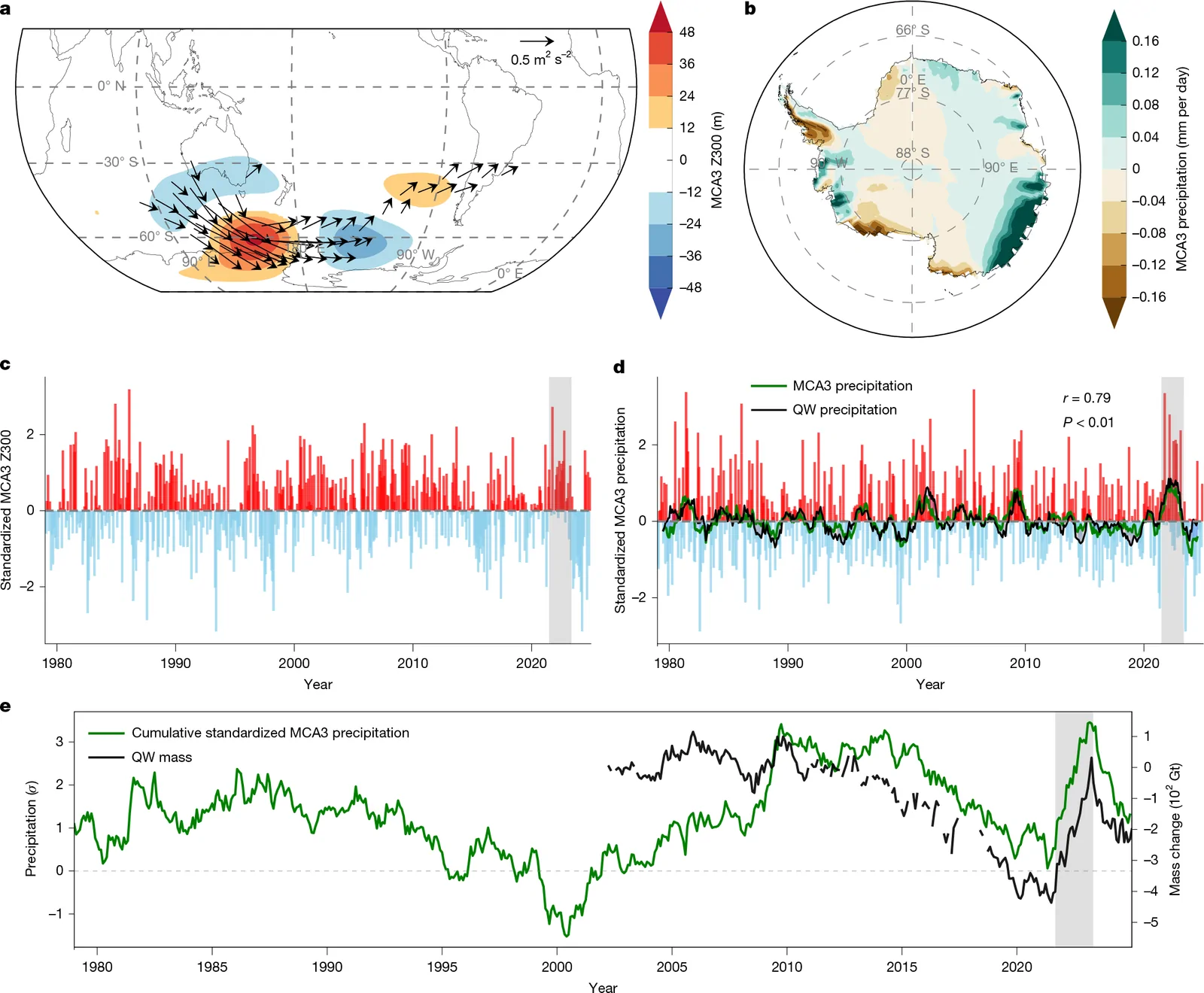
Third MCA mode linking Antarctic precipitation and Southern Hemisphere Z300. MCA is applied to deseasonalized and detrended ERA5 monthly fields (south of 23° N for Z300) for 1979–2024; the third mode explains 8% of the total squared covariance. **a**,**b**, Spatial patterns of MCA3 for Z300 (**a**) and Antarctic precipitation (**b**). Black vectors in **a** denote the associated WAF. **c**,**d**, Standardized time series of the MCA3 Z300 (**c**) and precipitation (**d**). **e**, Comparison between the cumulative standardized MCA3 precipitation time series (green) and the QW mass change time series (black), highlighting their generally coherent variability. In **d**, the green and black solid lines denote the 12-month running means of the standardized MCA3 precipitation time series and the standardized QW precipitation anomalies, respectively; the two time series show a high correlation (*r *= 0.79, *P* < 0.01 based on effective degrees of freedom). Note that the MCA results are insensitive to whether the long-term trend is removed.

**Extended Data Fig. 3:**
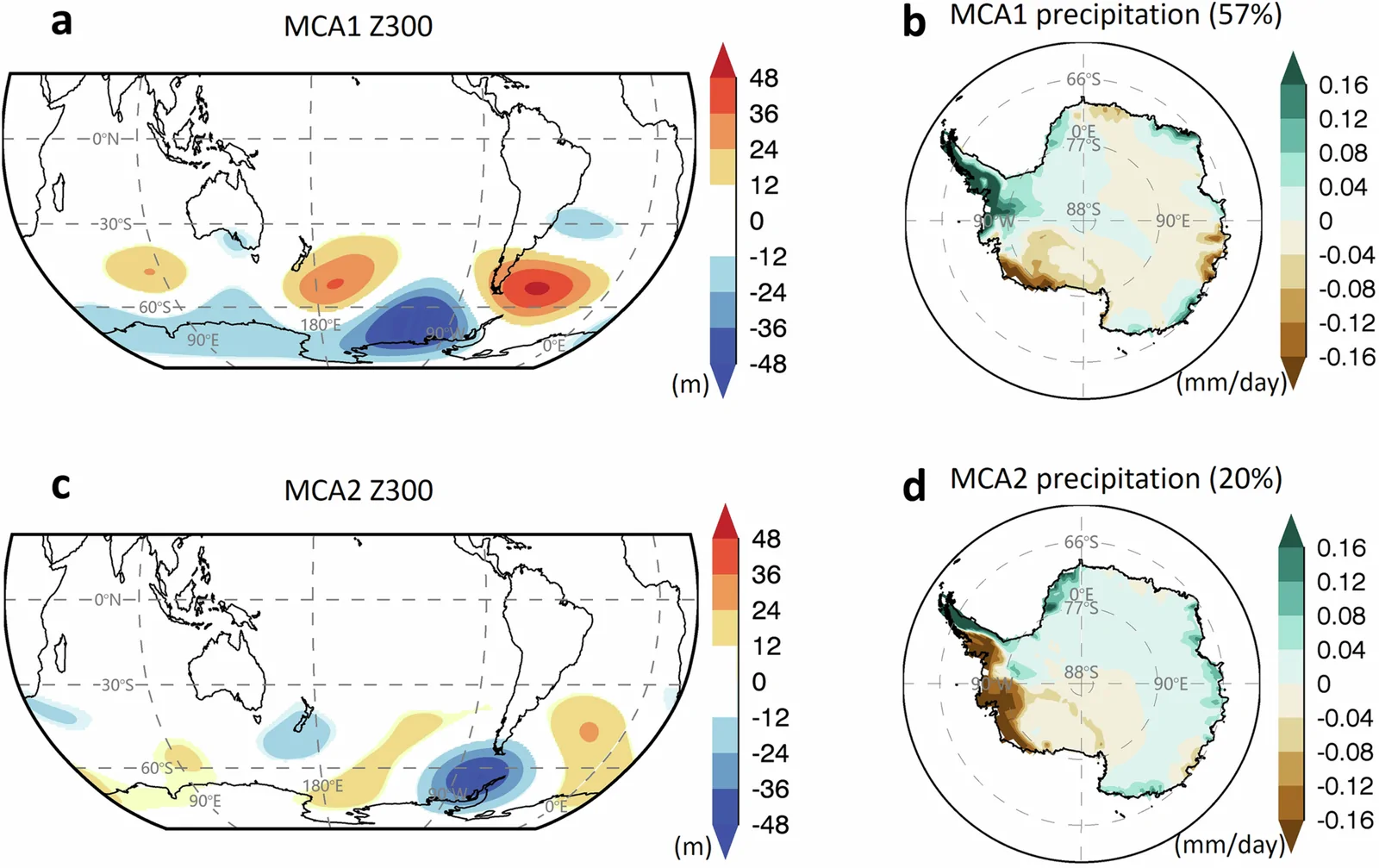
The leading MCA modes between Antarctic precipitation and Southern Hemisphere Z300. MCA is applied to deseasonalized and detrended ERA5 monthly fields (south of 23°N for Z300) for 1979–2024. The first (second) mode explains 57% (20%) of the total squared covariance. **a**, **b**, Spatial patterns of MCA1 mode for Z300 (**a**) and precipitation (**b**). **c**, **d**, As in **a**, **b**, but for MCA2.

**Extended Data Fig. 4:**
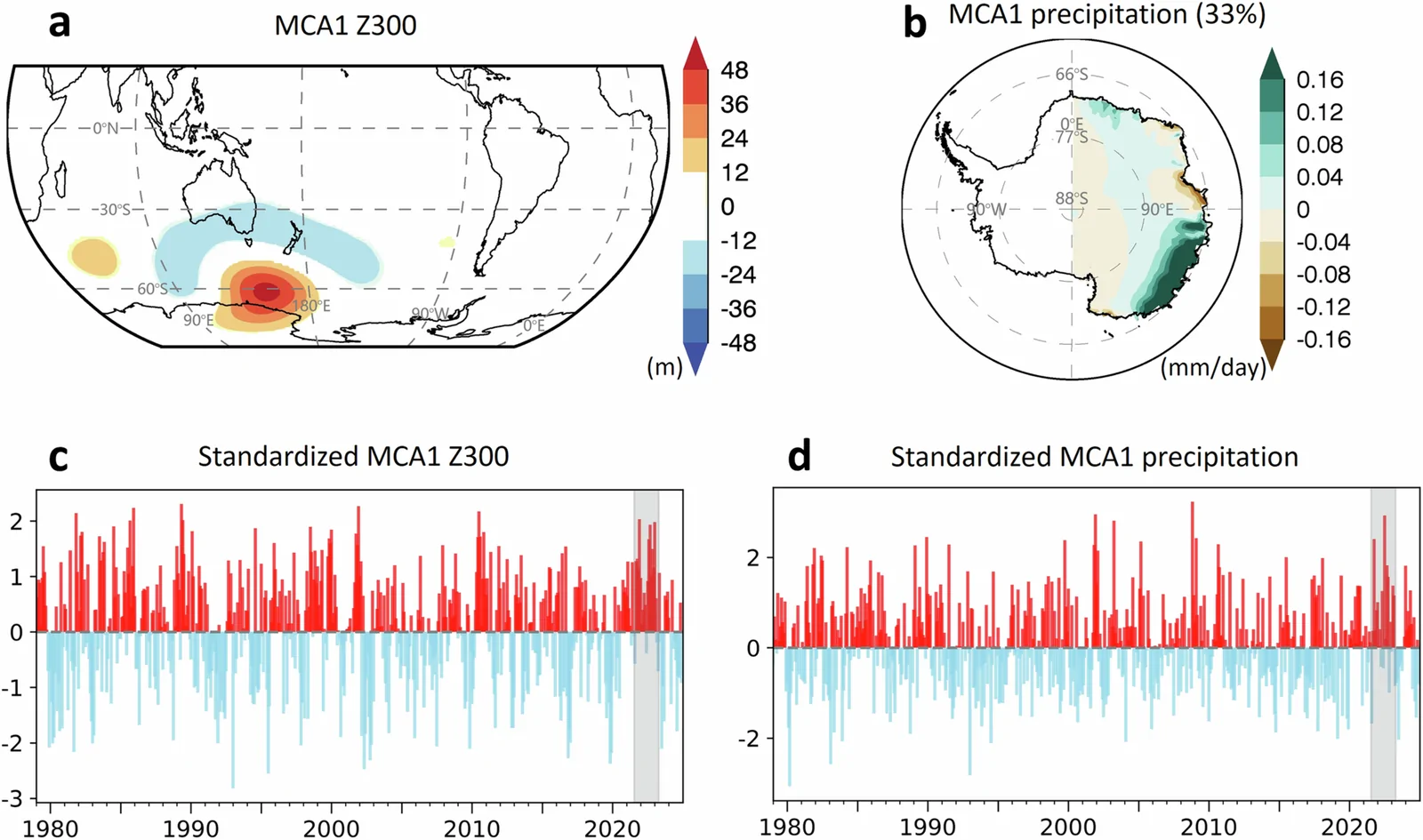
The first MCA mode between Southern Hemisphere Z300 and EA precipitation. MCA is applied to deseasonalized and detrended ERA5 monthly fields (south of 23°N for Z300; 0°–180°E for Antarctic precipitation) for 1979–2024. The first mode explains 33% of the total squared covariance. **a**, **b**, Spatial patterns of MCA1 for Z300 (**a**) and EA precipitation (**b**). **c**, **d**, Standardized time series of MCA1 Z300 (**c**) and EA precipitation (**d**).

To identify the dominant large-scale circulation modes associated with EA precipitation variability, we perform a maximum covariance analysis (MCA) between detrended and deseasonalized monthly ERA5 precipitation over Antarctica and Southern Hemisphere 300-hPa geopotential height (Z300) anomalies for 1979–2024. Z300 is used because it captures large-scale atmospheric teleconnection patterns and is minimally affected by the high topography of the AIS. Because circulation and precipitation variability are more active over the Pacific sector of the Antarctic, the leading two MCA modes capture circulation patterns associated mainly with precipitation variability over West Antarctica and the Antarctic Peninsula, whereas some spatially non-uniform signals are also present over EA (Extended Data Fig. 3a–d). MCA3 shows enhanced precipitation along the EA coast, coupled with a dipole circulation pattern consisting of a low-pressure centre south of Australia and a high-pressure centre over the EA coast (Fig. 2a,b). Although MCA3 explains only about 8% of the total squared covariance, it represents the leading mode associated with precipitation variability over EA. This is further confirmed by an MCA using Southern Hemisphere circulation and precipitation fields with West Antarctica excluded (Extended Data Fig. 4). These MCA results also indicate that precipitation over QW tends to vary more coherently across EA to first order and is most strongly linked to a north–south oriented dipole circulation pattern, both of which are consistent with the co-occurrence of anomalous precipitation and the high-pressure anomaly during the 2021–2023 event.

**Extended Data Fig. 5:**
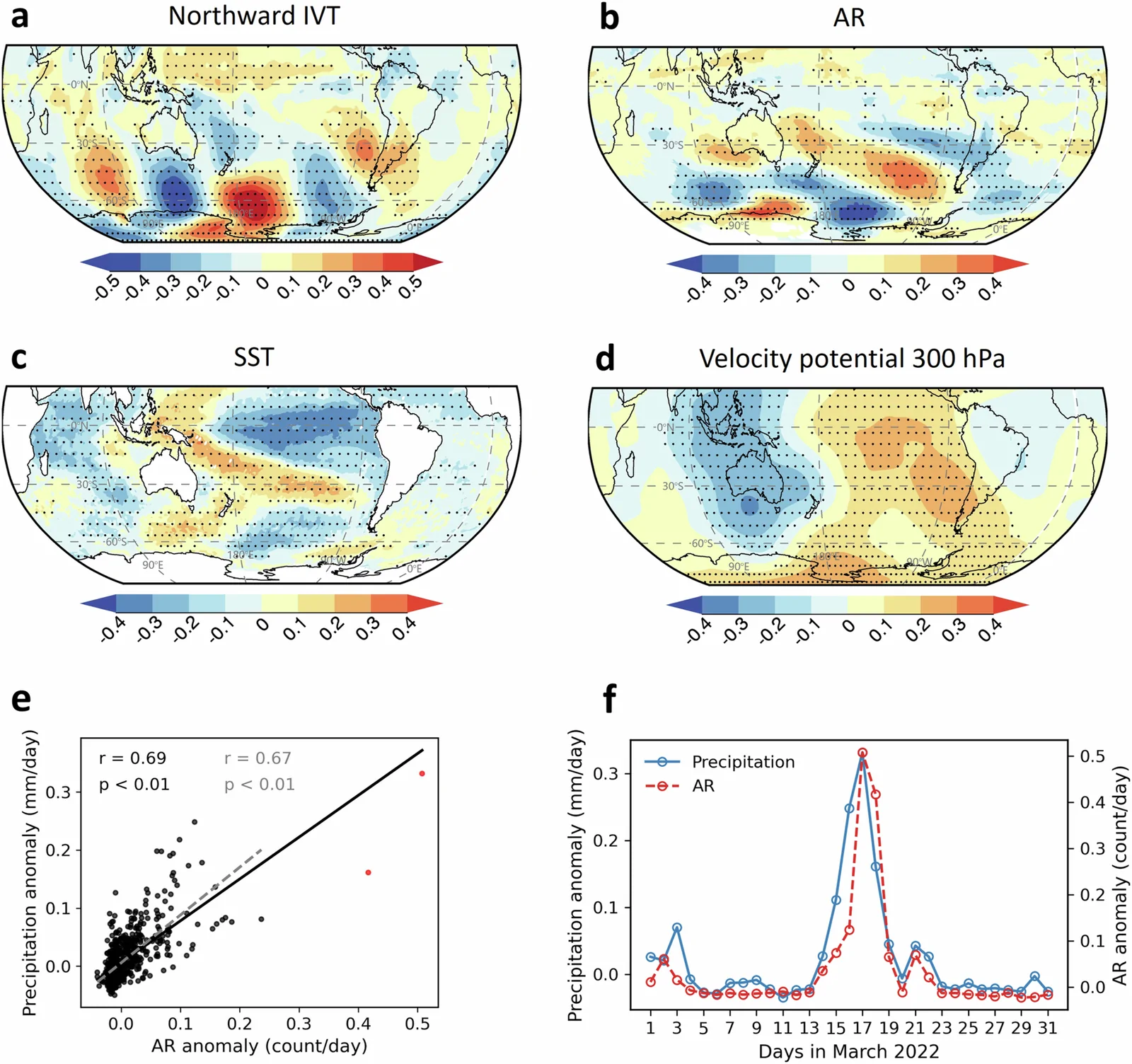
Climate anomalies associated with the MCA3 Z300 index and daily AR–precipitation relationships over QW. **a–d**, Correlation patterns of deseasonalized and detrended ERA5 climate anomalies with the standardized MCA3 Z300 index for 1979–2024. **a**, Meridional integrated water vapour transport (northward IVT). **b**, AR frequency. **c**, SST. **d**, 300-hPa velocity potential. Stippling indicates regions significant at the 95% level based on an FDR-corrected Student’s t test. **e**, Scatterplot of QW area-averaged daily precipitation anomalies versus AR-frequency anomalies during July 2021–April 2023. The solid black line shows the linear fit using all data points (r = 0.69), and the dashed line shows the fit excluding the two days (red dots) with the most extreme ARs (r = 0.67). f, Daily precipitation and AR-frequency anomalies during March 2022, highlighting the close covariability between AR activity and precipitation during the extreme event.

**Extended Data Fig. 6:**
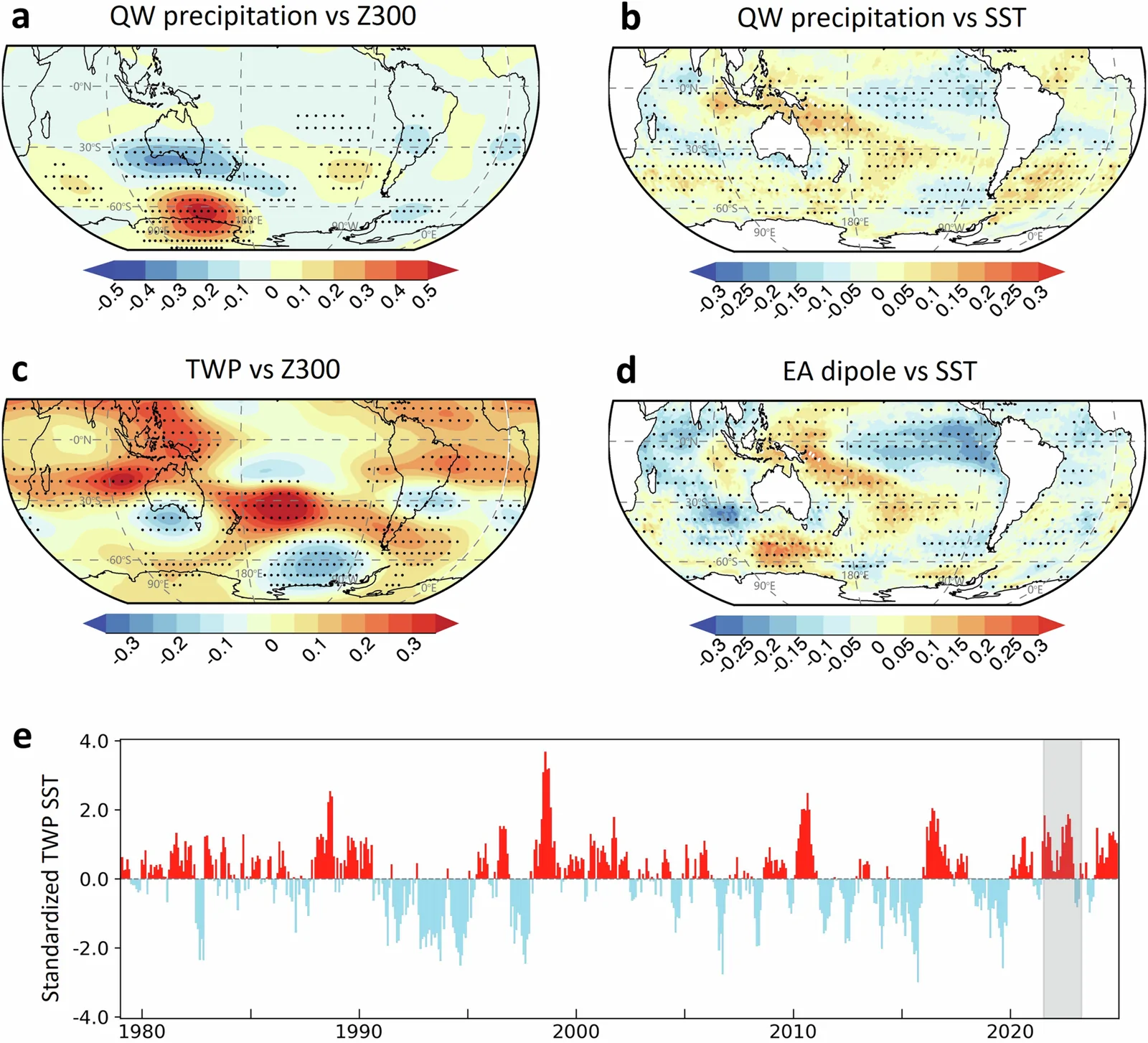
Correlation patterns linking QW precipitation, the EA dipole, and TWP SST variability from ERA5 for 1979–2024. **a**, Correlation between Z300 anomalies and the standardized QW precipitation index. **b**, As in a, but for SST anomalies. **c**, Correlation between Z300 anomalies and the TWP SST index. **d**, Correlation between SST anomalies and the EA dipole index, defined as the area-mean Z300 anomaly over the EA high-pressure center (55°–70°S, 120°–160°E) minus that over the low-pressure center south of Australia (30°–50°S, 80°–150°E). **e**, Standardized time series of the TWP SST index. The TWP index is positive in most months during July 2021–April 2023 and exceeds +1 standard deviation in 12 of the 22 months. **c** and **d** show that TWP SST anomalies are associated with a wave-like Z300 pattern extending toward EA and that the EA dipole index is linked to a similar TWP-related SST pattern. All variables are deseasonalized and detrended before the correlation analysis. Stippling in **a**–**d** indicates regions significant at the 95% level based on a Student’s t test.

The high-pressure anomaly in this dipole circulation pattern enhances poleward moisture transport and AR activity towards EA^40,41^, as reflected by significant anomalies in meridional moisture transport extending from southern Australia into EA and positive AR-frequency anomalies along the western flank of the high-pressure anomaly, leading to increased precipitation over QW (Extended Data Fig. 5a,b). This mode simultaneously reduces precipitation in parts of West Antarctica, but its spatially integrated effect over Antarctica is positive and dominated by the EA contribution. Both the MCA3 Z300 and precipitation time series significantly correlate with QW precipitation over 1979–2024, with coefficients of 0.40 and 0.79, respectively, and also show an extended positive phase from July 2021 to April 2023 (Fig. 2c,d), consistent with QW total precipitation changes in the three datasets (Fig. 1f–h). Using QW precipitation as an index and correlating it with global circulation, a similar local dipole circulation mode can be identified (Extended Data Fig. 6a). We find the time series of the cumulative MCA3 precipitation pattern closely aligns with the GRACE-derived mass-balance gains in QW (Fig. 2e), indicating that MCA3 effectively captures precipitation variations shaping mass change in this region. This consistency is particularly evident after 2010.

The correlation between the MCA3 Z300 time series and global SSTs further suggests a potential linkage between the dipole circulation and TWP warming, accompanied by SST cooling over the Indian Ocean and eastern Pacific and SST warming along the South Pacific Convergence Zone (SPCZ) (Extended Data Fig. 5c). Further correlations based on QW precipitation, TWP SST and the EA dipole further support the presence of a recurrent TWP–EA teleconnection (Extended Data Fig. 6). Diagnostics of Rossby-wave activity flux (WAF) indicate that the dipole circulation structure is associated with pronounced poleward-propagating Rossby-wave activity towards EA (Fig. 2a). Together with the positive SST anomalies and upper-tropospheric divergence over the TWP region (Extended Data Fig. 5c,d), this pattern suggests a possible tropical forcing of the stationary Rossby-wave response. In light of these statistical relationships, we propose that 2–3 years of enhanced precipitation over QW could be remotely driven by concurrent TWP SST warming, which favours a Rossby-wave train propagating towards high southern latitudes and facilitates the buildup of a high-pressure anomaly over EA. This high-pressure anomaly may favour increased precipitation over QW by enhancing moisture transport and promoting AR-related extremes, as reflected by the close day-to-day association between QW precipitation and AR-frequency anomalies during the event (Extended Data Fig. 5e,f).

**Extended Data Fig. 7:**
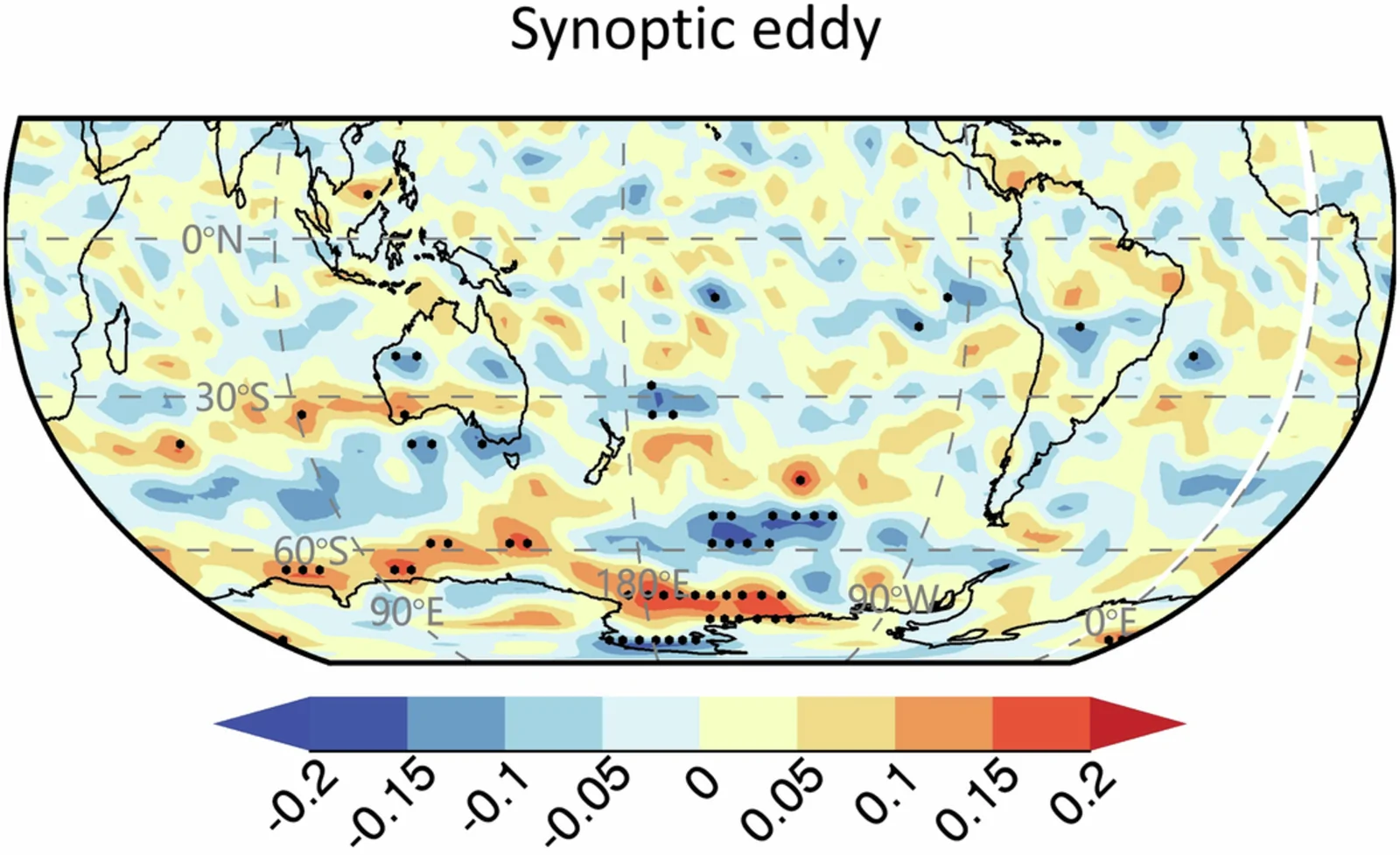
Correlations between the standardized MCA3 Z300 time series and the monthly vorticity tendency term contributed by synoptic eddies (periods < 7 days) for 1979–2024. Stippling indicates regions significant at the 95% confidence level after FDR correction.

It has long been known that low-frequency circulation variability over the Indian Ocean sector of the Southern Hemisphere extratropics, especially a Southern Annular Mode-like dipole circulation pattern, normally involves local eddy-mean flow feedbacks^36^ (Methods). To illustrate the contribution of these feedbacks in maintaining the circulation pattern identified above, we calculate the correlation between the vorticity tendency induced by synoptic eddy forcing and the MCA3 Z300 time series. The positive correlation near EA, although weak (*r* = 0.2), is statistically significant given the ~500-month sample size, indicating an active eddy-mean flow feedback that strengthens the dipole anomalies^36^. This dipole pattern, possibly initiated by remote TWP forcing, acts first to decelerate the subpolar westerly jet near 60° S over the 60° E–180° E sector of the Southern Ocean (Extended Data Fig. 7). The slowdown of the westerly jet then modulates eddy activity along the jet, consequently altering eddy-driven vorticity forcing, which further reinforces both the EA anticyclonic anomaly and the cyclonic anomaly to the north. This positive feedback, together with the tropically driven Rossby-wave train, may work to maintain the observed north–south dipole circulation anomalies near EA.

## TWP warming excites the high-latitude dipole

**Fig. 3:**
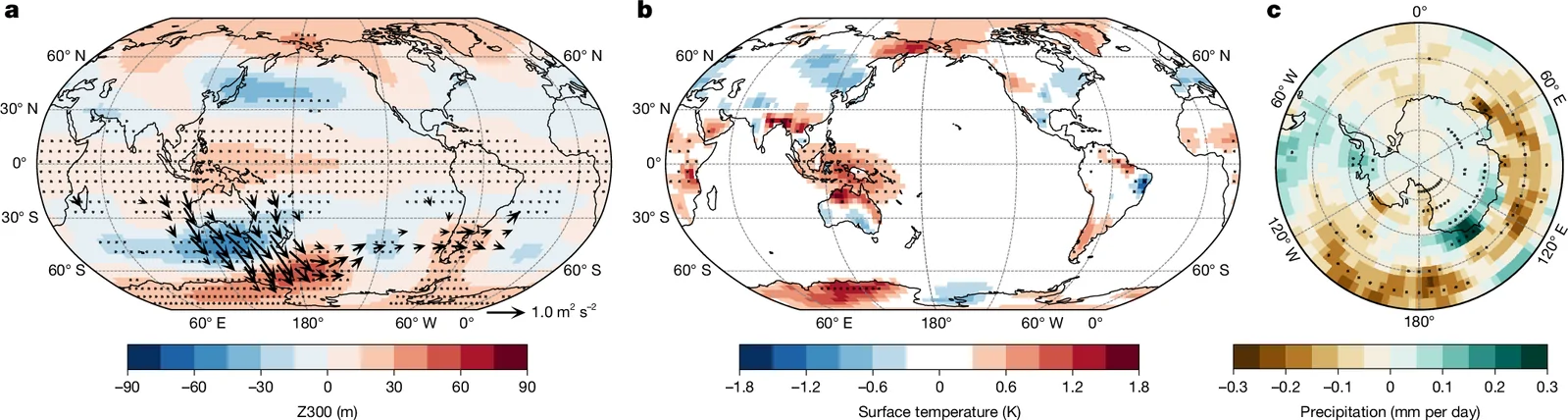
Simulated TWP–EA teleconnection using ECHAM5. Simulated responses to positive TWP SST anomalies, shown as the difference between the 40-year mean of the sensitivity experiment and that of the control experiment. **a**–**c**, Responses of Z300 (**a**), surface temperature (**b**) and precipitation (**c**). Black arrows indicate WAF. Stippling indicates regions significant at the 95% level based on a Student’s *t*-test with false discovery rate correction. The TWP is defined as a 15° clockwise-rotated ellipse centred at 135° E, 5° S, with semi-major and semi-minor axes of 35° and 15°, respectively. Positive SST anomalies within the ellipse increase from 0.5 K at the edge to 1 K at the centre. This smooth gradient avoids sharp SST discontinuities at the forcing boundary.

To test the proposed teleconnection between TWP SST warming and high-latitude precipitation anomalies, we conduct a suite of experiments (a control and a set of sensitivity experiments including experiments 1 to 8; Methods) using two atmospheric general circulation models (AGCMs), ECHAM5 and CAM5. The control experiment is a standard AMIP-type simulation forced with climatological SSTs (Methods). All settings in experiment 1 are the same as in the control experiment, except that we impose positive SST anomalies centred over the Maritime Continent (TWP warming pattern) in ECHAM5. The resulting Z300 response features a low-pressure anomaly south of Australia and a downstream high-pressure anomaly extending southeastward across the Ross Sea into EA (Fig. 3). This circulation pattern closely resembles the observed MCA3-related anomalies (Fig. 2) and the covarying relationships observed during 2021–2023. This pattern appears to be excited by Rossby-wave energy linked to the TWP forcing and propagating poleward, indicating that TWP warming is critical for reproducing the observed dipole circulation pattern over EA. In the tropics, the imposed heating anomaly induces a Gill-type response^42^, characterized by Rossby gyres west of the heating region, which then triggers the extratropical Rossby-wave train. This westward-extended Gill response explains why the diagnosed WAF can appear to emanate from the Indian Ocean sector. During the 2021–2023 event, the observed TWP SST anomaly reaches roughly 0.5 K (Extended Data Fig. 2b), whereas the associated precipitation and circulation anomalies over EA are about 0.3 mm per day and 20 m (Fig. 1e,f and Extended Data Fig. 2a), respectively. This scaling relationship is well reproduced in the model experiments, in which an imposed SST anomaly with an area-mean magnitude of 0.67 K over the forcing region produces precipitation and circulation anomalies of roughly 0.3 mm per day and 40 m over EA (Fig. 3).

**Extended Data Fig. 8:**
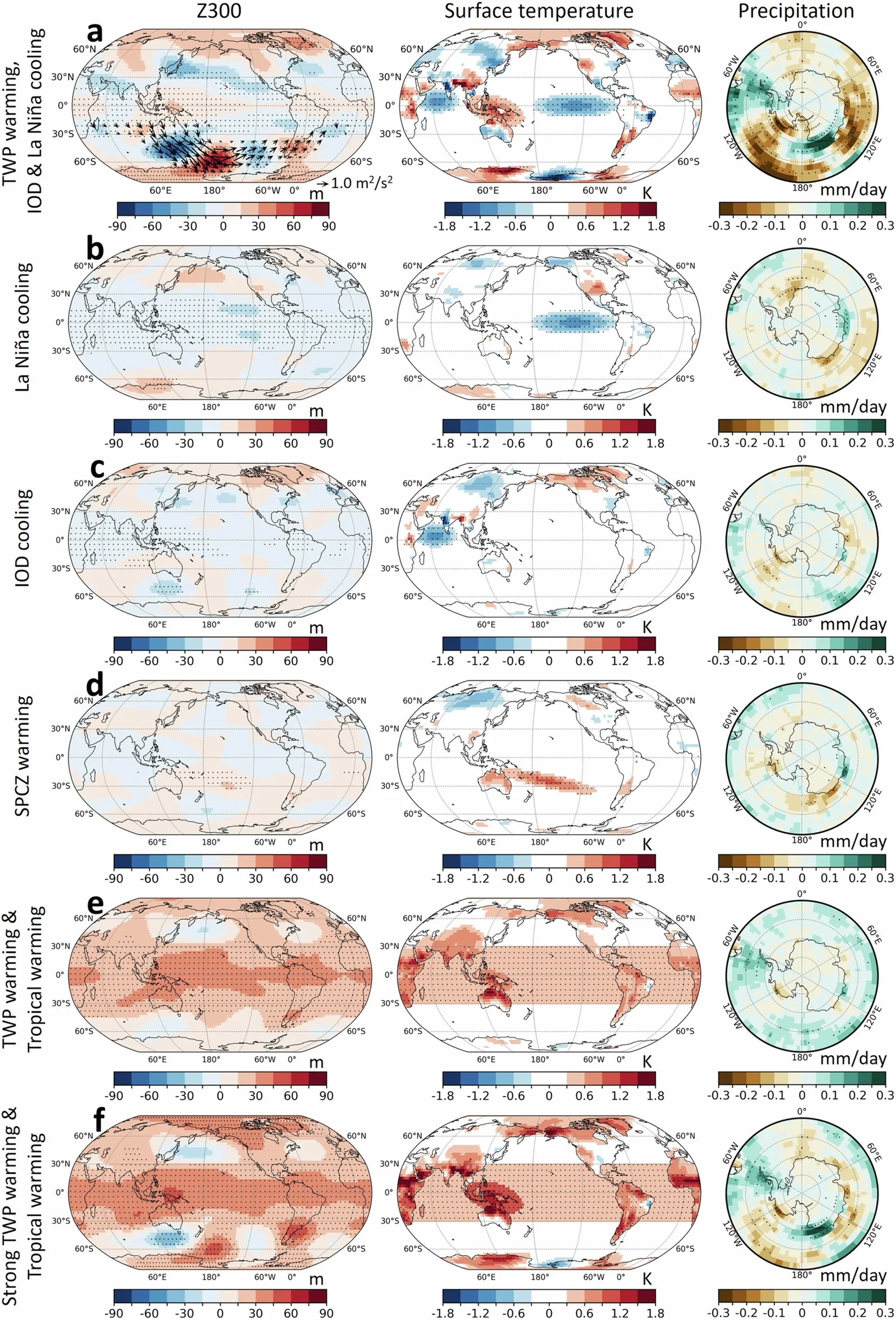
ECHAM5 simulated atmospheric and surface responses to tropical forcing. **a**, Responses of Z300 (left), surface temperature (middle), and precipitation (right) to combined forcing by TWP warming, IOD cooling, and La Niña-like cooling. **b**, **c**, As in **a**, but for isolated La Niña-like cooling (**b**) and isolated IOD cooling (**c**). **d**, As in **a**, but for isolated SPCZ warming. **e**, As in **a**, but for TWP warming superimposed on a uniform tropical warming of 0.5 K between 30°S and 30°N. **f**, As in **e**, but for strong TWP warming. Stippling indicates regions significant at the 95% level based on an FDR-corrected Student’s t test. The TWP is defined as a 15° clockwise-rotated ellipse centered at 135°E, 5°S, with semi-major and semi-minor axes of 35° and 15°, respectively. SST anomalies in the ellipses generally follow a gradient from 0.5 K (or −0.5 K) at the edge to 1 K (or −1 K) at the center, except for the strong TWP warming in **f**, which ranges from 1 K at the edge to 1.5 K at the center.

**Extended Data Fig. 9:**
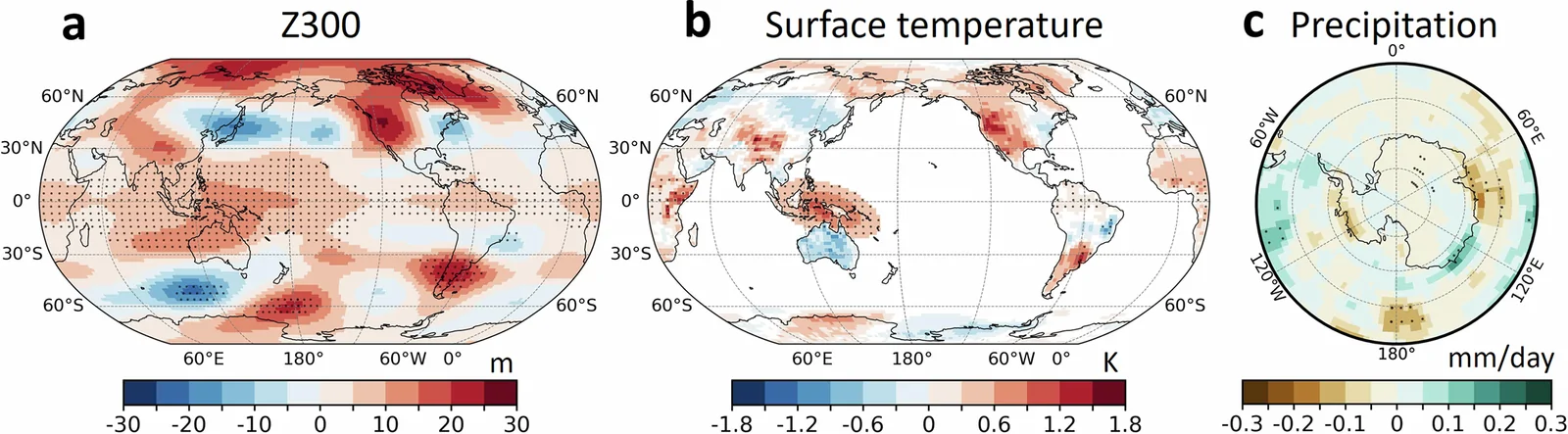
CAM5-simulated TWP–EA teleconnection. Simulated responses to positive TWP SST anomalies, shown as the difference between the 40-year mean of the sensitivity experiment and that of the control experiment. **a**–**c**, Responses of Z300 (a), surface temperature (b), and precipitation (c). Stippling indicates regions significant at the 95% level based on an FDR-corrected Student’s t test.

More sensitivity experiments, shown in Extended Data Figs. 8 and 9 and discussed in the Methods, suggest that the observed tropical–EA teleconnection is most directly driven by TWP SST warming through stationary Rossby-wave propagation. La Niña-like cooling, negative IOD-like cooling and SPCZ-related warming alone do not reproduce the EA dipole or the positive QW precipitation response, but they may modulate TWP SST forcing strength. This series of experiments supports the role of localized TWP SST anomalies, not broad tropical warming, in shaping QW precipitation.

## Moisture sources of QW precipitation anomalies

Identifying the moisture-source regions of the increased QW precipitation may help elucidate the processes governing the regional hydrological response during this event. To address this, we use a water-tagging framework in iCESM1.2, with the atmospheric circulation nudged to ERA5 (Methods), following recent process-oriented water-tracing approaches for diagnosing moisture sources and transport pathways^43–45^. Fifty-four tagging regions are defined globally, each spanning 60° in longitude and 20° in latitude, such that the QW precipitation (climatology or anomalies) equals the sum of contributions from all source regions.

**Fig. 4:**
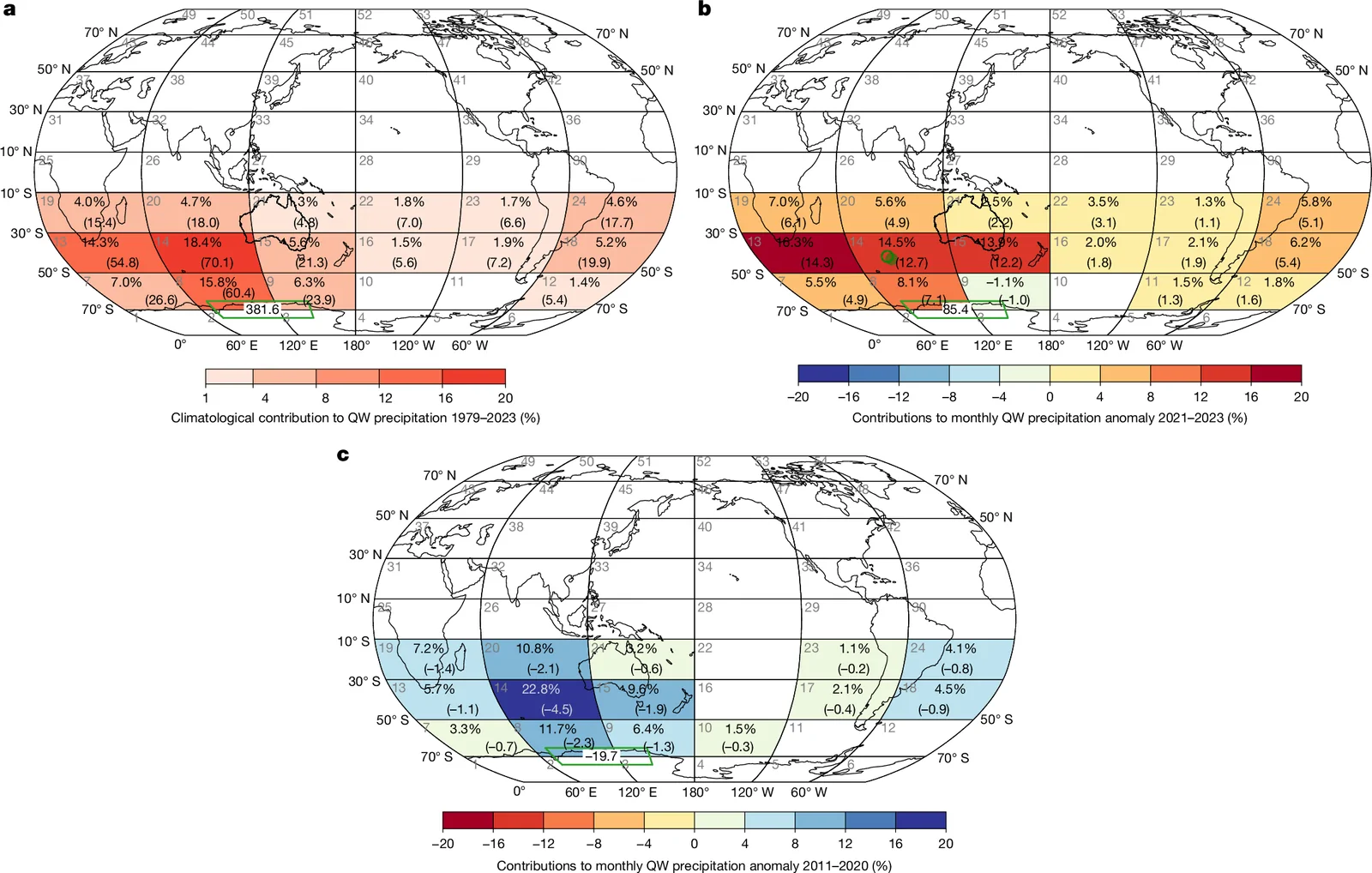
Moisture sources of QW precipitation in the water-tagging run. **a**, Climatological contributions of 54 tagged moisture-source regions to QW precipitation for 1979–2023. **b**, Contributions from each source region to the monthly QW precipitation anomaly during the 2021–2023 event. The green filled dot marks the climatological mean moisture-source location for QW precipitation during 1979–2022, based on the water-tracer dataset from ref. ^47^, and the green open circle marks the corresponding location for July 2021–December 2022. **c**, As in **b**, but for 2011–2020. In all panels, the value within each region denotes the region’s percentage contribution (for example, 18.4% in zone 14 in **a**), with its absolute contribution provided below in parentheses (for example, 70.1 mm per year). Percentages are calculated relative to the QW-domain total (green box value), which represents the area-weighted mean tagged precipitation summed across all 54 regions. Note that the colour bar in **c** is inverted relative to **b** to reflect contributions to a negative QW precipitation anomaly. Only regions with a contribution exceeding 1% are shown.

**Extended Data Fig. 10:**
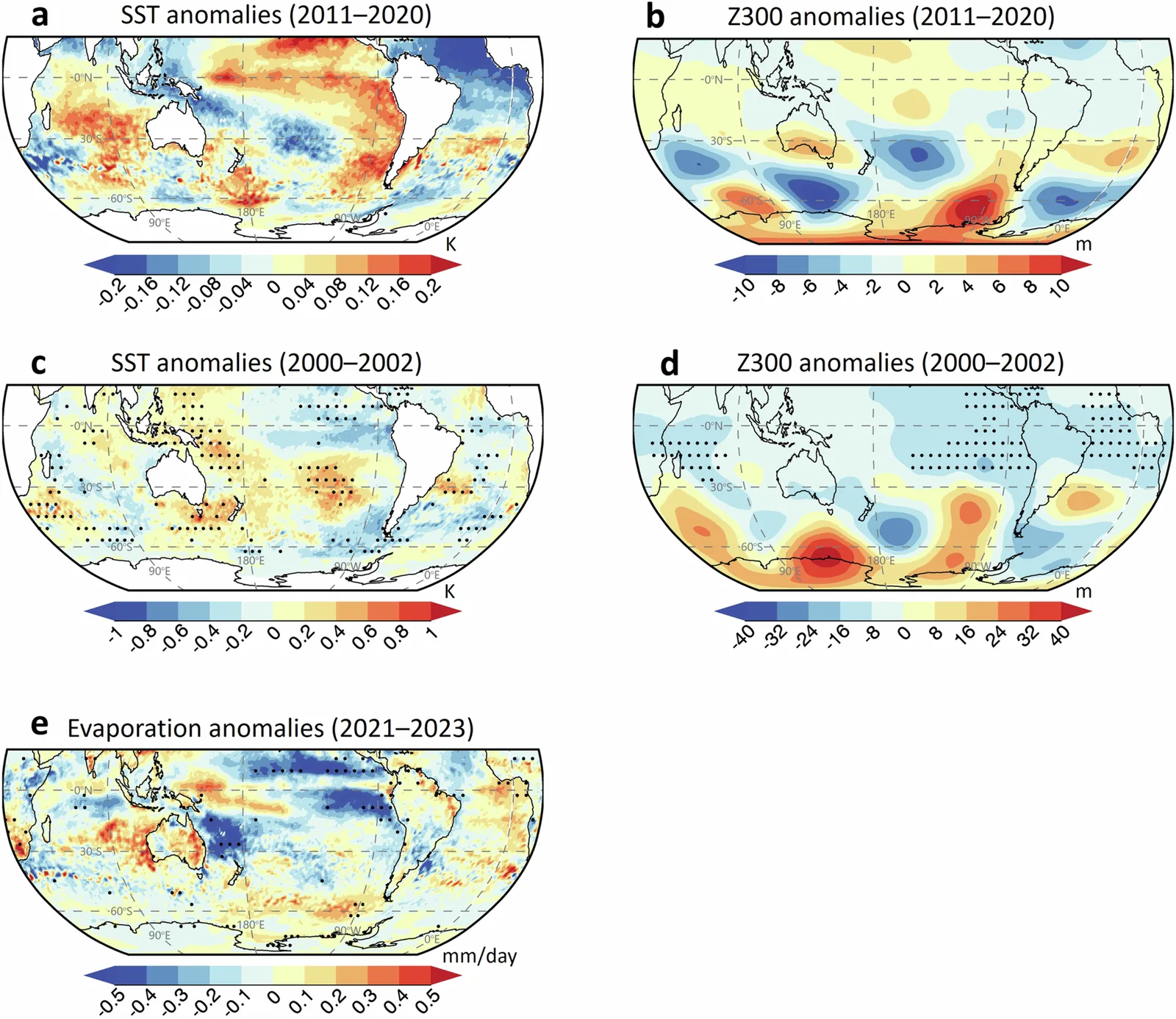
Anomalies of SST, Z300, and evaporation from ERA5. **a**, Mean SST anomalies during 2011 to 2020. **b**, As in **a**, but for Z300. **c**, **d**, As in **a**, **b**, but for the period from August 2000 to June 2002. **e**, As in a, but for evaporation during July 2021 to April 2023, where red (blue) shading indicates enhanced (suppressed) evaporation. Stippling indicates regions significant at the 95% level based on an FDR-corrected Student’s t test.

Climatologically, three regions in the mid-southern Indian Ocean collectively account for roughly 49% of the QW precipitation (Fig. 4a). Among these, zone 14 in the central-southern Indian Ocean (18.4%), zone 8 north of EA (15.8%) and zone 13 over southern Africa (14.3%) rank as the most important sources. During the 2021–2023 event, QW precipitation increases by 85.4 mm per year, primarily driven by increased moisture sourced from zones 13, 14 and 15, which together contribute 45% of this increase (Fig. 4b). Notably, zone 9 is the only region with a negative contribution, a reduction that aligns well with the offshore winds induced by the EA dipole circulation (Fig. 1e), which impede moisture transport to QW from that direction. Together, the anomaly patterns during this event demonstrate that the prolonged precipitation anomalies are supplied primarily by moisture transported from the distant, warm and moisture-rich Indian Ocean sector, rather than by enhanced local evaporation near Antarctica^3^, consistent with AR life-cycle analyses near coastal EA^46^. The anomalous moisture transport shows a slight equatorward shift during 2021–2023 relative to its long-term mean, and this result is further supported by an independent water-tracer dataset^47^ (Fig. 4b). This anomaly is mainly attributed to the EA dipole circulation, which represents reorganized regional storm tracks and AR pathways and favours moisture transport from the mid-latitude Indian Ocean into QW (Extended Data Fig. 5a,b,e,f). It is noteworthy that evaporation over the key source regions (zones 13–15) is not significantly enhanced during 2021–2023 (Extended Data Fig. 10e). This indicates that the primary factor determining precipitation over QW is not the total amount of moisture evaporated from the source regions, but rather the orientation of the large-scale circulation that sets up the transport pathways and trajectories of ARs and storms, determining where this moisture is ultimately delivered.

During 2011–2020, QW experienced a prolonged precipitation deficit (Fig. 1f–h), coinciding with cooling conditions in the TWP (Extended Data Fig. 10a), in contrast to the SST warming observed during the 2021–2023 event (Extended Data Fig. 2b). The corresponding 10-year mean circulation anomalies also show an opposite large-scale atmospheric circulation configuration, characterized by low pressure over EA and high pressure over southern Australia (Extended Data Fig. 10b). This circulation pattern suppresses poleward moisture transport from the mid-latitudes and reduces moisture convergence over QW. Consistent with this suppressed transport, moisture tagging confirms that the precipitation deficit is driven by a substantial decrease in moisture contributions from these key mid-latitude Indian Ocean regions (Fig. 4c). The contrasting SST, circulation and moisture-source anomaly patterns between the 2011–2020 and 2021–2023 periods further support the key role of TWP SST variability in modulating QW precipitation through the reorganization of large-scale circulation and moisture-transport pathways.

## Discussion

**Fig. 5:**
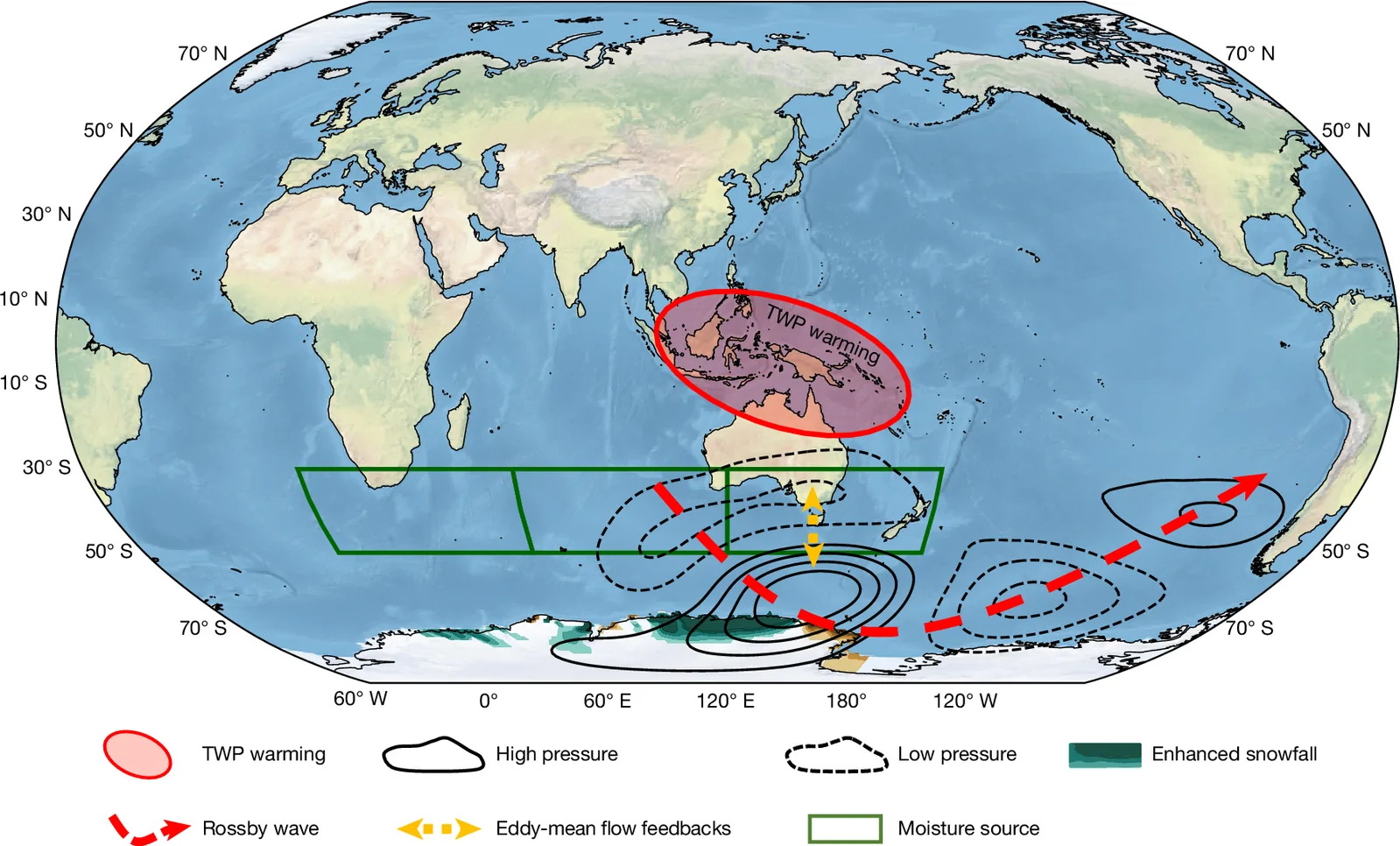
Schematic of the TWP-warming-driven teleconnection regulating Antarctic mass gain. TWP warming (red oval) excites a poleward-propagating Rossby-wave train (red dashed arrows), inducing a dipole circulation characterized by a high-pressure anomaly over EA (solid black contour) and an upstream low-pressure anomaly (dashed black contour). The high-pressure anomaly is dynamically reinforced and maintained by eddy-mean flow feedbacks (orange dashed double arrow). This circulation pattern facilitates efficient moisture transport from subtropical source regions (green boxes) towards QW by means of ARs, resulting in enhanced snowfall (green shading) and surface mass accumulation. The circulation and precipitation patterns are derived from MCA3.

**Extended Data Fig. 11:**
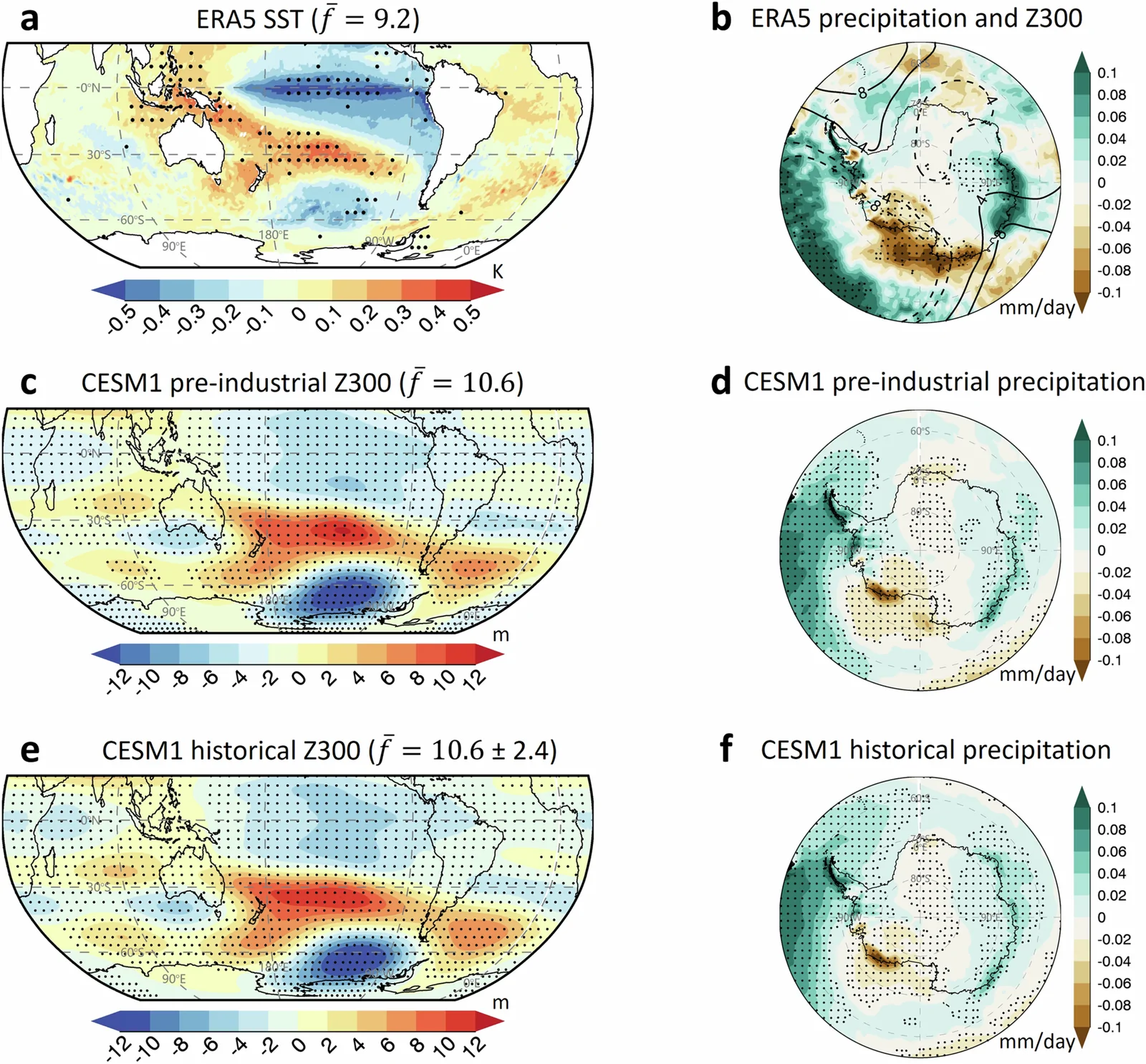
Composite anomalies during TWP warming events in observations and model simulations. **a**, **b**, Composites based on TWP warming events identified using ERA5 SST for 1950–2025. SST anomalies (a). Precipitation anomalies overlaid with Z300 contours (b). **c**, **d**, Composites based on events identified in the 1,800-year CESM1 pre-industrial control run. Z300 anomalies (c). Precipitation anomalies (d). **e**, **f**, As in **c**, **d**, but for CESM1 historical simulations for 1920–2005. TWP warming events are identified from the cumulative TWP SST anomaly index after removing the EMD residual, which represents the long-term trend. SST, precipitation and Z300 composites are calculated from deseasonalized monthly anomalies, with the component associated with the EMD residual of the TWP SST index removed by linear regression. Stippling indicates regions significant at the 95% confidence level. Values in parentheses in the left-column panel titles indicate the mean frequency of TWP warming events in events per century. For CESM1 historical simulations, the uncertainty denotes the inter-member standard deviation of event frequency.

**Extended Data Fig. 12:**
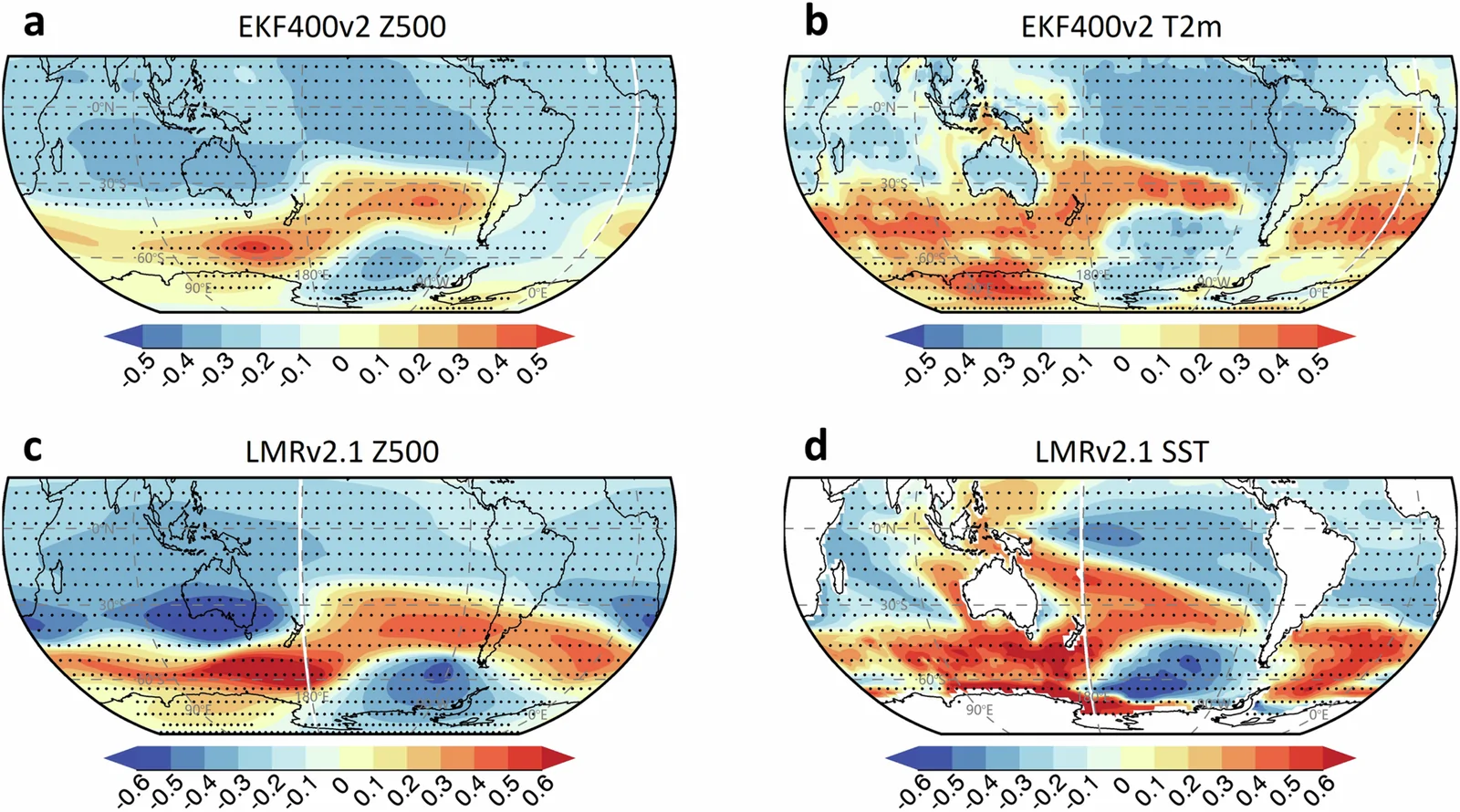
Annual-mean correlations between the QW precipitation index and large-scale climate fields in paleo-reanalysis. **a**, Correlation between the QW precipitation index and Z500 anomalies in EKF400v2. **b**, As in **a**, but for EKF400v2 2-m air temperature. **c**, As in a, but for Z500 anomalies in LMR v2.1. **d**, As in **c**, but for LMR v2.1 SST. EKF400v2 is a monthly paleo-reanalysis spanning 1603–2003, assimilating early instrumental observations, documentary indices, and tree-ring proxies using a Kalman-filtering approach. Monthly fields were averaged to annual means before the correlation analysis. LMR v2.1 is the Last Millennium Reanalysis, which combines proxy records with climate-model information through ensemble data assimilation. Because LMR v2.1 is annual and EKF400v2 lacks SST, these paleo-reanalyses cannot recreate the TWP-event composite analysis shown in Extended Data Fig. 11. We therefore use correlations to assess whether the relationships among QW precipitation, the EA dipole, and tropical SST variability persist over longer periods. To reduce the influence of long-term nonlinear trends, we divide each dataset into multiple non-overlapping 40-year segments. Within each segment, the QW precipitation index and field were linearly detrended before the correlation analysis, and the correlation coefficients were then averaged across all segments. The QW precipitation index is defined as the area-weighted mean precipitation over 70°E–145°E, 65°S–75°S. Stippling indicates regions significant at the 95% level based on an FDR-corrected Student’s t test. These results show that enhanced QW precipitation has covaried with a similar TWP–EA teleconnection over the past few hundred to two thousand years. In addition, the Law Dome ice-core record further supports this link. During 1950–2010, mean snow-accumulation anomalies are positive (+ 0.42 standard deviation) when EA dipole index (Methods) exceeds +1 standard deviation, and negative (− 0.43 standard deviation) when the index falls below −1 standard deviation.

This study demonstrates that the pronounced Antarctic mass gain during 2021–2023, dominated by accumulation in the QW region of EA, was favoured by a recurrent tropical–extratropical teleconnection driven by multiyear TWP SST warming (Fig. 5 and Methods), with other tropical modes and anthropogenic forcing acting more as secondary modulators of the QW mass change than as the immediate drivers. The recurrence of this mode is further assessed over longer observational and model-simulation records using an objective index of prolonged TWP SST warming, with events defined as a cumulative TWP SST anomaly increase of 4.0 K or more over 36 months (Methods). The results show that such events tend to occur about once per decade, and are consistently associated with a clear TWP–EA teleconnection across both observations and simulations (Extended Data Fig. 11). Paleo-reanalyses (EKF400v2 and LMR v.2.1) and the Law Dome ice-core record further support this TWP–EA linkage, showing covariability among TWP anomalies, the EA dipole and QW precipitation (Extended Data Fig. 12).

**Extended Data Fig. 13:**
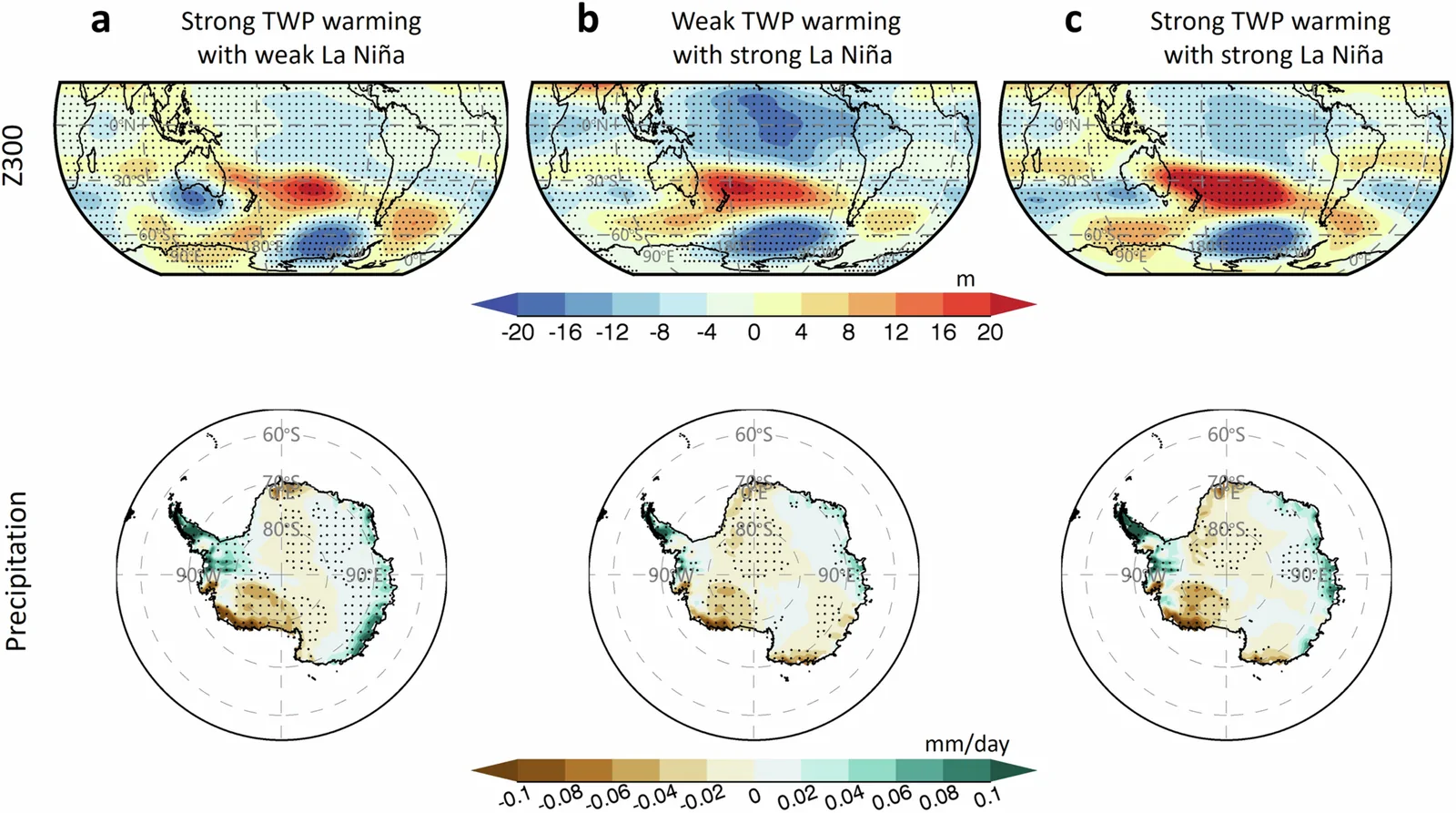
Composite Z300 and precipitation anomalies under different TWP and La Niña states in the CESM1 pre-industrial simulation. **a**, Strong TWP warming and weak La Niña. **b**, Weak TWP warming with strong La Niña. **c**, Concurrent strong TWP warming and strong La Niña. The TWP and Niño3.4 indices are derived from monthly SST. All anomalies are calculated relative to the full-period monthly climatology. Strong TWP warming is defined as TWP > 1 standard deviation, strong La Niña as Niño3.4 <−1 standard deviation, weak La Niña as −1 < Niño3.4 < 0 standard deviation, and weak TWP warming as 0 < TWP < 1 standard deviation. Stippling indicates regions significant at the 95% level based on an FDR-corrected Student’s t test. Additional tests comparing the effects of TWP warming and IOD-like cooling also show the dominant role of TWP warming (not shown).

The mechanism that triggers and maintains prolonged TWP warming remains unclear. The prolonged event from 2021 to 2023 may be linked to concurrent or preceding La Niña and negative IOD, or modulated by the Interdecadal Pacific Oscillation (IPO). Over the past four decades, the IOD, ENSO and IPO are moderately correlated with TWP SST (*r* = −0.48, −0.41, −0.45, respectively; *P* < 0.05 for all). Its correlations with the Southern Annular Mode and zonal wave-3 indices are weak and insignificant, but we cannot rule out the possibility that the dipole pattern still retains some influence from these two modes near EA. Lead-lag relationships indicate that tropical Atlantic SST warming precedes TWP SST warming by about 6 months, which subsequently leads to a La Niña-type pattern by 3 months (not shown), possibly through tropical atmospheric waves and associated atmosphere–ocean feedbacks^48^. These relationships suggest that TWP warming is shaped by complex inter-basin interactions, but our AGCM experiments indicate that the local TWP SST anomaly provides the more direct forcing for the EA dipole and QW precipitation response. La Niña and the IOD may contribute through indirect pathways, such as cooling across the entire tropical region and thereby increasing the relative strength of SST warming over the TWP. To further distinguish the relative roles of TWP SST warming and La Niña forcing in shaping the EA dipole, we use the long CESM1 pre-industrial simulation to demonstrate that the EA high-pressure anomaly and enhanced QW precipitation are more closely tied to strong TWP warming than to strong La Niña or negative IOD cooling (Extended Data Fig. 13). This analysis also shows that TWP warming, La Niña and IOD-like cooling do not always co-occur.

Previous research on tropical–Antarctic teleconnections has focused largely on ENSO-related pathways, especially the connections between central-eastern tropical Pacific SST forcing and West Antarctica climate^31^. These pathways remain predominant, operating through both direct Rossby-wave forcing and their influence on the Walker and Hadley circulations^33^. However, the tropics can affect the climate of different Antarctic regions through many pathways. Here we identify the TWP–EA teleconnection, which acts as a recurrent climate driver, episodically producing precipitation extremes and associated mass gain or loss over QW and, at times, broader EA. Thus, the recent slowdown may represent a temporary halt in continued Antarctic mass loss that remains strongly affected by ocean-induced basal melt and dynamic ice loss. The duration of this temporary slowdown will probably depend on the combined effects of this teleconnection mode, ocean-induced basal melt and dynamic thinning around the continent in the coming decades, especially in West Antarctica and vulnerable EA outlet glacier sectors such as Totten Glacier. How this teleconnection will change in the future remains uncertain, and its influence on Antarctic precipitation may be amplified in a warming world through changes in high-latitude moisture, AR intensity and storm tracks. Accurately representing this teleconnection in climate models is therefore essential for predicting Antarctic hydroclimate variability and improving sea-level projections.

## Methods

### Data

Ice mass variability was quantified using GRACE and GRACE-FO gravimetry data^50^ spanning 2003–2024. SMB (precipitation minus meltwater runoff, sublimation, evaporation and wind erosion) outputs from RACMO2.4p1 (ref. ^51^) and MARv3.14 (refs. ^52,53^) were used to link precipitation variability to observed ice mass changes. Precipitation variability was assessed using several independent datasets, including ERA5 (ref. ^54^) and MERRA-2 (ref. ^55^) reanalyses and RACMO2.4p1 regional climate-model precipitation, to ensure consistency across data sources. The Law Dome ice-core snow-accumulation record^49^ was used as extra observational input. Large-scale circulation and moisture transport were analysed primarily using ERA5 fields, including SST, Z300, evaporation and integrated water vapour transport (IVT). Velocity potential was obtained from NCEP-NCAR Reanalysis 1. The Niño 3.4, IOD, IPO, zonal wave-3 and Southern Annular Mode^56^ indices were used to examine their relationships with TWP variability. To assess the frequency of TWP warming events, we analysed both ERA5 (1950–2025) and the CESM1 Large Ensemble^57^, including the 1,800-year fully coupled pre-industrial control simulation and the 40-member historical and RCP8.5 simulations. To provide an independent long record check of the TWP–East Antarctic dipole–QW precipitation relationship, we also used two paleo-reanalysis products. EKF400v2 (ref. ^58^) provides monthly fields of Z500, 2-m air temperature and precipitation from 1603 to 2003. LMR v.2.1 (ref. ^59^) provides last-millennium ensemble reanalysis fields of Z500, SST and precipitation on an annual basis. Because EKF400v2 does not provide SST, its 2-m air temperature field was used as an indicator of SST variability. Monthly anomalies were generally calculated by removing the climatological seasonal cycle.

Guided by the spatial pattern of mass and cumulative precipitation anomalies, QW was defined as the region spanning 70–145° E and 65–75° S. The TWP was defined as a 15° clockwise-rotated ellipse centred at 135° E, 5° S, with semi-major and semi-minor axes of 35° and 15°, respectively, on the basis of the observed relationships between EA precipitation and tropical SSTs. We also defined an EA dipole index as the area-mean Z300 anomaly over the EA high-pressure centre (55°–70° S, 120°–160° E) minus that over the low-pressure centre south of Australia (30°–50° S, 80°–150° E).

### Significance testing

Statistical significance of correlations, regressions and trends was assessed using two-tailed Student’s *t*-test at the 95% confidence level, with the effective sample size corrected for autocorrelation.

1 $${N}^{* }=N\frac{1-{r}_{1}{r}_{2}}{1+{r}_{1}{r}_{2}}$$

Here *N* is the total number of samples, and *r*_1_ and *r*_2_ are the lag-1 autocorrelation coefficients of the two correlated time series. For spatial fields, statistical significance was further controlled using false discovery rate correction^60^.

### MCA decomposition

MCA^61^ is a multivariate statistical technique that uses singular value decomposition on the cross-covariance matrix of two fields to extract paired spatial patterns and time series that maximize their covariance. Each MCA mode represents a linear combination of the original variables that captures a specific covarying structure shared by the two fields. The importance of each mode is measured by the squared covariance fraction, which indicates the proportion of total squared covariance explained by that mode. Higher squared covariance fraction values denote leading modes of coupled variability. In this study, MCA was applied to deseasonalized and detrended monthly anomalies of ERA5 Z300 south of 23° N and Antarctic precipitation for 1979–2024.

### WAF diagnosis

Stationary Rossby-wave propagation was diagnosed using the Takaya and Nakamura^62^ WAF formulation. Horizontal WAF vectors (**W**) were computed from geopotential height anomalies relative to the climatological mean flow:

2 $$\begin{array}{c}{{\bf{W}}}_{{\rm{h}}}=\frac{p\cos \phi }{2|{\bf{U}}|}\\ \left(\begin{array}{c}\frac{U}{{a}^{2}{\cos }^{2}\phi }\left[{\left(\frac{{\rm{\partial }}{\psi }^{{\prime} }}{{\rm{\partial }}\lambda }\right)}^{2}-{\psi }^{{\prime} }\frac{{{\rm{\partial }}}^{2}{\psi }^{{\prime} }}{{\rm{\partial }}{\lambda }^{2}}\right]+\frac{V}{{a}^{2}\cos \phi }\,\left[\frac{{\rm{\partial }}{\psi }^{{\prime} }}{{\rm{\partial }}\lambda }\frac{{\rm{\partial }}{\psi }^{{\prime} }}{{\rm{\partial }}\phi }-{\psi }^{{\prime} }\frac{{{\rm{\partial }}}^{2}{\psi }^{{\prime} }}{{\rm{\partial }}\lambda {\rm{\partial }}\phi }\right]\\ \frac{U}{{a}^{2}\cos \phi }\left[\frac{{\rm{\partial }}{\psi }^{{\prime} }}{{\rm{\partial }}\lambda }\frac{{\rm{\partial }}{\psi }^{{\prime} }}{{\rm{\partial }}\phi }-{\psi }^{{\prime} }\frac{{{\rm{\partial }}}^{2}{\psi }^{{\prime} }}{{\rm{\partial }}\lambda {\rm{\partial }}\phi }\right]+\frac{V}{{a}^{2}}\left[{\left(\frac{{\rm{\partial }}{\psi }^{{\prime} }}{{\rm{\partial }}\phi }\right)}^{2}-{\psi }^{{\prime} }\frac{{{\rm{\partial }}}^{2}{\psi }^{{\prime} }}{{\rm{\partial }}{\phi }^{2}}\right]\end{array}\right)\end{array}$$

where ${\psi }^{{\prime} }$ denotes the geostrophic streamfunction anomaly derived from the geopotential height (*Z*), defined as ${\psi }^{{\prime} }=g{Z}^{{\prime} }/f$ (where *g* is gravitational acceleration and *f* is the Coriolis parameter); **U** = (*U*, *V*) represents the climatological mean horizontal wind vector with a magnitude of $|{\bf{U}}|=\sqrt{{U}^{2}+{V}^{2}}$. The coordinates are given by longitude (*λ*) and latitude (*φ*). In addition, *a* represents the Earth’s radius and *p* denotes the pressure level (normalized by 1,000 hPa).

### AR detection

Monthly AR-frequency anomalies were regressed onto standardized MCA3 Z300 time series to diagnose their relationship with the dipole circulation. ARs were identified using the Guan and Waliser^63^ detection algorithm. This updated and validated version is particularly suitable for high-latitude regions such as Antarctica, where background moisture is climatologically low. Its effectiveness in these regions is attributed to its use of season-dependent and location-dependent IVT thresholds, instead of a fixed global threshold, allowing for the robust identification of poleward moisture intrusions relative to the local background state.

The detection was applied to 6-hourly IVT fields derived from 6-hourly wind and specific humidity integrated from 1,000 hPa to 300 hPa:

3 $$\mathrm{IVT}\,=\,\frac{1}{g}\sqrt{{\left({\int }_{1,000}^{300}{uq}{\rm{d}}p\right)}^{2}+{\left({\int }_{1,000}^{300}{vq}{\rm{d}}p\right)}^{2}}$$

where *g* is the gravitational acceleration, *u* and *v* are the zonal and meridional wind components and *q* is the specific humidity. The detection process involved three key criteria^63^: (1) intensity threshold: the IVT magnitude had to exceed the 85th percentile of the local monthly IVT climatology, or 100 kg m^−1^ s^−1^, whichever was larger; (2) geometry: the detected filament had to have a length greater than 2,000 km and a length-to-width ratio greater than 2 and (3) directional coherence: the mean IVT direction had to be consistent to exclude moisture features without coherent poleward transport. Monthly AR frequency was calculated by averaging the daily occurrence counts within each month, with daily counts derived from the 6-hourly detections. We note that the Guan and Waliser^63^ detection algorithm tends to sufficiently capture ARs over the Southern Ocean, but may be less effective at capturing ARs after landfall^34,37^. We adopted it here because our analysis required a consistent AR detection method across the SH, rather than a regional algorithm.

### Eddy vorticity budget diagnosis

To diagnose the dynamical processes responsible for the formation and maintenance of the meridional dipole between the southern Australian low and the EA high, we analysed the relative vorticity budget following the framework of ref. ^64^. The relative vorticity tendency at a given pressure level is given by ref. ^65^:

4 $$\frac{\partial \zeta }{\partial t}=-\nabla \cdot [(\zeta +f){\bf{u}}]-\omega \frac{\partial \zeta }{\partial p}+\hat{{\bf{k}}}\cdot \frac{\partial {\bf{u}}}{\partial p}\times \nabla \omega +F$$

where *ζ* is relative vorticity, *f* is the Coriolis parameter, **u** = (*u*, *v*) is the horizontal wind vector, *ω* is vertical velocity in pressure coordinates and *F* represents frictional forcing.

Using standard scaling arguments^65^, the vertical advection and tilting terms are at least one order of magnitude smaller than the horizontal vorticity flux convergence and are therefore neglected. Each variable is decomposed into a seasonal-mean component (denoted by an overbar) and an anomaly (denoted by a prime). The anomalous vorticity tendency equation can then be written as^64^:

5 $$\frac{\partial {\zeta }^{{\prime} }}{\partial t}=\mathop{\underbrace{[-(\bar{\zeta }+f)\nabla \cdot {{\bf{u}}}^{{\prime} }-{\zeta }^{{\prime} }\nabla \cdot \bar{{\bf{u}}}]}}\limits_{{\rm{stretching}}\,{\rm{term}}}+\mathop{\underbrace{[-\nabla \cdot ({\zeta }^{{\prime} }{{\bf{u}}}^{{\prime} })]}}\limits_{{\rm{eddy}}\,{\rm{term}}}+\mathop{\underbrace{[-{{\bf{u}}}^{{\prime} }\cdot \nabla (\bar{\zeta }+f)-\bar{{\bf{u}}}\cdot \nabla {\zeta }^{{\prime} }]}}\limits_{{\rm{wave}}\,{\rm{term}}}+\mathop{\underbrace{\{-\nabla \cdot [\bar{{\bf{u}}}(\bar{\zeta }+f)]\}}}\limits_{{\rm{climatological}}\,{\rm{term}}}+{F}^{{\prime} }$$

The first term represents vorticity generation by anomalous divergence and is referred to as the stretching term. The second term denotes the convergence of anomalous vorticity fluxes and is referred to as the eddy forcing term, which captures the dynamical feedback between transient eddies (periods shorter than 7 days) and the low-frequency circulation. The third term is the linear wave term, describing the advection of background vorticity by anomalous winds and advection of anomalous vorticity by the mean flow, and is associated with Rossby-wave propagation. The fourth term consists solely of climatological quantities and represents stationary-wave forcing; it varies weakly in time and was therefore not considered in the temporal correlation analysis.

### Sensitivity experiments

To assess the role of TWP SST anomalies in driving the precipitation over EA, we conducted a suite of sensitivity experiments using two AGCMs^66^, ECHAM5 and CAM5. The ECHAM5 model was run at T42 spectral resolution (roughly 2.8° × 2.8°) with 19 vertical levels extending to 10 hPa, following a standard AMIP-style configuration. A 40-year control simulation was first performed using monthly climatological SSTs and sea ice concentrations derived from ERA5 for 1979–2024. Greenhouse gas concentrations and aerosols were fixed at year-2000 levels, thereby excluding transient anthropogenic forcing.

A total of eight sensitivity experiments were conducted by superimposing prescribed SST anomalies onto the control SST climatology. Each experiment was integrated for 40 years, and the atmospheric response was defined as the difference between the sensitivity experiment and the control simulation. Experiment 1 imposed a positive SST anomaly over the TWP. The TWP region was defined as a 15° clockwise-rotated ellipse centred at 135° E, 5° S, with semi-major and semi-minor axes of 35° and 15°, respectively. Experiment 2 combined the TWP warming with a negative IOD-like cooling anomaly (ellipse centred at 65° E, 5° N; axes 30° × 15°) and a La Niña-like cooling anomaly over the eastern Pacific (ellipse centred at 130° W, 0° N; axes 60° × 15°). Experiments 3 and 4 were designed to isolate the effects of the cooling anomalies: experiment 3 was driven solely by the La Niña-like cooling in the eastern Pacific and experiment 4 was driven solely by the negative IOD cooling in the northwestern Indian Ocean. Experiment 5 was driven by positive SST anomalies over the SPCZ region (a 10° clockwise-rotated ellipse centred at 200° E, 25° S, with semi-major and semi-minor axes of 60° and 8°, respectively) to examine the impact of SPCZ-related warming. Experiment 6 examined the modulation by background warming by superimposing the TWP SST anomaly onto a uniform +0.5 K SST increase applied across the tropical band (30° S–30° N). Experiment 7 was identical to experiment 6 but intensified the TWP warming magnitude by an extra 0.5 K. Experiment 8 replicated the forcing configuration of experiment 1 but used the CAM5 model to verify the robustness of the results and rule out model dependence.

For standard warming or cooling scenarios (experiments 1–6 and 8), anomalies increased from ±0.5 K at the boundaries to a peak amplitude of ±1 K at the centre. For the strong TWP warming case (experiment 7), the anomaly ranged from 1 K at the edge to 1.5 K at the centre.

As observed in both the SST anomaly pattern associated with the MCA3 mode (Extended Data Fig. 5c) and the mean SST anomalies during 2021–2023 (Extended Data Fig. 2b), La Niña-like SST cooling, negative IOD cooling and SPCZ warming co-occurred with the prolonged TWP warming. When both cooling patterns were imposed alongside TWP warming (Experiment 2, Extended Data Fig. 8a), the Z300 response showed a more localized low-pressure anomaly over southern Australia and a more pronounced high-pressure anomaly centred north of the Ross Sea, accompanied by a precipitation response over EA comparable to that in experiment 1. By contrast, experiments 3–5, which isolated the La Niña-like cooling, IOD-like cooling and SPCZ warming, respectively (Extended Data Fig. 8b–d), failed to reproduce the significant EA dipole circulation pattern or positive precipitation anomalies near QW, suggesting that these three SST anomaly components were not the key forcings driving the observed response.

In a future warming world, tropical mean SSTs are expected to increase, as is evident in CESM1 future projections. The 30-year mean SST during 2021–2050 is warmer than that during 1991–2020 by about 0.5 K across nearly the entire tropics (not shown). How this background warming modulates the circulation response to TWP warming was illustrated with experiment 6, representing the occurrence of TWP anomalies within the warmer background state expected in the coming decades. Under this forcing, the Z300, surface temperature and precipitation responses over EA were notably less significant (Extended Data Fig. 8e). This attenuated response probably resulted from the uniform tropical warming reducing the relative SST gradient between the TWP and its surrounding regions, thereby damping the effective convective heating anomaly over the TWP. When the magnitude of the TWP warming was increased (experiment 7), the response over EA became pronounced again, aligning closely with the results of experiment 1 (Fig. 3 and Extended Data Fig. 8f). This indicates that the atmospheric circulation over EA is preferentially sensitive to the localized SST gradients driven by TWP warming, rather than to broad, basin-wide tropical warming.

Finally, experiment 8 repeated the experiment 1 forcing using CAM5, performed in the same AMIP-style framework at 1.9° × 2.5° horizontal resolution with 30 vertical levels, and reproduced a north–south dipole circulation pattern and enhanced precipitation over EA similar to those simulated by ECHAM5 (Extended Data Fig. 9), confirming that the results were not model dependent.

### Precipitation source tracing

Moisture-source attribution was quantified using the isotope-enabled CESM1.2 (iCESM1.2) with the finite-volume dynamical core at 1.9° × 2.5° resolution. This water-tagging and circulation-nudging configuration provided a new way to trace moisture sources and transport pathways under observed circulation and temperature conditions. Monthly SSTs and sea ice concentrations were prescribed from ERA5. To constrain the large-scale circulation, the model atmosphere was fully nudged towards ERA5 following the NCAR DART framework. Horizontal winds and temperature throughout the column, together with near-surface specific humidity, were relaxed towards ERA5 fields with a 6-h timescale:

6 $$\frac{{\rm{d}}x}{{\rm{d}}t}={F}_{\mathrm{model}}(x)+\alpha \frac{{x}_{\mathrm{ERA}5}-x}{\tau }$$

where *F*_model_ denotes the internally generated tendencies from the model’s physics and dynamics, *τ* is the nudging timescale (6 h, matching the reanalysis update frequency) and *α* = 1 corresponds to full nudging. This configuration constrains the circulation while allowing the hydrological cycle and isotopic fractionation to evolve freely.

The moisture tracing implementation followed the isotope-enabled Community Atmosphere Model framework in iCESM, where water tagging is achieved by reusing the existing isotope moisture-tracer infrastructure. Each tag was treated as an isotope tracer, but without isotopic fractionation. The model simulated all moist processes (convection, cloud microphysics, condensation, precipitation formation and re-evaporation) identically to the standard Community Atmosphere Model for each tagged component in water vapour, cloud liquid and ice, and precipitation. Within this framework, 54 geographically defined surface regions were tagged as distinct moisture sources. Evaporated water vapour from each region was tracked through transport and phase changes without exerting radiative or dynamical feedback. Contributions from each source to QW precipitation were quantified for the climatological mean, the 2021–2023 precipitation anomaly and the contrasting 2011–2020 deficit. We tracked only the immediate, direct contribution that supplied moisture for the most recent precipitation.

### Mechanism of the TWP–EA teleconnection

By integrating analyses of satellite gravimetry and reanalysis data, water-tagging and nudging-enabled simulations and AGCM experiments, we identified a mechanism linking TWP SST anomalies to a persistent high-latitude circulation anomaly that enhances poleward moisture transport and precipitation over QW, as exemplified by the abrupt mass gains during the 2021–2023 event and by the decade-long mass loss from 2011 to 2020. Prolonged TWP warming serves as an efficient source of upper-tropospheric divergence and Rossby-wave activity, initiating a poleward-propagating wave train that reaches high southern latitudes. This wave train establishes a meridionally oriented dipole with a high-pressure anomaly over the EA coast that is dynamically reinforced by eddy-mean flow feedbacks. This high-pressure anomaly tends to favour EA blocking activity, enhancing moisture transport and precipitation near EA. The associated precipitation increases over QW are induced mainly by circulation-driven moisture transport from subtropical sources and AR-related extremes (Extended Data Fig. 5e,f), not by enhanced local evaporation.

### EMD

Because long-term SST time series spanning more than a century may contain nonlinear trends, we applied empirical mode decomposition (EMD)^67^ to remove such nonlinearity. EMD is a fully adaptive and data-driven method that decomposes a signal into a finite set of intrinsic mode functions (IMFs) representing oscillatory variability across distinct timescales, without requiring predefined basis functions. Each IMF satisfied two criteria: (1) the number of local extrema and zero crossings differed by at most one over the entire record, and (2) the mean of the upper and lower envelopes defined by local maxima and minima was zero at any point. Using EMD, the original signal *X*(*t*) is expressed as:

7 $$X(t)=\mathop{\sum }\limits_{i=1}^{L}{{\rm{IMF}}}_{i}(t)+r(t)$$

where earlier IMFs capture higher-frequency variability, subsequent IMFs represent progressively lower-frequency oscillations and the residual *r*(*t*) represents the long-term background trend after all IMFs are extracted.

### Decadal recurrence of multiyear TWP warming

The MCA and modelling experiments supported the hypothesis that TWP SST anomalies play a key role in regulating precipitation variability over EA. However, the extent to which this mechanism contributed to earlier events before the GRACE observational period remained unclear. Before 2003, another increase in QW precipitation (MCA3) occurred during 2000–2002 (Fig. 2e, green line), showing a similar but weaker teleconnection pattern compared with the 2021–2023 event (Extended Data Figs. 2a,b and 10c,d). By contrast, the 10-year (2011–2020) precipitation deficit (Fig. 1b) was characterized by circulation and SST patterns in the opposite phase (Extended Data Fig. 10a,b). These contrasting periods highlight the role of TWP SST variability in modulating QW precipitation on decadal timescales. Thus, TWP SST anomalies may serve as a useful indicator for identifying large-scale climate conditions favourable for QW precipitation anomalies.

Motivated by the consistent coincidence of step-like increases in cumulative TWP SST anomalies with prolonged QW precipitation extremes, a cumulative SST-based criterion was adopted to identify prolonged TWP warming events. On the basis of the magnitude and duration of the TWP SST anomalies during the two recent events (2000–2002 and 2021–2023), we defined prolonged TWP warming events using the cumulative TWP SST anomaly index. An event was identified when the index rose by at least 4 K within a 36-month window. The event began at the preceding cumulative minimum. After this threshold was reached, the cumulative index was tracked until it decreased by 0.4 K from its subsequent maximum; the month of this maximum was defined as the event end. We applied this criterion to ERA5 for 1950–2025, the CESM1 pre-industrial control and historical ensemble. Because these long-term observational and simulated time series contained complex low-frequency variability, we removed the EMD residual, which represents the long-term trend of the input time series, before detecting TWP events. This step allowed the detected events to represent prolonged TWP anomalies relative to the evolving long-term background. Without this step, it was difficult to distinguish isolated and independent TWP events from background SST changes.

During the observational period, ERA5 (1950–2025) yielded an estimated frequency of 9.2 prolonged TWP warming events per century (Extended Data Fig. 11a). The ERA5 SST composite for these events showed a clear TWP warming pattern, indicating that events similar to those in 2021–2023 were not unprecedented in the historical record and may occur on interdecadal timescales. The corresponding ERA5 composite further showed a consistent EA high-pressure anomaly and enhanced precipitation over QW (Extended Data Fig. 11b), closely resembling the observed circulation and precipitation anomalies during the 2021–2023 event (Fig. 1e).

To further assess the recurrence frequency of this teleconnection in a longer model simulation, we used a 1,800-year CESM1 pre-industrial control simulation, which yielded a frequency of 10.6 TWP warming events per century (Extended Data Fig. 11c). Composite analysis confirmed that TWP warming events were accompanied by significant large-scale atmospheric circulation anomalies and positive precipitation anomalies over QW (Extended Data Fig. 11c,d), closely resembling the 2021–2023 pattern (Fig. 1e). The CESM1 historical simulations (1920–2005) produced a comparable mean frequency of 10.6 ± 2.4 events per century and similar composite patterns (Extended Data Fig. 11e,f). These results indicate that the roughly decadal recurrence of the TWP–EA teleconnection is a consistent feature across observations and model simulations.

## Online content

Any methods, additional references, Nature Portfolio reporting summaries, source data, extended data, supplementary information, acknowledgements, peer review information; details of author contributions and competing interests; and statements of data and code availability are available at https://doi.org/10.1038/s41586-026-10912-x.

## Supplementary information


Peer Review File


## Data Availability

The processed datasets and intermediate files generated during this study, including those required to reproduce the main and extended data figures, are publicly available at Figshare (https://doi.org/10.6084/m9.figshare.31596730)^68^. Antarctic mass change data are based on the CSR RL06 Mascon solutions derived from the GRACE and GRACE-FO missions, available through the University of Texas at https://www2.csr.utexas.edu/grace/RL06_mascons.html. The RACMO2.4p1 SMB and precipitation data are available at Zenodo (https://doi.org/10.5281/zenodo.19255213)^69^. The MARv3.14 SMB data are available at http://ftp.climato.be/fettweis/MARv3.14/Antarctica/. ERA5 precipitation, evaporation, SST and northward IVT data are available through the ECMWF ERA5 single levels dataset at https://cds.climate.copernicus.eu/datasets/reanalysis-era5-single-levels-monthly-means?tab=download, and Z300 and wind vectors are available through the ERA5 pressure levels dataset at https://cds.climate.copernicus.eu/datasets/reanalysis-era5-pressure-levels-monthly-means?tab=download. MERRA-2 precipitation data are available through NASA at https://disc.gsfc.nasa.gov/datasets/M2TMNXFLX_5.12.4/summary?keywords=tavgM_2d_flx_Nx. The CESM1-LE dataset, including the historical simulations, RCP8.5 future projections and pre-industrial control simulations, is available through the NCAR community project website at https://www.cesm.ucar.edu/community-projects/lens. Velocity potential data from NCEP-NCAR Reanalysis 1 are available through NOAA Physical Sciences Laboratory (PSL) at https://psl.noaa.gov/data/gridded/data.ncep.reanalysis.derived.html. The Niño 3.4 index is available through NOAA PSL at https://psl.noaa.gov/data/timeseries/month/DS/Nino34_CPC/, the IOD index is available through NOAA PSL at https://psl.noaa.gov/data/timeseries/month/DS/DMI/ and the IPO index is available through NOAA PSL at https://psl.noaa.gov/data/timeseries/IPOTPI/. The Law Dome ice-core snow-accumulation record is available through the UK Polar Data Centre at https://ramadda.data.bas.ac.uk/repository/entry/show?entryid=cc1d42de-dfe6-40aa-a1a6-d45cb2fc8293. The EKF400v2 paleo-reanalysis is available through the World Data Center for Climate at https://doi.org/10.26050/WDCC/EKF400_v2.0. The LMR v.2.1 data are available through NOAA/NCEI Paleoclimatology at https://doi.org/10.25921/gn22-5866.

## Code availability

The full code stack used to produce the analyses and figures in this study is publicly available at Figshare (https://doi.org/10.6084/m9.figshare.31596730)^68^.
