# Supplementary material for: Multiyear tropical warm pool warming drives slowdown in Antarctic mass loss
Source: Nature. 2026 Aug 19;656(8129):897–904. doi: 10.1038/s41586-026-10912-x (PMC13518241; doi:10.1038/s41586-026-10912-x)
Supplement: Supplementary file 1 [file 41586_2026_10912_MOESM1_ESM.pdf]

nature portfolio

Peer Review File

Multiyear tropical warm pool warming drives slowdown in Antarctic mass loss

Corresponding Author: Professor Qinghua Ding

Any redactions in this file are there to maintain patient confidentiality, the confidentiality of unpublished data, or to remove third-party material.

This file contains all reviewer reports in order by version, followed by all author rebuttals in order by version.

Attachments originally included by the reviewers as part of their assessment can be found at the end of this file.

Version 0:

Reviewer comments:

Referee #1

(Remarks to the Author)
General comments

This study attributes the cause of the recent pause in Antarctic mass loss to a persistent tropical warm pool (TWP) located across the central to southern Pacific Ocean. Through a robust and extensive analysis, the authors argue that the existence of this TWP triggers a poleward propagating Rossby wave train that leads to a blocking high over East Antarctica which allows for the frequent poleward advection of warm, moist airmasses sourced for the mid-latitude Indian Ocean. The linking of similar TWP events in recent decades with East Antarctic snowfall trends along with the projected occurrence of similar TWP in future climate simulations marks this feature as something that is independent of the general warming and moistening trend that is projected to increase Antarctic snowfall.

The argument for this framework of the TWP controlling East Antarctic snowfall trends is compelling due to the in-depth analysis of the teleconnection. These methods include multiple reanalysis and observational datasets, maximum covariance analysis (MCA) for identifying distinct circulation modes, AMIP model experiments involving multiple atmospheric configurations with two different atmospheric general circulation models, atmospheric river detection, CESM1 Large Ensemble for assessing precipitation patterns during historical and future periods, and moisture source tracing using iCESM. Overall, the wide variety of methods employed by the authors are impressive and are well respected in the field of large-scale atmospheric dynamics.

The manuscript succeeds in convincing the reader that TWP and thus internal variabilty is driving snowfall trends across parts of East Antarctica. However, my main two issues are the narrative framing of the research problem and missing links with existing literature related to this topic. For instance, the abstract states that the recent increase in ice mass across East Antarctica, “has been seen as an early indicator of an expected long-term response to global warming, in which storm tracks shift poleward and deliver more precipitation to Antarctica”. Then the results from this study are presented as a counter to this prevailing narrative. This seems misleading as there is no existing literature that claimed this recent pause in Antarctic mass loss is the result of a long-term sustained global warming signal causing more precipitation. Of course, it is very interesting to see the authors show that the recent mass gains in East Antarctica are driven by internal variability as opposed to a forced response, but the framing of the results as something counter to a prevailing narrative seems unnecessary.

This leads to my second issue as most existing literature frames this mass gain in East Antarctica as an extreme weather topic, but extreme weather is largely not addressed in this manuscript besides correlations in atmospheric river (AR) activity with the MCA3 Z300 index. As the recently published Kolbe et al. (2026) shows in Figure 2a, the mean precipitation across the Antarctic ice sheet from 2020-2024 is marginally higher than the 2003-2019 mean, but extreme precipitation events largely drove the surface mass balance trends. This notion is backed up in other literature like Turner et al. (2019). One suggestion could be to examine the TWP conditions during the notorious extreme East Antarctica AR event in March 2022 to see how it compares with the teleconnection framework identified in this manuscript. Regardless, I feel this manuscript

needs to bridge the gap between teleconnections and extreme weather events for it to be considered holistic.

Overall, I feel this manuscript is well written and the analysis is robust and timely. On the scientific merits, I would support publication after the following concerns are addressed.

Specific comments

1. Looking at Figure 2, the MSCA precipitation index has had other spikes in the past like most recently around 2010. In this case, the mass balance in the Queen Mary Land and Wilkes Land sector (QW) area also increased, but the overall Antarctic Ice Sheet mass balance remained largely negative. How can you explain this difference in Antarctica mass balance? Was there something different about the precipitation patterns across East Antarctica or was mass loss across West Antarctica higher?
2. As seen in Fig. 1d, while the SMB changes in the QW area were the most positive, it also appears increased snowfall across Queen Maud Land and around the Antarctica Peninsula also helped to increase the Antarctic mass balance. Can the TWP also explain the increased snowfall in these regions or could this be related to another mechanism like the longer-term increased moisture signal from global warming or the sea-ice minima identified in (Kolbe et al. 2026).
3. Disentangling the TWP teleconnection: In the Discussion section, I would like to see how the proposed teleconnection is related to other teleconnections like the recent La Nina event as outlined in Wang et al. (2025). Also Marshall et al. (2017), highlighted how the PSA2 pattern (related to ENSO) can drive an enhanced moisture flux towards Wilkes Land.
4. Previous work has identified similar mechanisms where tropical SST hotspots can drive a Rossby wave train towards Antarctica and trigger extreme weather conditions (see (Clem et al. 2022). It could be nice to see a short discussion on how your methods compare with these studies and whether this concept of tropical SST hotspots triggering poleward extreme heat and precipitation is something that can be generalized across Antarctica.

5. Looking at Figure 5, the difference in TWP warming event frequency between historical and future climate projections appear minimal. Are there other ways that global warming could be superimposed on future TWP events. Like it is possible for these events to cause more snowfall across East Antarctica in the future?

Minor comments

Line 30: “Sharp mass gain” should be more specific. The increase in surface mass balance offset the ice discharge.

Line 31-32: Is this really the case? I am not aware of any discourse that stated this recent increase in snowfall was part of a long-term response to global warming. Most literature about this mass loss pause has focused on extreme weather events.

Line 66: Citation 30 deals with future atmospheric river changes. It would be more appropriate to cite Maclennan et al. (2022) to describe AR contribution to total precipitation.

Line 73: “landfalls”

Line 98-100: None of the studies cited (59-62) actually analyzed the link between teleconnections and ARs in East Antarctica. You could cite Shields et al. (2022), which found links between ARs and the Indian Ocean Dipole/ENSO.

Figure 1: I suggest reversing the colorbar for c and d so that blue is positive and red is negative

Line 128-129: According to Extended Data Fig. 1, while mass loss remained slightly negative across the rest of Antarctica (excluding QW), SMB also increased and actually follows the CESM1 simulation. This would imply that the gradual increase of moisture from anthropogenic changes is having an impact there and thus should be impacting QW as well. Is it possible that the long-term global warming trends are having an impact on other parts of Antarctica?

Line 141: “rarely observed”? please specify what this is in context to?

Figure 3c: I suggest reversing the colorbar as green is normally associated with excessive precipitation.

Line 328-331: The increased moisture sourcing from the Indian ocean subtropics follows an analysis from Wille et al. (2024) which found enhanced latent heating in this region in the days preceding AR landfalls at Dumont d’Urville station.

Line 726-727: In fact, most studies of ARs around Antarctica tend to avoid using the Guan and Waliser algorithm given its tendency to be very permissive about detecting ARs across the Southern Ocean, but not detecting ARs once they move beyond the Antarctica coastline (see Shields et al. 2022). Still, for this study, the Guan and Waliser algorithm is a reasonable choice over regional algorithms like Wille et al. (2021) given the need for analyzing AR activity across the Southern hemisphere (Extended Data Fig. 5). I suggest adding this caveat about the Guan and Waliser algorithm here.

Code availability: It would be preferable if all scripts used in this analysis were publicly available on Zenodo or Github

References:

Clem, K. R., D. Bozkurt, D. Kennett, J. C. King, and J. Turner, 2022: Central tropical Pacific convection drives extreme high temperatures and surface melt on the Larsen C Ice Shelf, Antarctic Peninsula. Nature Communications, 13, 3906,

https://doi.org/10.1038/s41467-022-31119-4.
Kolbe, M., J. A. Torres Alavez, R. Mottram, M. Katurji, R. Bintanja, and E. C. van der Linden, 2026: Atmospheric rivers and winter sea ice drive recent reversal in Antarctic ice mass loss. Communications Earth & Environment, 7, 255, https://doi.org/10.1038/s43247-026-03242-3.
Maclennan, M. L., J. T. M. Lenaerts, C. Shields, and J. D. Wille, 2022: Contribution of Atmospheric Rivers to Antarctic Precipitation. Geophysical Research Letters, 49, e2022GL100585, https://doi.org/10.1029/2022GL100585.
Marshall, G. J., D. W. J. Thompson, and M. R. van den Broeke, 2017: The Signature of Southern Hemisphere Atmospheric Circulation Patterns in Antarctic Precipitation. Geophysical Research Letters, 44, 11,580-11,589, https://doi.org/10.1002/2017GL075998.
Shields, C. A., J. D. Wille, A. B. Marquardt Collow, M. Maclennan, and I. V. Gorodetskaya, 2022: Evaluating Uncertainty and Modes of Variability for Antarctic Atmospheric Rivers. Geophysical Research Letters, 49, e2022GL099577, https://doi.org/10.1029/2022GL099577.
Turner, J., and Coauthors, 2019: The Dominant Role of Extreme Precipitation Events in Antarctic Snowfall Variability. Geophysical Research Letters, https://doi.org/10.1029/2018GL081517.
Wang, S., J. Liu, W. Cai, D. Yang, T. Kerzenmacher, S. Ding, and X. Cheng, 2025: Strong impact of the rare three-year La Niña event on Antarctic surface climate changes in 2021–2023. npj Climate and Atmospheric Science, 8, 173, https://doi.org/10.1038/s41612-025-01066-0.
Wille, J. D., and Coauthors, 2021: Antarctic Atmospheric River Climatology and Precipitation Impacts. Journal of Geophysical Research: Atmospheres, 126, e2020JD033788, https://doi.org/10.1029/2020JD033788.
——, and Coauthors, 2024: Examining Atmospheric River Life Cycles in East Antarctica. Journal of Geophysical Research: Atmospheres, 129, e2023JD039970, https://doi.org/10.1029/2023JD039970.

-Jonathan Wille

Referee #2

(Remarks to the Author)
I co-reviewed this manuscript with one of the reviewers who provided the listed reports. In addition I provide the following comments.

Summary

The work presents an insight into understanding East Antarctica precipitation variability. The authors were able to show that the recent mass gain in Queen Mary Land and Wilkes Land can be linked to a teleconnection driven by positive SST anomalies in the tropical warm pool, which triggers a Rossby wave train and induces a high pressure anomaly over East Antarctica, rather than representing a direct global warming response. This provides a useful contribution to understanding East Antarctic mass change. In general, the manuscript is well structured and follows a clear logic, but it could benefit from several comments to further strengthen its clarity and impact.

General comments
A number of established climate modes—including the Southern Annular Mode (SAM), ENSO, the Indian Ocean Dipole (IOD), and the Interdecadal Pacific Oscillation (IPO)—are known to influence Antarctic mass variability through their impacts on cyclonic and anticyclonic circulation patterns. Several of these modes also correlate with TWP in this study. In this context, it would strengthen the manuscript to clarify how the influence of these modes was accounted for when isolating the TWP contribution. It would be helpful to understand whether the roles of SAM, ENSO, IOD, and IPO can be separated from the TWP signal and whether their relative contributions to the 2021–2023 mass gain event can be quantified. This question is particularly relevant because SAM and ENSO were largely in phase during 2021–2023 (positive SAM and La Niña), which could influence the high latitude circulation response. Clarifying how these overlapping modes were treated in the analysis would help reinforce the conclusion that the observed teleconnection is uniquely attributable to TWP SST anomalies rather than a combined response from multiple climate modes.

Specific
L33: “we here show that”… Could brief describe how you did it

L40: “historical period” suggests mentioning the period

L42: “cooling phase also explains a decade-long mass loss over the same region from 2011 to 2020”.. Why not also mention the mass gain observed in the same region during 2000–2002, 2021-2023?

L42:43 “cooling phase also explains a decade-long mass loss over the same region from 2011 to 2020. Therefore, the recent Antarctic Ice Sheet (AIS) mass gain is likely temporary’”….. How likely is it that the warm phase of the TWP could produce a comparable decade long mass gain, similar to the mass loss effect attributed to the cooling phase?

L55-56: “In contrast, the East Antarctic Ice Sheet (EAIS), which contains nearly 80% of Earth's land ice, is more sensitive to precipitation-driven surface mass balance (SMB) variability” … Please add an appropriate reference to support this statement.

L123-124: “between July 2021 and April 2023, during which the AIS gained approximately 695.0 Gt of compared to that in July 2021.”… Is it possible to compare this change to the mass variations over the entire study period rather than only to July

2021?

L124-125: “As a result, the net Antarctic mass balance since 2021 has remained close to zero”… Is this conclusion derived directly from your results, or does it require a supporting reference?

L126-129: “Spatially, this mass reversal is dominated by an extreme mass gain over EA (Fig. 1c,d), with the largest accumulation concentrated in the Queen Mary Land and Wilkes Land sector (QW; 70–145°E, 65–75°S). In contrast, the integrated mass changes in other 129 Antarctic regions remain negative during the event (Extended Data Fig. 1).”…. What about the mass gain observed in the Antarctic Peninsula? Should this also be discussed or clarified?

L143–145: “This 2021–2023 precipitation anomaly is accompanied by a quasi barotropic anticyclonic circulation anomaly near EA (Extended Data Fig. 2a), embedded within an Antarctic wide low pressure anomaly produced by the mega La Niña.” This statement is somewhat ambiguous. Is East Antarctica experiencing a high pressure anomaly, a low pressure anomaly, or both simultaneously at different levels? Additionally, is the Antarctic wide low pressure anomaly attributed to the mega La Niña or SAM?

L145–147: Since the mass change is computed as April 2023 minus July 2021, is there any particular reason the contour pressure pattern was not computed to represent the mean circulation pattern during this period, but instead was done only for the 10 months of the event?

L153: In Fig. 1, panel “b QW mass cumulative precipatation” — please check the spelling of precipitation.

L191: “SH Z300 anomalies for the …” Please provide a brief explanation of the rationale for using Z300.

L193-195: “the leading two MCA modes capture circulation patterns associated with precipitation variability over WA and the Antarctic Peninsula (Extended Data 9 195 Fig. 3a–d)”…. I believe precipitation signals can also be observed in East Antarctica across all three MCA modes. However, the MCA3 pattern in QW appears closest to the observed mass change during 2021–2023. Perhaps this sentence could be rephrased to indicate that the MCA modes capture mass change patterns across Antarctica. Also, can each mode be associated with a particular large scale climate pattern?

L215-216: “We find the time series of the cumulative MCA3 precipitation pattern closely aligns with the GRACE-derived mass balance gains in the QW (Fig. 2e)”….. How close is the alignment between the precipitation pattern and GRACE for the first MCA mode, particularly the mode linking Southern Hemisphere Z300 and East Antarctic precipitation?

L231–233: “The correlation between the MCA3 Z300 time series and global SSTs further suggests a potential linkage between the dipole circulation and SST warming over the TWP, accompanied by SST cooling over the Indian Ocean and eastern Pacific (Extended Data Fig. 5c).” Again, is this result similar to what is obtained when using the first MCA mode between Southern Hemisphere Z300 and East Antarctic precipitation?

L416: I think the discussion could benefit from a comparison and contrast between the high-pressure anomaly associated with the TWP and previous studies that have examined blocking events in East Antarctica. Blocking events have been linked to East Antarctic precipitation variability, e.g., King and Turner 1997; Walsh et al. 2000; Scarchilli et al. 2011; Udy et al. 2021.

Referee #3

(Remarks to the Author)
The authors present an analysis of the causes of the previously published reduction in Antarctic mass loss over 2020-2023. This is widely understood to be driven by SMB increases. Previous work has shown that this is largely contained in the Wilkes Land region of East Antarctica. They use reanalysis and models to suggest that the origin of this signal is associated with prolonged warming of the Tropical Warm Pool, resulting in transport of moisture to East Antarctica. This explanation is contrary (or additional?) to previous explanations surrounding a triple La Nina or more localised weather systems were responsible, or more generally attributed to increased atmospheric rivers. Of these, the association with the triple La Nina is most mechanistic with these previous studies being heavily reliant on correlation analysis.

Overall, I found the manuscript to be very well written and very clear, stepping through the analysis impressively. While the modelling and broader climatological aspects are not my strength, and hence this needs to be taken with a grain of salt, I thought the modelling and attribution to TWP was convincing.

Certainly, this work is the deepest analysis so far and the most convincing of the currently published studies, due to the detailed modelling work. That said, I am not sure the authors disproved a role of the triple La Nina or even increased ARs. And maybe they don't suggest that, but it is not entirely clear what they think on that point. The TWP SSTs are shown to have modest correlations with IOD and ENSO and weaker with PDO (L406). I wonder if the cumulative indices have similar or different correlations, given that it is the cumulative effect that matters to the ice sheet, as the authors note. After all, a cumulative TWP effect is being linked to the cumulative SMB.

I note that the precip from the TWP perturbation experiments (Fig 3c) fell well to the east of the observed region and in an even more narrow region - I figure that is within the bounds of model uncertainty. This does cause me to encourage the authors to ensure their conclusions clearly pertain to quite a narrow part of EAIS, let alone the entire AIS. Of course, the

overall mass of hte ice sheet is affected.

I found the work that suggested that TWP events may have only slightly increased since the pre-industrial and will only slightly increase further in the future to be interesting. Can the authors say or speculate if these events may be getting bigger in time due to the larger moisture capacity of the atmosphere? That is, near-constant frequency but increasing cumulative SMB impact.

Finally, I found the analysis of the observational and SMB data to be generally robust, although there are a few places where refinements to the text are required, as I detail below.

More substantial remarks
L31 and in the introduction: I did not see evidence in the paper that people have actually published that this is part of an expected response to warming. Maybe there are corridor conversations, but I don't think that's enough to jump at. Certainly, the literature suggests that increased precipitation is expected with warming, but I've not seen a suggestion that this would be episodic and sudden.

L124 'since 2021' is overly loose language since it only applies to the data period being presented. so we need 'over 2021-2024'. please revise throughout if 'is' or 'since' is used.

L131, was there an unambiguous mass loss in QW before it paused? The authors show a decrease in precipitation in the decade prior, so overall mass loss from which to pause is a mischaracterisation. Also note the large uncertainties in background models (e.g., GIA models correcting GRACE are poorly constrained here) that mean GRACE rates could be biased at some level - this can be addressed through some tweaking of text.

L141 that this event is 'rarely observed' is not demonstrated, and not clear from the figure. Rarely inside 20y? That is a very short period. Or is there another analysis that shows it is rare? Of course, rare, would contradict a return period of 10y shown later. So some revision to the text is required.

L187 I don't see how a 2-3-year event can shape long-term mass variability. What am I missing?

L366 I didn't see evidence that the TWP was affecting all EA, so should this be more tightly constrained geographically?

L380 'events' are only defined in the methods. Could do with a reference to methods and a very brief summary of how an event is defined.

More minor remarks:
L42 'same regions' - not clear what regions are being referred to
L59, while this is the reconciled number, the actual individual data does not agree within their uncertainties for EAIS. This could be reflected with a variant of this text
L62 mCDW. I think you need citations here such as Rintoul et al 2025 for the Denman.
L66 'relatively stable mass balance' where is this shown? There is work that shows large natural variability over all timescales in EAIS. See e.g., King and Watson GRL 2020
Figure 1. i was surprised not to see RACMO in panels f-h given it is the superior SMB model. precip fields are available.
L165 'with QW precip exceeding one sd' -> 'where QW precip exceeding one s.d.'?
L232 I was not sure if TWP was defined to this point
L322 i thought the Kolbe et al paper coudl be cited here
L360 where do i look to see the 2000-2002 jump?
L446 add references
L447 add references
L456 i was not sure what was meant by 'more sensitive to oceanic changes'

Referee #4

(Remarks to the Author)
Review for the manuscript ‘Prolonged tropical warm pool warming drives a slowdown in Antarctic mass loss’ by Wang et al.
Wang et al. investigated the positive anomalies in Antarctic surface mass balance over the Queen Mary Land and Wilkes Land sector (QW) between July 2021 to April 2023. Based on the ERA5 reanalysis, the positive precipitation anomalies are linked to the third leading mode from a maximum covariance analysis (MCA3) between Antarctic precipitation and Z300 south of 23°N. Based on ECHAM5 simulations, the main dipole circulation patterns associated with MCA3, with a low pressure system to the south of Australia and a high pressure system close to QW, are linked to positive SST anomalies over the tropical warm pool (TWP) region. The manuscript is nicely written, and the results are presented clearly. However, I have some concerns about the novelty, the methodological approaches, the conclusion, and the implications, about which I would like to hear some thoughts from the authors. My comments are split into major and minor ones as below.
In summary, I am sceptical of the conclusion that the authors made in the title for two reasons: 1) The SST warming anomalies associated with the anomalous QW precipitation event are mainly located around the South Pacific Convergence Zone (SPCZ) rather than TWP, and the warming/cooling pattern resembles that of La Nina. The relationship between TWP/SPCZ warming and ENSO should be better clarified. 2) The causal-impact relationship between tropical SST warming

and EA precipitation anomalies that the authors propose should be further supported by and compared quantitatively to past observations (e.g. ice core records of surface mass balance from Medley and Thomas, (2018)).
Major comments:
1. I am not a teleconnection expert, but I am aware of multiple articles discussing the tropical-Antarctic teleconnection through Rossby wave dynamics (e.g. Li et al., 2021). I am not sure whether the specific pathway between TWP and QW has been documented before, but the dynamics resemble the link between the Interdecadal Pacific Oscillation (IPO) with the Amundsen Sea Low (ASL) (see Fig. 2b from (Li et al., 2021)), though the locations are displaced. I am curious what is the link between TWP warming and El Niño–Southern Oscillation (ENSO) events. Could a strong La Nina also excite a Rossby wave train as shown in Fig 2a? Are the pressure anomalies and wave activity fluxes (WAF) associated with MCA3 also found in low latitudes in Fig 2a? Based on the current presentation, the Rossby wave seems more likely emanate from Indian Ocean.
2. Surface mass balance from regional models and precipitation from global reanalyses and GPCP should be complemented by observations for quantitative analysis. Uncertainties in the GRACE/GRACE-FO estimates of QW mass balance should be reported and compared with observations as well. These observations could include surface mass balance stakes (Wang et al., 2025a), CloudSat snowfall retrievals (Palerme et al., 2017), ice core measurements (Medley and Thomas, 2018), and potential direct precipitation observations. Each of these datasets would contain their own uncertainties, but they should be helpful to further constrain the magnitude of mass balance gain during this period measured by GRACE/GRACE-FO. GPCP is normally considered not suitable for Antarctic studies, as can be seen from its large differences from other reanalyses. I understand the current datasets may be enough to showcase qualitatively the anomalous precipitation accumulation, but I think it is necessary to search for longer records to check whether similar events occurred in the past.
3. The authors link anomalous QW precipitation to MCA3, and then to TWP warming, which recurs every ~10 years based on the ERSSTv5 and CESM1. Since the authors conclude the TWP warming drives the anomalous QW precipitation, are there evidences that there are recurrent anomalous QW precipitation that slows down Antarctic mass loss. So a null hypothesis could be, the Rossby wave train induced by the TWP warming favours a high pressure system near QW, which favours moisture transport but not necessarily induces stronger moisture transport. My understanding is that creating favourable conditions is different from a definitive causal-impact link, which implies consistent relationships and long-term impacts.
Minor comments:
Line 36: Regarding ‘unusually persistent warming’ in Extended Data Fig. 2b: If the linear trend is not removed, is the warming more significant? Are there temporal variations during this period? Student’s t test may be affected by false discovery rates, so it is suggested to use the Benjamini–Hochberg procedure alongside the test (Benjamini and Hochberg, 1995). The location of the warming resembles the South Pacific Convergence Zone (SPCZ). Could it be more directly linked to convective activities there?
Line 37: ‘A blocking high’: I only see Z300 anomalies rather than any high pressure system in the manuscript. During the whole period, is there a persistent high pressure system, or transient ones? I assume Z300 means geopotential height at 300 hPa, please define it. Why is 300 rather than 500 hPa used? I assume moisture is more abundant at 500 hPa, and thus moisture transport.
Line 41: The authors attempt to define a new tropical mode with influence distinct from global warming and other tropical modes. However, the signature positive SST anomalies are located closer to SPCZ rather than TWP, and differences are generally not significant. Based on the Methods section ‘Identification of TWP warming events’, it is hard to confirm the independence of TWP warming events from La Nina conditions.
Line 59: Is it a positive or negative trend of 3±15 Gt/yr?
Line 71: Was the anomalous precipitation primarily associated with Atmospheric River (AR)? How many AR events occurred, and would it worth to show a case study? Does global warming contribute to the intensity of the extreme precipitation associated with AR?
Line 72: Since Wang et al. (2025b) have linked this event to the three-year La Nina, is the mechanism reported in this manuscript against or complementary to their findings?
Line 131: ‘470.3 Gt’: uncertainty estimates should be provided, and it should be compared with other potential data sources and the following analysis (e.g. Fig. 3c and Extended Data Fig. 12b).
Line 141: A smaller positive anomaly is also evident in these datasets around 2009 in Fig 1b. Is it also associated with MCA3 and TWP warming?
Line 154: Fig. 1a, dashed red line showing MAR SMB is hardly distinguishable from the solid red line showing RACMO SMB. Why is there missing data in Ice mass from GRACE/GRACE-FO? Fig. 1b, QW mass rapidly declines after the peak in April 2023, why is that? Fig. 1e, does the Amundsen Sea Low have a role in maintaining and strengthening the high pressure ridge?
Line 165: Standard Deviation assumes normal distribution, but precipitation normally follows log-normal or gamma distribution? Maybe another approach such as a threshold as used in the literature makes more sense?
Line 172: The underlying assumption for this whole paragraph is that global warming resulted from anthropogenic forcings manifest in a uniform magnitude, which can be captured by CESM1. I am not fully convinced, as it has been widely understood large regional variability exists in response to global warming. I am wondering how much of this precipitation anomaly could be attributed to the fact that a warmer atmosphere could hold more moisture based on the Clausius–Clapeyron relation, i.e. a thermodynamic response, and how much can be decomposed to the dynamic factor. Could the authors attribute this dynamic factor to natural variability or (direct or indirect) anthropogenic forcings, or both (as it was said to be a key question this manuscript is investigating)?
Line 220: I am curious about the relationship between MCA3 and other common modes such as the Southern Annular Mode and the Zonal Wave-3 index (Goyal et al., 2022). Fig. 2e, why do QW mass and Cumulative standardized MCA3 precipitation diverge before ~2009? Do I miss a time series of MCA3 or why does the positive Standardized MCA3 Z300 before 2020 in Fig. 2c not result in large precipitation anomalies in Fig. 2d?

Line 232: Again, the warming in Extended Data Fig. 5c is more significant and pronounced over SPCZ rather than TWP. If the authors argue it is the TWP warming that affects MCA3, why is MCA3 also linked to cooling over the Indian Ocean and eastern Pacific? Could you conduct a partial correlation analysis between MCA3 and SST, while controlling IOD, La Nina, etc.?
Line 237: 'a recurrent physically robust teleconnection mode': MCA3 is dependent on precipitation. To be robust, I would expect more rigorous analysis between SST over TWP/SPCZ and circulation dipole near EA, without dependence on Antarctic precipitation and taking into account the impact of other tropical and SH modes.
Line 239: Extended Data Fig. 6c, the Standardized TWP SST is even more positive around 2000. If the hypothesis of the authors is correct, should it induce even stronger precipitation anomalies?
Line 252: 'eddy-mean flow feedback': It is far beyond my expertise, but I consider it extremely interesting about potential positive feedbacks that maintain the dipole circulation patterns. Without referring to the references, it is very hard to follow the arguments of the authors about the slow down of westerly jet and its consequences. Is it a hypothesis? Do you have more evidences in addition to the correlation patterns between Synoptic Eddy and MCA3 Z300 in Extended Data Fig. 7? As I asked before, are there temporal variations in TWP warming and the Rossby wave train during this event?
Line 264: The authors argue TWP warming recurs every ~10 years, whereas the high-latitude dipole circulation is more like an internal model of circulation patterns. Does the dipole circulation or MCA3 positive phase also recurs every 10 years following TWP warming?
Line 271: If a sensitivity experiment is conducted with SST anomalies imposed over SPCZ (more align with observed anomalies and correlation patterns), would you also observe the dipole circulation anomalies? The authors argue 'TWP warming is essential to producing the dipole circulation pattern over EA'. I am not yet fully convinced. It can produce does not mean it is the only way or a necessary requirement to produce.
Line 278: what does 'Gill-type' mean? In Fig. 3b, I am curious why the SST is increased more in the centre of the ellipse?
Line 296: The sensitivity experiment does show that the La Nina-related cooling over the eastern Pacific alone cannot explain the dipole circulation, but it cannot differentiate TWP warming from a La Nina-related warming over the tropical western Pacific.
Line 303: 'lower latitudes' compared to what?
Line 316: It seems to align with the findings of Gao et al., (2024) that Antarctic extreme precipitation is sourced from lower latitudes with smaller zonal transport compared to normal precipitation. Though they only publish data until 2022 (Gao et al., 2025), would it be worth to briefly check the overlapping period? Is it also worth using or noting the latest developments in process-oriented water tracing that could identify moisture source locations and environmental conditions associated with water phase change in iCAM6 (Fiorella et al., 2021), ECHAM6 (Gao et al., 2024), and UM (McLaren et al., 2025)?
Line 323: I am not really sure whether the Ferrel and polar cells are just patterns in the climatologically averaged fluxes or are really responsible for moisture and energy transport. Could the authors find a reference that documents their role in moisture and energy transport? I assume the authors mean 'the ascending branch' rather than 'the upper branch'? What are the relationships between 'storm-track activity and ARs' and is their role in moisture and energy transport quantified anywhere?
Line 327: If the evaporation over the moisture source regions does not increase, it could be expected that precipitation along the transport pathways decreases, as can be seen in Fig. 3c. This may affect ocean surface salinity and thus deep water formation (if any). Could the authors briefly comment on this impact?
Line 333: Again, the cooling in Extended Data Fig. 11a is more significant over SPCZ.
Line 369: Fig. 5a, what does x-axis mean? What is the average duration of these events?
Line 381: Extended Data Fig. 12a, the warming pattern is quite different from those in Fig. 2b and 5c. Although precipitation is lacking before 1979, ice core measurements from (Medley and Thomas, 2018) may still be more reliable than ERA-20C, which I assume is simply a model product without observational constraints before 1979? The precipitation anomalies in Extended Data Fig. 12b are small and insignificant over QW. Could you compare the difference with the 2021-23 event?
Line 391: Extended Data Fig. 12c&d, The circulation anomalies are displaced from EA and the precipitation anomalies seem small.
Line 447: 'poleward-shifted storm tracks': The author argued that 'This transport is further facilitated by storm-track activity and ARs' (Line 324), and argued here that a poleward shift of storm track is insufficient for the explanation of this event (Line 473). The poleward-shift is an average pattern and constitutes the behaviours of many storms. Since the storms or ARs here shift to the EA, it is more poleward than other SH storms or ARs. Could the author really argue it is unrelated and rule out the potential role of all other mechanisms proposed to explain the poleward shift of storm tracks?
Line 464: I do not see any discussions about sea ice. Could the record-low sea ice extent in 2022-23 contribute to the organization of the low/high pressure systems?

Benjamini, Y. and Hochberg, Y.: Controlling the False Discovery Rate: A Practical and Powerful Approach to Multiple Testing, Journal of the Royal Statistical Society: Series B (Methodological), 57, 289–300, https://doi.org/10.1111/J.2517-6161.1995.TB02031.X, 1995.
Fiorella, R. P., Siler, N., Nusbaumer, J., and Noone, D. C.: Enhancing Understanding of the Hydrological Cycle via Pairing of Process-Oriented and Isotope Ratio Tracers, J. Adv. Model. Earth Syst., 13, e2021MS002648, https://doi.org/10.1029/2021MS002648, 2021.
Gao, Q., Sime, L. C., McLaren, A. J., Bracegirdle, T. J., Capron, E., Rhodes, R. H., Steen-Larsen, H. C., Shi, X., and Werner, M.: Evaporative controls on Antarctic precipitation: an ECHAM6 model study using innovative water tracer diagnostics, Cryosphere, 18, 683–703, https://doi.org/10.5194/TC-18-683-2024, 2024.
Gao, Q., Sime, L. C., McLaren, A. J., and Werner, M.: Datasets for the article "Moisture source controls on water isotopes in Antarctic precipitation - insights from water tracers in ECHAM6-wiso" [Dataset], Zenodo, https://doi.org/10.5281/zenodo.15107251, 2025.
Goyal, R., Jucker, M., Gupta, A. Sen, England, M. H., Goyal, R., Jucker, M., Gupta, A. Sen, and England, M. H.: A New Zonal Wave-3 Index for the Southern Hemisphere, J. Clim., 35, 5137–5149, https://doi.org/10.1175/JCLI-D-21-0927.1, 2022.

Li, X., Cai, W., Meehl, G. A., Chen, D., Yuan, X., Raphael, M., Holland, D. M., Ding, Q., Fogt, R. L., Markle, B. R., Wang, G., Bromwich, D. H., Turner, J., Xie, S. P., Steig, E. J., Gille, S. T., Xiao, C., Wu, B., Lazzara, M. A., Chen, X., Stammerjohn, S., Holland, P. R., Holland, M. M., Cheng, X., Price, S. F., Wang, Z., Bitz, C. M., Shi, J., Gerber, E. P., Liang, X., Goosse, H., Yoo, C., Ding, M., Geng, L., Xin, M., Li, C., Dou, T., Liu, C., Sun, W., Wang, X., and Song, C.: Tropical teleconnection impacts on Antarctic climate changes, Nature Reviews Earth & Environment 2021 2:10, 2, 680–698, https://doi.org/10.1038/s43017-021-00204-5, 2021.
McLaren, A. J., Sime, L. C., Wilson, S., Ridley, J., Gao, Q., Gorguner, M., Line, G., Werner, M., and Valdes, P.: Implementation of water tracers in the Met Office Unified Model, Geosci. Model Dev., 18, 8129–8142, https://doi.org/10.5194/gmd-18-8129-2025, 2025.
Medley, B. and Thomas, E. R.: Increased snowfall over the Antarctic Ice Sheet mitigated twentieth-century sea-level rise, Nat. Clim. Chang., 9, 34–39, https://doi.org/10.1038/s41558-018-0356-x, 2018.
Palerme, C., Claud, C., Dufour, A., Genthon, C., Wood, N. B., and L'Ecuyer, T.: Evaluation of Antarctic snowfall in global meteorological reanalyses, Atmos. Res., 190, 104–112, https://doi.org/10.1016/J.ATMOSRES.2017.02.015, 2017.
Wang, D., Ma, H., Li, X., Hu, Y., Hu, Z., An, C., Ding, M., Li, C., Jiang, S., Li, Y., Lu, S., Sun, B., Zeng, G., van den Broeke, M., and Shi, G.: Sustained decrease in inland East Antarctic surface mass balance between 2005 and 2020, Nature Geoscience 2025 18:6, 18, 462–470, https://doi.org/10.1038/s41561-025-01699-z, 2025a.
Wang, S., Liu, J., Cai, W., Yang, D., Kerzenmacher, T., Ding, S., and Cheng, X.: Strong impact of the rare three-year La Niña event on Antarctic surface climate changes in 2021–2023, npj Climate and Atmospheric Science 2025 8:1, 8, 173-, https://doi.org/10.1038/s41612-025-01066-0, 2025b.

Version 1:

Reviewer comments:

Referee #1

(Remarks to the Author)
General comments

The authors have addressed most of my comments in a satisfactory manner. The manuscript does appear more robust with a cleaner narrative in this latest version. I feel this manuscript represents an important advancement in understanding tropical-Antarctic teleconnections and will serve as a good example in terms of methodology for future studies when making similar tropical-polar teleconnection studies. Overall, I support the publication of this manuscript after the following specific comment and minor comments are addressed.

Specific comments

1. There is one comment from my previous review that I would like to see further investigated. Regarding the March 2022 extreme East Antarctic AR, it was obvious that the event was accompanied by an increase in AR activity and precipitation in the Queen Mary Land and Wilkes Land. I was hoping instead that you could look at the tropical SST conditions and Rossby wave activity during March 2022 and see if there are any similarities with the TWP SST patterns identified in your analysis. I thought it could be nice for the reader to see a concrete example of a precipitation event during the 2021-2023 that fits the narrative of the TWP/QW precipitation teleconnection narrative. Of course, if word limits are a problem, then do not worry about trying to include this example as it is not a crucial component of the study.

Minor comments

Fig. 1e: The AR anomaly contours are a nice addition, but I find it difficult to follow the contours. In the current form, it is hard to tell if the red contours imply that there were positive AR anomalies around most of coastal Antarctica or just in the interior although I assume the answer is coastal Antarctica. I guess the red and blue contours naturally end at the coastline as you are only showing AR anomalies over land? Can you also color the coastline where relevant so that the contours represent enclosed regions?

Line 72: “Rising height trend of the tropopause”. Just to make it clearer for readers not familiar with the tropopause.

Line 225: The phrasing makes it sound like the Rossby wave train is a continuous feature. Suggest rephrasing it to “which increases the likelihood of a triggering a Rossby wave train that propagates towards the high southern latitudes and …”

Line 282: I recommend adding a quick sentence or a few works pointing the reader towards the “Extended Text for Extended Data Fig. 8: Sensitivity Experiments 2–7” just so the reader is aware that a greater discussion on the sensitivity experiments is available.

Referee #2

(Remarks to the Author)
I co-reviewed this manuscript with one of the reviewers who provided the listed reports.

Referee #3

(Remarks to the Author)
The authors have presented a major revision to their paper. I am generally content with their revision and remain enthusiastic about their idea and the manuscript.

The one part of the revision I am still not entirely content with is related to the clear growth in the Antarctic Peninsula. This does not negate the current analysis but it clearly doesn;t allow the perception to be given that WL is the only relevant area as it still is in L192 or L339. The argument that other modes are secondary modulators L342 requires that MCA1 and 2 are just a redistribution of mass with zero net effect. So I think a further refinement of language is required.

L116 the law Dome ice core shown in Extended Figure 1b does not go past 2010. So there is a major error in "precipitation anomaly during July 2021–April 2023, which is evident in all three precipitation datasets and is also observed in the snow-accumulation record from the Law Dome ice core (Fig. 1f–h and Extended Data Fig. 1b)." If teh data somehow does go beyond 2010 but is not visibile in the plot then a new data reference is required as Thomas et al do not include data up to date.

Minor comments:

Abstract: 'moistening of Antarctica' is a strange phrase and does not reflect what is happening. Antarctica is not becoming more moist! Maybe just 'Therefore, the recent Antarctic Ice Sheet mass gain is likely temporary and does not yet reflect a sustained, global warming-driven surface-mass shift.'

L46 the uncertainty is over some timeframe. multidecadal or centennial or something "long term uncertainty". the uncertainty is not largest this decade.

L50: "In contrast, the current mass balance of the East Antarctic Ice Sheet (EAIS), which contains 50 nearly 80% of Earth's land ice, *is more sensitive to large* precipitation-driven variability".

L52 it is not clear why ice shelf melt is introduced here and what the link to snowfall variability is. If the desire is to link to ice dynamic changes, then I think invoking change in basal melt is required. I don't see the value of this sentence though and think the snowfall variability part can be merged with teh previous sentence

L52 diverge *from zero and* beyond their uncertainties

L98 " halted between July 2021 and April 2023" - htese are very precise dates. approximately? Is the end confidently locked to mid 2023 or did it continue longer?

L101 delete "has"

L262 check for circulation figure ref is correct as not on Fig 1

Referee #4

(Remarks to the Author)
Thank you, I really appreciate your efforts in addressing my questions. I can see most questions are addressed in satisfactory ways. However, my major concern regarding the link between TWP and La Nina, as also pointed out by other reviewers, remains. I consider it sloppy to characterize La Nina as just cooling over eastern tropical Pacific, though the index is normally defined based only on SST in this region. My detailed comments are listed below.
The link between TWP and La Nina was suggested by a few reviewers for better clarification, and it is addressed by the authors through sensitivity experiments with La-Nina-like cooling over eastern tropical Pacific. I consider it an oversimplification to describe La Nina as just cooling in this region, though it is defined based on SST in this region (e.g. Nino 3.4). La Nina is a basin-wide phenomenon, characterised by a stronger Walker Circulation in the atmospheric part,

warmer-than-usual SST in its ascending branch, and colder-than-usual SST in its descending branch (See Wikipedia 2020–2023 La Niña event, https://en.wikipedia.org/wiki/2020%E2%80%932023_La_Ni%C3%B1a_event). A focus on the cooling part while ignoring the warming part close to TWP is not satisfactory.
My null hypothesis is that TWP warming proposed by the authors is indeed part of the triple La Nina. I believe the author would agree that the real world will never exhibits TWP warming as in the ECHAM5 simulations, and a slight shift (north-south or east-west by a few degrees) or reshape (more circular or rectangle) of the TWP warming could still excites Rossby wave train and favours the dipole pattern near Antarctica. This simple fact raises concern that the impact of TWP warming may be hardly distinguishable from the impact of the warming associated with La Nina. And I speculate they are basically the same thing named differently when their spatial extent is slightly different. To test this hypothesis, I would suggest another set of ECHAM5 sensitivity experiments, where SST anomalies associated with La Nina (this triple event or a composite from multi-decades SST data) over the whole tropical regions (not just cooling over eastern tropical Pacific) and over the globe (just to test the role of extratropical regions, even if negligible) are superimposed on the climatological SST. If these experiments also show dipole pattern near Antarctica and precipitation anomalies over QW, then it suggests the teleconnection patterns may be better discussed within the framework of ENSO-Antarctica teleconnections. In this case, I would wonder how much scientific value this paper adds for our understanding of the global impacts of ENSO, and how innovative it is to detect a different teleconnection pathway that is just slightly shifted eastward or westward.
I would expect some discussions on the mechanisms regarding how TWP warming, or La Nina warming, is linked to intensified or more concentrated convection over the ascending branch of the Walker Circulation, and how these convection activities are linked to Rossby wave train. If the findings can be framed within the context of the impact of strengthened Walker Circulation on Antarctic climate, it may be helpful to incorporate the role of IOD.
It could be expected that La Niña-like cooling or IOD-like cooling are not conducive to convection and thus Rossby wave excitation. It is interesting that they help to produce a response slightly stronger than that in the TWP-only experiment. This seems to suggest that more concentrated convective activities in the ascending branch of the Walker circulation, which are favoured by warming over TWP and cooling over eastern tropical Pacific and tropical Indian Ocean, are more effective at excites Rossby wave train.
Resp Fig. 4.6a, Resp Fig. 4.7d, Resp Fig. 4.9b, and Resp Fig. 4.23a resemble what may be expected from La Nina events in tropical Pacific. Resp Fig. 4.22. and Resp Fig. 4.23c suggests that while controlling ENSO and IOD influences, partial correlation between the MCA3 Z300 index and TWP SST is insignificant.

Open Access This Peer Review File is licensed under a Creative Commons Attribution 4.0 International License, which permits use, sharing, adaptation, distribution and reproduction in any medium or format, as long as you give appropriate credit to the original author(s) and the source, provide a link to the Creative Commons license, and indicate if changes were made.
In cases where reviewers are anonymous, credit should be given to 'Anonymous Referee' and the source.
The images or other third party material in this Peer Review File are included in the article's Creative Commons license, unless indicated otherwise in a credit line to the material. If material is not included in the article's Creative Commons license and your intended use is not permitted by statutory regulation or exceeds the permitted use, you will need to obtain permission directly from the copyright holder.
To view a copy of this license, visit https://creativecommons.org/licenses/by/4.0/

We thank all reviewers for their careful evaluation and constructive comments. We have substantially revised the manuscript in response to these comments. To facilitate the review process, we include figures, clarifications, and explanations for each comment, rather than only referring reviewers to responses to similar concerns raised by other reviewers. As a result, the response document is 68 pages long. Response figures are numbered as Resp Fig. X.Y, where X indicates the reviewer number and Y indicates the sequence of figures for that reviewer. The main changes are summarized below:

1. We revised the Abstract, Introduction, and Discussion to clarify the framing and rationale of the study. We more carefully state that one intuitive interpretation of the 2021–2023 East Antarctic mass gain is that it is "consistent" with the expected long-term precipitation response to global warming, rather than being "seen" as such a signal. This is a fair statement, as a number of studies, including IPCC AR6 chapters, stress that Antarctic precipitation is expected to increase under global warming.

2. We strengthened the discussion of the role of atmospheric rivers (ARs) and extreme precipitation in contributing to the 2021–2023 slowdown in Antarctic mass loss over the Queen Mary Land and Wilkes Land (QW) region. We added daily AR–precipitation analyses over QW, including an illustration of the March 2022 extreme AR event, and clarified that the TWP teleconnection provides a large-scale circulation background that favors AR-related moisture transport and snowfall extremes toward QW.

3. We clarified the relationship between the TWP–East Antarctica teleconnection and other climate modes. We added analyses and discussion comparing our mode with ENSO/La Niña, IOD, SAM, IPO, and zonal wave-3. We also added a new SPCZ-only AGCM experiment, which shows that SPCZ forcing alone does not reproduce the observed East Antarctic (EA) dipole, whereas TWP-only forcing does.

4. We added evidence from multiple additional datasets and simulations to test the robustness of the proposed mechanism, including RACMO2.4 precipitation, ice-core accumulation records, and two long-term paleo-reanalysis datasets, EKF400v2 and LMR v2.1. These results support the link between QW precipitation and the EA dipole over longer time periods.

5. We revised the discussion of the future relationship between TWP events and global warming. We removed the CESM1 future event-frequency analysis and now treat future changes in the TWP–EA teleconnection as an open question. We clarify that the future precipitation impact of this teleconnection may depend on changes in high-latitude moisture, AR intensity, storm tracks, and other coupled climate processes, which are beyond the scope of the present study.

6. We moved the analysis of the occurrence frequency of TWP events and the schematic diagram summarizing our mechanism to the Extended Data, which helps tighten the reasoning and improve the flow of the presentation. If the reviewers and editor feel that this material would help readers better understand the results, and if space allows, we can consider moving it back to the main text in a future revision.

7. We improved the figures, captions, methods, and wording throughout the manuscript, including revised colorbars, clearer event definitions, a clearer explanation of Z300, the use of false discovery rate (FDR) correction for significance testing, and other minor corrections.

Below, we provide detailed point-by-point responses to the reviewers' comments. Reviewer comments are shown in black, and our responses are shown in blue.

Referees' comments:
Referee #1 (Remarks to the Author):

General comments

This study attributes the cause of the recent pause in Antarctic mass loss to a persistent tropical warm pool (TWP) located across the central to southern Pacific Ocean. Through a robust and extensive analysis, the authors argue that the existence of this TWP triggers a poleward propagating Rossby wave train that leads to a blocking high over East Antarctica which allows for the frequent poleward advection of warm, moist airmasses sourced for the mid-latitude Indian Ocean. The linking of similar TWP events in recent decades with East Antarctic snowfall trends along with the projected occurrence of similar TWP in future climate simulations marks this feature as something that is independent of the general warming and moistening trend that is projected to increase Antarctic snowfall.

The argument for this framework of the TWP controlling East Antarctic snowfall trends is compelling due to the in-depth analysis of the teleconnection. These methods include multiple reanalysis and observational datasets, maximum covariance analysis (MCA) for identifying distinct circulation modes, AMIP model experiments involving multiple atmospheric configurations with two different atmospheric general circulation models, atmospheric river detection, CESM1 Large Ensemble for assessing precipitation patterns during historical and future periods, and moisture source tracing using iCESM. Overall, the wide variety of methods employed by the authors are impressive and are well respected in the field of large-scale atmospheric dynamics.

The manuscript succeeds in convincing the reader that TWP and thus internal variabilty is driving snowfall trends across parts of East Antarctica. However, my main two issues are the narrative framing of the research problem and missing links with existing literature related to this topic. For instance, the abstract states that the recent increase in ice mass across East Antarctica, “has been seen as an early indicator of an expected long-term response to global warming, in which storm tracks shift poleward and deliver more precipitation to Antarctica”. Then the results from this study are presented as a counter to this prevailing narrative. This seems misleading as there is no existing literature that claimed this recent pause in Antarctic mass loss is the result of a long-term sustained global warming signal causing more precipitation. Of course, it is very interesting to see the authors show that the recent mass gains in East Antarctica are driven by internal variability as opposed to a forced response, but the framing of the results as something counter to a prevailing narrative seems unnecessary.

This leads to my second issue as most existing literature frames this mass gain in East Antarctica as an extreme weather topic, but extreme weather is largely not addressed in this manuscript besides correlations in atmospheric river (AR) activity with the MCA3 Z300 index. As the recently published Kolbe et al. (2026) shows in Figure 2a, the mean precipitation across the Antarctic ice sheet from 2020-2024 is marginally higher than the 2003-2019 mean, but extreme precipitation events largely drove the surface mass balance trends. This notion is backed up in other literature like Turner et al. (2019). One suggestion could be to examine the TWP conditions during the notorious extreme East Antarctica AR event in March 2022 to see how it compares with the teleconnection framework identified in this manuscript. Regardless, I feel this manuscript needs to bridge the gap between teleconnections and extreme weather events for it to be considered holistic.

Overall, I feel this manuscript is well written and the analysis is robust and timely. On the scientific merits, I would support publication after the following concerns are addressed.

We thank you for the time and effort spent evaluating our manuscript. We are encouraged by your positive assessment of our work and appreciate the constructive comments. These suggestions are very helpful for improving the clarity and quality of the manuscript.

We agree with both of your main points and have revised the manuscript accordingly. A consensus has not yet been established that the 2021–2023 East Antarctic mass gain reflects a long-term global warming signal, as this slowdown event is quite recent and many studies on this topic may still be underway. However, it is widely accepted in the community that Antarctic precipitation is expected to increase in a warmer climate (Nicola et al., 2023; Frieler et al., 2015; IPCC AR6). To be more accurate, we revised the Abstract to emphasize that the recent slowdown is "consistent" with the expected long-term precipitation response to global warming, rather than being "seen" as such a signal. This is a fair statement, as a number of studies, including IPCC AR6 chapters, stress that Antarctic precipitation is expected to increase under warming, although total Antarctic mass will likely continue to decline. Nicola et al. (2023) also stated in the first sentence of their abstract that "With progressing global warming, snowfall in Antarctica is expected to increase, which could counteract or even temporarily overcompensate increased ice-sheet mass losses caused by increased ice discharge and melting." Thus, in the polar community, an intuitive explanation for why East Antarctic mass loss slowed during 2021–2023 is that global warming may be driving this change. Please see the revised manuscript at Abstract [L31–33].

Regarding your second point about the role of ARs in our analysis, our interpretation is not that the TWP teleconnection provides an alternative explanation to AR-driven extremes, but rather that it provides the large-scale circulation control that favors them. In the revised text, we state that the TWP-related dipole circulation enhances poleward moisture transport and increases AR activity toward East Antarctica, thereby helping organize the extreme snowfall events that drive much of the SMB anomaly. This finding is consistent with previous studies emphasizing the importance of extreme precipitation events and ARs for Antarctic snowfall variability. To strengthen this point, we further examined the relationship between daily precipitation and AR activity over QW during the 2021–2023 event. We added a new analysis showing that pointwise daily precipitation and AR-frequency anomalies, after removing the seasonal cycle, are positively correlated along much of the Antarctic coastal margin during 1979–2024, with correlations exceeding 0.4 near East Antarctica (Resp Fig. 1.1; based on more than 16,000 daily samples). Over the QW sector from July 2021 to April 2023, both precipitation and AR anomalies are anomalously high (Resp Fig. 1.2), and daily QW-averaged AR-frequency and precipitation anomalies are strongly correlated ($r = 0.69$, $p < 0.01$; black solid line in Resp Fig. 1.3). Even after excluding the two days with the most extreme AR anomalies (red dots), the correlation remains high ($r = 0.67$). We also overlaid AR-frequency anomalies as contours in Fig. 1e. The enhanced precipitation over QW clearly coincides with increased AR activity during the event. In addition, we examined the well-known March 2022 extreme East Antarctic AR event and found that it was accompanied by a sharp increase in both AR activity and precipitation anomalies over QW (Resp Fig. 1.4). These new results support our interpretation that the TWP-driven teleconnection helped establish a favorable large-scale circulation background for extreme AR-related snowfall over East Antarctica during the event period. Please see Fig. 1e and Extended Data Fig. 5e,f, as well as L226–229 and 304–307 in the revised manuscript.

Nicola, L., Notz, D. & Winkelmann, R. Revisiting temperature sensitivity: how does Antarctic precipitation change with temperature? Cryosphere 17, 2563-2583 (2023).

Frieler, K. et al. Consistent evidence of increasing Antarctic accumulation with warming. Nat. Clim. Change 5, 348-352 (2015).

IPCC. Regional fact sheet – Polar Regions. In Climate Change 2021: The Physical Science Basis. Contribution of Working Group I to the Sixth Assessment Report of the Intergovernmental Panel on Climate Change (2021).

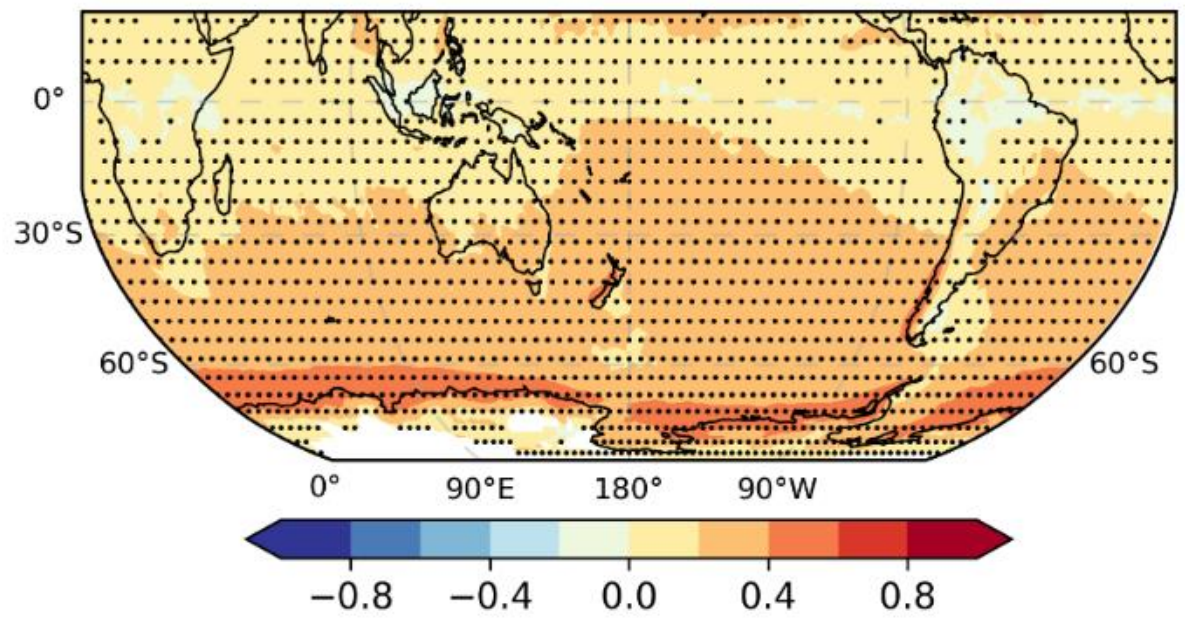

Resp Fig. 1.1. Correlation between daily precipitation anomalies and AR-frequency anomalies during 1979–2024. Daily precipitation is from ERA5, and AR frequency is based on the Guan and Waliser AR dataset. Both time series were deseasonalized and detrended before the analysis. Stippling indicates regions significant at the 95% level based on an FDR-corrected Student's t test.

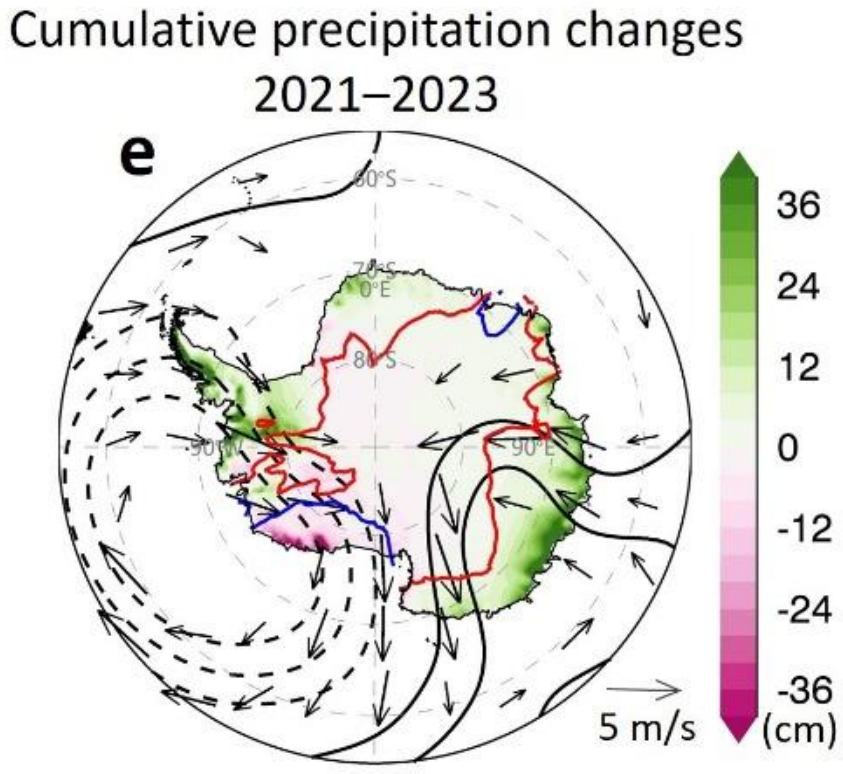

Resp Fig. 1.2 (also Fig. 1e in the main text). Cumulative Antarctic precipitation changes during July 2021–April 2023 (April 2023 minus July 2021), together with AR and circulation anomalies during the event. Shading shows cumulative ERA5 precipitation anomalies. Red and blue contours indicate positive and negative AR-frequency anomalies during the event, respectively, at ±0.001 count/day. The unit count/day (ranging from 0 to 1) represents the daily AR frequency. Black contours and vectors represent mean 300 hPa geopotential height (Z300) and wind anomalies for the 10 months during the event when the area-weighted average QW precipitation exceeded the 80th percentile of the raw monthly distribution for 1979–2024.

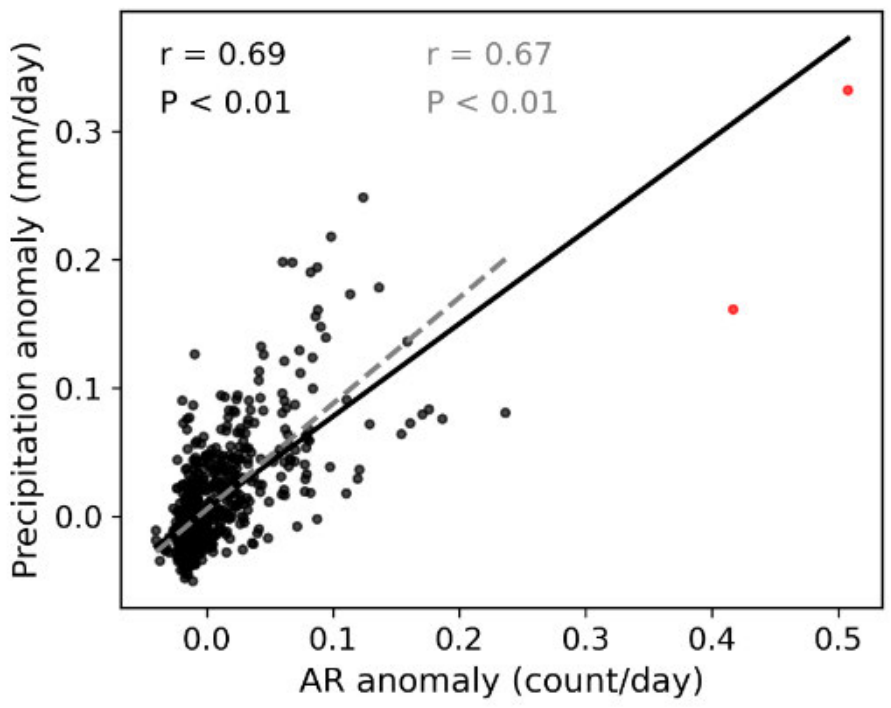

Resp Fig. 1.3. Scatterplot of daily precipitation anomalies versus AR-frequency anomalies, both averaged over the QW sector (70–145°E, 65–75°S), from July 2021 to April 2023. The solid black line shows the linear fit using all data points (r = 0.69). The dashed line shows the fit excluding the two days (red dots) with the most extreme ARs (r = 0.67).

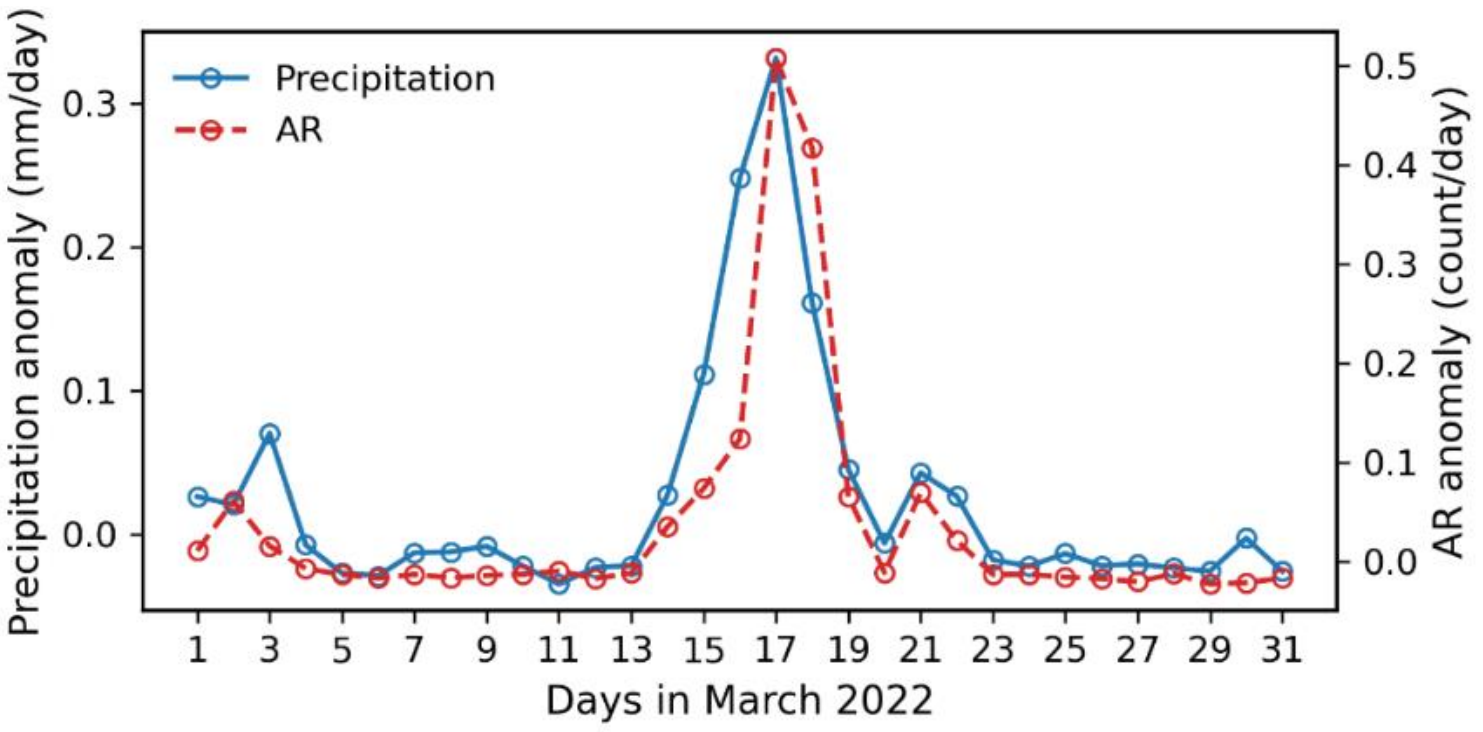

Resp Fig. 1.4. Daily time series of precipitation anomalies and AR-frequency anomalies, both averaged over the QW sector (70–145°E, 65–75°S), during March 2022. The figure shows that the March 2022 extreme East Antarctic AR event was accompanied by a sharp increase in both AR activity and precipitation over QW.

Specific comments

1. Looking at Figure 2, the MCA precipitation index has had other spikes in the past like most recently around 2010. In this case, the mass balance in the Queen Mary Land and Wilkes Land sector (QW) area also increased, but the overall Antarctic Ice Sheet mass balance remained largely negative. How can you explain this difference in Antarctica mass balance? Was there something different about the precipitation patterns across East Antarctica or was mass loss across West Antarctica higher?

Yes, a smaller positive excursion also occurred around 2010 in the QW sector. The QW mass series (black line in Resp Fig. 1.5b), the cumulative QW precipitation series (green line in Resp Fig. 1.5b), and the cumulative MCA3 precipitation series (green line in Resp Fig. 1.6) show a coherent increase around 2010. However, this event was weaker and shorter-lived than the 2021–2023 event. Therefore, the QW mass gain around 2010 only partly slowed the Antarctic-wide mass loss. This small effect is still visible as a temporary slowdown signal in the Antarctic-wide mass series (black line in Resp Fig. 1.5a).

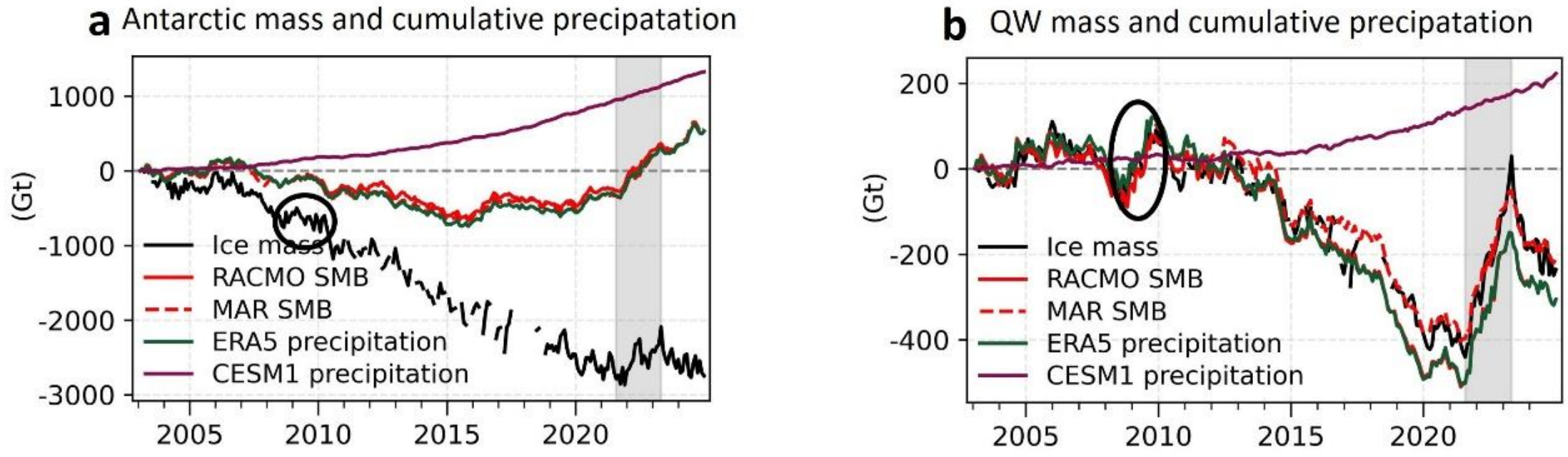

*Resp Fig. 1.5 (also Fig. 1a-b in the main text). Antarctic mass change and associated precipitation anomalies in the past two decades. **a**, Time series of deseasonalized Antarctic ice mass changes (black) from GRACE/GRACE-FO, compared with cumulative anomalies of SMB (precipitation minus meltwater runoff, sublimation, evaporation, and wind erosion) from RACMO2.4 (solid red line) and MAR3.14 (dashed red line), precipitation from ERA5 (green), and ensemble-mean precipitation from the 40-member CESM1 Large Ensemble (magenta). All series are relative to January 2003. The grey shading marks the mass reversal period (July 2021–April 2023). **b**, As in **a**, but for the QW sector (70–145°E, 65–75°S).*

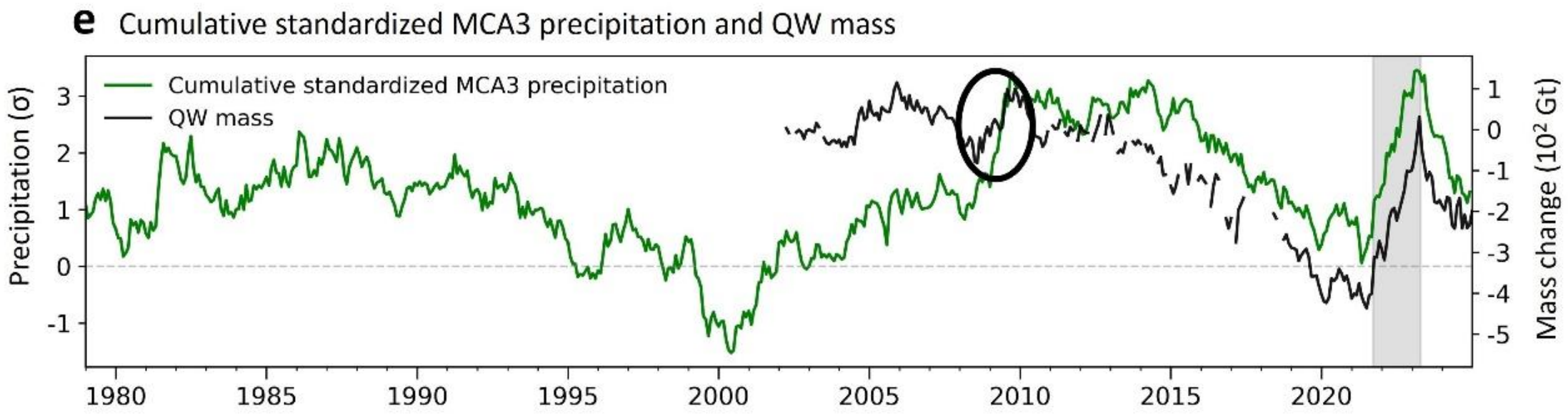

Resp Fig. 1.6 (also Fig. 2e in the main text). Comparison between the cumulative standardized MCA3 precipitation time series (green) and the QW mass change time series (black).

2. As seen in Fig. 1d, while the SMB changes in the QW area were the most positive, it also appears increased snowfall across Queen Maud Land and around the Antarctica Peninsula also helped to increase the Antarctic mass balance. Can the TWP also explain the increased snowfall in these regions or could this be related to another mechanism like the longer-term increased moisture signal from global warming or the sea-ice minima identified in (Kolbe et al. 2026).

We thank you for this comment. Our results suggest that the TWP teleconnection mainly explains the enhanced snowfall over the QW sector, but not necessarily snowfall increases in other Antarctic regions. Specifically, the MCA3 precipitation pattern shown in Resp Fig. 1.7 does not exhibit significant positive anomalies over Queen Maud Land or the Antarctic Peninsula. In addition, the ECHAM5 and CAM5 experiments forced by TWP warming do not produce significant positive precipitation responses in either region (Resp Fig. 1.8 and Resp Fig. 1.9). We also examined the spatial correlation pattern between the QW precipitation index and precipitation anomalies south of 30°S, and found that precipitation variability in East Antarctica is not significantly correlated with that in West Antarctica (Resp Fig. 1.10), suggesting that precipitation changes in the QW sector and in these two regions may not be driven by the same TWP forcing. We note that precipitation changes over these two regions may be related to circulation-driven AR activity. The low-pressure anomaly over West Antarctica may favor more AR activity into the Antarctic Peninsula region and help explain the local precipitation anomalies (Resp Fig. 1.2). Thus, circulation-driven

AR activity may also help explain precipitation changes in other parts of Antarctica. Furthermore, we cannot rule out the possibility that global warming and sea-ice changes also contributed to precipitation changes in these regions. However, over QW, the CESM1 forced precipitation increase during July 2021–April 2023 is only 32.3 ± 2.4 Gt, about 9% of the observed ERA5 cumulative precipitation anomaly over the same region and period (351.2 ± 26.2 Gt). This suggests that the QW precipitation increase during this event is mainly associated with anomalous circulation rather than the forced response to global warming. In addition, Kolbe et al. (2026) found that sea-ice loss through increased evaporation accounts for only about 10% of the winter precipitation increase, and is small compared with the contribution from remote large-scale processes. We cited Kolbe et al. (2026) as a recent study linking Antarctic sea-ice loss to Antarctic ice-mass changes. Please see the revised manuscript at L156–158.

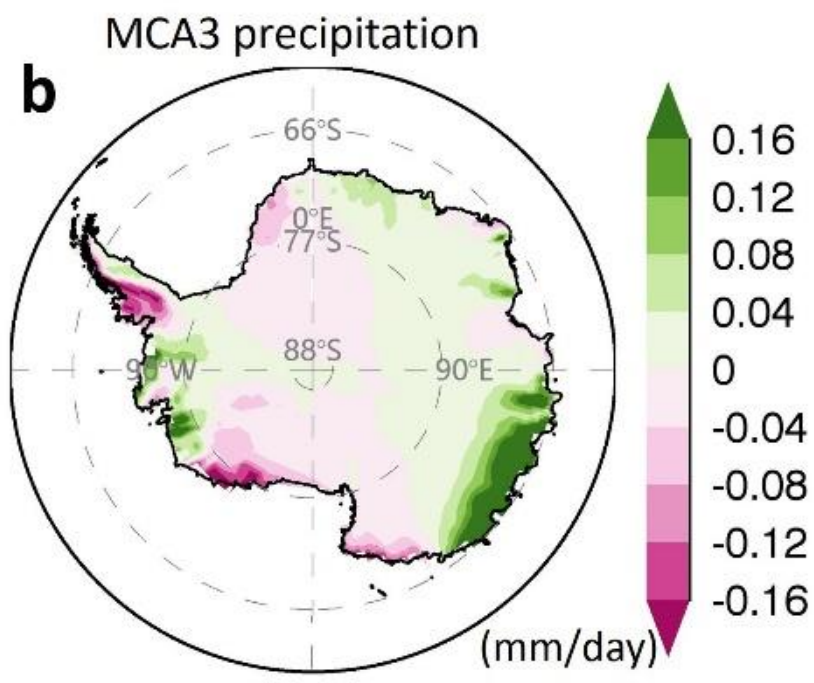

Resp Fig. 1.7 (also Fig. 2b in the main text). The third precipitation MCA mode linking Antarctic precipitation and SH Z300 for 1979–2024.

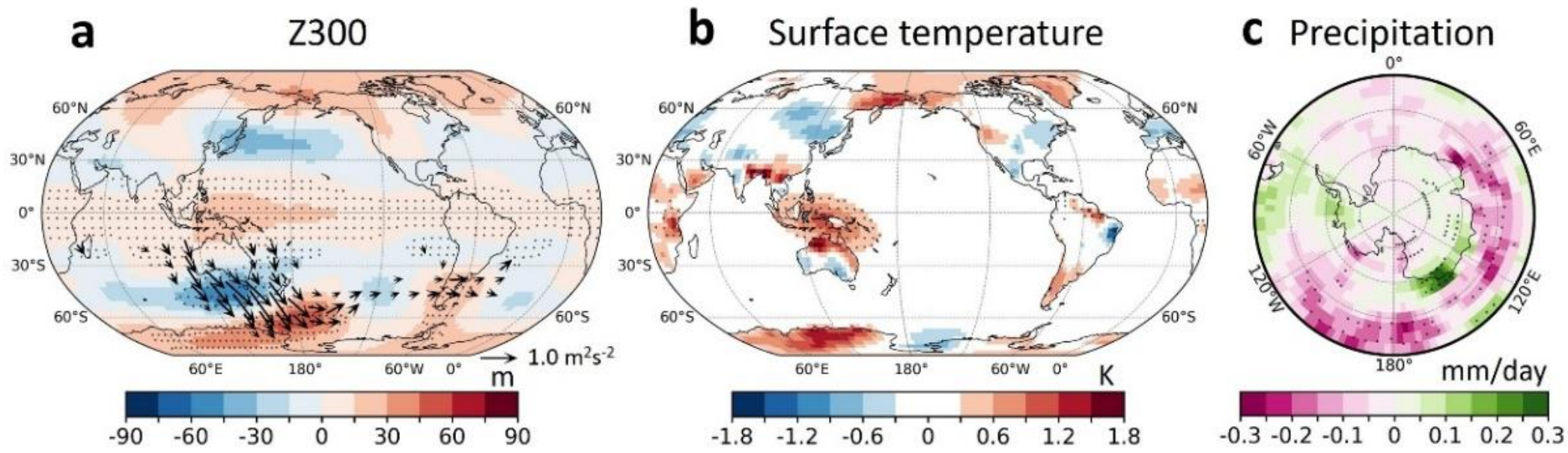

*Resp Fig. 1.8 (also Fig. 3 in the main text). Simulated EA-TWP teleconnection using ECHAM5. Simulated responses to positive TWP SST anomalies, shown as the difference between the 40-year mean of the sensitivity experiment and that of the control experiment. **a–c**, Responses of Z300 (**a**), surface temperature (**b**), and precipitation (**c**). Black vectors indicate WAF. Stippling indicates regions significant at the 95% level based on an FDR-corrected Student's t test. The TWP is defined as a 15° clockwise-rotated ellipse centered at 135°E, 5°S, with semi-major and semi-minor axes of 35° and 15°, respectively. Positive SST anomalies within the ellipse increase from 0.5 K at the edge to 1 K at the center.*

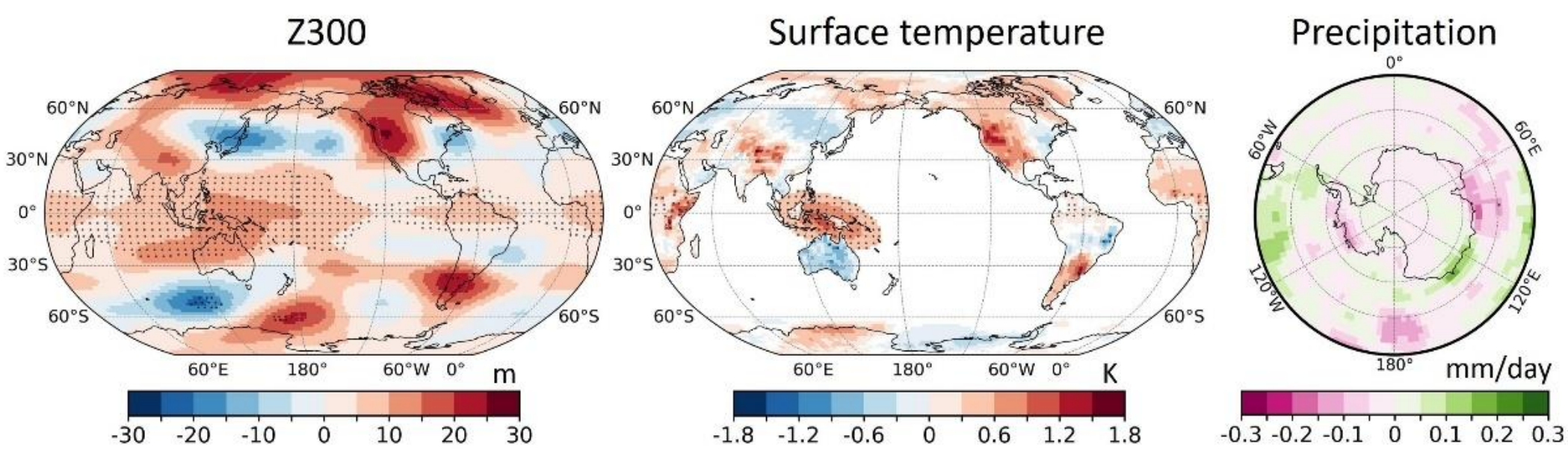

Resp Fig. 1.9 (also Extended Data Fig. 9). As in Fig. 3 in the main text, but simulated by the CAM5.

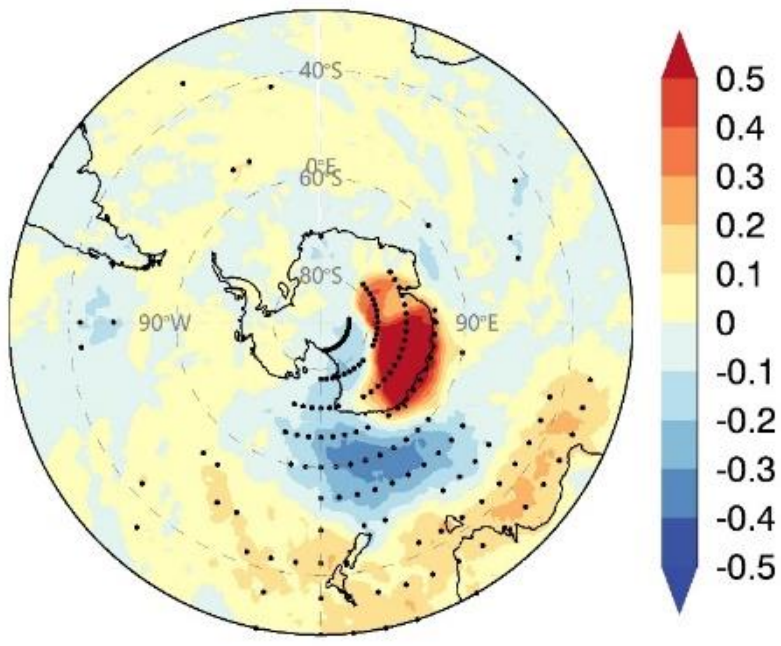

Resp Fig. 1.10. Correlation pattern between the QW precipitation index and precipitation anomalies south of 30°S during 1979–2024. The QW precipitation index is defined as the area-mean precipitation anomaly over the QW sector (70–145°E, 65–75°S). Both the index and precipitation anomalies were deseasonalized and detrended before the correlation analysis. Stippling indicates regions significant at the 95% level.

3. Disentangling the TWP teleconnection: In the Discussion section, I would like to see how the proposed teleconnection is related to other teleconnections like the recent La Nina event as outlined in Wang et al. (2025). Also Marshall et al. (2017), highlighted how the PSA2 pattern (related to ENSO) can drive an enhanced moisture flux towards Wilkes Land.

We agree that the proposed TWP teleconnection should be discussed more clearly in relation to other known Southern Hemisphere teleconnections. Wang et al. (2025) interpreted the 2021–2023 Antarctic anomalies mainly in the context of the rare triple-dip La Niña. Their EOF/regression analyses emphasize a La Niña-related Ferrel Cell shift and a PSA-like circulation pattern, including a deepened Amundsen Sea Low. This pattern is associated with broader Antarctic surface-climate anomalies, including sea-ice loss and increased precipitation/SMB over parts of East Antarctica and the Antarctic Peninsula. However, it does not show regional East Antarctic dipole similar to the one identified in our study, which consists of a low-pressure anomaly south of Australia and a high-pressure anomaly over the East Antarctic coast. Therefore, their La Niña-related large-scale circulation response differs from the TWP-related dipole mechanism emphasized here. Our idealized AGCM experiments show that TWP warming alone can reproduce this dipole circulation and the positive precipitation response over East Antarctica, whereas La Niña-like cooling alone does not reproduce a comparable dipole or positive precipitation anomaly.

Marshall et al. (2017) showed that PSA2 is associated with enhanced meridional moisture flux in the Eastern Hemisphere, including toward Wilkes Land. However, the PSA2 pattern is not identical to the dipole circulation identified here. Please see the revised manuscript at L351–366.

4. Previous work has identified similar mechanisms where tropical SST hotspots can drive a Rossby wave train towards Antarctica and trigger extreme weather conditions (see (Clem et al. 2022). It could be nice to see a short discussion on how your methods compare with these studies and whether this concept of tropical SST hotspots triggering poleward extreme heat and precipitation is something that can be generalized across Antarctica.

Our study is consistent with Clem et al. (2022) in the sense that regional tropical SST/convection anomalies can trigger a poleward Rossby wave response and Antarctic extremes. However, the two studies differ in both regions of interest and methods used to address the issue. Clem et al. (2022) focused on extreme high-temperature and surface-melt events over the Antarctic Peninsula/Larsen C, where central tropical Pacific

convection drives a South Pacific wave train, ARs, and foehn warming. In contrast, our study focuses on East Antarctica, especially the QW sector, and on a multi-year precipitation and mass-gain event rather than short-lived heat extremes. Methodologically, Clem et al. (2022) mainly identified the tropical link from observations and reanalysis, whereas our study additionally tests the forcing more directly using idealized AGCM sensitivity experiments. These experiments show that TWP-only warming can reproduce the East Antarctic dipole and positive precipitation response, while La Niña-like cooling alone cannot. We also use water-tagging experiments to trace the main moisture source regions of QW precipitation anomaly. Together, our study and Clem et al. (2022) may offer a useful reference for future studies of tropical–Antarctic teleconnections. We have cited this work [L87].

5. Looking at Figure 5, the difference in TWP warming event frequency between historical and future climate projections appear minimal. Are there other ways that global warming could be superimposed on future TWP events. Like it is possible for these events to cause more snowfall across East Antarctica in the future?

We agree that changes in the frequency of TWP events are not the only way global warming may affect future TWP-related precipitation over the EAIS. Global warming may also influence the precipitation impact of future TWP-driven events through several more complex pathways, many of which may be model dependent. In the revision, we removed the CESM1 future event-frequency analysis, because future changes in the TWP–East Antarctica teleconnection are likely controlled by several coupled processes, including changes in high-latitude atmospheric moisture, AR intensity, storm tracks, precipitation phase, and ocean–atmosphere feedbacks. These processes are complex and require a more comprehensive coupled-model analysis, which is beyond the scope of the present study. In the revised manuscript, we therefore treat this issue as an open question. Please see the revised manuscript at L374–376.

Minor comments

Line 30: “Sharp mass gain” should be more specific. The increase in surface mass balance offset the ice discharge.
Thank you. We have revised “sharp mass gain” to “sharp increase in surface mass balance” [L30].

Line 31-32: Is this really the case? I am not aware of any discourse that stated this recent increase in snowfall was part of a long-term response to global warming. Most literature about this mass loss pause has focused on extreme weather events.

Please see our response to your related similar comments General comment 1 for more details. A quick answer is that you are right: no specific papers argue that the 2021–2023 EAIS mass gain was caused by global warming. However, an accepted explanation in the community is that warming will favor increased Antarctic precipitation, which may partly offset ice-mass loss at some point in the future (Nicola et al., 2023; Frieler et al., 2015; IPCC AR6).

Line 66: Citation 30 deals with future atmospheric river changes. It would be more appropriate to cite Maclennan et al. (2022) to describe AR contribution to total precipitation.

Thank you for pointing this out. We have replaced it with Maclennan et al. (2022) [L61].

Line 73: “landfalls”

We have deleted this sentence in the revised manuscript.

Line 98-100: None of the studies cited (59-62) actually analyzed the link between teleconnections and ARs in East Antarctica. You could cite Shields et al. (2022), which found links between ARs and the Indian Ocean Dipole/ENSO.

Thank you for pointing this out. We have revised the sentence and added Shields et al. (2022). [L89]

Figure 1: I suggest reversing the colorbar for c and d so that blue is positive and red is negative

We have reversed the colorbars in Fig. 1–3.

Line 128-129: According to Extended Data Fig. 1, while mass loss remained slightly negative across the rest of Antarctica (excluding QW), SMB also increased and actually follows the CESM1 simulation. This would imply that the gradual increase of moisture from anthropogenic changes is having an impact there and thus should be impacting QW as well. Is it possible that the long-term global warming trends are having an impact on other parts of Antarctica?

You are correct that long-term global warming in the real world may contribute to increased atmospheric moisture and precipitation over QW during the event. As we responded to your early point, this thought is a very natural and intuitive answer to explain the EAIS mass gain event across the community. To estimate the impact of global warming, we rely on model simulations forced by anthropogenic forcing. Using the CESM1 Large Ensemble, we find that the cumulative precipitation anomaly over QW during July 2021 to April 2023 in observations was roughly 11 times larger than the precipitation increase induced by the long-term warming trend alone in the CESM1 Large Ensemble (Resp Fig. 1.5b). This suggests that the SMB increase over QW during 2021–2023 is still primarily attributable to anomalous circulation and its impact on steering AR activity and enhancing associated precipitation anomalies. Please see the revised manuscript at L156–158.

Line 141: “rarely observed”? please specify what this is in context to?

We revised “Such a strong positive anomaly is rarely observed” to “The recent mass gain over QW was the most pronounced in the 20-year GRACE observational record” in the revised manuscript [L118–119].

Figure 3c: I suggest reversing the colorbar as green is normally associated with excessive precipitation.

We have reversed the colorbars in Fig. 1–3.

Line 328-331: The increased moisture sourcing from the Indian ocean subtropics follows an analysis from Wille et al. (2024) which found enhanced latent heating in this region in the days preceding AR landfalls at Dumont d’Urville station.

We thank you for pointing this out and for referring us to the important study by Wille et al. (2024). We have cited this study in the revised manuscript [L301–302].

Line 726-727: In fact, most studies of ARs around Antarctica tend to avoid using the Guan and Waliser algorithm given its tendency to be very permissive about detecting ARs across the Southern Ocean, but not detecting ARs once they move beyond the Antarctica coastline (see Shields et al. 2022). Still, for this study, the Guan and Waliser algorithm is a reasonable choice over regional algorithms like Wille et al. (2021) given the need for analyzing AR activity across the Southern hemisphere (Extended Data Fig. 5). I suggest adding this caveat about the Guan and Waliser algorithm here.

We thank you for this helpful suggestion. We have added a sentence to highlight this caveat in the Methods: "We note that the Guan and Waliser detection algorithm tends to sufficiently capture ARs over the Southern Ocean, but may be less effective at capturing ARs after landfall (Wille et al., 2021; Shields et al., 2022). We adopt it here because our analysis requires a consistent AR detection method across the SH, rather than a regional algorithm." [L655–658]

Code availability: It would be preferable if all scripts used in this analysis were publicly available on Zenodo or Github

All scripts used in this analysis are publicly available on figshare: https://doi.org/10.6084/m9.figshare.31596730.[L768]

References:

Clem, K. R., D. Bozkurt, D. Kennett, J. C. King, and J. Turner, 2022: Central tropical Pacific convection drives extreme high temperatures and surface melt on the Larsen C Ice Shelf, Antarctic Peninsula. Nature Communications, 13, 3906, https://doi.org/10.1038/s41467-022-31119-4.

Kolbe, M., J. A. Torres Alavez, R. Mottram, M. Katurji, R. Bintanja, and E. C. van der Linden, 2026: Atmospheric rivers and winter sea ice drive recent reversal in Antarctic ice mass loss. Communications Earth & Environment, 7, 255, https://doi.org/10.1038/s43247-026-03242-3.

Maclennan, M. L., J. T. M. Lenaerts, C. Shields, and J. D. Wille, 2022: Contribution of Atmospheric Rivers to Antarctic Precipitation. Geophysical Research Letters, 49, e2022GL100585, https://doi.org/10.1029/2022GL100585.

Marshall, G. J., D. W. J. Thompson, and M. R. van den Broeke, 2017: The Signature of Southern Hemisphere Atmospheric Circulation Patterns in Antarctic Precipitation. Geophysical Research Letters, 44, 11,580-11,589, https://doi.org/10.1002/2017GL075998.

Shields, C. A., J. D. Wille, A. B. Marquardt Collow, M. Maclennan, and I. V. Gorodetskaya, 2022: Evaluating Uncertainty and Modes of Variability for Antarctic Atmospheric Rivers. Geophysical Research Letters, 49, e2022GL099577, https://doi.org/10.1029/2022GL099577.

Turner, J., and Coauthors, 2019: The Dominant Role of Extreme Precipitation Events in Antarctic Snowfall Variability. Geophysical Research Letters, https://doi.org/10.1029/2018GL081517.

Wang, S., J. Liu, W. Cai, D. Yang, T. Kerzenmacher, S. Ding, and X. Cheng, 2025: Strong impact of the rare three-year La Niña event on Antarctic surface climate changes in 2021–2023. npj Climate and Atmospheric Science, 8, 173, https://doi.org/10.1038/s41612-025-01066-0.

Wille, J. D., and Coauthors, 2021: Antarctic Atmospheric River Climatology and Precipitation Impacts. Journal of Geophysical Research: Atmospheres, 126, e2020JD033788, https://doi.org/10.1029/2020JD033788.

——, and Coauthors, 2024: Examining Atmospheric River Life Cycles in East Antarctica. Journal of Geophysical Research: Atmospheres, 129, e2023JD039970, https://doi.org/10.1029/2023JD039970.

Referee #2 (Remarks to the Author):
I co-reviewed this manuscript with one of the reviewers who provided the listed reports. In addition I provide the following comments.

Summary

The work presents an insight into understanding East Antarctica precipitation variability. The authors were able to show that the recent mass gain in Queen Mary Land and Wilkes Land can be linked to a teleconnection driven by positive SST anomalies in the tropical warm pool, which triggers a Rossby wave train and induces a high pressure anomaly over East Antarctica, rather than representing a direct global warming response. This provides a useful contribution to understanding East Antarctic mass change. In general, the manuscript is well structured and follows a clear logic, but it could benefit from several comments to further strengthen its clarity and impact.

General comments
A number of established climate modes—including the Southern Annular Mode (SAM), ENSO, the Indian Ocean Dipole (IOD), and the Interdecadal Pacific Oscillation (IPO)—are known to influence Antarctic mass variability through their impacts on cyclonic and anticyclonic circulation patterns. Several of these modes also correlate with TWP in this study. In this context, it would strengthen the manuscript to clarify how the influence of these modes was accounted for when isolating the TWP contribution. It would be helpful to understand whether the roles of SAM, ENSO, IOD, and IPO can be separated from the TWP signal and whether their relative contributions to the 2021–2023 mass gain event can be quantified. This question is particularly relevant because SAM and ENSO were largely in phase during 2021–2023 (positive SAM and La Niña), which could influence the high latitude circulation response. Clarifying how these overlapping modes were treated in the analysis would help reinforce the conclusion that the observed teleconnection is uniquely attributable to TWP SST anomalies rather than a combined response from multiple climate modes.

Thank you for reviewing our manuscript and for your positive assessment. We appreciate your suggestions, particularly regarding the need to better clarify the relationship between the TWP signal and other large-scale climate modes, including SAM, ENSO, IOD, and IPO. These comments helped us improve the clarity and strength of the manuscript, and we hope that our revisions have satisfactorily addressed your concerns.

We agree that the relationship between the TWP signal and other large-scale climate modes should be clarified more explicitly. Over the past four decades, the TWP SST index is significantly correlated with IOD-, ENSO-, and IPO-related variability, with correlation coefficients of 0.48, 0.41, and 0.25, respectively. Following your suggestion, we also calculated the correlation between the TWP SST index and the SAM index (Marshall et al. 2003), and found that it is very weak and not significant ($r = 0.02$). In the original submission, we addressed this issue using AGCM sensitivity experiments. The TWP-only experiment reproduces the dipole circulation and the positive precipitation response over East Antarctica (Resp Fig. 2.1). In contrast, the La Niña-like cooling-only and negative-IOD-like cooling-only experiments do not reproduce a significant East Antarctic dipole or a clear positive precipitation anomaly over this region (Resp Fig. 2.2b,c). In addition, the combined experiment with TWP warming, La Niña-like cooling, and IOD-like cooling gives a response slightly stronger than the TWP-only case (Resp Fig. 2.2a versus Resp Fig. 2.1). These results suggest that TWP warming is the more direct tropical forcing of the observed East Antarctic

dipole, while La Niña cooling and IOD cooling likely contribute to a tropical background cooling condition that may enhance the relative SST warming impact in the TWP.

In our study, the key circulation feature is a regional dipole, with a low-pressure center south of Australia and a high-pressure center over the East Antarctic coast. This pattern is associated with poleward moisture transport from the mid-latitude Indian Ocean into East Antarctica and enhanced AR activity along the western flank of the high-pressure anomaly. This is the main circulation feature linked to the QW precipitation anomaly in our results, and it is distinct from other modes that have been extensively studied in previous research on Southern Hemisphere teleconnections. Regarding the role of ENSO, we note that the typical ENSO pathway is different from the one emphasized here. ENSO usually affects Antarctica mainly through the PSA pathway, with the strongest influence often seen in West Antarctica and the Antarctic Peninsula, rather than in the East Antarctic sector (Yuan et al., 2018). The IOD pathway is also different. Previous studies suggest that IOD-related variability can affect eastern East Antarctica through circulation anomalies linked to the tropical Indian Ocean, but its spatial structure is not the same as the dipole pattern found in our results (Nuncio and Yuan, 2015). IPO-related impacts are broader, lower-frequency, and mainly tied to large-scale circulation variability over the Pacific basin, differing from the more localized TWP-origin forcing and regional dipole near the EAIS identified here (Li et al., 2021).

We would also like to elaborate on the relationship between SAM and our mode. SAM is usually described as a hemispheric annular mode associated with meridional shifts of the Southern Hemisphere westerly jet, rather than a localized tropical-forced dipole over the south-of-Australia/East Antarctica sector. On the other hand, we also agree that our mode may contain some SAM-like regional signal. This was first proposed by Ding et al. (2012), a previous study by a co-author of our manuscript, which showed that SAM has strong zonal asymmetry: in the Pacific sector, it resembles a tropically forced PSA-like wave train (Resp Fig. 2.3), while in the Indian Ocean sector, it includes a meridional dipole and is maintained by eddy–mean flow feedback. In that study, the dipole structure near East Antarctica was suggested to be internally driven due to strong eddy-mean flow feedback there. This is also consistent with our diagnosis in the study that synoptic eddy forcing helps maintain the dipole pattern once it is established. In our analysis, we show that this dipole structure can also be tropically-driven by TWP forcing, which is one of the main contributions and novel aspects of our study.

This interpretation is also consistent with Ayabilah et al. (2026), which is cited in the revised main text [L51]. They showed that AIS mass responses during ENSO-dominated periods are sensitive to ENSO diversity. They also showed that ENSO impacts on Antarctica can be modulated by the SAM and other processes, and that East Antarctic mass variability is closely related to the position of cyclonic and anticyclonic circulation anomalies. We have revised the manuscript so that it no longer implies that the event can be explained by TWP warming alone. We emphasize that prolonged TWP warming appears to be the more direct tropical forcing of the East Antarctic dipole, while other climate modes likely also influenced the large-scale circulation background in the tropics and potentially regulated the SST forcing in the TWP. Please see the revised manuscript at L351–366.

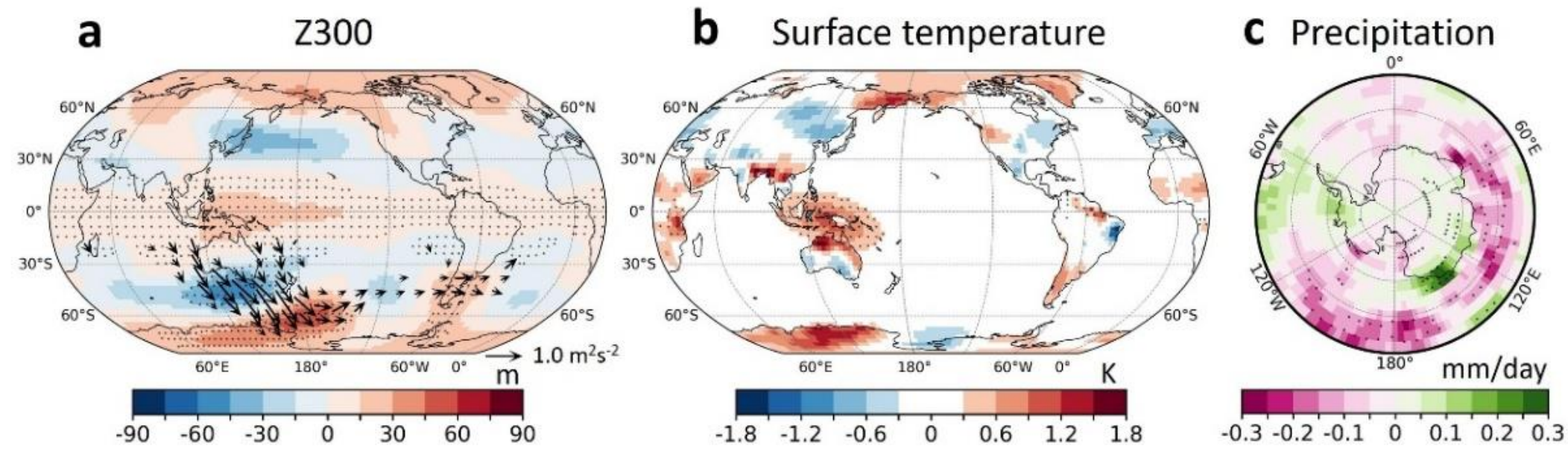

Resp Fig. 2.1 (also Fig. 3 in the main text). Simulated EA-TWP teleconnection using ECHAM5. Simulated responses to positive TWP SST anomalies, shown as the difference between the 40-year mean of the sensitivity experiment and that of the control experiment. ***a–c****, Responses of Z300 (****a****), surface temperature (****b****), and precipitation (****c****). Black vectors indicate WAF. Stippling indicates regions significant at the 95% level based on an FDR-corrected Student's t test. The TWP is defined as a 15° clockwise-rotated ellipse centered at 135°E, 5°S, with semi-major and semi-minor axes of 35° and 15°, respectively. Positive SST anomalies within the ellipse increase from 0.5 K at the edge to 1 K at the center.*

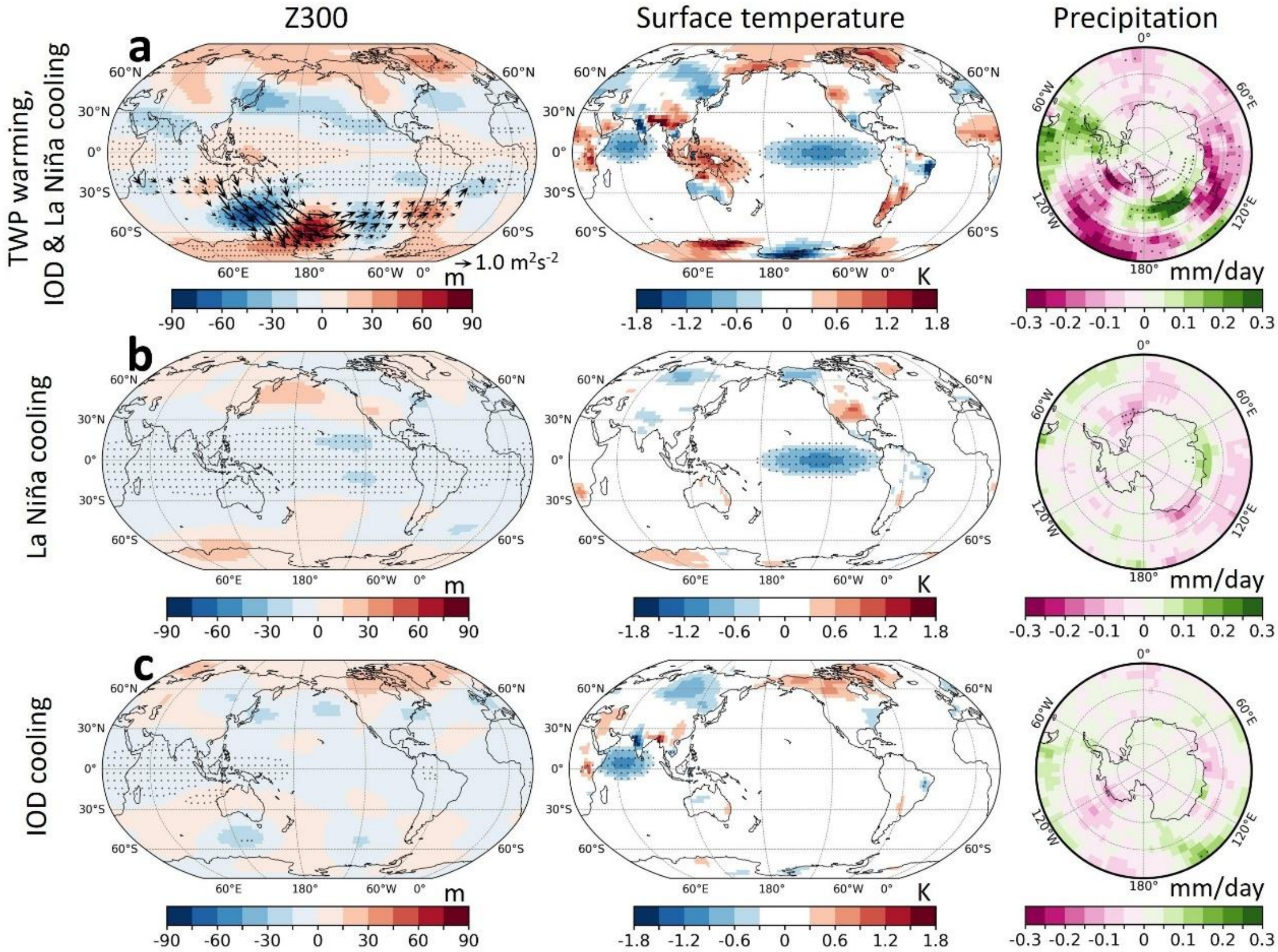

Resp Fig. 2.2 (also Extended Data Fig. 8a-c). ECHAM5 simulated atmospheric and surface responses to tropical forcing. ***a****, Responses of Z300 (left), surface temperature (middle), and precipitation (right) to combined forcing by TWP warming, IOD cooling, and La Niña-like cooling.* ***b****,* ***c****, As in* ***a****, but for isolated La Niña-like cooling (****b****) and isolated IOD cooling (****c****). Stippling indicates regions significant at the 95% level based on an FDR-corrected Student's t test. The TWP is defined as a 15° clockwise-rotated ellipse centered at 135°E, 5°S, with semi-major and semi-minor axes of 35° and 15°, respectively. SST anomalies in the ellipses generally follow a gradient from 0.5 K (or −0.5 K) at the edge to 1 K (or −1 K) at the center.*

[Redaction]
Figure Includes
Third Party Material

Resp Fig. 2.3. Schematic adapted from Ding et al. (2012) showing the roles of the subtropical jet and midlatitude jet in shaping the SAM during the nonsummer season, when the basic state is characterized by a double-jet structure. The red solid ovals denote anomalous high-pressure centers, and the blue dashed ovals denote anomalous low-pressure centers. Ding et al. (2012) argued that the SAM exhibits strong zonal asymmetry and tends to behave differently between the Eastern and Western Hemispheres. Internal dynamics, especially eddy–mean flow feedbacks, dominate variability in the Indian Ocean sector, whereas tropical SST-forced responses exert a stronger influence in the Pacific sector.

References

Ayabilah, J. B., King, M., Udy, D. & Vance, T. The changing mass of the Antarctic Ice Sheet during ENSO-dominated periods in the GRACE era (2002–2022). Cryosphere 20, 1237-1255 (2026).

Marshall, G. J. Trends in the Southern Annular Mode from observations and reanalyses. J. Clim. 16, 4134-4143 (2003).

Yuan, X., Kaplan, M. R. & Cane, M. A. The interconnected global climate system—A review of tropical–polar teleconnections. J. Clim. 31, 5765-5792 (2018).

Nuncio, M. & Yuan, X. The Influence of the Indian Ocean Dipole on Antarctic Sea Ice. J. Clim. 28, 2682-2690 (2015).

Li, X. et al. Tropical teleconnection impacts on Antarctic climate changes. Nat. Rev. Earth Environ. 2, 680-698 (2021).

Ding, Q., Steig, E. J., Battisti, D. S. & Wallace, J. M. Influence of the Tropics on the Southern Annular Mode. J. Clim. 25, 6330-6348 (2012).

Specific

L33: "we here show that"… Could brief describe how you did it

We revised this sentence to: " Using observations and model experiments, we here find that ". [L34]

L40: "historical period" suggests mentioning the period

Thank you for this suggestion. This statement is supported by both the observational record and CESM1 simulations, including the pre-industrial and historical runs, rather than by one particular period only. For this reason, instead of replacing it with one specific period, we revised the sentence to refer more clearly to observations and CESM1 simulations. We revised "Similar prolonged TWP SST warming recurs about once per decade during the historical period and maintains consistent recurrence in a warmer climate." to " Similar

prolonged warming in the tropical warm pool recurs about once per decade in observations and historical simulations." [L40–41]

L42: “cooling phase also explains a decade-long mass loss over the same region from 2011 to 2020”. Why not also mention the mass gain observed in the same region during 2000–2002, 2021-2023?

We would like to clarify that our manuscript is centered on the 2021–2023 mass gain event, because it is the most prominent ice mass gain event during the GRACE-observed period. The 2000–2002 event is discussed in the manuscript mainly as a similar but a weaker increase in QW precipitation and MCA3, and therefore was not included in the Abstract. In addition, this event occurred before the start of the GRACE record used in our analysis. We only emphasized the 2011–2020 mass-loss period in the Abstract because it reflects the opposite phase of the TWP–East Antarctic teleconnection. This helps show that TWP variability may be linked not only to East Antarctic mass gain during its warm phase, but also to East Antarctic mass loss during its cool phase. We have removed the text about 2011–2020 from the Abstract and moved the discussion of the 2000–2002 period to the Extended Text for Extended Data Fig. 12.

L42:43 “cooling phase also explains a decade-long mass loss over the same region from 2011 to 2020. Therefore, the recent Antarctic Ice Sheet (AIS) mass gain is likely temporary’”….. How likely is it that the warm phase of the TWP could produce a comparable decade long mass gain, similar to the mass loss effect attributed to the cooling phase?

In our results, the 2011–2020 ice-loss phase in QW was weaker but more persistent, averaging about −40 Gt/yr, whereas the 2021–2023 gain event was shorter but much stronger, averaging about +257 Gt/yr. This difference is clear in the monthly QW ice mass changes (Resp Fig. 2.4). We therefore think that whether a TWP phase can induce a decade-long signal in mass change relies on both the duration and amplitude of the phase.

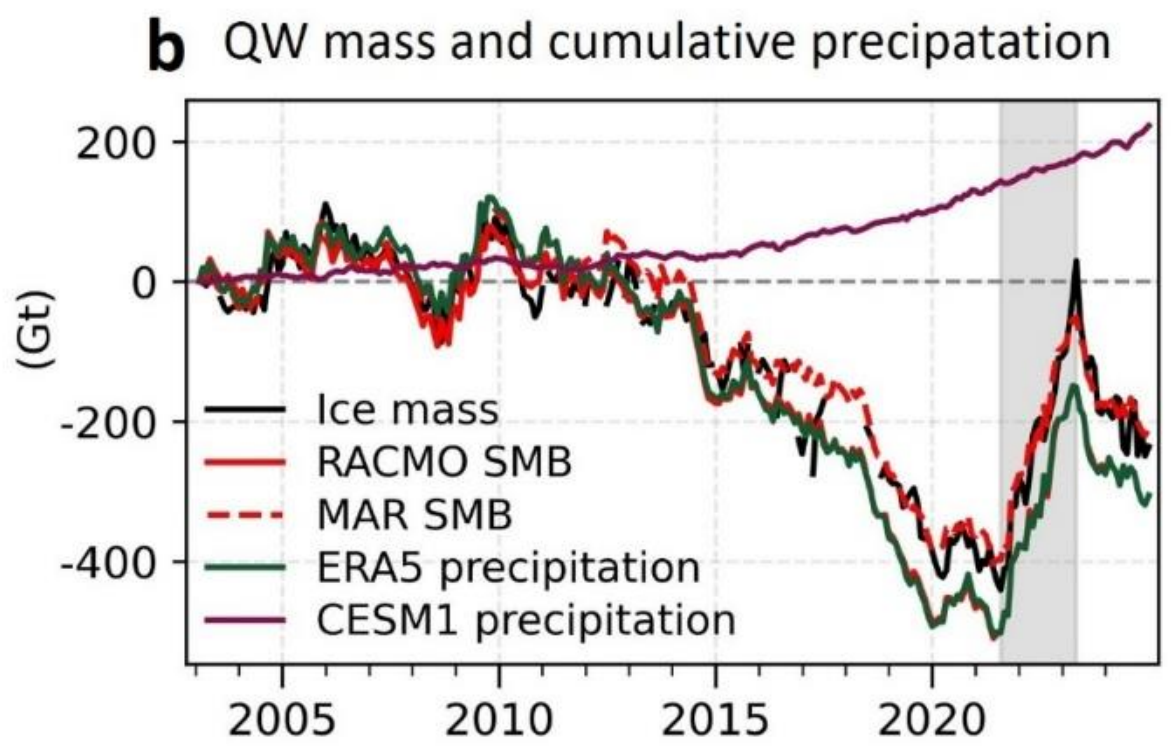

Resp Fig. 2.4(also Fig. 1b in the main text). Mass change and associated precipitation anomalies in the QW sector (70–145°E, 65–75°S). Time series of deseasonalized ice mass changes (black line) from GRACE/GRACE-FO, compared with cumulative anomalies of surface mass balance (SMB; precipitation minus meltwater runoff, sublimation, evaporation, and wind erosion) from RACMO2.4 (solid red line) and MAR3.14 (dashed red line), precipitation from ERA5 (green line), and ensemble-mean precipitation from the 40-member CESM1 Large Ensemble (magenta line). All series are relative to January 2003. The grey shading marks the mass reversal period (July 2021–April 2023).

L55-56: “In contrast, the East Antarctic Ice Sheet (EAIS), which contains nearly 80% of Earth's land ice, is

more sensitive to precipitation-driven surface mass balance (SMB) variability" … Please add an appropriate reference to support this statement.

We added four references to support this statement [L51]:

Ayabilah, J. B., King, M., Udy, D. & Vance, T. The changing mass of the Antarctic Ice Sheet during ENSO-dominated periods in the GRACE era (2002–2022). Cryosphere 20, 1237-1255 (2026).
Wang, D. et al. Sustained decrease in inland East Antarctic surface mass balance between 2005 and 2020. Nat. Geosci. 18, 462-470 (2025).
Kim, B.-H. et al. Partitioning the drivers of Antarctic glacier mass balance (2003–2020) using satellite observations and a regional climate model. Proc. Natl. Acad. Sci. USA. 121, e2322622121 (2024).
Medley, B. & Thomas, E. R. Increased snowfall over the Antarctic Ice Sheet mitigated twentieth-century sea-level rise. Nat. Clim. Change 9, 34-39 (2019).

L123-124: "between July 2021 and April 2023, during which the AIS gained approximately 695.0 Gt of compared to that in July 2021."… Is it possible to compare this change to the mass variations over the entire study period rather than only to July 2021?

We have calculated AIS mass changes for all running 22-month windows during 2003–2024 and compared them with the July 2021–April 2023 change. The AIS gained 695.0 Gt between July 2021 and April 2023. This ranks highest among the absolute values of all 22-month changes during the full 2003–2024 GRACE/GRACE-FO period. We have briefly mentioned this in the main text [L100].

L124-125: "As a result, the net Antarctic mass balance since 2021 has remained close to zero"… Is this conclusion derived directly from your results, or does it require a supporting reference?

This statement is derived directly from our GRACE results highlighted by the black circle in Resp Fig. 2.5, which show a net mass change close to zero after 2021.

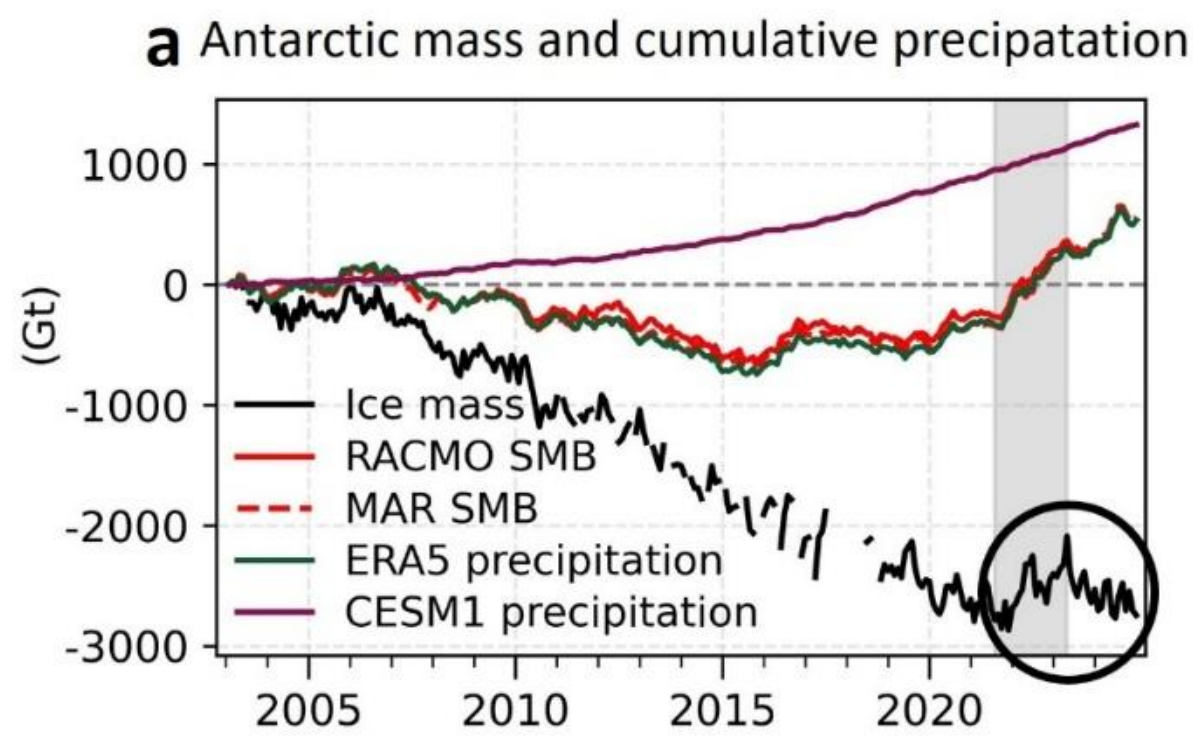

Resp Fig. 2.5 (also Fig. 1a in the main text). Antarctic mass change and associated precipitation anomalies in the past two decades. Time series of deseasonalized Antarctic ice mass changes (black) from GRACE/GRACE-FO, compared with cumulative anomalies of surface mass balance (SMB) from RACMO2.4 (solid red line) and MAR3.14 (dashed red line), precipitation from ERA5 (green), and ensemble-mean precipitation from the 40-member CESM1 Large Ensemble (magenta). All series are relative to January 2003. The grey shading marks the mass reversal period (July 2021–April 2023).

L126-129: "Spatially, this mass reversal is dominated by an extreme mass gain over EA (Fig. 1c,d), with the largest accumulation concentrated in the Queen Mary Land and Wilkes Land sector (QW; 70–145°E, 65–75°S). In contrast, the integrated mass changes in other Antarctic regions remain negative during the event (Extended Data Fig. 1).".… What about the mass gain observed in the Antarctic Peninsula? Should this also be discussed or clarified?

Our focus here is to identify the dominant contributor to the slowdown in Antarctic mass loss. In our results, this dominant contribution comes from East Antarctica, especially the QW sector, which accounts for approximately 68% of the continent-wide mass increase. We agree that positive mass anomalies also appear over the Antarctic Peninsula, accounting for about 22% of the total AIS mass gain during the event. However, negative anomalies of similar magnitude are present over the Amundsen Sea and Ross Sea sectors (Resp Fig. 2.6). These regional anomalies appear to be influenced by the negative phase of the ASL and largely offset each other in the integrated mass change over the West Antarctica–Antarctic Peninsula region. Following your comment, we revised the manuscript to clarify that the mass reversal is dominated by mass gain over East Antarctica and the Antarctic Peninsula, with the largest accumulation concentrated in the QW sector [L102–103].

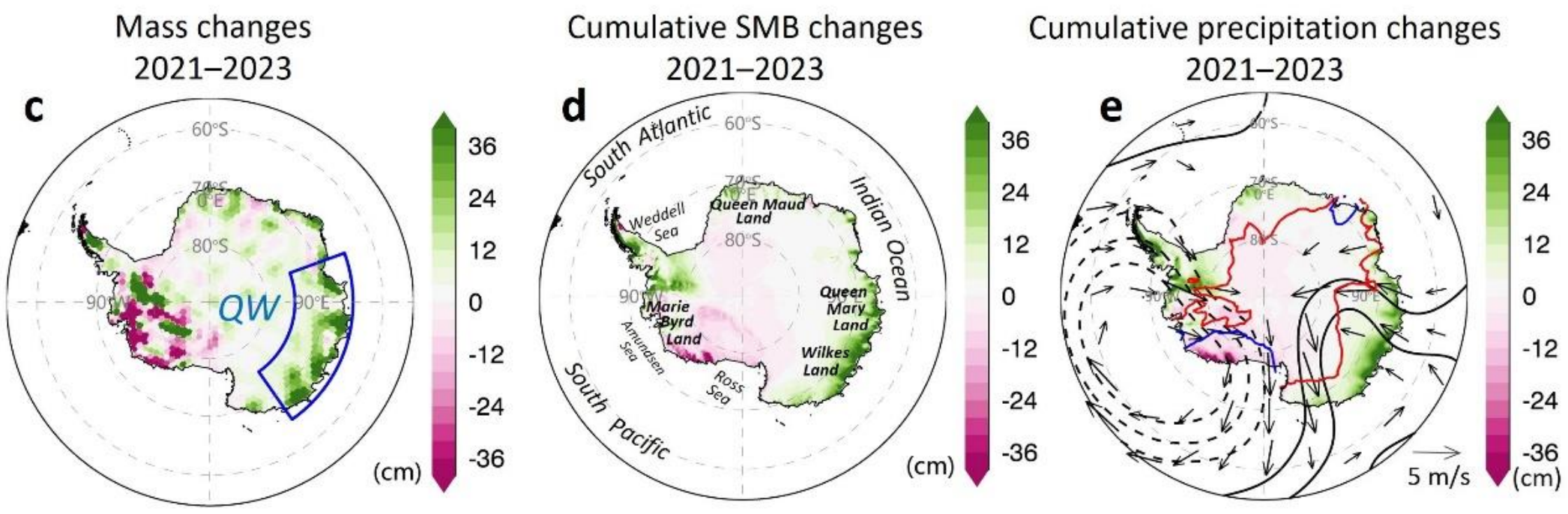

Resp Fig. 2.6 (also Fig. 1c-e in the main text). Spatial patterns of Antarctic mass change and associated climate anomalies. ***c****, Antarctic mass changes from GRACE/GRACE-FO during the event (April 2023 minus July 2021). The blue polygon defines the QW sector (70–145°E, 65–75°S).* ***d****, As in* ***c****, but for cumulative surface mass balance (SMB) changes from RACMO2.4.* ***e****, As in c, but for cumulative ERA5 precipitation changes. Red and blue contours indicate positive and negative AR anomalies (count/day) during the event, respectively. Black contours and vectors denote the mean Z300 and wind anomalies averaged over the 10 months when the area-weighted average QW precipitation exceeded the 80th percentile of the full-period raw monthly QW precipitation distribution.*

L143–145: "This 2021–2023 precipitation anomaly is accompanied by a quasi barotropic anticyclonic circulation anomaly near EA (Extended Data Fig. 2a), embedded within an Antarctic wide low pressure anomaly produced by the mega La Niña." This statement is somewhat ambiguous. Is East Antarctica experiencing a high pressure anomaly, a low pressure anomaly, or both simultaneously at different levels? Additionally, is the Antarctic wide low pressure anomaly attributed to the mega La Niña or SAM?

East Antarctica is characterized by a quasi-barotropic anticyclonic anomaly, while this regional high-pressure center is embedded within a broader low-pressure background over much of Antarctica south of 60°S (Resp Fig. 2.7). These two features are therefore not contradictory. They reflect a regional high-

pressure anomaly over East Antarctica superimposed on a broader Antarctic-scale low-pressure anomaly. We have revised the sentence to make this point clearer [please see L120].

The broader circulation anomaly is dominated by a deepened Amundsen Sea Low (ASL) and accompanying wave-like high-pressure anomalies across the Southern Hemisphere midlatitudes, forming a PSA-like pattern. This large-scale pattern is consistent with La Niña-related forcing during 2021–2023. At the same time, this pattern can also project onto a positive SAM-like structure, because SAM is not purely zonally symmetric and can contain strong zonal asymmetry. Ding et al. (2012) showed that the Pacific-sector component of SAM can resemble a tropically forced PSA-like wave train, while the Indian Ocean sector includes a meridional dipole structure that can be maintained by eddy–mean flow feedback. Therefore, we interpret SAM here mainly as part of the resulting high-latitude pressure pattern, rather than as the primary forcing of the anomaly. MCA1 between Z300 and precipitation across the entire Antarctic displays the La Niña-related PSA pattern, which shows a strong low-pressure anomaly over West Antarctica that also extends deep into East Antarctica. This low pressure covering much of the continent is what we refer to as the negative height anomaly in the background.

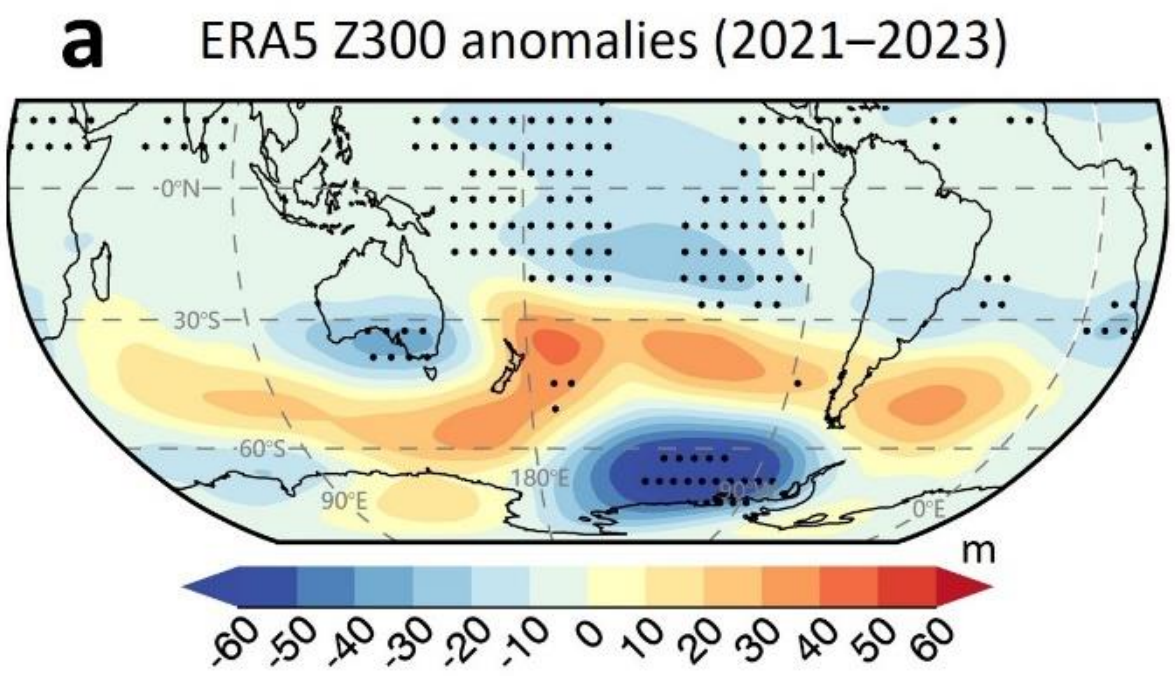

Resp Fig. 2.7 (also Extended Data Fig. 2a). Mean 300 hPa geopotential height (Z300) anomalies derived from ERA5 for the period of July 2021 to April 2023. Anomalies are computed after removing the climatological seasonal cycle and linear trend over 1979–2024. Stippling indicates regions significant at the 95% level based on an FDR-corrected Student's t test.

L145–147: Since the mass change is computed as April 2023 minus July 2021, is there any particular reason the contour pressure pattern was not computed to represent the mean circulation pattern during this period, but instead was done only for the 10 months of the event?

We did calculate the mean Z300 anomaly over the full event period (July 2021 to April 2023), and this pattern is shown in Extended Data Fig. 2a (Resp Fig. 2.7). In Fig. 1e (Resp Fig. 2.6e), we further overlaid the mean Z300 and wind anomalies for the 10 months when the area-weighted average QW precipitation exceeded the 80th percentile of the full-period raw monthly QW precipitation distribution. We used these 10 months because our purpose in Fig. 1e was to highlight the circulation pattern most directly associated with the strongest precipitation anomalies. The two circulation patterns are consistent, but the 10-month composite is stronger and more clearly shows the circulation associated with the extreme precipitation months. [L141–142].

We also note that persistent TWP forcing from 2021 to 2023 does not necessarily guarantee that the dipole structure will be equally persistent in every month. Eddy–mean flow feedbacks and strong internal atmospheric variability (such the SAM and the PSA related pattern) also regulate month-to-month changes

in high-latitude circulation. Thus, remote low-frequency SST forcing may increase the chance for the dipole structure to stay in a preferred phase, but the monthly circulation can still vary substantially. In addition, we emphasize that increased precipitation and AR activity over the EAIS are more sensitive to the emergence of high-pressure anomalies, which can steer more ARs into the region. In the opposite phase, when low-pressure anomalies develop, fewer AR landfalls occur, but precipitation can decrease only toward zero, rather than becoming negative in an absolute sense. This asymmetric response of precipitation and AR activity to circulation patterns makes high-pressure anomalies, even if they occur only during certain periods within the 22-month window, more effective in generating extreme precipitation and mass gain.

L153: In Fig. 1, panel "b QW mass cumulative precipatation" — please check the spelling of precipitation.

Thank you for pointing this out. We corrected this typo in Fig. 1.

L191: "SH Z300 anomalies for the …" Please provide a brief explanation of the rationale for using Z300.

We used Z300 because it is a standard upper-tropospheric level for diagnosing large-scale teleconnections and Rossby wave propagation. Circulation anomalies at this level usually have large amplitude and are less affected by Antarctic topography and the high elevation of the ice sheet than lower-level fields. Z300 also clearly captures teleconnection patterns linked to tropical forcing, because tropical forcing can excite Rossby wave responses that are strongest in the upper troposphere through divergent outflow and Rossby wave propagation. Another reason is that our results are not sensitive to the exact pressure level used. When we repeated the MCA analysis using Z500, we found a similar East Antarctic dipole mode. We have made this point clear in the main text [L172–173].

L193-195: "the leading two MCA modes capture circulation patterns associated with precipitation variability over WA and the Antarctic Peninsula (Extended Data 9 195 Fig. 3a–d)"…. I believe precipitation signals can also be observed in East Antarctica across all three MCA modes. However, the MCA3 pattern in QW appears closest to the observed mass change during 2021–2023. Perhaps this sentence could be rephrased to indicate that the MCA modes capture mass change patterns across Antarctica. Also, can each mode be associated with a particular large scale climate pattern?

We agree that some East Antarctic precipitation signals can also be seen in MCA1 and MCA2. However, our main point is that MCA3 is the mode that most clearly captures the coherent QW/East Antarctic precipitation variability relevant to the 2021–2023 event (Resp Fig. 2.8). In contrast, MCA1 and MCA2 do not show a similarly strong and coherent East Antarctic precipitation pattern. In addition, their East Antarctic positive and negative anomalies partly cancel each other, making them less effective in explaining the pronounced precipitation increase over the entire East Antarctic sector during 2021–2023. We also added an analysis showing the spatial correlation between the QW precipitation index and Antarctic precipitation anomalies (Resp Fig. 2.9). This pattern shows strong positive correlations over QW and nearby East Antarctica, indicating that QW precipitation mainly covaries well with regional precipitation over East Antarctica, but has a much weaker relationship with West Antarctic precipitation. We have revised "the leading two MCA modes capture circulation patterns associated with precipitation variability over WA and the Antarctic Peninsula" to "the leading two MCA modes capture circulation patterns associated mainly with precipitation variability over WA and the Antarctic Peninsula, while some spatially non-uniform signals are also present over EA (Extended Data Fig. 3a–d)" [L175–177].

Regarding the second point, MCA1 and MCA2 do show some similarity to known PSA-like patterns. However, the MCA1 Z300 pattern not only resembles a PSA1-like wave train, but also exhibits some features of the SAM and wavenumber-3 structure. MCA2 is also PSA-like, with some similarity to PSA2, but it is not identical because its main high-latitude circulation center is located near the Antarctic Peninsula rather than over the Ross Sea. Because PSA1 and PSA2 were originally defined based on specific datasets and periods, and because reanalysis over recent decades may change the detailed structure of each mode, assigning each MCA mode to a single circulation pattern may not be very comprehensive. In addition, the definitions of the SAM, PSA, and wavenumber-3 patterns were all based on EOF analysis, which ensures orthogonality among modes. Here, the focus is to assess which circulation pattern is most critical in shaping regional precipitation changes over the continent, rather than isolate the leading circulation modes in the SH. Given this, we prefer not to assign each MCA mode to one typical pattern in the manuscript. In this study, we focus on the newly identified MCA3, which is associated with the East Antarctic dipole and is most closely linked to the observed 2021–2023 event.

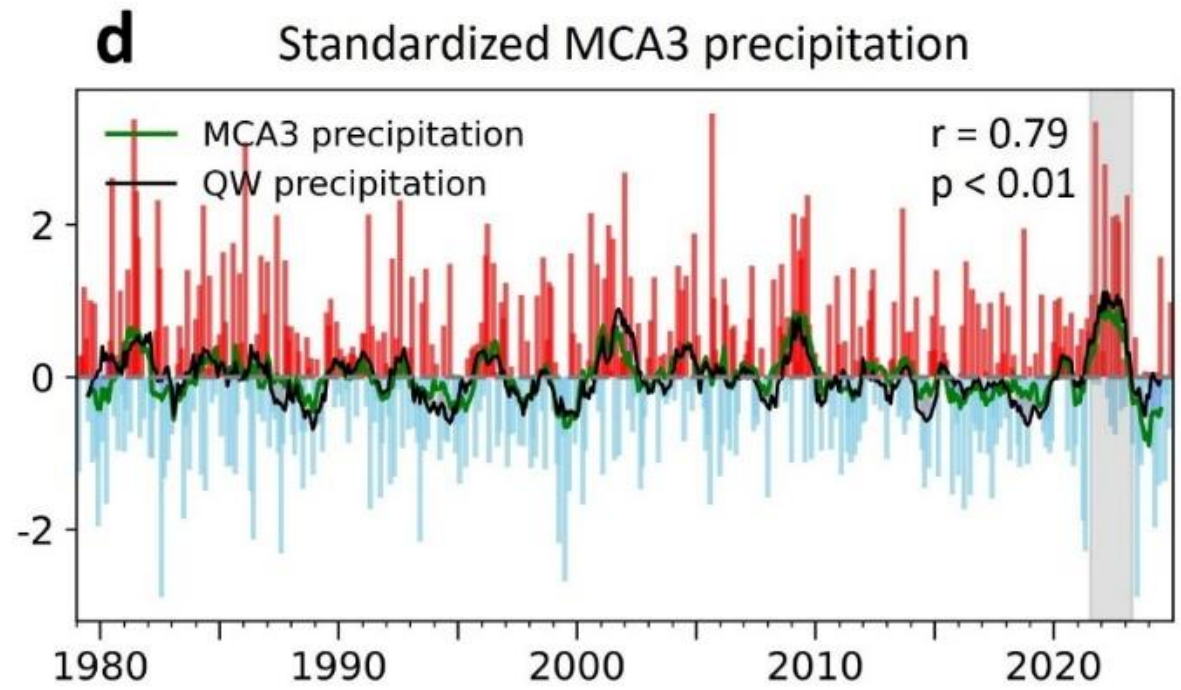

Resp Fig. 2.8 (also Fig. 2d in the main text). Standardized time series of the Antarctic precipitation for the third MCA mode. MCA linking Antarctic precipitation and Southern Hemisphere Z300 is applied to deseasonalized and detrended ERA5 monthly fields (south of 23°N for Z300) for 1979–2024. The green and black solid lines denote the 12-month running means of the standardized MCA3 precipitation time series and the standardized QW precipitation anomalies, respectively. The two time series exhibit a high correlation (r = 0.79, p < 0.05 based on effective degrees of freedom). Note that the MCA results are insensitive to whether the long-term trend is removed.

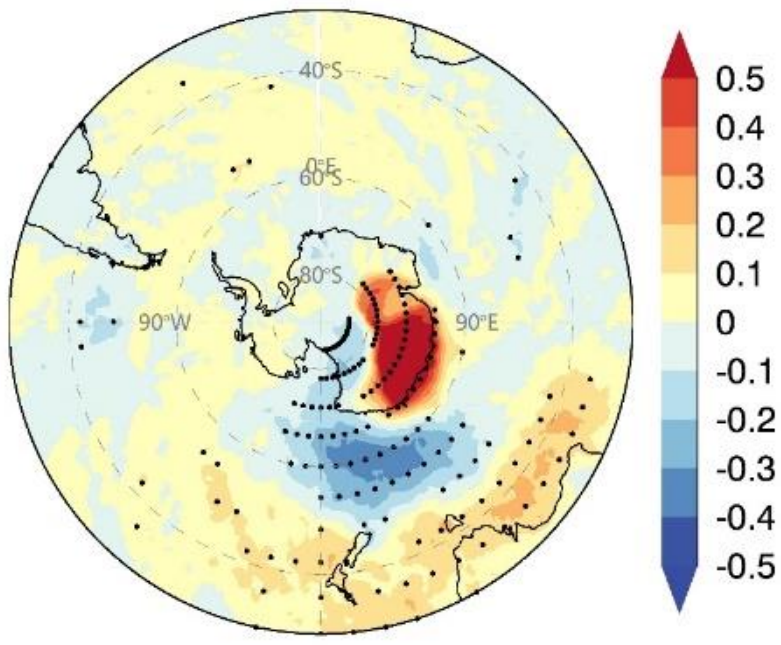

Resp Fig. 2.9. Correlation pattern between the QW precipitation index and precipitation anomalies across the Southern Hemisphere south of 30°S. The QW precipitation index is defined as the area-mean precipitation anomaly over the QW sector. Stippling indicates regions significant at the 95% level.

L215-216: "We find the time series of the cumulative MCA3 precipitation pattern closely aligns with the GRACE-derived mass balance gains in the QW (Fig. 2e)"….. How close is the alignment between the precipitation pattern and GRACE for the first MCA mode, particularly the mode linking Southern

Hemisphere Z300 and East Antarctic precipitation?

We checked this explicitly. The alignment between the GRACE-derived QW mass change and the cumulative precipitation time series of the first MCA mode linking Southern Hemisphere Z300 and East Antarctic precipitation (Resp Fig. 2.10) is also very strong, similar to that for MCA3 (Resp Fig. 2.11). This is expected, because that particular MCA mode is constructed specifically using East Antarctic precipitation and is therefore more directly linked to the East Antarctic SMB signal. Although the alignment is strong for the EA-focused MCA1, we still mainly focus on MCA3 in the main text because this mode is derived from the relationship between Southern Hemisphere circulation and Antarctic-wide precipitation. MCA3 clearly isolates the coherent QW/East Antarctic precipitation variability from the Antarctic-wide precipitation field, and therefore provides a more comprehensive view of the local precipitation–circulation coupling.

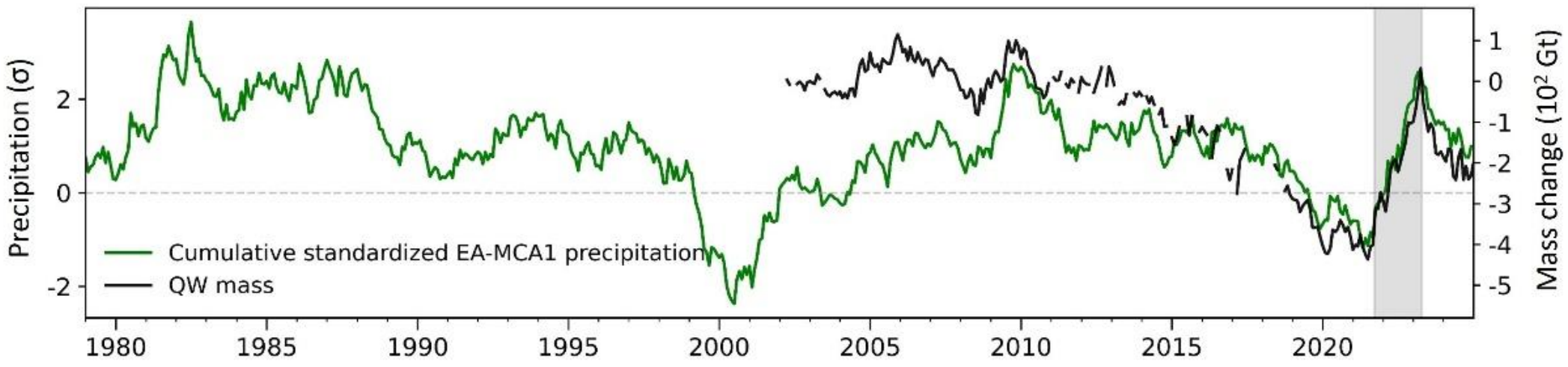

Resp Fig. 2.10. Comparison between the cumulative standardized precipitation time series of the first MCA mode (green) linking Southern Hemisphere Z300 and East Antarctic precipitation and the QW mass change time series from GRACE (black).

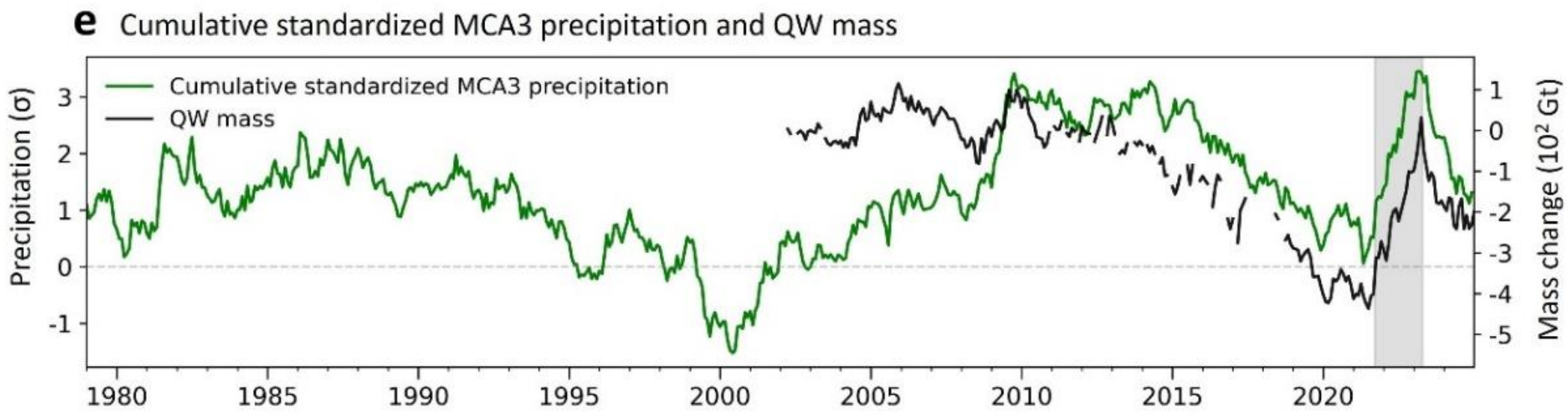

Resp Fig. 2.11 (also Fig. 2e in the main text). Comparison between the cumulative standardized MCA3 precipitation time series (green) and the QW mass change time series (black).

L231–233: "The correlation between the MCA3 Z300 time series and global SSTs further suggests a potential linkage between the dipole circulation and SST warming over the TWP, accompanied by SST cooling over the Indian Ocean and eastern Pacific (Extended Data Fig. 5c)." Again, is this result similar to what is obtained when using the first MCA mode between Southern Hemisphere Z300 and East Antarctic precipitation?

The SST correlation pattern (Resp Fig. 2.12) based on the first MCA mode linking Southern Hemisphere Z300 and East Antarctic precipitation is similar to that of MCA3 (Resp Fig. 2.13), although somewhat weaker, including warming over the TWP and cooling over the Indian Ocean and eastern Pacific. This suggests that the tropical SST linkage is also present in the EA-focused MCA1, which further supports our main result. In our paper, we make it clear that TWP warming plays a key role in generating the dipole structure over East Antarctica. However, La Niña-like cooling and IOD-like cooling may also contribute to the broader tropical SST background by increasing the relative magnitude of TWP warming and

consequently amplifying its impact to some extent. This may help explain why the tropical SST pattern related to the EA-focused MCA1 is slightly weaker than that related to MCA3. The EA-focused MCA1 mainly captures East Antarctic precipitation variability and naturally excludes any La Niña–West Antarctica-related signals. Because La Niña and TWP warming are significantly related during this period, part of the TWP-related signal may be statistically removed in the EA-focused MCA1 Z300 time series, which may explain the weaker SST correlation. This also indicates that statistical analysis alone is not sufficient to determine causality, and that it is necessary to further use other approaches, such as our idealized AGCM experiments, to test the inferred mechanism more directly. Our AMIP experiments show that TWP warming alone can reproduce the East Antarctic dipole and positive precipitation response, whereas La Niña-like cooling and IOD-like cooling alone cannot.

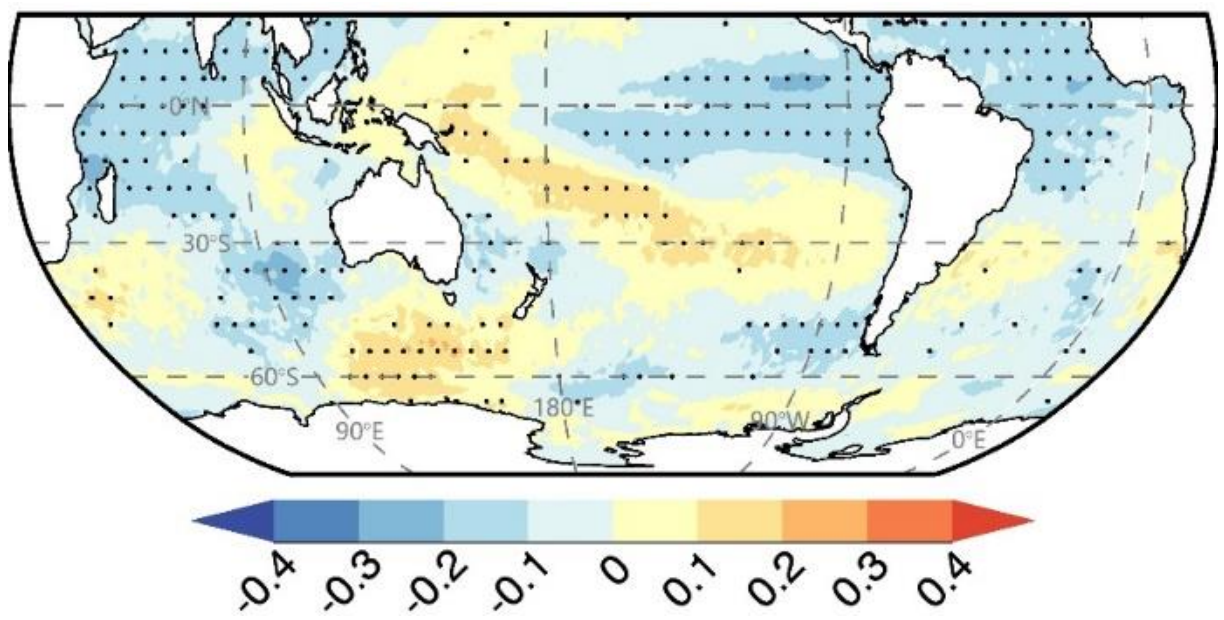

Resp Fig. 2.12. Correlation pattern of SST anomalies from ERA5 with the standardized MCA1 Z300 index linking Southern Hemisphere Z300 and East Antarctic precipitation for 1979–2024. The SST data are deseasonalized and detrended before the computation of the correlations. Stippling indicates regions significant at the 95% level based on an FDR-corrected Student's t test.

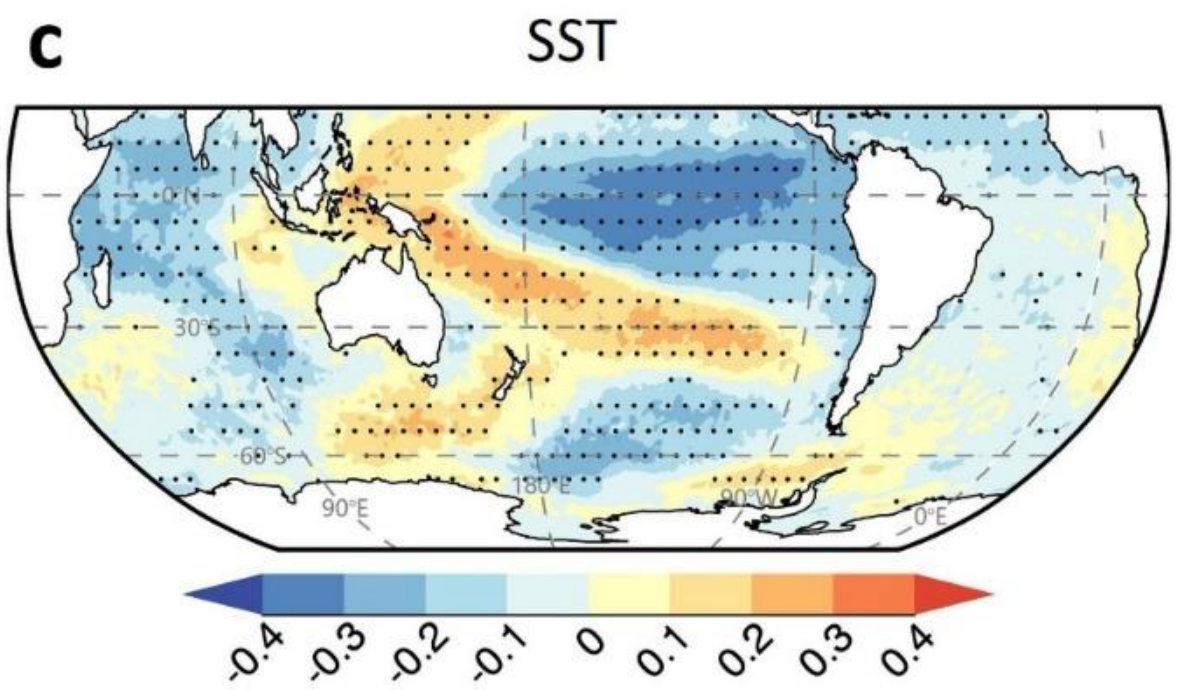

Resp Fig. 2.13 (also Extended Data Fig. 5c). Same as Resp Fig. 2.12 but for the correlation pattern of SST anomalies from ERA5 with the standardized MCA3 Z300 index linking Southern Hemisphere Z300 and Antarctic-wide precipitation.

L416: I think the discussion could benefit from a comparison and contrast between the high-pressure anomaly associated with the TWP and previous studies that have examined blocking events in East Antarctica. Blocking events have been linked to East Antarctic precipitation variability, e.g., King and Turner 1997; Walsh et al. 2000; Scarchilli et al. 2011; Udy et al. 2021.

Thank you for pointing us to these works and for drawing our attention to the relationship between the TWP-related high-pressure anomaly and the blocking events documented in previous studies (King and Turner, 1997; Walsh et al., 2000; Scarchilli et al., 2011; Udy et al., 2021). As you suggested, we acknowledge in the revised manuscript that the high-pressure center within our identified dipole structure is consistent with

previous literature. This high pressure provides favorable conditions for air masses to overcome the steep coastal topography, facilitating the meridional transport of Indian Ocean moisture into the Antarctic interior.

We also agree with Udy et al. (2021) that these local synoptic blocking systems can be modulated by large-scale climate variability. Building on this perspective, we believe our analysis provides a new extension of previous work. In our paper, we emphasize that the TWP-driven anomaly represents a prolonged, large-scale teleconnection forcing that can significantly modulate short-lived synoptic weather events, such as ARs, and induce significant precipitation anomalies. All these four studies have been cited in the revised main text [L188].

Udy, D. G., Vance, T. R., Kiem, A. S., Holbrook, N. J. & Curran, M. A. J. Links between Large-Scale Modes of Climate Variability and Synoptic Weather Patterns in the Southern Indian Ocean. J. Clim. 34, 883-899 (2021).

King, J. C. & Turner, J. Antarctic Meteorology and Climatology. (Cambridge University Press, 1997).

Walsh, K. J. E., Simmonds, I. & Collier, M. Sigma-coordinate calculation of topographically forced baroclinicity around Antarctica. Dyn. Atmos. Oceans 33, 1-29 (2000).

Scarchilli, C., Frezzotti, M. & Ruti, P. M. Snow precipitation at four ice core sites in East Antarctica: provenance, seasonality and blocking factors. Clim. Dyn. 37, 2107-2125 (2011).

Referee #3 (Remarks to the Author):

The authors present an analysis of the causes of the previously published reduction in Antarctic mass loss over 2020-2023. This is widely understood to be driven by SMB increases. Previous work has shown that this is largely contained in the Wilkes Land region of East Antarctica. They use reanalysis and models to suggest that the origin of this signal is associated with prolonged warming of the Tropical Warm Pool, resulting in transport of moisture to East Antarctica. This explanation is contrary (or additional?) to previous explanations surrounding a triple La Nina or more localised weather systems were responsible, or more generally attributed to increased atmospheric rivers. Of these, the association with the triple La Nina is most mechanistic with these previous studies being heavily reliant on correlation analysis.

Overall, I found the manuscript to be very well written and very clear, stepping through the analysis impressively. While the modelling and broader climatological aspects are not my strength, and hence this needs to be taken with a grain of salt, I thought the modelling and attribution to TWP was convincing.

Certainly, this work is the deepest analysis so far and the most convincing of the currently published studies, due to the detailed modelling work. That said, I am not sure the authors disproved a role of the triple La Nina or even increased ARs. And maybe they don't suggest that, but it is not entirely clear what they think on that point. The TWP SSTs are shown to have modest correlations with IOD and ENSO and weaker with PDO (L406). I wonder if the cumulative indices have similar or different correlations, given that it is the cumulative effect that matters to the ice sheet, as the authors note. After all, a cumulative TWP effect is being linked to the cumulative SMB.

I note that the precip from the TWP perturbation experiments (Fig 3c) fell well to the east of the observed region and in an even more narrow region - I figure that is within the bounds of model uncertainty. This does cause me to encourage the authors to ensure their conclusions clearly pertain to quite a narrow part of EAIS, let alone the entire AIS. Of course, the overall mass of hte ice sheet is affected.

I found the work that suggested that TWP events may have only slightly increased since the pre-industrial and will only slightly increase further in the future to be interesting. Can the authors say or speculate if these events may be getting bigger in time due to the larger moisture capacity of the atmosphere? That is, near-constant frequency but increasing cumulative SMB impact.

Finally, I found the analysis of the observational and SMB data to be generally robust, although there are a few places where refinements to the text are required, as I detail below.

We sincerely thank you for dedicating your time to evaluating our work and for providing such encouraging feedback. We have carefully incorporated these suggestions into the revised version. We agree that these comments are vital in improving the clarity and rigor of our findings. Because your major points emphasize four aspects, we briefly address each of them below.

Point 1: Regarding the role of TWP vs. triple La Niña and ARs
Thank you for highlighting this point. You are correct that the triple La Niña and atmospheric rivers (ARs) are important contributors to Antarctic SMB variability. Our results also suggest that increased AR landfalls over the EAIS were critical to the SMB gain during the event, consistent with previous studies. Furthermore,

our work suggests that the TWP-forced teleconnection plays an important role in providing large-scale control over these AR-driven precipitation extremes. The TWP-related dipole circulation enhances poleward moisture transport and increases AR activity toward East Antarctica. We have clarified this in the revised text [L226–229 and L304–307]

Our work does not conflict with the proposed role of the triple La Niña in Wang et al. (2025), which is suggested to induce a basin-wide Ferrel-cell adjustment responsible for the mass gain. In our interpretation, their finding of a Ferrel-cell adjustment may reflect changes in the intensity of the midlatitude jet over the Indian Ocean sector. If it is the case, this is consistent to our finding that the establishment of the dipole structure is associated with a jet-stream response. We examine our mechanism using AGCM experiments and CESM1-LE simulations. These results suggest that TWP warming alone can reproduce the dipole circulation pattern and the positive precipitation response over East Antarctica, whereas La Niña-like cooling alone cannot (Resp Fig. 3.1b). La Niña and the IOD likely contribute through other pathways, such as cooling surrounding tropical regions and thereby increasing the relative SST warming over the TWP. This enhanced SST contrast may help trigger Rossby waves from the TWP, promoting the Antarctic dipole and driving extreme precipitation over East Antarctica (Resp Fig. 3.1a). We have clarified this in the revised manuscript [L361-366]

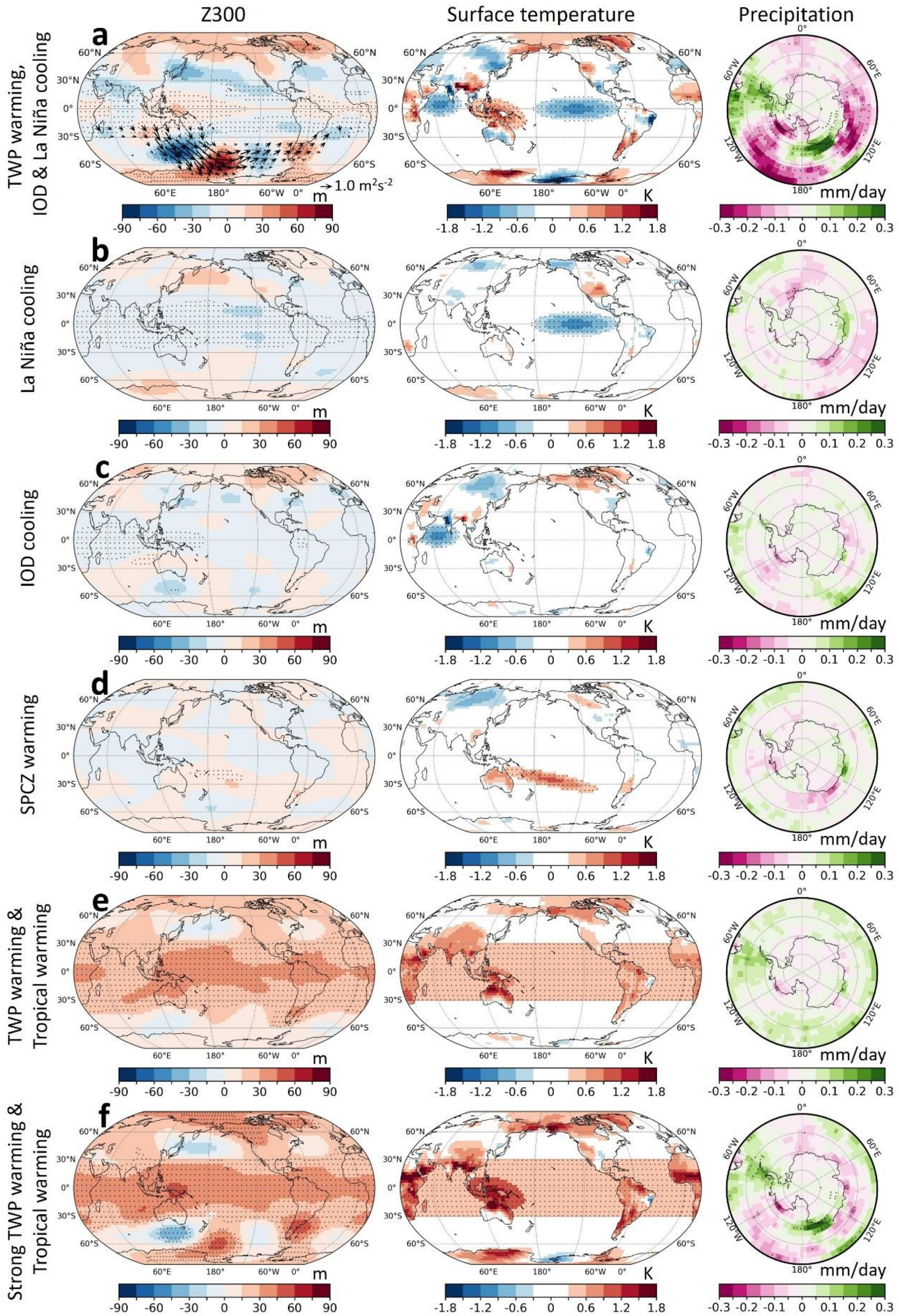

*Resp Fig. 3.1 (also Extended Data Fig. 8). ECHAM5 simulated atmospheric and surface responses to tropical forcing. **a**, Responses of Z300 (left), surface temperature (middle), and precipitation (right) to combined forcing by TWP warming, IOD cooling, and La Niña-like cooling. **b**, **c**, and **d**, As in **a**, but for isolated La Niña-like cooling (**b**), isolated IOD cooling (**c**), and SPCZ warming (**d**). **e**, As in **a**, but for TWP warming superimposed on a uniform tropical warming of 0.5 K between 30°S and 30°N. **f**, As in **e**, but for strong TWP warming. Stippling indicates regions significant at the 95% level based on an FDR-corrected*

Student's t test. The TWP is defined as a 15° clockwise-rotated ellipse centered at 135°E, 5°S, with semi-major and semi-minor axes of 35° and 15°, respectively. SST anomalies in the ellipses generally follow a gradient from 0.5 K (or −0.5 K) at the edge to 1 K (or −1 K) at the center, except for the strong TWP in ***e****, which ranges from 1 K at the edge to 1.5 K at the center.*

Point 2: Regarding the cumulative indices and correlations
Thank you for highlighting this point. You are correct that the cumulative effect matters to the ice sheet. In anomaly time series, persistent anomalies of the same sign produce step-like changes, whereas short-lived fluctuations largely cancel out over a period. The cumulative curve is therefore particularly effective for highlighting such prolonged events. However, when performing correlation analysis, we mainly use the original anomaly time series rather than the cumulative ones, because cumulative sequences have extremely low effective degrees of freedom. Using cumulative curves for correlation calculations makes it difficult to evaluate the associated statistical significance effectively.

Point 3: Regarding the spatial extent of precipitation
We agree that the simulated precipitation anomalies in our TWP experiments are narrower and shifted slightly east relative to observations. However, this model response is a 40-member mean, whereas observations represent one realization of the real world. The difference could reflect some limitations in our model setting, such as the lack of a coupled ocean in the AMIP-type setup, the underrepresentation of detailed topography due to the low model resolution, and the use of idealized anomalous SST forcing. It may also reflect the difference between one observed realization, represented by ERA5, and the 40-member mean response from the AMIP experiments. We also revised the text to emphasize that the precipitation anomalies induced by the TWP–East Antarctica teleconnection are mainly confined to the QW sector, rather than extending broadly across East Antarctica. For example, we changed "However, a sharp mass gain during 2021–2023, strongest in East Antarctica" to "however, a sharp increase in surface mass balance over Queen Mary Land and Wilkes Land (QW) in East Antarctica" [L29–30]. We also checked the full manuscript and revised the wording where needed to describe the affected region more precisely.

Point 4: Regarding the future impact and atmospheric moisture
As you suggested, we made further revisions to improve this part. We agree that changes in the frequency of TWP events are not the only way global warming may affect future TWP-related precipitation over the EAIS. Global warming may also influence the precipitation impact of future TWP-driven events through several more complex pathways, many of which may be model dependent. In the revision, we removed the CESM1 future event-frequency analysis, because future changes in the TWP–East Antarctica teleconnection are likely controlled by several coupled processes, including changes in high-latitude atmospheric moisture, AR intensity, storm tracks, precipitation phase, and ocean–atmosphere feedbacks. These processes are complex and require a more comprehensive coupled-model analysis, which is beyond the scope of the present study. In the revised manuscript, we therefore treat this issue as an open question. We also note that nonlinear effects should be examined in future work, because global warming may amplify circulation-driven ARs and precipitation into East Antarctica in a warmer and moister climate. Please see the revised manuscript at L374–376.

More substantial remarks
L31 and in the introduction: I did not see evidence in the paper that people have actually published that this is part of an expected response to warming. Maybe there are corridor conversations, but I don't think that's

enough to jump at. Certainly, the literature suggests that increased precipitation is expected with warming, but I've not seen a suggestion that this would be episodic and sudden.

Agreed. A consensus has not yet been established that the 2021–2023 East Antarctic mass gain reflects a long-term global warming signal, as this slowdown event is quite recent and many studies on this topic may still be underway. However, it is widely accepted in the community that Antarctic precipitation is expected to increase in a warmer climate (Nicola et al., 2023; Frieler et al., 2015; IPCC AR6). To be more accurate, we revised the Abstract to emphasize that the recent slowdown is "consistent" with the expected long-term precipitation response to global warming, rather than being "seen" as such a signal. This is a fair statement, as a number of studies, including IPCC AR6 chapters, stress that Antarctic precipitation is expected to increase under warming, although total Antarctic mass will likely continue to decline. Nicola et al. (2023) also stated in the first sentence of their abstract that "With progressing global warming, snowfall in Antarctica is expected to increase, which could counteract or even temporarily overcompensate increased ice-sheet mass losses caused by increased ice discharge and melting." Thus, in the polar community, an intuitive explanation for why East Antarctic mass loss slowed during 2021–2023 is that global warming may be driving this change. We revised the sentence in the Abstract from "has been seen as an early indicator of an expected long-term response to global warming" to "Although this slowdown is consistent with the expected long-term precipitation response to global warming through poleward-shifted storm tracks and Antarctic moistening" [L31–33].

Nicola, L., Notz, D. & Winkelmann, R. Revisiting temperature sensitivity: how does Antarctic precipitation change with temperature? Cryosphere 17, 2563-2583 (2023).

Frieler, K. et al. Consistent evidence of increasing Antarctic accumulation with warming. Nat. Clim. Change 5, 348-352 (2015).

IPCC. Regional fact sheet – Polar Regions. In Climate Change 2021: The Physical Science Basis. Contribution of Working Group I to the Sixth Assessment Report of the Intergovernmental Panel on Climate Change (2021).

L124 'since 2021' is overly loose language since it only applies to the data period being presented. so we need 'over 2021-2024'. please revise throughout if 'is' or 'since' is used.

Thanks, we have corrected 'since 2021' to ' between 2021–2024'. Following your suggestion, we have also thoroughly reviewed the entire manuscript, replacing similar terms. [L101]

L131, was there an unambiguous mass loss in QW before it paused? The authors show a decrease in precipitation in the decade prior, so overall mass loss from which to pause is a mischaracterisation. Also note the large uncertainties in background models (e.g., GIA models correcting GRACE are poorly constrained here) that mean GRACE rates could be biased at some level - this can be addressed through some tweaking of text.

Thank you for highlighting this issue. The QW ice-mass series shows a clear decade-long decline during about 2010–2020, followed by a pronounced reversal during 2021–2023. Given the uncertainty in background corrections such as GIA in this region, we revised the text from "pause in mass loss" to "reversal of the previous decade-long decline." [L106]

L141 that this event is 'rarely observed' is not demonstrated, and not clear from the figure. Rarely inside 20y? That is a very short period. Or is there another analysis that shows it is rare? Of course, rare, would contradict a return period of 10y shown later. So some revision to the text is required.

We thank you for pointing out this inconsistency. We have replaced "rarely observed" with " the most pronounced in the 20-year GRACE observational record". [L119]

L187 I don't see how a 2-3-year event can shape long-term mass variability. What am I missing?

Because ice mass is a cumulative variable, a strong and persistent 2–3-year anomaly can produce a large step-like jump in the mass curve and thus strongly affect longer-term mass variability. As this point has already been mentioned in an earlier paragraph, we have removed it here.

L366 I didn't see evidence that the TWP was affecting all EA, so should this be more tightly constrained geographically?

We agree. To be more accurate, we have replaced “EA” with “QW” [This part has been moved to L203 in the Extended Data] and throughout the manuscript wherever the described phenomena are specifically confined to this regional sector.

L380 'events' are only defined in the methods. Could do with a reference to methods and a very brief summary of how an event is defined.

Thank you for this suggestion. We added a brief definition after “events” and referred the reader to the Methods: “The recurrence of this mode is further assessed over longer observational and model-simulation records using an objective index of prolonged TWP SST warming, with events defined as a cumulative TWP SST anomaly increase of $\geqslant$4.0 K over 36 months (Methods).” [L343–345]

More minor remarks:
L42 'same regions' - not clear what regions are being referred to

We have deleted this sentence in the revised manuscript.

L59, while this is the reconciled number, the actual individual data does not agree within their uncertainties for EAIS. This could be reflected with a variant of this text

Thank you for highlighting this point. We revised “the EAIS has maintained a quasi-balanced state, with a mean trend of 3 ± 15 Gt/yr over the same period” to “the EAIS was quasi-balanced over the same period (3 ± 15 Gt/yr), though individual estimates diverge beyond their uncertainties” [L53–54]

L62 mCDW. I think you need citations here such as Rintoul et al 2025 for the Denman.

Thank you for bringing this study to our attention. It has been cited [L56]:

Rintoul, S. R., van Wijk, E. M., Herraiz-Borreguero, L. & Rosevear, M. G. Circulation and ocean–ice shelf interaction beneath the Denman and Shackleton Ice Shelves. Sci. Adv. 11, eadx1024 (2025).

L66 'relatively stable mass balance' where is this shown? There is work that shows large natural variability over all timescales in EAIS. See e.g., King and Watson GRL 2020

Thank you for this comment and for letting us know the important study by King and Watson (2020). That study clearly shows that Antarctic surface mass balance has large natural variability across various timescales. We agree that the phrase "relatively stable" was not accurate in conveying our original point. We revised the sentence from "During 2021–2023, the relatively stable mass balance over EA departed from its prior state, marked by the largest mass gain in the past two decades, leading to a hiatus in integrated Antarctic-wide mass loss despite persisting mass loss over WA" to "As a result, surface mass balance (SMB) in EA exhibits substantial natural variability across a wide range of timescales (King and Watson, 2020). During 2021–2023, EA experienced a large mass gain, leading to a slowdown in integrated Antarctic-wide mass loss over the past two decades, despite continuing mass loss in WA." [L61–64]

King, M. A. & Watson, C. S. Antarctic Surface Mass Balance: Natural Variability, Noise, and Detecting New Trends. Geophys. Res. Lett. 47, e2020GL087493 (2020).

Figure 1. i was surprised not to see RACMO in panels f-h given it is the superior SMB model. precip fields are available.

In light of your suggestion and Reviewer 4's point that GPCP is normally considered not suitable for Antarctic studies, we have updated Figure 1h (Resp Fig. 3.2h) by replacing the GPCP precipitation data with RACMO2.4. The results show that the precipitation fields from ERA5 and MERRA-2 are highly consistent with those from RACMO2.4.

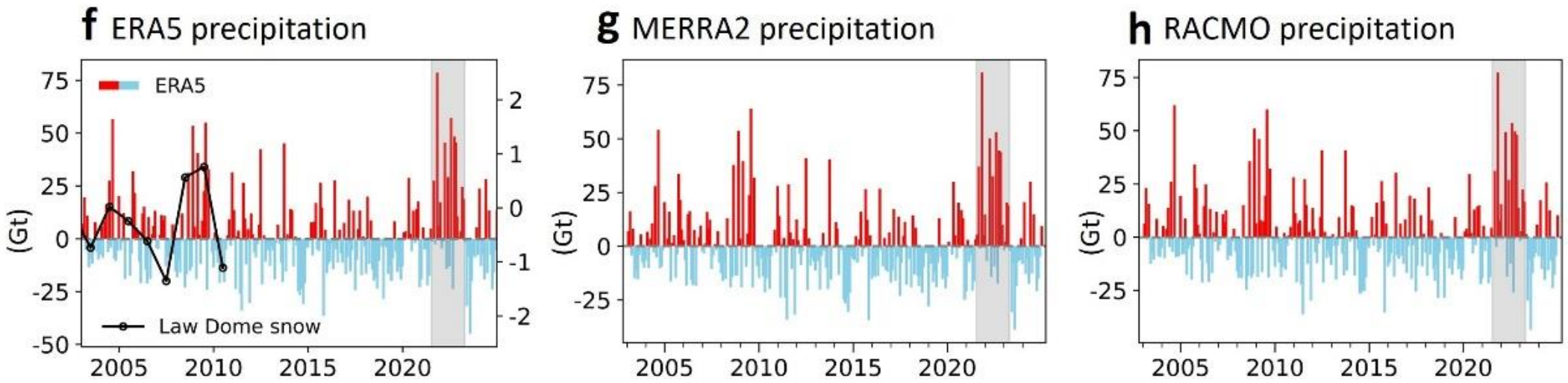

Resp Fig. 3.2 (also Fig. 1f-h in the main text). Monthly precipitation anomalies in the QW sector and Law Dome ice-core-derived snow accumulation. ***f–h****, Monthly precipitation anomaly time series over the QW sector from ERA5 (****f****), MERRA2 (****g****), and RACMO2.4 (****h****). Red and light blue bars represent positive and negative anomalies, respectively. Panel f additionally overlays the Law Dome ice-core snow-accumulation record, shown as standardized annual anomalies relative to 1979–2010 and referenced to the right y-axis. The grey shading highlights the mass reversal period (July 2021–April 2023).*

L165 'with QW precip exceeding one sd' -> 'where QW precip exceeding one s.d.'?

Thank you. We also incorporated the related comment from Reviewer 4 and revised "the 10 months of the event with QW precipitation exceeding one standard deviation" to "the 10 months during the event when the

area-weighted average QW precipitation exceeded the 80th percentile of the raw monthly distribution for 1979–2024" [L141–142].

L232 I was not sure if TWP was defined to this point

We would like to clarify that 'TWP' (tropical warm pool) was previously defined at Line 127.

L322 i thought the Kolbe et al paper coudl be cited here

We have added the citation to Kolbe et al. (2026) in the revised text. [Line 301]

Kolbe, M. et al. Atmospheric rivers and winter sea ice drive recent reversal in Antarctic ice mass loss. Commun. Earth Environ. (2026).

L360 where do i look to see the 2000-2002 jump?

Thank you for pointing out this issue. We have referred readers to Fig. 2e (Resp Fig. 3.3) to make our statement clearer: "Before 2003, another increase in QW precipitation (MCA3) occurred during 2000–2002 (Fig. 2e, green line)" [This part has been moved to L198–199 of the Extended Data]

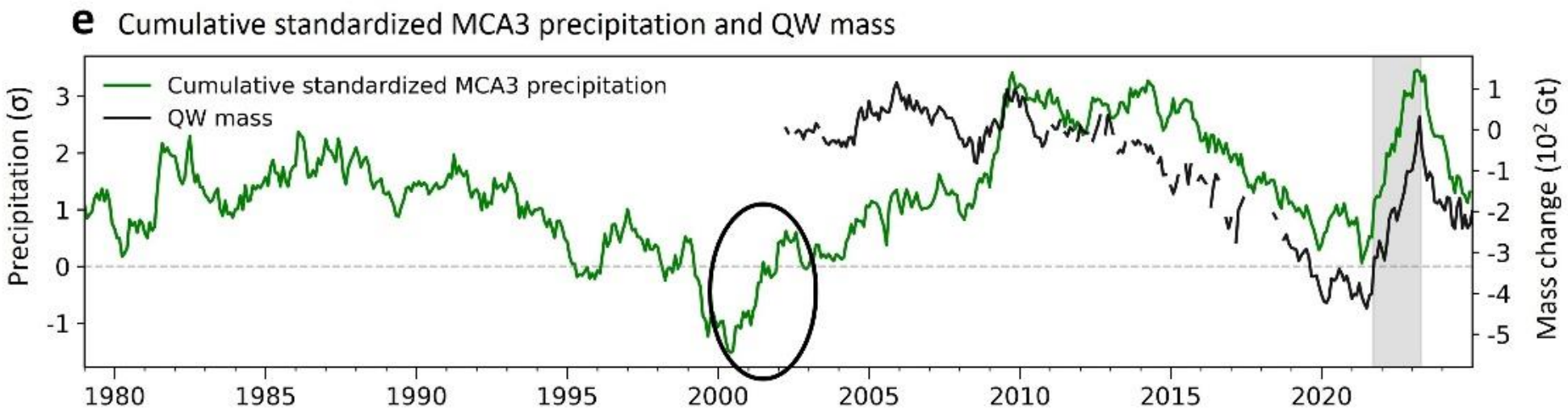

Resp Fig. 3.3 (also Fig. 2e in the main text). Comparison between the cumulative standardized MCA3 precipitation time series (green) and the QW mass change time series (black).

L446 add references

In the revised manuscript, we have substantially shortened and simplified the Discussion. The sentence referred to here has been removed.

L447 add references

In the revised manuscript, we have substantially shortened and simplified the Discussion. The sentence referred to here has been removed.

L456 i was not sure what was meant by 'more sensitive to oceanic changes'

Thank you for bringing this to our attention. We revised "more sensitive to oceanic changes" to the clearer expression "remains strongly affected by ocean-induced basal melt and dynamic ice loss." [L370–371]

Referee #4 (Remarks to the Author):

Review for the manuscript 'Prolonged tropical warm pool warming drives a slowdown in Antarctic mass loss' by Wang et al.

Wang et al. investigated the positive anomalies in Antarctic surface mass balance over the Queen Mary Land and Wilkes Land sector (QW) between July 2021 to April 2023. Based on the ERA5 reanalysis, the positive precipitation anomalies are linked to the third leading mode from a maximum covariance analysis (MCA3) between Antarctic precipitation and Z300 south of 23°N. Based on ECHAM5 simulations, the main dipole circulation patterns associated with MCA3, with a low pressure system to the south of Australia and a high pressure system close to QW, are linked to positive SST anomalies over the tropical warm pool (TWP) region. The manuscript is nicely written, and the results are presented clearly. However, I have some concerns about the novelty, the methodological approaches, the conclusion, and the implications, about which I would like to hear some thoughts from the authors. My comments are split into major and minor ones as below.

In summary, I am sceptical of the conclusion that the authors made in the title for two reasons: 1) The SST warming anomalies associated with the anomalous QW precipitation event are mainly located around the South Pacific Convergence Zone (SPCZ) rather than TWP, and the warming/cooling pattern resembles that of La Nina. The relationship between TWP/SPCZ warming and ENSO should be better clarified. 2) The causal-impact relationship between tropical SST warming and EA precipitation anomalies that the authors propose should be further supported by and compared quantitatively to past observations (e.g. ice core records of surface mass balance from Medley and Thomas, (2018)).

We sincerely thank you for the careful, thoughtful, and constructive evaluation of our manuscript. The comments were very helpful in improving the clarity, rigor, and balance of our analysis and interpretation. We hope these revisions address your concerns and make the interpretation of our conclusions clearer.

We agree that the two main concerns raised here are important. To address the first concern, we added new AGCM sensitivity experiments, including an SPCZ-only experiment, and compared them with the TWP-only, La Niña-only, IOD-only, and combined-forcing experiments. These results show that the TWP-only experiment successfully reproduces the East Antarctic dipole and positive QW precipitation response, whereas the SPCZ-only and La Niña-only experiments do not produce a comparable response. The detailed responses are provided in our replies to your Major Comment 1 and the minor comments labeled Lines 36, 41, 232, 237, 271, and 296 in your review.

To address your second point concerning the quality of Antarctic precipitation data, we have added several analyses using independent and longer-period datasets. We compared ERA5 QW precipitation with the Law Dome ice-core snow-accumulation record and replaced GPCP with RACMO2.4 precipitation in Fig 1. We also replaced the ERA-20C-based event composites with ERA5-based composites for 1950–2025, which provide more reliable modern reanalysis constraints. These composites, together with CESM1 simulations, show a consistent association between recurrent TWP warming, the East Antarctic dipole, and positive precipitation anomalies over QW. In addition, we also added analyses based on EKF400v2 and LMR v2.1 paleo-reanalyses. EKF400v2 is a monthly paleo-reanalysis spanning 1603–2003, which assimilates early instrumental observations, documentary indices, and tree-ring proxies using a Kalman-filtering approach. LMR v2.1 is the Last Millennium Reanalysis, which combines proxy records with climate-model

information through ensemble data assimilation. These analyses show that QW precipitation is associated with a similar East Antarctic high-pressure anomaly and an equatorward low-pressure anomaly over the past 400 to 2,000 years. The corresponding surface-temperature fields also show positive correlations over the TWP sector, using EKF400v2 2-m air temperature and LMR v2.1 SST. Together, these additions provide further support that the TWP–East Antarctic teleconnection is evident during 2021–2023 and recurs in longer records. Detailed responses are provided in our replies to your Major Comments 2 and 3 and your minor comments labeled Lines 131, 141, 165, 172, 239, and 264 in your review.

Major comments:

1. I am not a teleconnection expert, but I am aware of multiple articles discussing the tropical-Antarctic teleconnection through Rossby wave dynamics (e.g. Li et al., 2021). I am not sure whether the specific pathway between TWP and QW has been documented before, but the dynamics resemble the link between the Interdecadal Pacific Oscillation (IPO) with the Amundsen Sea Low (ASL) (see Fig. 2b from (Li et al., 2021)), though the locations are displaced. I am curious what is the link between TWP warming and El Niño–Southern Oscillation (ENSO) events. Could a strong La Nina also excite a Rossby wave train as shown in Fig 2a? Are the pressure anomalies and wave activity fluxes (WAF) associated with MCA3 also found in low latitudes in Fig 2a? Based on the current presentation, the Rossby wave seems more likely emanate from Indian Ocean.

We thank you for this insightful comment. We agree that tropical–Antarctic teleconnections through Rossby wave dynamics have been well documented in previous studies, including Li et al. (2021). However, the TWP–East Antarctic pathway identified in our study is dynamically distinct from those well-known tropical Pacific–West Antarctic pathways. Li et al. (2021) primarily discussed teleconnections between tropical Pacific variability, such as ENSO and IPO, and West Antarctica, which often operate through changes in the Amundsen Sea Low (ASL). By contrast, the remote tropical controls on East Antarctic precipitation and mass balance are much less well understood. Our study identifies a different pathway, in which SST anomalies over the tropical warm pool excite a Rossby wave train that preferentially affects East Antarctica, especially the Queen Mary Land and Wilkes Land sector (QW). This pathway differs spatially from the ENSO/IPO–ASL pathway and helps explain why the 2021–2023 mass-gain event was concentrated over East Antarctica rather than West Antarctica.

Regarding the relationship between TWP warming and ENSO. During 2021–2023, the tropical SST pattern included both TWP warming and La Niña-like cooling in the eastern Pacific. A strong La Niña can also excite a poleward-propagating Rossby wave train, but its wave source and propagation pathway are generally displaced eastward and more directly influence the Pacific sector and West Antarctica. In contrast, the TWP-related forcing is located farther west (Resp Fig. 4.1), and the associated Rossby wave train propagates toward the Indian Ocean sector of the Southern Ocean and East Antarctica. This distinction is important because the observed MCA3 pattern is characterized by a low-pressure anomaly south of Australia and a high-pressure anomaly over the East Antarctic coast, rather than by a canonical Amundsen Sea Low response. Furthermore, once the high-pressure anomaly is established over East Antarctica, it can be further maintained by active eddy–mean flow feedback in this region, in addition to the large-scale Rossby wave forcing (Resp Fig. 4.2).

Based on the AGCM sensitivity experiments, the TWP-only experiment successfully reproduces the observed north–south dipole circulation and positive precipitation response over East Antarctica (Resp Fig.

4.2). In contrast, the La Niña-like cooling-only and negative-IOD-like cooling-only experiments do not reproduce a significant East Antarctic dipole or a coherent positive precipitation anomaly over QW (Resp Fig. 4.3b,c). In addition, the combined experiment, which includes TWP warming, La Niña-like cooling, and IOD-like cooling, produces a response slightly stronger than that in the TWP-only experiment (Resp Fig. 4.3a versus Resp Fig. 4.2). These results suggest that prolonged TWP warming is the more direct tropical forcing for the East Antarctic dipole, while La Niña-like cooling and IOD-like cooling may increase the relative magnitude of TWP SST warming and thus amplify its impact to some extent.

Lastly, the apparent wave source over the Indian Ocean sector is consistent with our physical understanding of tropically-forced teleconnections. Local tropical heating can generate a Gill-type response with a pair of Rossby gyres to the west of the heating source (Gill, 1980). These Rossby gyres can then act as an effective source region for triggering stationary Rossby wave propagation toward higher latitudes. Therefore, the fact that the diagnosed WAF appears to originate mainly from the Indian Ocean sector, rather than directly from the center of the TWP, is in line with classical tropical wave dynamics. In addition, stationary Rossby wave dynamics indicate that tropical forcing always tends to affect high-latitude circulation downstream, at the same longitude or propagating eastward, of the forcing region. Therefore, eastern Pacific SST forcing more readily influences the Pacific sector and West Antarctica, whereas TWP forcing is more favorably positioned to influence the Indian Ocean sector, East Antarctica, and downstream regions. The observed TWP–EA teleconnection is therefore consistent with these physical principles. We have revised the manuscript to clarify the distinction between the TWP–EA pathway and the well-known ENSO–ASL pathway, and to explain why the diagnosed WAF source appears strongest over the Indian Ocean sector. Please see the revised manuscript at L257–260, L277–282, and L351–366.

Gill, A. E. Some simple solutions for heat-induced tropical circulation. Q. J. R. Meteorol. Soc. 106, 447-462 (1980).
Li, X. et al. Tropical teleconnection impacts on Antarctic climate changes. Nat. Rev. Earth Environ. 2, 680-698 (2021).

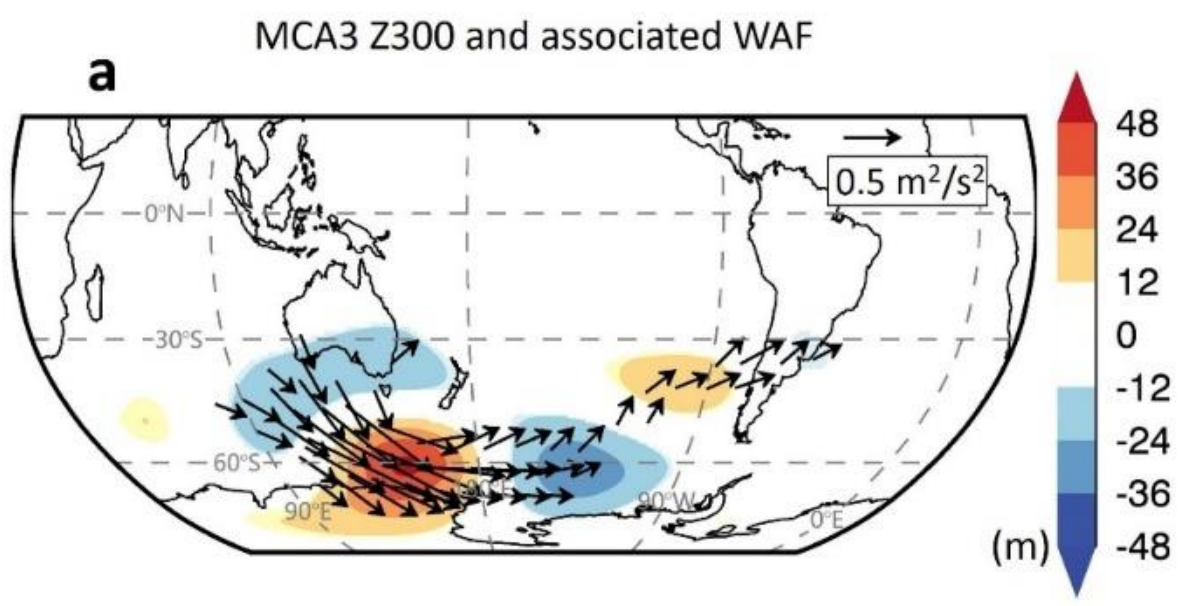

Resp Fig. 4.1 (also Fig. 2a in the main text). Spatial pattern of the third MCA mode for SH Z300. MCA is applied to deseasonalized and detrended ERA5 monthly fields (south of 23°N for Z300) and Antarctic precipitation for the period 1979–2024; the third mode explains 8% of the total squared covariance. Black vectors denote the associated Rossby wave activity flux (WAF). Note that the MCA results are insensitive to whether the long-term trend is removed.

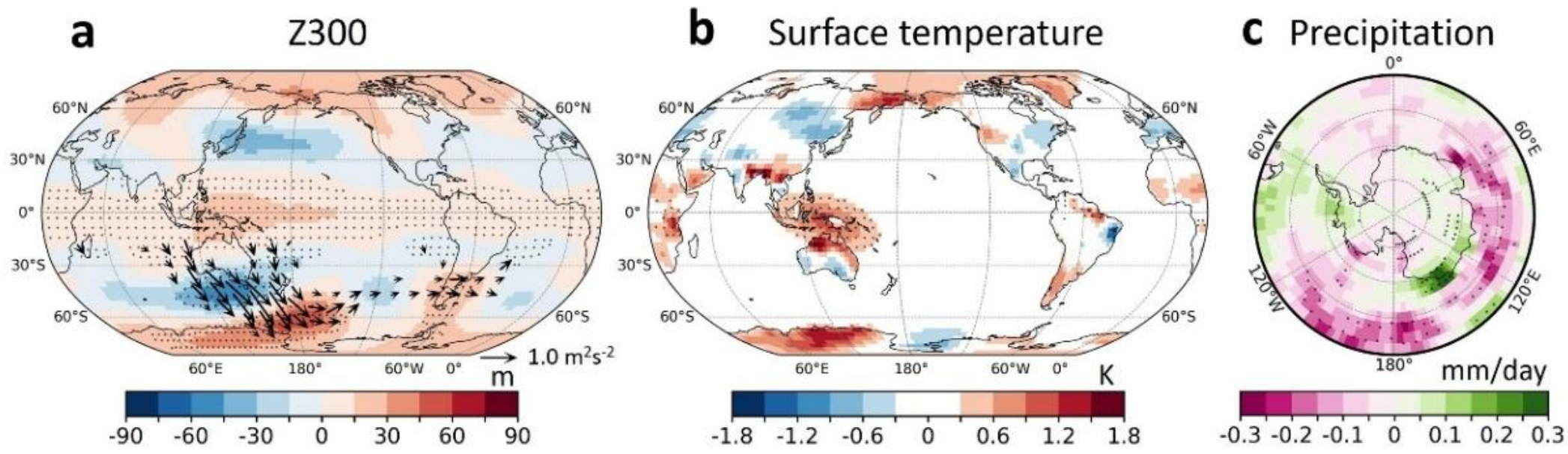

Resp Fig. 4.2 (also Fig. 3 in the main text). Simulated EA-TWP teleconnection using ECHAM5. Simulated responses to positive TWP SST anomalies, shown as the difference between the 40-year mean of the sensitivity experiment and that of the control experiment. ***a–c****, Responses of Z300 (****a****), surface temperature (****b****), and precipitation (****c****). Black vectors indicate WAF. Stippling indicates regions significant at the 95% level based on an FDR-corrected Student's t test. The TWP is defined as a 15° clockwise-rotated ellipse centered at 135°E, 5°S, with semi-major and semi-minor axes of 35° and 15°, respectively. Positive SST anomalies within the ellipse increase from 0.5 K at the edge to 1 K at the center.*

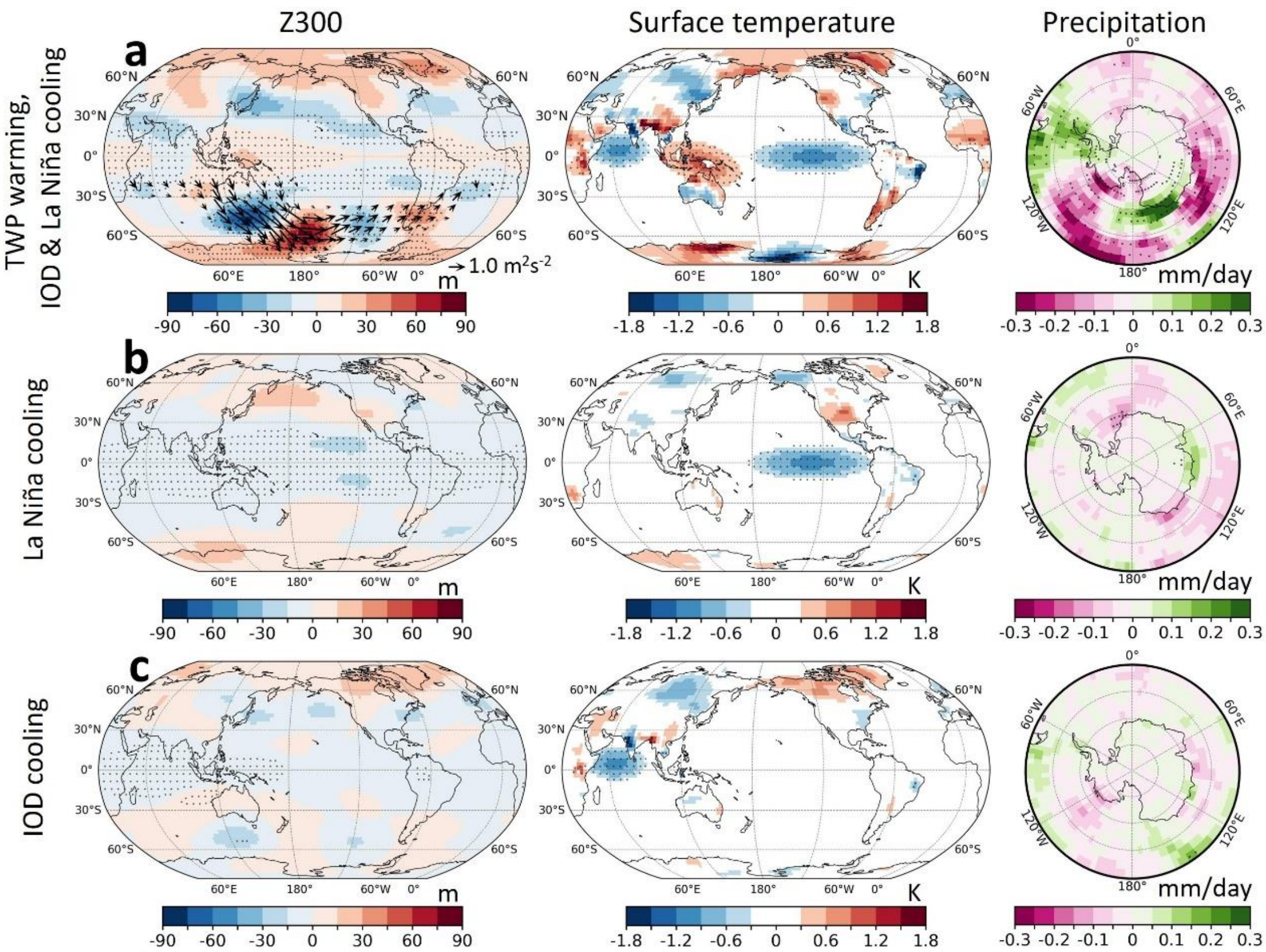

Resp Fig. 4.3 (also Extended Data Fig. 8a-c). ECHAM5 simulated atmospheric and surface responses to tropical forcing. ***a****, Responses of Z300 (left), surface temperature (middle), and precipitation (right) to combined forcing by TWP warming, IOD cooling, and La Niña-like cooling.* ***b****,* ***c****, As in* ***a****, but for isolated La Niña-like cooling (****b****) and isolated IOD cooling (****c****). Stippling indicates regions significant at the 95% level based on an FDR-corrected Student's t test. The TWP is defined as a 15° clockwise-rotated ellipse centered at 135°E, 5°S, with semi-major and semi-minor axes of 35° and 15°, respectively. SST anomalies in the ellipses generally follow a gradient from 0.5 K (or −0.5 K) at the edge to 1 K (or −1 K) at the center.*

2. Surface mass balance from regional models and precipitation from global reanalyses and GPCP should be

complemented by observations for quantitative analysis. Uncertainties in the GRACE/GRACE-FO estimates of QW mass balance should be reported and compared with observations as well. These observations could include surface mass balance stakes (Wang et al., 2025a), CloudSat snowfall retrievals (Palerme et al., 2017), ice core measurements (Medley and Thomas, 2018), and potential direct precipitation observations. Each of these datasets would contain their own uncertainties, but they should be helpful to further constrain the magnitude of mass balance gain during this period measured by GRACE/GRACE-FO. GPCP is normally considered not suitable for Antarctic studies, as can be seen from its large differences from other reanalyses. I understand the current datasets may be enough to showcase qualitatively the anomalous precipitation accumulation, but I think it is necessary to search for longer records to check whether similar events occurred in the past.

Thank you for suggesting these valuable observational datasets and references. We have added the uncertainty for the GRACE/GRACE-FO mass changes in the revised manuscript. The QW mass gain during July 2021–April 2023 is now reported as 470.3 ± 29.1 Gt. We also compared this GRACE/GRACE-FO estimate with independent surface-input estimates over the same sector and period, including RACMO SMB (348.3 ± 25.3 Gt), MAR SMB (346.3 ± 24.8 Gt), and ERA5 precipitation (351.2 ± 26.2 Gt).

We further considered the Antarctic accumulation reconstruction you suggested. In our search, the relevant Medley and Thomas regional reconstruction is the 2019 dataset, which extends only to 2000 and therefore does not cover the GRACE/GRACE-FO era. We therefore used the original Law Dome ice-core snow-accumulation record compiled in Thomas et al. (2017), which extends to 2010, to compare with ERA5 precipitation. Law Dome is located at 112.81°E, 66.77°S, near the central part of the QW sector, and is marked by the black star in Fig. 1c in the main text. The comparison, shown in Resp Fig. 4.4 and now added as Extended Data Fig. 1b, shows good agreement in interannual variability between annual ERA5 QW precipitation anomalies and Law Dome snow-accumulation anomalies over their overlapping period. The Law Dome record is highly correlated with both ERA5 QW precipitation anomalies and ERA5 Law Dome precipitation anomalies, with $r = 0.83$ ($p < 0.05$) for both comparisons. We also overlaid the Law Dome record on the ERA5 precipitation time series in Fig. 1f of the main text (Resp Fig. 4.5f) to provide additional observational support for the reliability of the precipitation variability shown in ERA5. Following your suggestion and Reviewer 3's related comment, we have also updated Fig. 1h in the main text (Resp Fig. 4.5h) by replacing the GPCP precipitation series with RACMO precipitation. The results show that the precipitation anomalies from ERA5 and MERRA-2 are highly consistent with those from RACMO. Please see the revised manuscript at L107–109 and 116–117, Fig. 1e, and Extended Data Fig. 1b in the revised manuscript.

Thomas, E. R. et al. Regional Antarctic snow accumulation over the past 1000 years. Clim. Past 13, 1491-1513 (2017).

Medley, B. & Thomas, E. R. Increased snowfall over the Antarctic Ice Sheet mitigated twentieth-century sea-level rise. Nat. Clim. Change 9, 34-39 (2019).

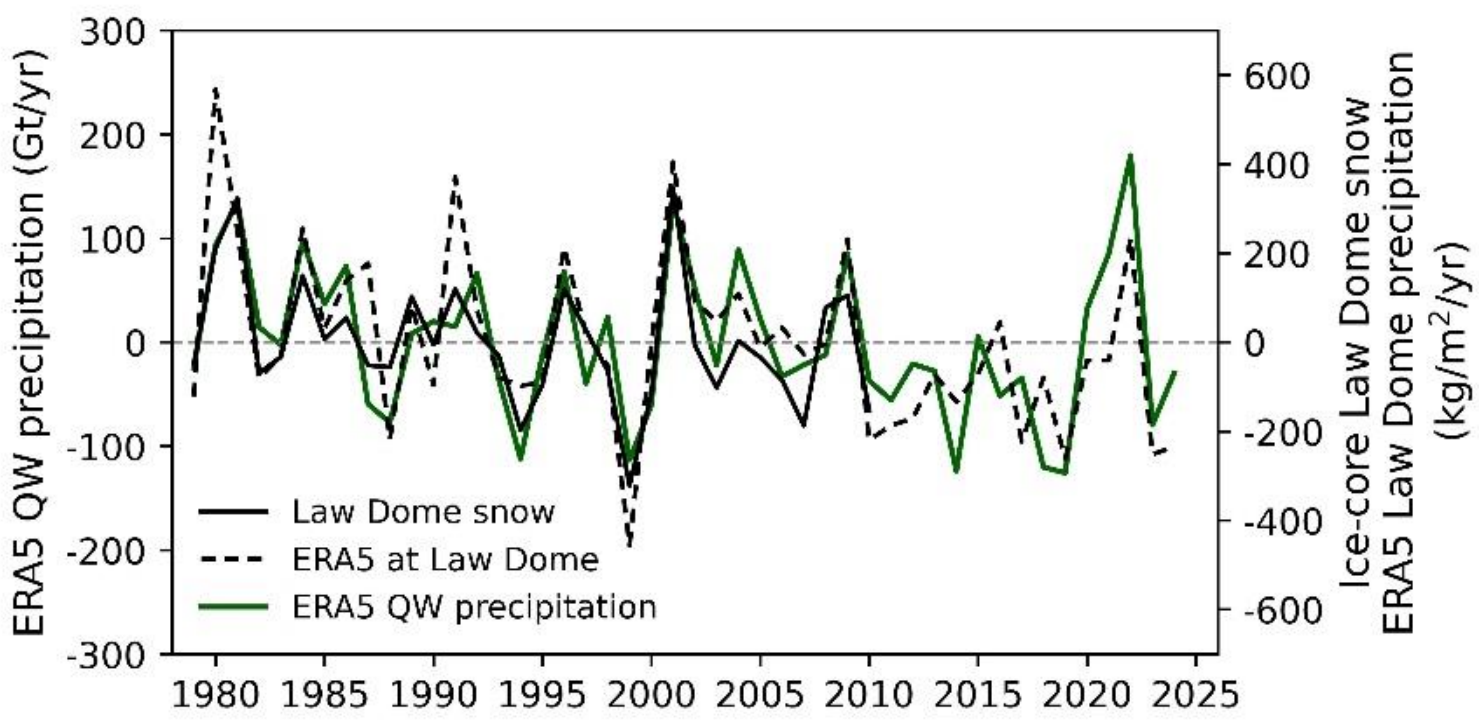

Resp Fig. 4.4 (also Extended Data Fig. 1b). Annual ERA5 QW precipitation anomalies, Law Dome ice-core snow-accumulation anomalies, and ERA5 precipitation anomalies at the Law Dome grid point. Law Dome anomalies are relative to the 1979–2010 mean. The left axis shows ERA5 QW precipitation in Gt yr^{-1}, and the right axis shows Law Dome snow accumulation and ERA5 Law Dome precipitation in kg m^{-2} yr^{-1}. The Law Dome record is highly correlated with ERA5 QW precipitation and ERA5 Law Dome precipitation anomalies, with $r = 0.83$ ($p < 0.05$) for both comparisons.

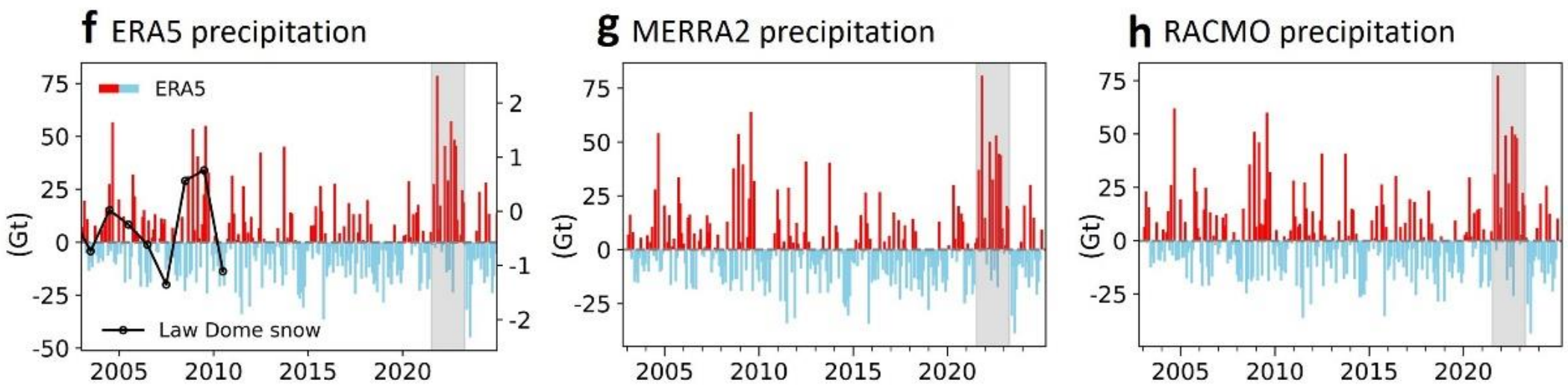

Resp Fig. 4.5 (also Fig. 1f-h in the main text). Monthly precipitation anomalies in the QW sector and Law Dome ice-core-derived snow accumulation. ***f–h****, Monthly precipitation anomaly time series over the QW sector from ERA5 (****f****), MERRA2 (****g****), and RACMO2.4 (****h****). Red and light blue bars represent positive and negative anomalies, respectively. Panel f additionally overlays the Law Dome ice-core snow-accumulation record, shown as standardized annual anomalies relative to 1979–2010 and referenced to the right y-axis. The grey shading highlights the mass reversal period (July 2021–April 2023).*

3. The authors link anomalous QW precipitation to MCA3, and then to TWP warming, which recurs every ~10 years based on the ERSSTv5 and CESM1. Since the authors conclude the TWP warming drives the anomalous QW precipitation, are there evidences that there are recurrent anomalous QW precipitation that slows down Antarctic mass loss. So a null hypothesis could be, the Rossby wave train induced by the TWP warming favours a high pressure system near QW, which favours moisture transport but not necessarily induces stronger moisture transport. My understanding is that creating favourable conditions is different from a definitive causal-impact link, which implies consistent relationships and long-term impacts.

We agree that identifying favorable large-scale conditions is not the same as demonstrating a fully deterministic causal link. In Extended Data Fig. 12 (Resp Fig. 4.6), we composited not only the SST anomalies associated with TWP warming events, but also the corresponding Southern Hemisphere circulation and Antarctic precipitation anomalies. These composites show that recurrent TWP warming events are consistently accompanied by a similar high-latitude circulation response and by positive precipitation anomalies over the QW sector, in both ERA5 and CESM1 simulations.

To further test whether this circulation–precipitation relationship is robust beyond the modern reanalysis period, we added new analyses using two paleo-reanalysis products, EKF400 v2 and LMR v2.1. Specifically, we first constructed a QW precipitation index from each dataset and then correlated it with the corresponding large-scale fields in the same dataset. The Z500 correlations show a similar large-scale circulation structure in both products: an East Antarctic high-pressure anomaly and a low-pressure anomaly south of Australia (Resp Fig. 4.7a,c). This dipole-like pattern is consistent with the MCA3-related circulation pattern identified from ERA5 (Resp Fig. 4.1) and the 2021–2023 circulation anomaly (Resp Fig. 4.8). Despite differences in amplitude and spatial details, the repeated emergence of the East Antarctic high-pressure anomaly provides an independent long-record check of the link between QW precipitation and large-scale circulation beyond the modern reanalysis period. We also examined the temperature fields in these paleo-reanalyses. EKF400v2 does not provide SST, so we used its 2-m air temperature field as a indicator of large-scale surface temperature variability. The correlation shows positive temperature anomalies over the TWP sector (Resp Fig. 4.7b). In LMR v2.1, which includes SST, the QW precipitation index is also positively correlated with SST anomalies over the TWP sector (Resp Fig. 4.7d). These results further support that the QW precipitation-related circulation pattern is accompanied by broad tropical surface warming features consistent with the TWP–East Antarctic teleconnection. Please see L346–350 in the revised manuscript and L180–249 in the Extended Data.

East Antarctic snowfall is closely linked to synoptic systems over the southern Indian Ocean, and blocking conditions can strengthen poleward moisture transport and precipitation over East Antarctica (Udy et al., 2021; Pohl et al., 2021). Scarchilli et al. (2011) further showed that snowfall at East Antarctic ice-core sites is associated with Indian Ocean moisture advection under blocking conditions. Our results and previous studies support our interpretation that the East Antarctic dipole in our study is dynamically effective in transporting moisture into East Antarctica. We agree that the TWP-induced Rossby wave train should not be interpreted as producing the same precipitation response in every event. QW precipitation anomalies also depend on background circulation, synoptic storm activity, atmospheric moisture availability, and interactions with other climate modes. We therefore revised the manuscript to state that prolonged TWP warming favors the establishment of an East Antarctic high-pressure anomaly and enhanced moisture transport, increasing the likelihood of positive QW precipitation anomalies, rather than implying a deterministic relationship. Please see the revised manuscript at L226–229, L304–307 and L340.

Udy, D. G., Vance, T. R., Kiem, A. S., Holbrook, N. J. & Curran, M. A. J. Links between Large-Scale Modes of Climate Variability and Synoptic Weather Patterns in the Southern Indian Ocean. J. Clim. 34, 883-899 (2021).

Pohl, B. et al. Relationship Between Weather Regimes and Atmospheric Rivers in East Antarctica. J. Geophys. Res. Atmos. 126, e2021JD035294 (2021).

Scarchilli, C., Frezzotti, M. & Ruti, P. M. Snow precipitation at four ice core sites in East Antarctica: provenance, seasonality and blocking factors. Clim. Dyn. 37, 2107-2125 (2011).

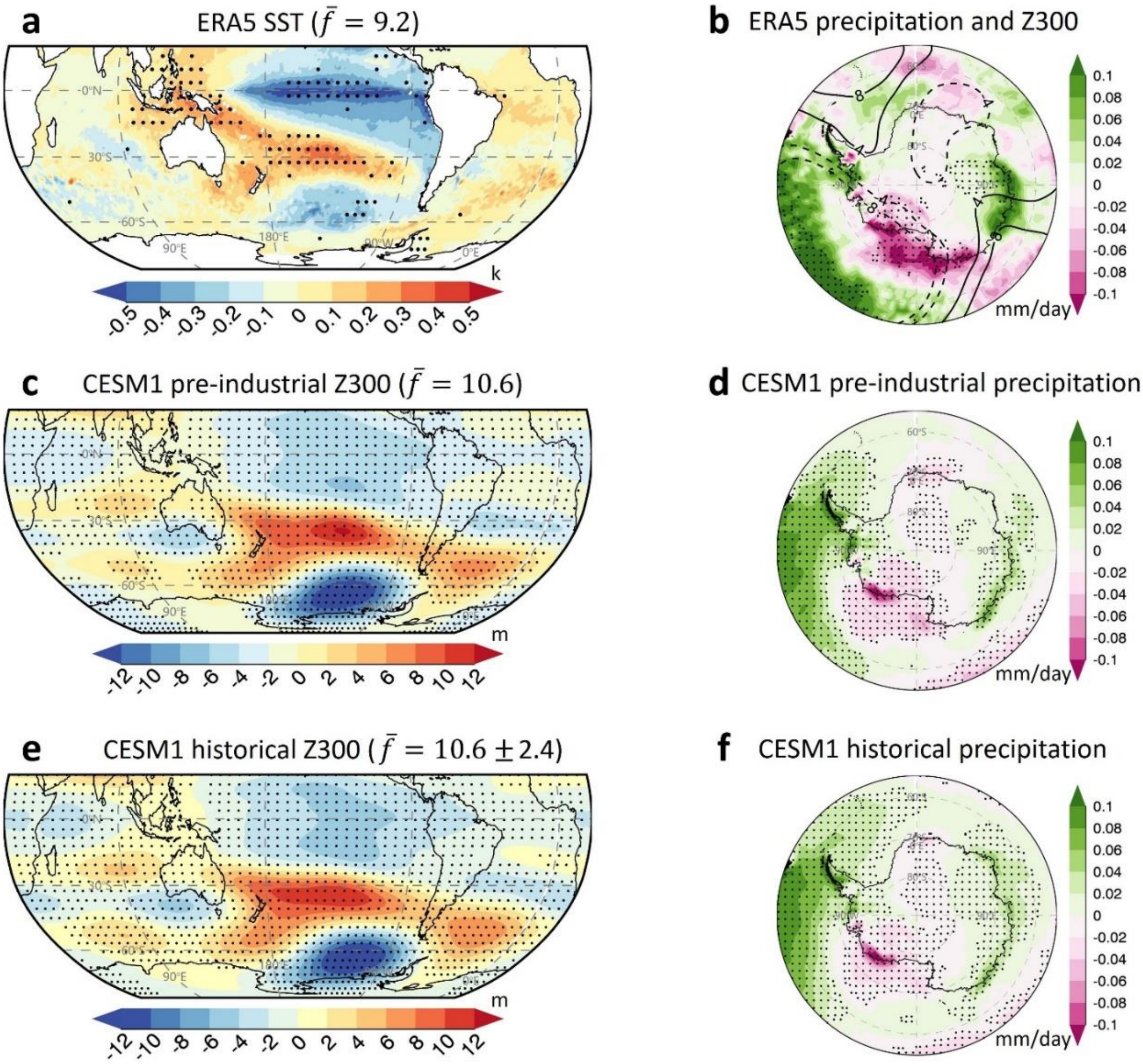

Resp Fig. 4.6 (also Extended Data Fig. 12). Composite anomalies during TWP warming events in observations and model simulations. ***a***, ***b***, *Composites based on TWP warming events identified using ERA5 SST for 1950–2025. SST anomalies (****a****). Precipitation anomalies overlaid with Z300 contours (****b****).* ***c***, ***d***, *Composites based on events identified in the 1,800-year CESM1 pre-industrial control run. Z300 anomalies (****c****). Precipitation anomalies (****d****).* ***e***, ***f***, *As in* ***c***, ***d***, *but for CESM1 historical simulations for 1920–2005. TWP warming events are identified from the cumulative TWP SST anomaly index after removing the EMD residual, which represents the long-term trend. SST, precipitation and Z300 composites are calculated from deseasonalized monthly anomalies, with the component associated with the EMD residual of the TWP SST index removed by linear regression. Stippling indicates regions significant at the 95% confidence level. Values in parentheses in the left-column panel titles indicate the mean frequency of TWP warming events in events per century. For CESM1 historical simulations, the uncertainty denotes the inter-member standard deviation of event frequency.*

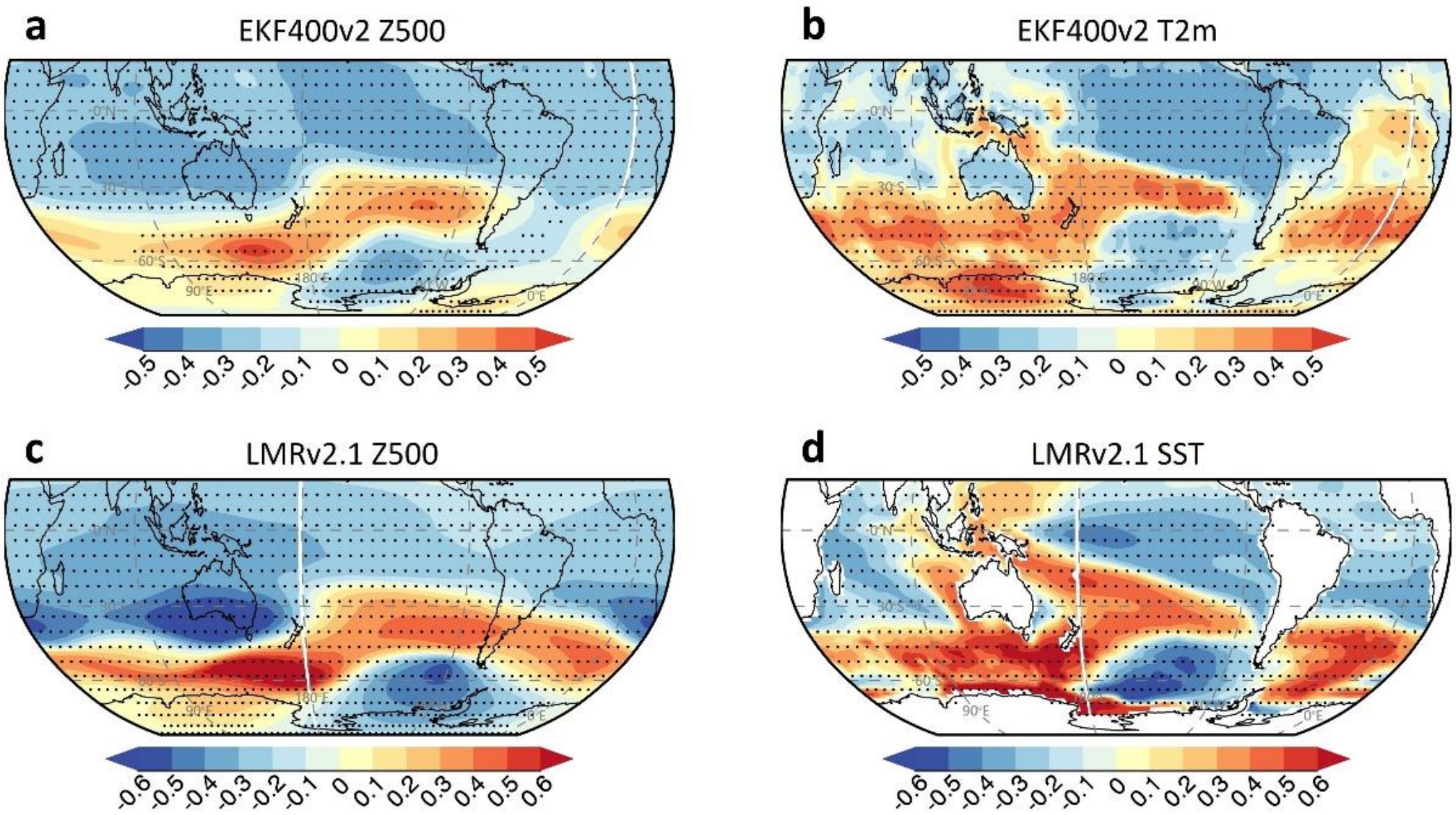

Resp Fig. 4.7 (also Extended Data Fig. 13). Annual-mean correlations between the QW precipitation index and large-scale climate fields in paleo-reanalysis products. ***a****, Correlation between the QW precipitation index and Z500 anomalies in EKF400v2.* ***b****, As in a, but for EKF400v2 2-m air temperature.* ***c****, As in* ***a****, but for Z500 anomalies in LMR v2.1.* ***d****, As in* ***c****, but for LMR v2.1 SST. EKF400v2 is a monthly paleo-reanalysis spanning 1603–2003, which assimilates early instrumental observations, documentary indices, and tree-ring proxies using a Kalman-filtering approach. Here, the monthly fields were averaged to annual means before the correlation analysis. LMR v2.1 is the Last Millennium Reanalysis, which combines proxy records with climate-model information through ensemble data assimilation. Because LMR v2.1 provides only annual fields and EKF400v2 does not include SST, these paleo-reanalyses cannot be used to recreate the TWP-event composite analysis shown in Extended Data Fig. 12. We therefore use these reanalyses to assess, through correlation analysis, whether the relationships among QW precipitation, the EA dipole, and tropical SST variability persist over longer periods. To reduce the influence of long-term nonlinear trends in these two datasets, we divide each dataset into multiple non-overlapping 40-year segments. Within each segment, both the QW precipitation index and the corresponding field were linearly detrended before the correlation analysis, and the correlation coefficients were then averaged across all segments. The QW precipitation index is defined as the area-weighted mean precipitation over 70°E–145°E, 65°S–75°S. Stippling indicates regions significant at the 95% level based on an FDR-corrected Student's t test.*

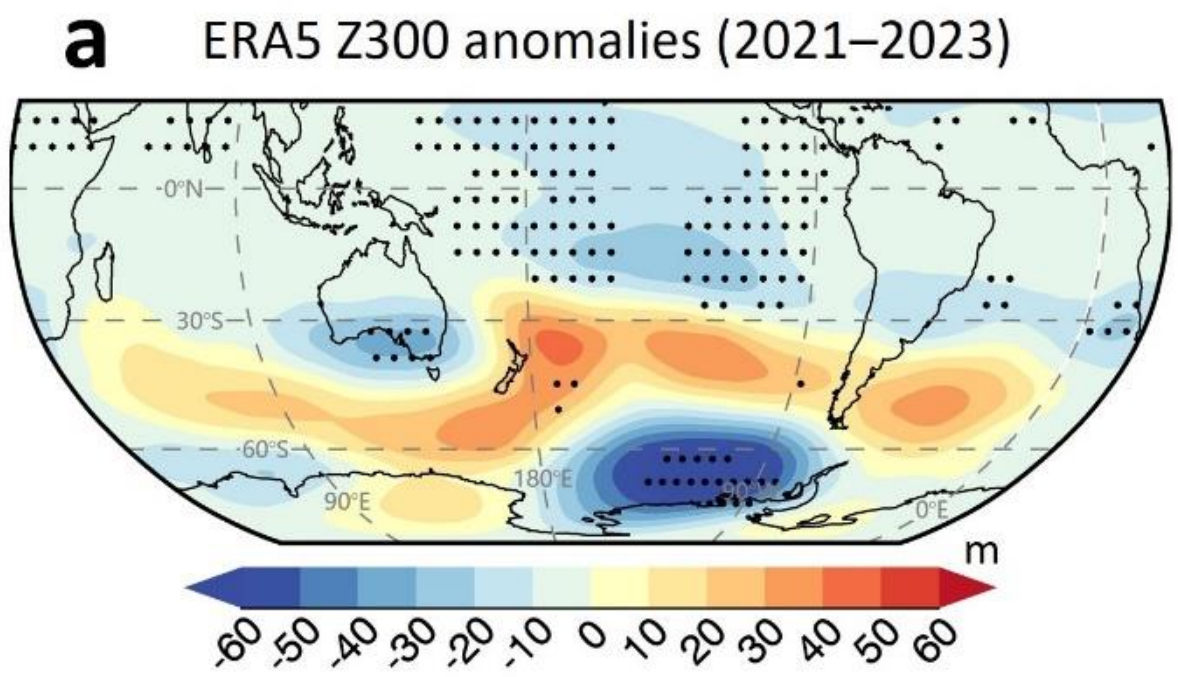

Resp Fig. 4.8 (also Extended Data Fig. 2a). Mean ERA5 Z300 anomalies during July 2021 to April 2023. The anomaly pattern is computed after removing the climatological seasonal cycle and linear trend over 1979–2024. Stippling indicates regions significant at the 95% level based on an FDR-corrected Student's t test.

Minor comments:

Line 36: Regarding 'unusually persistent warming' in Extended Data Fig. 2b: If the linear trend is not removed, is the warming more significant? Are there temporal variations during this period? Student's t test may be affected by false discovery rates, so it is suggested to use the Benjamini–Hochberg procedure alongside the test (Benjamini and Hochberg, 1995). The location of the warming resembles the South Pacific Convergence Zone (SPCZ). Could it be more directly linked to convective activities there?

Thank you for this helpful comment. We have applied the Benjamini–Hochberg false discovery rate (FDR) correction to the significance tests in the revised manuscript. We also examined the SST anomalies during July 2021–April 2023 both with and without removing the linear trend. Without detrending, the positive SST anomalies over the TWP-related region are stronger in amplitude (Resp Figs. 4.9), indicating that the event was superimposed on the long-term background warming. However, after removing the linear trend, the TWP warming pattern remains clearly positive.

This suggests that the 2021–2023 event cannot be explained by the long-term trend alone, but includes a substantial prolonged interannual anomaly component. We further quantified the temporal persistence of the warming using the monthly TWP index during July 2021–April 2023. The index is positive in 19 of the 22 months (86%), and exceeds +1 standard deviation in 12 of the 22 months (55%). These statistics show that the warming was not a short-lived fluctuation, but a sustained warm event with many months reaching moderate to strong intensity. Please see the caption of Extended Data Fig. 6 of the revised manuscript.

To assess the role of the SPCZ in forming the dipole mode, we conducted an additional ECHAM5 sensitivity experiment forced by positive SST anomalies over the SPCZ region, using the same framework as the other ECHAM5 experiments. This experiment does not reproduce the observed dipole circulation pattern over East Antarctica (Resp Fig. 4.10), and we have added it as Extended Data Fig. 8d. Although it produces positive precipitation anomalies over part of the QW sector, these are largely offset by negative anomalies within the same sector. The magnitude of the positive precipitation response over East Antarctica is much weaker than that driven by TWP warming. These results suggest that SPCZ-related convection alone is unlikely to be the primary driver of the observed 2021–2023 event. We therefore interpret the SPCZ-like signal in the related SST pattern as a feature accompanying the broader TWP warming and La Niña-like cooling pattern, rather than as the dominant forcing of the East Antarctic response. This interpretation is also consistent with the physical expectation that SST-forced tropical convection in the deep tropics can more effectively generate upper-level divergence and excite Rossby wave trains. By contrast, SST anomalies in the subtropics or along the SPCZ may not produce an equally effective teleconnection to high latitudes, because the convective response there is generally weaker and is less effective in generating upper-tropospheric divergence or convergence. We have clarified this point in the revised text [L277–282].

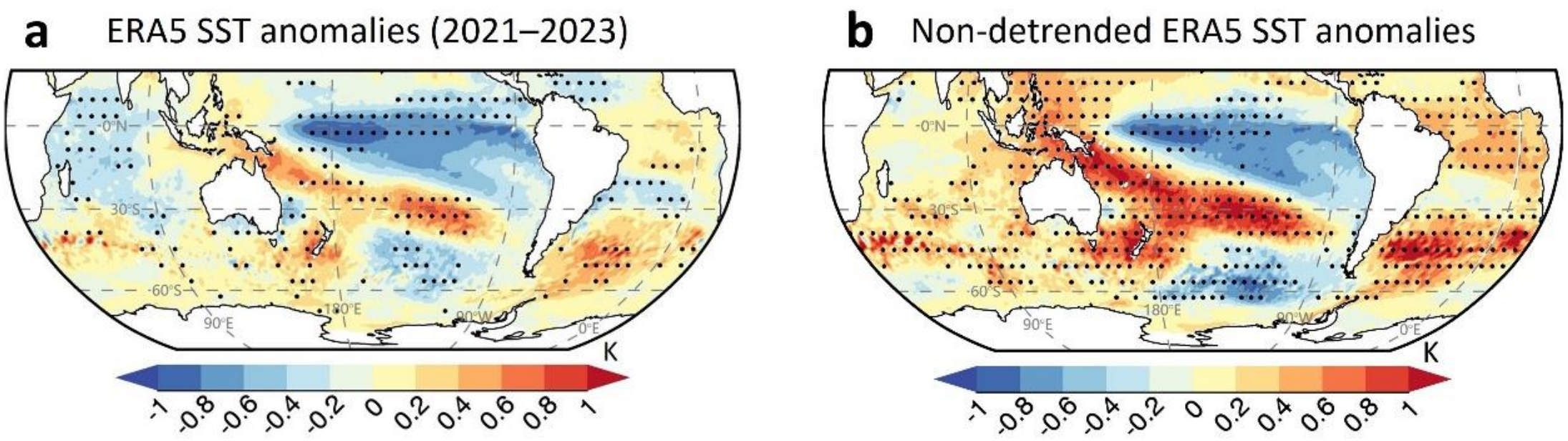

Resp Fig. 4.9. Mean ERA5 SST anomalies during July 2021–April 2023. ***a*** *(also Extended Data Fig. 2b), Anomalies computed after removing the climatological seasonal cycle and linear trend over 1979–2024.* ***b****, Anomalies computed after removing only the climatological seasonal cycle, with the long-term trend retained. Stippling indicates regions significant at the 95% level based on an FDR-corrected Student's t test.*

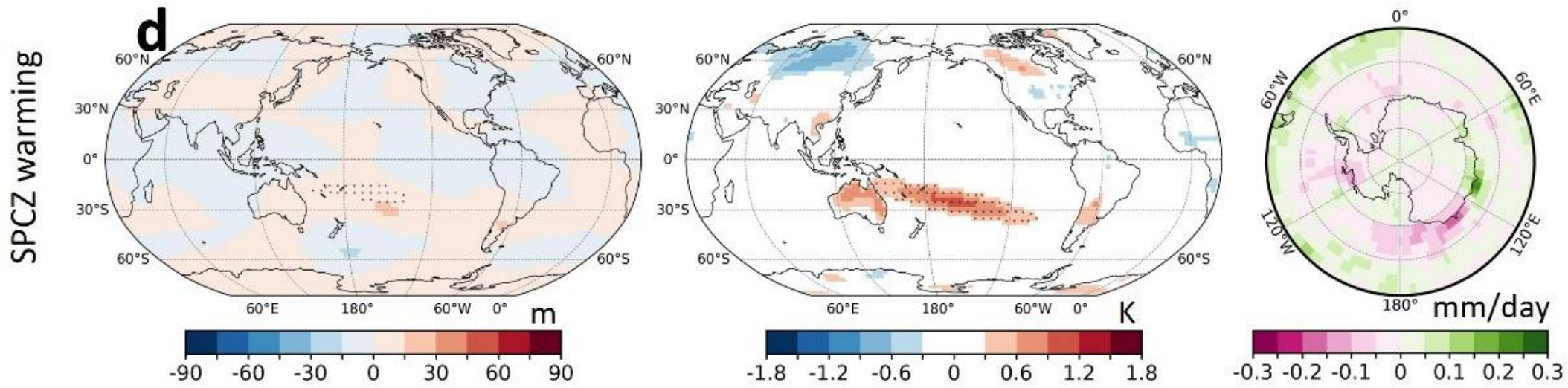

Resp Fig. 4.10 (also Extended Data Fig. 8d). ECHAM5 simulated atmospheric and surface responses to SPCZ-related SST forcing. Responses of Z300 (left), surface temperature (middle), and precipitation (right) to prescribed positive SST anomalies over the SPCZ region. SST anomalies in the ellipses generally follow a gradient from 0.5 K at the edge to 1 K at the center. Stippling indicates regions significant at the 95% level based on an FDR-corrected Student's t test.

Line 37: 'A blocking high': I only see Z300 anomalies rather than any high pressure system in the manuscript. During the whole period, is there a persistent high pressure system, or transient ones? I assume Z300 means geopotential height at 300 hPa, please define it. Why is 300 rather than 500 hPa used? I assume moisture is more abundant at 500 hPa, and thus moisture transport.

Thank you for this comment. Z300 denotes geopotential height at 300 hPa, and we have now defined it in the revised manuscript [L140, 172]. In the extratropical atmosphere, large-scale circulation anomalies are often characterized by a barotropic structure, so a positive geopotential height anomaly at 300 hPa is generally accompanied by a similar anomaly at mid- or lower-tropospheric levels. To verify this, we additionally examined the mean ERA5 Z500 anomalies during July 2021–April 2023. The Z500 field shows a similar dipole structure over East Antarctica, including a positive anomaly over the QW sector, consistent with the Z300 pattern (Resp Figs. 4.8 and 4.11). Due to the high topography of the EAIS, Z850 is below much of the surface of the ice sheet. We used Z300 because it is a standard upper-tropospheric level for diagnosing large-scale teleconnections and Rossby wave propagation. Circulation anomalies at this level usually have large amplitude and are less affected by Antarctic topography and the high elevation of the ice sheet itself than lower-level fields. Z300 also clearly captures the circulation anomalies linked to tropical forcing, because tropical forcing can excite Rossby wave responses that are very apparent in the upper troposphere through divergent outflow or convergent inflow and wave propagation. Moisture transport itself is diagnosed separately using IVT and AR frequency, rather than inferred directly from Z300. Please see the revised manuscript at L172–173.

In addition, we do not mean that a single blocking event persisted continuously throughout the entire July 2021–April 2023 period. Rather, the period mean shows a persistent positive geopotential height anomaly over East Antarctica, indicating a sustained large-scale anticyclonic circulation state in which short-timescale high-pressure anomalies are much more likely to occur than in the long-term mean. We have revised the term 'blocking high' to 'high-pressure anomaly' in the revised manuscript, including L38.

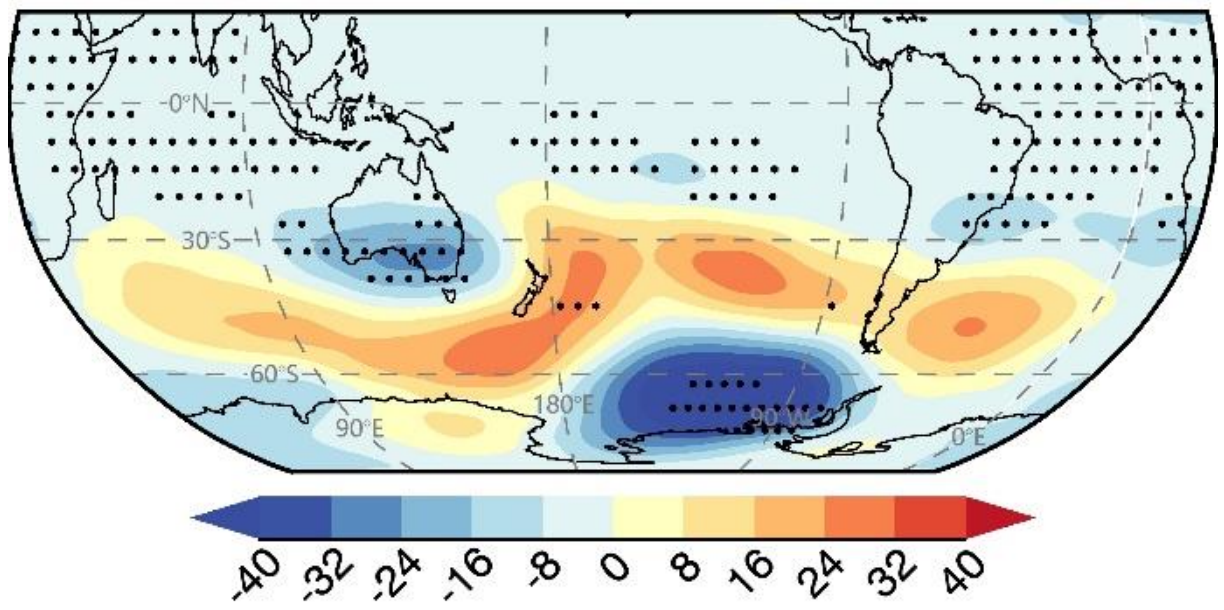

Resp Fig. 4.11. Same as Resp Fig. 4.8, but for the Z500.

Line 41: The authors attempt to define a new tropical mode with influence distinct from global warming and other tropical modes. However, the signature positive SST anomalies are located closer to SPCZ rather than TWP, and differences are generally not significant. Based on the Methods section 'Identification of TWP warming events', it is hard to confirm the independence of TWP warming events from La Nina conditions.

To address this point, we conducted an additional ECHAM5 sensitivity experiment forced by positive SST anomalies over the SPCZ region, using the same framework as the other ECHAM5 experiments. This experiment does not reproduce the observed dipole circulation pattern over East Antarctica (Resp Fig. 4.10). Although it produces positive precipitation anomalies over part of the QW sector, these are largely offset by negative anomalies within the same sector. The magnitude of the positive precipitation response over East Antarctica is much weaker than that driven by TWP warming. These results suggest that SPCZ-related convection alone is unlikely to be the primary driver of the observed 2021–2023 event. We therefore interpret the SPCZ signal in the MCA3-related SST pattern as a feature accompanying the broader TWP warming and La Niña-like cooling pattern, rather than as the dominant forcing of the East Antarctic response. This interpretation is also consistent with the physical constraint that SST-forced tropical convection in the deep tropics can more effectively generate upper-level divergence and excite extratropical Rossby wave trains. By contrast, SST anomalies in the subtropics or along the SPCZ may not produce an equally effective teleconnection to East Antarctica, because convection over the SPCZ region is generally less efficient in generating strong upper-tropospheric divergence or convergence.

The teleconnection pathways associated with TWP warming and La Niña also differ substantially. Constrained by the physical principles of stationary Rossby wave propagation, a strong La Niña can primarily excite a poleward- and eastward-propagating stationary Rossby wave train, so its generated teleconnection mainly influences the Pacific Basin and West Antarctica. In contrast, the TWP-related forcing is located farther west in comparison with ENSO forcing (Resp Fig. 4.1), and the associated Rossby wave train propagates toward the Indian Ocean sector of the Southern Ocean and East Antarctica. Furthermore, once the high-pressure anomaly is established over East Antarctica, it can be further maintained by active eddy–mean flow feedback in this region, in addition to the large-scale Rossby wave forcing.

We also clarify that although TWP warming and La Niña are not fully independent ($r = 0.41$, $p < 0.05$), prolonged TWP warming acts as the more direct tropical forcing of the East Antarctic dipole and associated positive precipitation anomalies. The AGCM sensitivity experiments support this interpretation. The TWP-only experiment successfully reproduces the dipole circulation and the positive precipitation response over East Antarctica (Resp Fig. 4.2). In contrast, the La Niña-like cooling-only and negative-IOD-like cooling-only experiments do not produce a significant East Antarctic dipole or a clear positive precipitation anomaly

over this region (Resp Fig. 4.3b,c). Moreover, the combined experiment with TWP warming, La Niña-like cooling, and IOD-like cooling yields a response slightly stronger than the TWP-only case (Resp Fig. 4.3a versus Resp Fig. 4.2). Together, these results indicate that TWP warming is a more direct driver in our framework, while ENSO and IOD likely contribute to the broader background state and may regulate the relative magnitude of SST anomalies in and around the TWP. Please see the revised manuscript at L277–282 and L351–366.

Line 59: Is it a positive or negative trend of 3±15 Gt/yr?

The value of 3 ± 15 Gt/yr refers to the consolidated estimate reported by Otosaka et al. (2023). Its small positive central estimate is not distinguishable from zero within the uncertainty range. We have revised "the EAIS has maintained a quasi-balanced state, with a mean trend of 3 ± 15 Gt/yr over the same period" to "the EAIS was quasi-balanced over the same period (3 ± 15 Gt/yr), though individual estimates diverge beyond their uncertainties" [L53–54]

Line 71: Was the anomalous precipitation primarily associated with Atmospheric River (AR)? How many AR events occurred, and would it worth to show a case study? Does global warming contribute to the intensity of the extreme precipitation associated with AR?

Thank you for this comment. We further examined the relationship between daily precipitation and AR activity over QW during the past 40 years and the 2021–2023 event. We added a new analysis showing that point-to-point daily precipitation and AR-frequency anomalies, after removing the seasonal cycle are positively correlated along much of the Antarctic coastal margin during 1979–2024, with correlations exceeding 0.4 near East Antarctica (365 × 46 = 16790 daily data points at each grid point, Resp Fig. 4.12). Over the QW sector from July 2021 to April 2023, both precipitation and AR anomalies are anomalously high (Resp Fig. 4.13), and daily QW-averaged AR and precipitation anomalies are strongly correlated ($r = 0.69$, $p < 0.01$; black solid line in Resp Fig. 4.14). Even after excluding the two outlier days with the most extreme ARs (red dots), the correlation remains high ($r = 0.67$). We also overlaid the AR anomalies during this period as contours in Fig. 1e. It is clear that increased precipitation over the QW region (green shading in Resp Fig. 4.13) coincides with enhanced AR activity (red contours in Resp Fig. 4.13) during the event. In addition, we examined the well-known March 2022 extreme East Antarctic AR event and found that it is accompanied by a sharp increase in both AR activity and precipitation anomalies over QW (Resp Fig. 4.15). These new results support the interpretation that the TWP-driven teleconnection helps establish a favorable large-scale condition for the intrusion of extreme AR-related snowfall into East Antarctica during the event period. Please see Fig. 1e and Extended Data Fig. 5e, f, as well as L226–229 and L304–307 in the revised manuscript.

Regarding the role of global warming, we examined the forced response derived from the CESM1 Large Ensemble and found that the observed ERA5 cumulative precipitation anomaly over QW during July 2021–April 2023 was roughly 11 times larger than the precipitation increase induced by the long-term warming trend alone in the CESM1 Large Ensemble (compare Resp Figs. 4.13, 4.16, and 4.17). This suggests that the SMB increase over QW during 2021–2023 is still primarily attributable to anomalous circulation and its role in steering AR activity and enhancing precipitation anomalies. Please see the revised manuscript at L156–161.

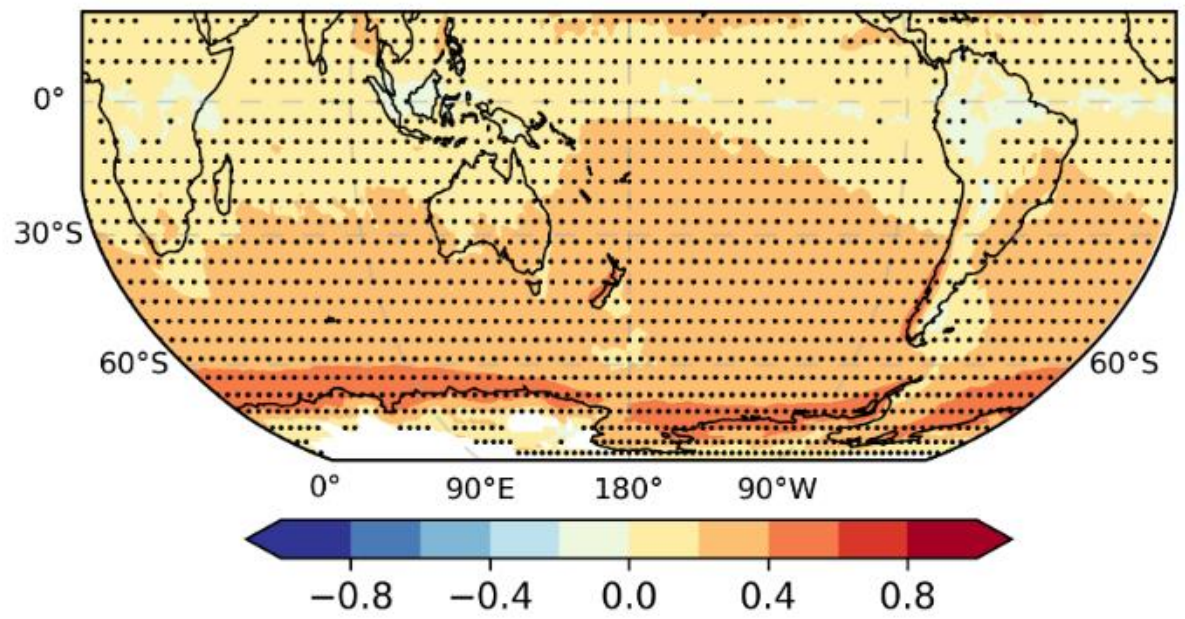

Resp Fig. 4.12. Correlation between daily precipitation anomalies and AR-frequency anomalies during 1979–2024. Daily precipitation is from ERA5, and AR frequency is based on the Guan and Waliser AR dataset. Both time series were deseasonalized and detrended before the analysis. Stippling indicates regions significant at the 95% level based on an FDR-corrected Student's t test.

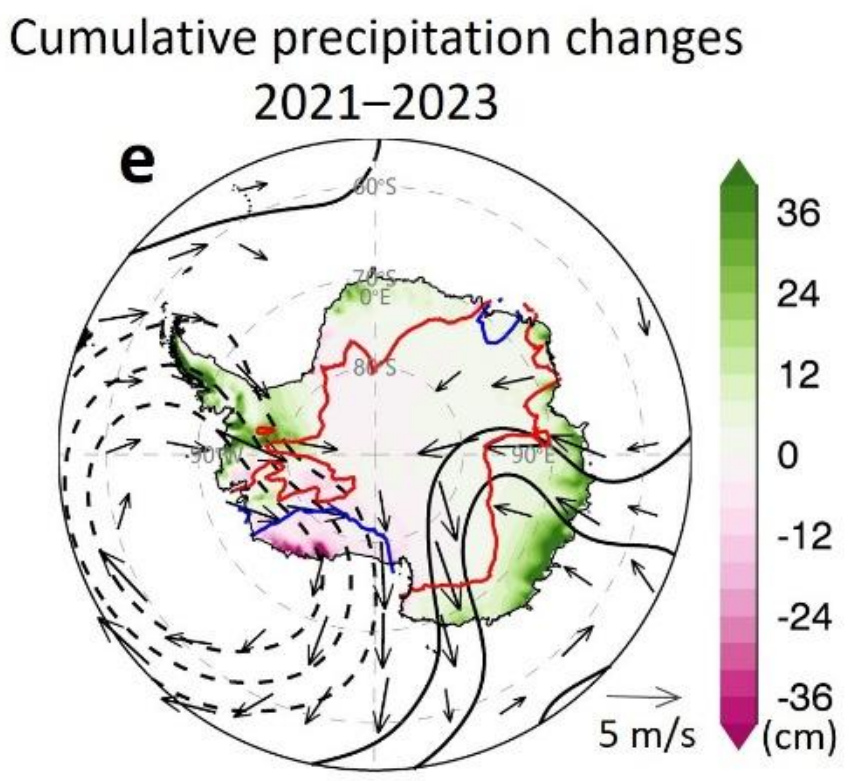

Resp Fig. 4.13 (also Fig. 1e in the main text). Cumulative Antarctic precipitation changes during July 2021–April 2023 (April 2023 minus July 2021), together with AR and circulation anomalies during the event. Shading shows cumulative ERA5 precipitation anomalies. Red and blue contours indicate positive and negative AR-frequency anomalies, respectively, at ±0.001 count day^{-1}. The unit count day^{-1} represents the daily AR occurrence frequency, defined as the number of AR detections per day normalized by the maximum possible daily count (ranging from 0 to 1). Black contours and vectors denote the mean Z300 and 300-hPa wind anomalies averaged over the 10 months during the event in which the area-weighted average QW precipitation exceeded the 80th percentile of the raw monthly distribution for 1979–2024.

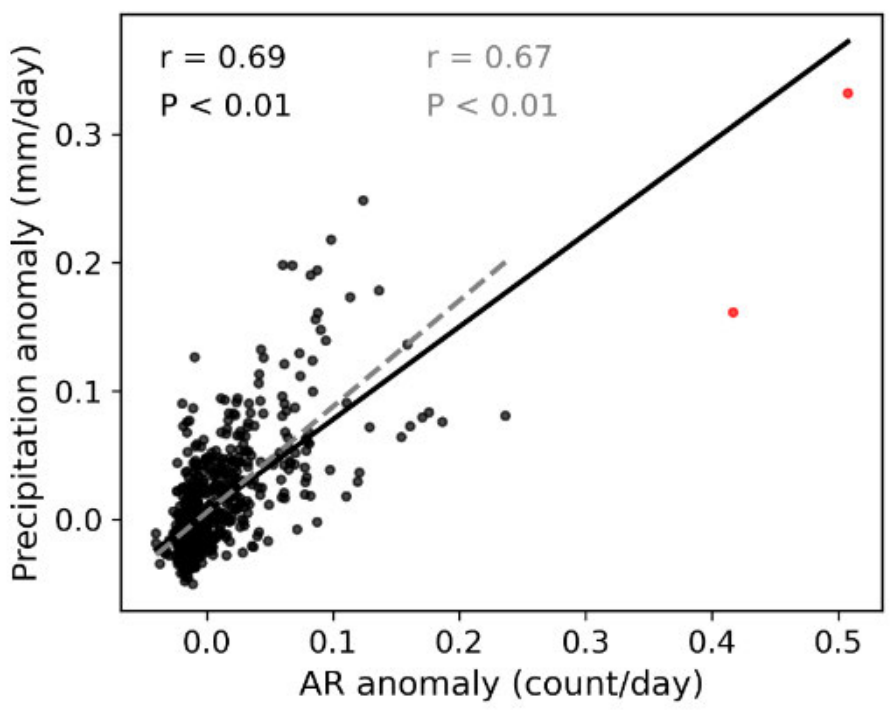

Resp Fig. 4.14. Scatterplot of daily precipitation anomalies versus AR-frequency anomalies, both averaged over the QW sector (70–145°E, 65–75°S), from July 2021 to April 2023. The solid black line shows the linear fit using all data points (r = 0.69). The dashed grey line shows the fit excluding the two days (red dots) with the most extreme ARs (r = 0.67). The unit count day^{-1} represents the daily AR occurrence frequency, defined as the number of AR detections per day normalized by the maximum possible daily count (ranging from 0 to 1).

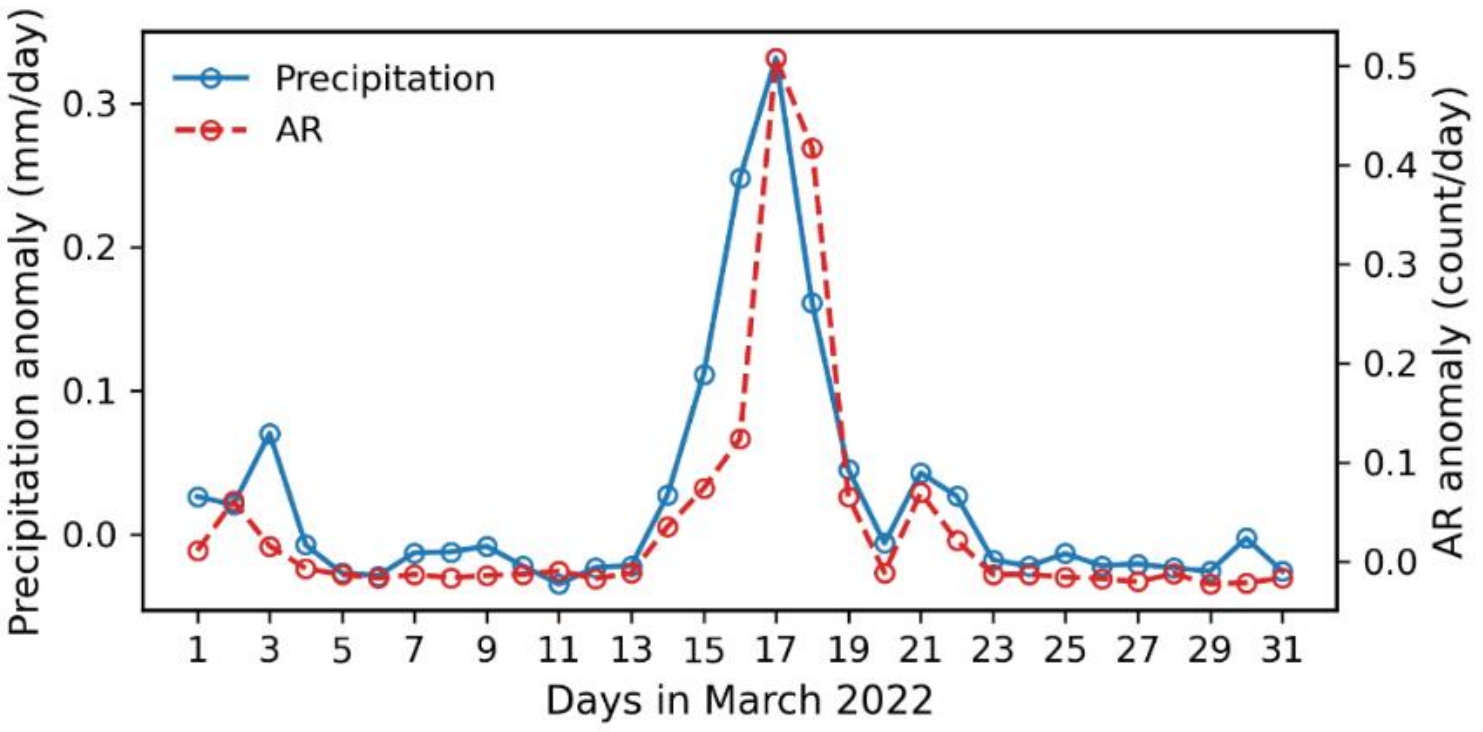

Resp Fig. 4.15. Daily time series of precipitation anomalies and AR-frequency anomalies, both averaged over the QW sector (70–145°E, 65–75°S), during March 2022. The figure shows that the March 2022 extreme East Antarctic AR event was accompanied by a sharp increase in both AR activity and precipitation over QW.

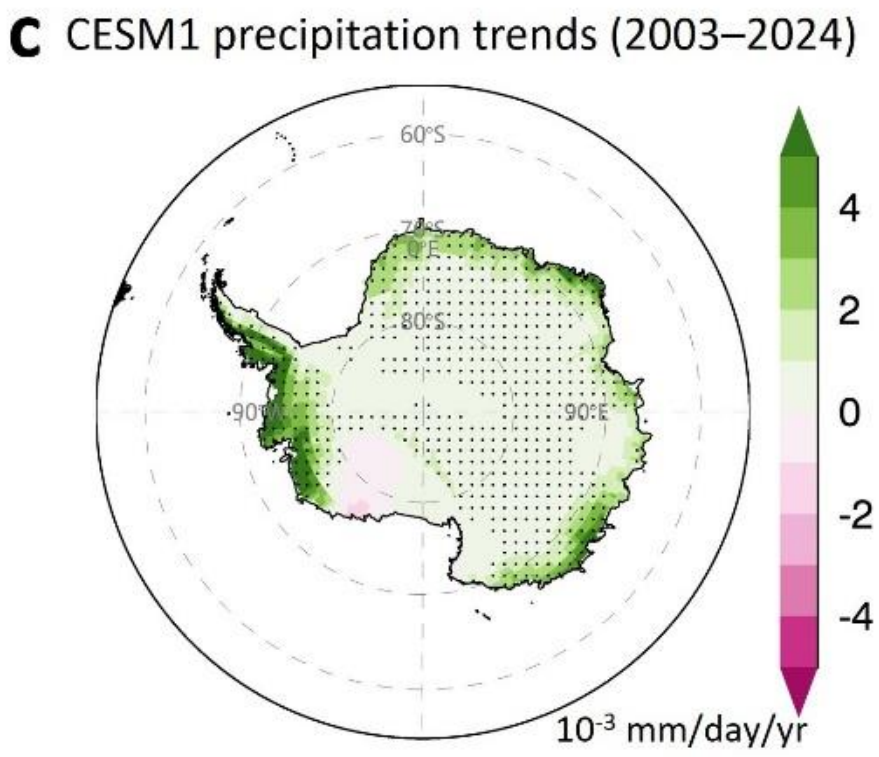

Resp Fig. 4.16 (also Extended Data Fig. 2c). CESM1 Antarctic precipitation trends for 2003–2024. Results represent the ensemble mean of 40 members. Stippling indicates regions significant at the 95% level based on an FDR-corrected Student's t test.

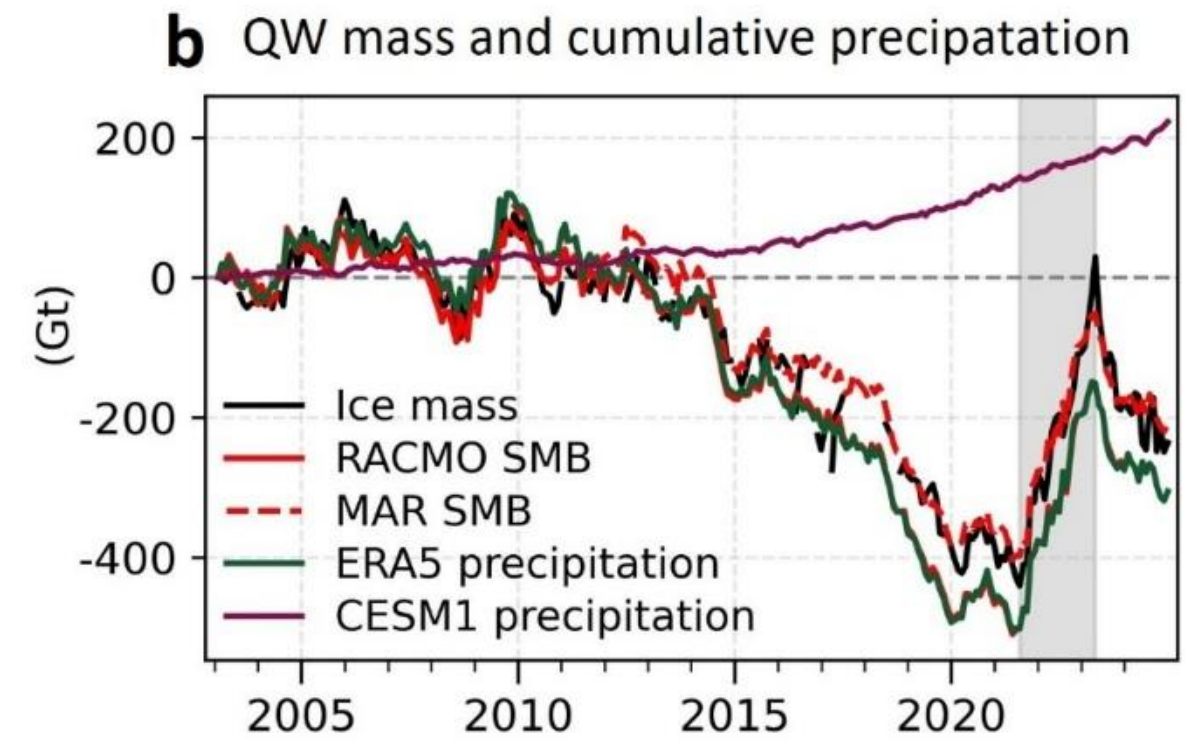

Resp Fig. 4.17 (also Fig. 1b). Mass change and associated precipitation anomalies in the QW sector (70–145°E, 65–75°S). Time series of deseasonalized ice mass changes (black line) from GRACE/GRACE-FO, compared with cumulative anomalies of surface mass balance (SMB; precipitation minus meltwater runoff, sublimation, evaporation, and wind erosion) from RACMO2.4 (solid red line) and MAR3.14 (dashed red line), precipitation from ERA5 (green line), and ensemble-mean precipitation from the 40-member CESM1 Large Ensemble (magenta line). All series are relative to January 2003. The grey shading marks the mass reversal period (July 2021–April 2023).

Line 72: Since Wang et al. (2025b) have linked this event to the three-year La Nina, is the mechanism reported in this manuscript against or complementary to their findings?

Thank you for this comment. Our mechanism does not contradict Wang et al. (2025b). Wang et al. (2025) interpreted the 2021–2023 Antarctic anomalies mainly in the context of the rare triple-dip La Niña. Their EOF/regression analyses emphasize a La Niña-related Ferrel Cell shift and a PSA-like circulation pattern, including a deepened Amundsen Sea Low. This pattern is associated with broader Antarctic surface-climate anomalies, including sea-ice loss and increased precipitation/SMB over parts of East Antarctica and the Antarctic Peninsula. However, it does not show the similar regional East Antarctic dipole identified in our study, which consists of a low-pressure anomaly south of Australia and a high-pressure anomaly over the East Antarctic coast. Therefore, their La Niña-related large-scale circulation response differs from the TWP-related dipole mechanism emphasized here. Thus, La Niña likely contributed to a tropical background cooling condition that may enhance the relative magnitude of SST warming in the TWP, while prolonged TWP warming provided the more direct tropical forcing for the formation of the regional East Antarctic dipole and the associated increase in QW precipitation. We have revised the manuscript to clarify this point [L279–281].

Line 131: '470.3 Gt': uncertainty estimates should be provided, and it should be compared with other potential data sources and the following analysis (e.g. Fig. 3c and Extended Data Fig. 12b).

We have added the uncertainty estimate for the GRACE/GRACE-FO mass change in the revised manuscript. The QW mass gain during July 2021–April 2023 is reported as 470.3 ± 29.1 Gt.
We have also clarified that this event-scale mass gain is consistent in magnitude with independent estimates of surface mass input over the same sector. During July 2021–April 2023, the cumulative changes over QW are 470.3 ± 29.1 Gt for GRACE/GRACE-FO ice mass, 348.3 ± 25.3 Gt for RACMO SMB, 346.3 ± 24.8 Gt for MAR SMB, and 351.2 ± 26.2 Gt for ERA5 precipitation (Resp Fig. 4.17). The uncertainty is estimated as one standard deviation of monthly values over the 22-month period. The small standard deviation, for example 29 Gt, relative to the much larger mean value of 470 Gt, indicates that the 22-month mean anomaly represents a persistent change throughout the period, rather than being dominated by a few months with extremely positive values. Although these values are not expected to agree exactly, because they represent different variables and are derived from different products, they consistently indicate a large positive anomaly over QW during the event. This supports our interpretation that the observed mass gain is physically consistent with the contemporaneous SMB and precipitation increase. Please see the revised manuscript at L107–109.

This estimate is also informative for gauging the simulated response in our AGCM experiment in a qualitative sense (Resp Fig. 4.2). The AGCM experiment does not attempt to reproduce the exact GRACE/GRACE-FO mass change directly, but it does reproduce a positive precipitation response over East Antarctica of about 0.3 mm day^{-1}, which is comparable to the observed event-scale precipitation anomaly. In Resp Fig. 4.13, the cumulative precipitation anomaly over the QW sector reaches about 36 cm during the event period, corresponding to an average anomaly on the order of 0.5 mm day^{-1}. Likewise, the historical composite associated with prolonged TWP warming (Resp Fig. 4.6) shows a consistent positive precipitation response over QW. We therefore use Resp Figs. 4.2 and 4.6 as proof of concept for the proposed mechanism, showing that the location, sign, and order of magnitude of the precipitation anomaly are

consistent with the observed event, rather than as a direct one-to-one quantitative estimate of the 470.3 ± 29.1 Gt mass gain. Please see the revised manuscript at L261–266.

Line 141: A smaller positive anomaly is also evident in these datasets around 2009 in Fig 1b. Is it also associated with MCA3 and TWP warming?

Thank you for this comment. We examined the 2009 anomaly patterns to assess whether the smaller positive episode was also related to the MCA3/TWP mechanism. The 2009 SST field shows weak positive anomalies over parts of the TWP, and the Z300 field shows a positive height anomaly over East Antarctica, with enhanced precipitation over parts of the QW sector (Resp Fig. 4.18). These features are consistent, to some extent, with the positive phase of the MCA3/TWP relationship. In particular, during this year, the eastern Pacific exhibited an El Niño-like pattern, indicating that the teleconnection between TWP warming and increased QW precipitation does not always covary with a La Niña-like pattern. However, we emphasize that our proposed mechanism represents one leading mode that helps explain QW precipitation variability among other influences. We do not intend to attribute all QW precipitation increases to TWP variability, as QW precipitation can be influenced by multiple processes operating across different time scales.

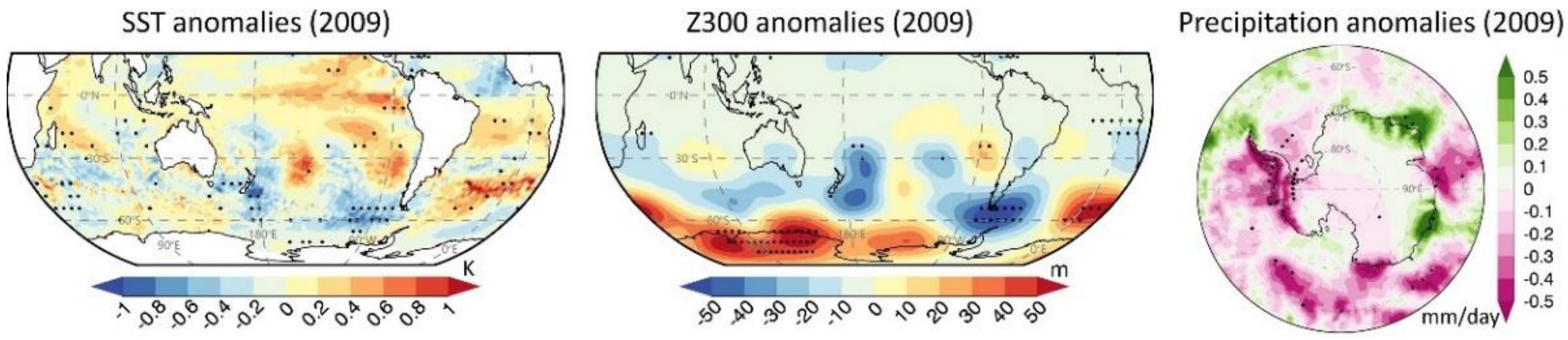

Resp Fig. 4.18. Annual-mean anomalies of ERA5 SST, Z300, and precipitation in 2009. The anomaly patterns are computed after removing the climatological seasonal cycle and linear trend over 1979–2024. Stippling indicates regions significant at the 95% level based on an FDR-corrected Student's t test.

Line 154: Fig. 1a, dashed red line showing MAR SMB is hardly distinguishable from the solid red line showing RACMO SMB. Why is there missing data in Ice mass from GRACE/GRACE-FO? Fig. 1b, QW mass rapidly declines after the peak in April 2023, why is that? Fig. 1e, does the Amundsen Sea Low have a role in maintaining and strengthening the high pressure ridge?

Regarding the MAR and RACMO SMB curves in Fig. 1a, we agree that the dashed MAR SMB line is difficult to distinguish from the solid RACMO SMB line because the two products show very similar cumulative SMB changes. The close agreement between MAR and RACMO provides independent support that the Antarctic-wide cumulative SMB anomaly is robust across regional climate model products. The missing values in the GRACE/GRACE-FO ice-mass record reflect data gaps, due to technical issues in the satellite gravimetry observations, rather than data processing choices. These include the gap between the end of the original GRACE mission and the start of GRACE-FO observations, as well as shorter periodic data gaps during the later GRACE period related to battery-management operations. We have clarified this in the caption of Fig. 1 of the revised manuscript [L136].

The rapid QW mass decline after the April 2023 peak may be caused by a transition from the prolonged positive precipitation anomaly during July 2021–April 2023 to negative precipitation anomalies afterward. To examine this change, we calculated the mean SST, Z300, and precipitation anomalies for May 2023–June 2024, when QW ice mass decreased rapidly. The results show a clear cooling anomaly over the TWP region,

accompanied by a negative phase of the East Antarctic dipole, with a low-pressure anomaly over East Antarctica (Resp Fig. 4.19). Consistent with this phase reversal, precipitation over the QW sector became negative.

Regarding the Amundsen Sea Low (ASL), we suggest that the ASL and the East Antarctic high-pressure anomaly in Fig. 1e should not be interpreted as one integrated circulation system. The ASL is mainly associated with La Niña-like Pacific forcing, whereas the East Antarctic high-pressure anomaly is linked to the TWP-forced Rossby wave train. This distinction is consistent with the expected pathway of stationary Rossby wave propagation: La Niña-related forcing tends to generate a wave train that more directly affects the Pacific sector and West Antarctica, whereas TWP forcing is more effective in producing the East Antarctic dipole emphasized in our study. The TWP-forced wave train can also produce a downstream low-pressure anomaly on the Pacific side of Antarctica if the dipole pattern over East Antarctica propagates farther downstream. However, because this low-pressure anomaly lies downstream of the East Antarctic high pressure anomaly, as evidenced by the clear wave activity shown in Resp Fig. 4.2, we do not interpret the ASL as the main forcing factor triggering or strengthening the upstream East Antarctic high-pressure anomaly.

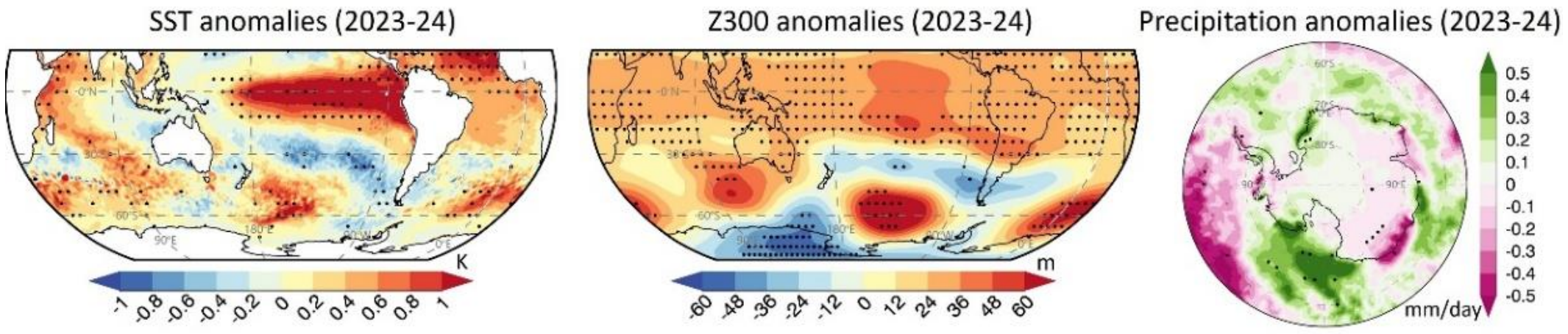

Resp Fig. 4.19. Mean ERA5 SST, Z300, and precipitation anomalies during May 2023 to June 2024. The anomaly patterns are computed after removing the climatological seasonal cycle and linear trend over 1979–2024. Stippling indicates regions significant at the 95% level based on an FDR-corrected Student's t test.

Line 165: Standard Deviation assumes normal distribution, but precipitation normally follows log-normal or gamma distribution? Maybe another approach such as a threshold as used in the literature makes more sense?

Thank you for this helpful suggestion. We revised the analysis using the 80th percentile of the area-weighted average QW precipitation for 1979–2024 as the threshold. During July 2021–April 2023, 10 of the 22 months exceeded this threshold [please see L141–142]. The occurrence of such extreme precipitation months in QW during the event was about 2.3 times the average occurrence of similar events. Specifically, the event frequency was 10/22 = 0.45, whereas the expected average occurrence is 1/5 = 0.2, because the threshold is defined by the 80th percentile.

Line 172: The underlying assumption for this whole paragraph is that global warming resulted from anthropogenic forcings manifest in a uniform magnitude, which can be captured by CESM1. I am not fully convinced, as it has been widely understood large regional variability exists in response to global warming. I am wondering how much of this precipitation anomaly could be attributed to the fact that a warmer atmosphere could hold more moisture based on the Clausius–Clapeyron relation, i.e. a thermodynamic response, and how much can be decomposed to the dynamic factor. Could the authors attribute this dynamic

factor to natural variability or (direct or indirect) anthropogenic forcings, or both (as it was said to be a key question this manuscript is investigating)?

We agree that anthropogenic warming does not produce a spatially uniform response and that Antarctic precipitation changes include both thermodynamic and dynamic components. We used the CESM1 Large Ensemble mean only as a first-order estimate of the externally forced response, because ensemble averaging largely suppresses internal variability and highlights the forced signal. The CESM1 ensemble-mean forced precipitation trend over QW is much smaller than the observed 2021–2023 anomaly (Resp Fig. 4.16). Based on the CESM1 ensemble-mean cumulative precipitation series (Resp Fig. 4.17), the forced precipitation increase over QW from July 2021 to April 2023 is only 32.3 ± 2.4 Gt, corresponding to about 9% of the observed ERA5 cumulative precipitation anomaly over the same region and period (351.2 ± 26.2 Gt). This comparison suggests that forced thermodynamic moistening may have contributed to the background precipitation increase, but cannot explain the majority of the event magnitude. We therefore interpret the 2021–2023 event as being dominated by the dynamic component induced by the dipole circulation pattern. This dynamic component is closely linked to the TWP-related teleconnection identified in our MCA and AGCM experiments. Please see the revised manuscript at L156–161.

We do not rule out a role of anthropogenic forcing in modulating similar events in the future. Global warming may influence the precipitation impact of future TWP-driven events through more complex pathways, including changes in high-latitude atmospheric moisture, AR intensity, storm tracks, precipitation phase, and ocean–atmosphere feedbacks. These processes require a more comprehensive coupled-model analysis and are beyond the scope of the present study. We therefore treat this issue as an open question in the revised manuscript.

Line 220: I am curious about the relationship between MCA3 and other common modes such as the Southern Annular Mode and the Zonal Wave-3 index (Goyal et al., 2022). Fig. 2e, why do QW mass and Cumulative standardized MCA3 precipitation diverge before ~2009? Do I miss a time series of MCA3 or why does the positive Standardized MCA3 Z300 before 2020 in Fig. 2c not result in large precipitation anomalies in Fig. 2d?

We would also like to elaborate on the relationship between SAM and our mode. SAM is usually described as a hemispheric annular mode associated with meridional shifts of the Southern Hemisphere westerly jet, rather than a localized tropical-forced dipole over the south-of-Australia/East Antarctica sector. On the other hand, we also agree that our mode may contain some SAM-like regional signal. Consistent with this, the MCA3 Z300 time series is significantly correlated with the SAM index ($r = -0.31$, $p < 0.01$). This relationship is also consistent with Ding et al. (2012), who showed that SAM has strong zonal asymmetry: in the Pacific sector, it resembles a tropically forced PSA-like wave train, while in the Indian Ocean sector, it includes a meridional dipole and is maintained by eddy–mean flow feedback. In that study, the dipole structure near East Antarctica was suggested to be internally driven. This is also consistent with our finding that synoptic eddy forcing helps maintain the dipole pattern once it is established. In our study, we make a significant advance by showing that this dipole structure can also be tropically driven by TWP forcing, which is one of the main contributions and novel aspects of our study. However, because the TWP–SAM correlation is near zero and the spatial pattern in our study is much more regional, our MCA3 mode is dynamically distinct from the canonical SAM pattern, although it may project onto the Indian Ocean component of the SAM. We also compared the MCA3 circulation pattern with the ZW3 spatial patterns

(Resp Fig. 4.20, adapted from Goyal et al., 2022, Fig. 2a–c). The two modes are fundamentally different. ZW3 is a zonally distributed wave-3 pattern around the Southern Hemisphere, whereas MCA3 is a regional meridional dipole over the Indian Ocean–East Antarctica sector, with an East Antarctic high-pressure anomaly and an equatorward low-pressure anomaly south of Australia.

The divergence of the two curves before about 2009 in Fig. 2e (Resp Fig. 4.21a) mainly reflects the cumulative nature of the MCA3 precipitation index. For example, the MCA3 precipitation index has a relatively strong positive anomaly around 2009, and once accumulated, this anomaly produces a persistent offset in the cumulative curve. To illustrate this effect, we performed a simple sensitivity and visualization test in Resp Fig. 4.21b. We set the MCA3 precipitation anomalies during June 2008–October 2009 to zero before calculating the cumulative sum, and adjusted the right-axis range for QW mass only to facilitate visual comparison with the green curve. After this adjustment, the cumulative MCA3 precipitation series aligns much better with the QW mass series. This does not imply that the 2008–2009 anomaly is the only cause of the divergence. It simply illustrates that small differences between the two original time series can accumulate over time and appear as a bifurcation in the cumulative curves. The two curves are also not expected to match exactly because the standardized MCA3 time series is a precipitation-mode index reflecting a typical precipitation change across the entire continent, whereas GRACE/GRACE-FO measures total mass change over the QW sector. We use Fig. 2e to show consistency in variability, especially the 2021–2023 increase, rather than a one-to-one reconstruction of QW mass. We emphasized this point in the Fig. 2e caption [L209].

Regarding Fig. 2c–d, Z300 represents the circulation component of the MCA mode, whereas precipitation represents the associated hydrological response. The two time series are linked through the MCA, but they are not expected to match month-by-month. Before 2020, the MCA3 Z300 index alternates frequently between positive and negative phases, so short positive Z300 episodes do not produce sustained precipitation anomalies. A clearer precipitation response occurs when the positive circulation phase is more persistent, as around 2000–2002 and especially during July 2021–April 2023.

Ding, Q., Steig, E. J., Battisti, D. S. & Wallace, J. M. Influence of the Tropics on the Southern Annular Mode. J. Clim. 25, 6330-6348 (2012).
Goyal, R., Jucker, M., Gupta, A. S. & England, M. H. A New Zonal Wave-3 Index for the Southern Hemisphere. J. Clim. 35, 5137-5149 (2022).

[Redaction]
Figure Includes
Third Party Material

Resp Fig. 4.20. Spatial patterns of the ZW3 mode adapted from Fig. 2a–c of Goyal et al. (2022). ZW3 mode is derived from EOF analysis of ERA5 monthly 500-hPa meridional wind anomalies over 1979–2020. ***a–c***, *The first three EOF modes, respectively. The values in brackets indicate the percentage of total variance*

explained by each EOF mode. The ZW3 index was defined using the first two principal components: its magnitude was calculated as the square root of the sum of the squared PC1 and PC2 values, and its phase was calculated from the arctangent of the ratio of PC2 to PC1.

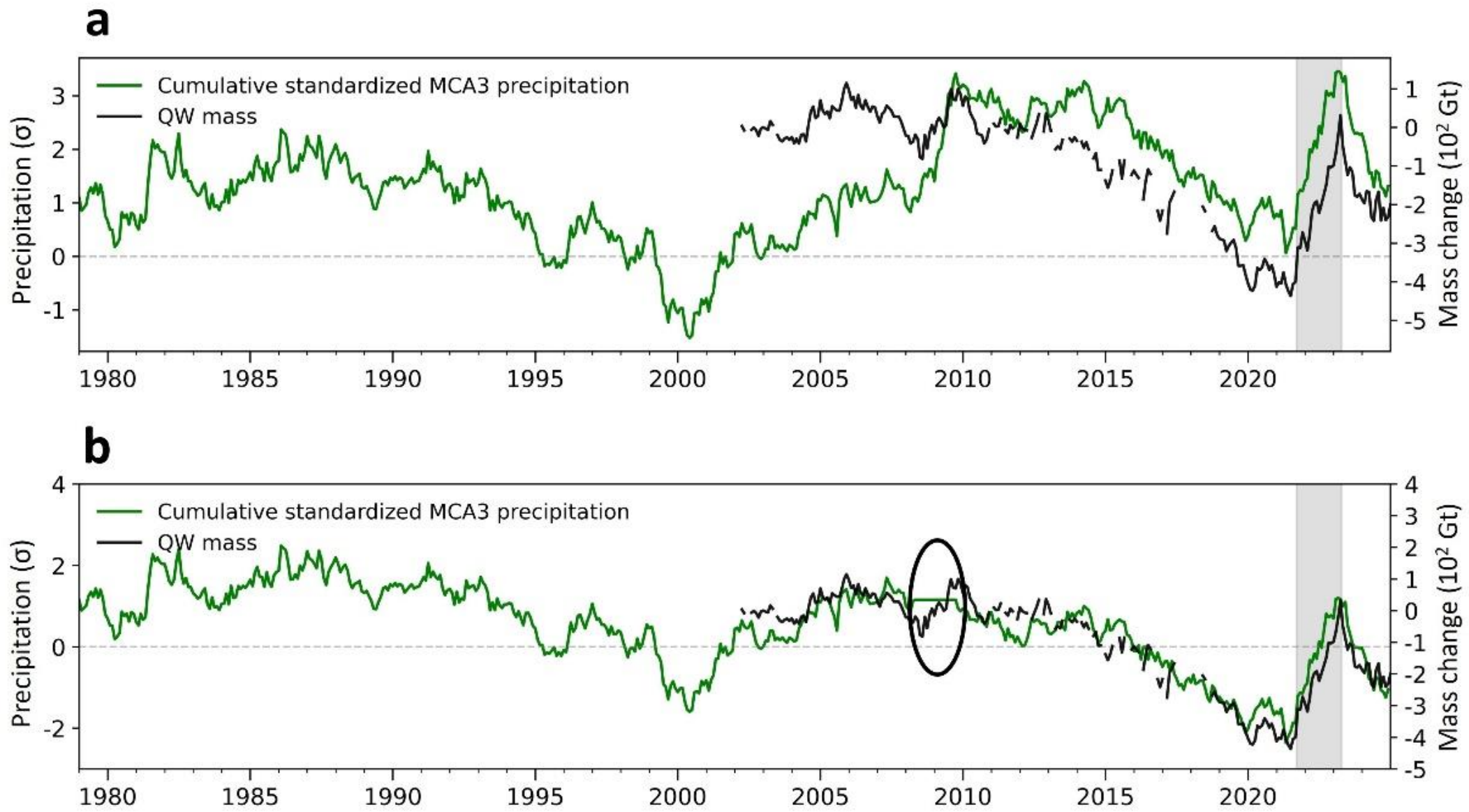

Resp Fig. 4.21. Comparison of precipitation and mass change time series. ***a*** *(also Fig. 2e in the main text), Comparison between the cumulative standardized MCA3 precipitation time series (green) and the QW mass change time series (black).* ***b****, Same as in a, but with the monthly MCA3 precipitation anomalies from June 2008 to October 2009 set to zero before calculating the cumulative sum. The right-axis range in b is adjusted only to facilitate visual comparison with the green curve. Gray shading marks the July 2021–April 2023 event period. This test illustrates that a short-period anomaly can produce a persistent offset in a cumulative curve.*

Line 232: Again, the warming in Extended Data Fig. 5c is more significant and pronounced over SPCZ rather than TWP. If the authors argue it is the TWP warming that affects MCA3, why is MCA3 also linked to cooling over the Indian Ocean and eastern Pacific? Could you conduct a partial correlation analysis between MCA3 and SST, while controlling IOD, La Nina, etc.?

Following your suggestion, we conducted a partial correlation analysis between the MCA3 Z300 index and global SST anomalies after removing the linear influence of ENSO and IOD. This procedure weakens the negative correlations over the Indian Ocean and eastern Pacific (Resp Fig. 4.22), indicating that some of cooling signals are associated with ENSO/IOD-related covariability. The positive SST correlations over the western Pacific/TWP–SPCZ sector also become weaker and less spatially coherent than in the original correlation map, although significant correlations remain over the eastern part of the TWP. However, partial linear correlation analysis not only removes the linear component associated with these selected indices, but also removes some direct impacts related to TWP SST forcing itself. Even so, we still find a clear connection with the TWP-residual SST pattern, which is similar to the mode identified in this study, giving us more confidence about the independence of the TWP-EA teleconnection from other climate drivers.

As discussed in our response to Major Comment 1, the TWP–EA pathway differs from the ENSO/IPO–ASL pathway in both the forcing location and the downstream Antarctic response. In our response to your minor comments labeled Line 36 in your review, we further tested the role of SPCZ-related SST forcing and

showed that the SPCZ-only experiment does not reproduce the observed East Antarctic dipole. In our response to your minor comments labeled Line 41 in your review, we clarified that TWP warming and La Niña are not fully independent, but that the AGCM experiments provide a more direct dynamical test of their relative roles. Specifically, the TWP-only experiment reproduces the East Antarctic dipole circulation and the positive precipitation response over the QW sector, whereas the La Niña-like cooling-only, IOD-like cooling-only, and SPCZ warming-only experiments do not produce a similar East Antarctic dipole or significant positive precipitation response. We have clarified this point in the revised manuscript [L277–282].

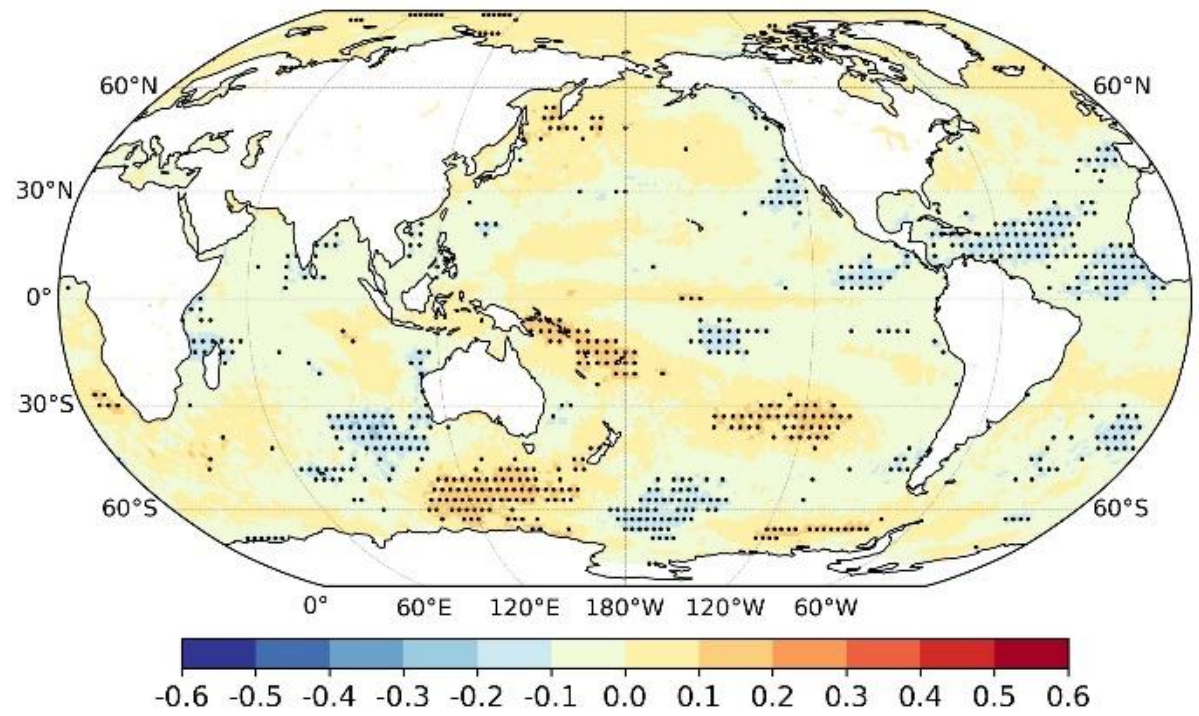

Resp Fig. 4.22. Partial correlation between the MCA3 Z300 index and global SST anomalies after removing ENSO and IOD influences. All time series were deseasonalized and detrended before the analysis. Stippling indicates regions significant at the 95% level based on an FDR-corrected Student's t test.

Line 237: 'a recurrent physically robust teleconnection mode': MCA3 is dependent on precipitation. To be robust, I would expect more rigorous analysis between SST over TWP/SPCZ and circulation dipole near EA, without dependence on Antarctic precipitation and taking into account the impact of other tropical and SH modes.

Thank you for the comment. We added an independent circulation-based analysis that does not use Antarctic precipitation. Specifically, we constructed an East Antarctic dipole index using only Z300 anomalies, defined as the area-mean Z300 anomaly over the East Antarctic high-pressure center (120°–160°E, 70°–55°S) minus that over the low-pressure center south of Australia (80°–150°E, 50°–30°S). We then correlated this Z300-only dipole index with global SST anomalies. The direct correlation shows a tropical SST pattern similar to that associated with MCA3, including positive SST correlations over the TWP sector and negative correlations over the Indian Ocean and eastern Pacific (Resp Fig. 4.23a). This supports the direct link between the East Antarctic circulation dipole and tropical SST variability. We further repeated this analysis using partial correlation analysis after removing the linear influences of ENSO, IOD, SAM, and IPO. After this step, the SST correlations become much weaker and less spatially coherent, especially over the Indian Ocean and eastern Pacific (Resp Fig. 4.23c). This indicates that part of the original SST pattern reflects covariability with established tropical and SH modes. As mentioned above, partial linear correlation analysis not only removes the linear component associated with these selected indices, but may also remove part of the direct impact of TWP SST forcing itself. However, weak positive correlations remain over parts of the TWP sector, suggesting that the circulation dipole still contains a residual relationship with western Pacific SST variability even after these modes are linearly removed.

We also performed a complementary analysis by correlating the TWP SST index with global Z300 anomalies. The direct correlation shows a clear wave-like Z300 pattern extending from the tropics into the

Southern Hemisphere extratropics, including circulation anomalies near the East Antarctic sector (Resp Fig. 4.23b). The pattern also shows a Gill-like tropical response over the TWP, together with opposite tropical height anomalies associated with La Niña-like, IOD-like, and tropical Atlantic SST signals. These broader tropical anomalies make the tropical height field more negative across other basins, while the Gill-type response to TWP warming acts as a key forcing agent that triggers the teleconnection toward East Antarctica. After removing the linear influences of ENSO, IOD, and SAM, the tropical Z300 pattern becomes more spatially uniform (Resp Fig. 4.23d). This is consistent with our physical understanding that tropical waves generated by TWP forcing can quickly propagate across the entire tropical basin if the influences of other tropical modes are removed. When other modes are not removed, modes with the opposite sign may also generate Gill-type patterns in the opposite phase, which may mute the Gill-type response generated by TWP warming (Resp Fig. 4.23b). In the extratropics, the positive Z300 anomaly over East Antarctica remains evident when the impacts of all other modes are removed (Resp Fig. 4.23d), indicating that the TWP–East Antarctic circulation linkage is independent from other climate modes to some extent. We have added the direct Z300-only dipole and TWP-index correlation analyses to Extended Data Fig. 6. Please see the revised manuscript at L217–219.

Importantly, partial linear correlations not only remove the linear component associated with these selected indices, but may also remove part of the direct impact of TWP SST forcing itself. Therefore, we use the AGCM sensitivity experiments as the main dynamical evidence for the proposed mechanism. As discussed in our response to your earlier comment labeled Line 232, the TWP-only experiment reproduces the East Antarctic dipole and QW precipitation response, whereas the La Niña-like cooling-only, IOD-like cooling-only, and SPCZ-only experiments do not produce a comparable response.

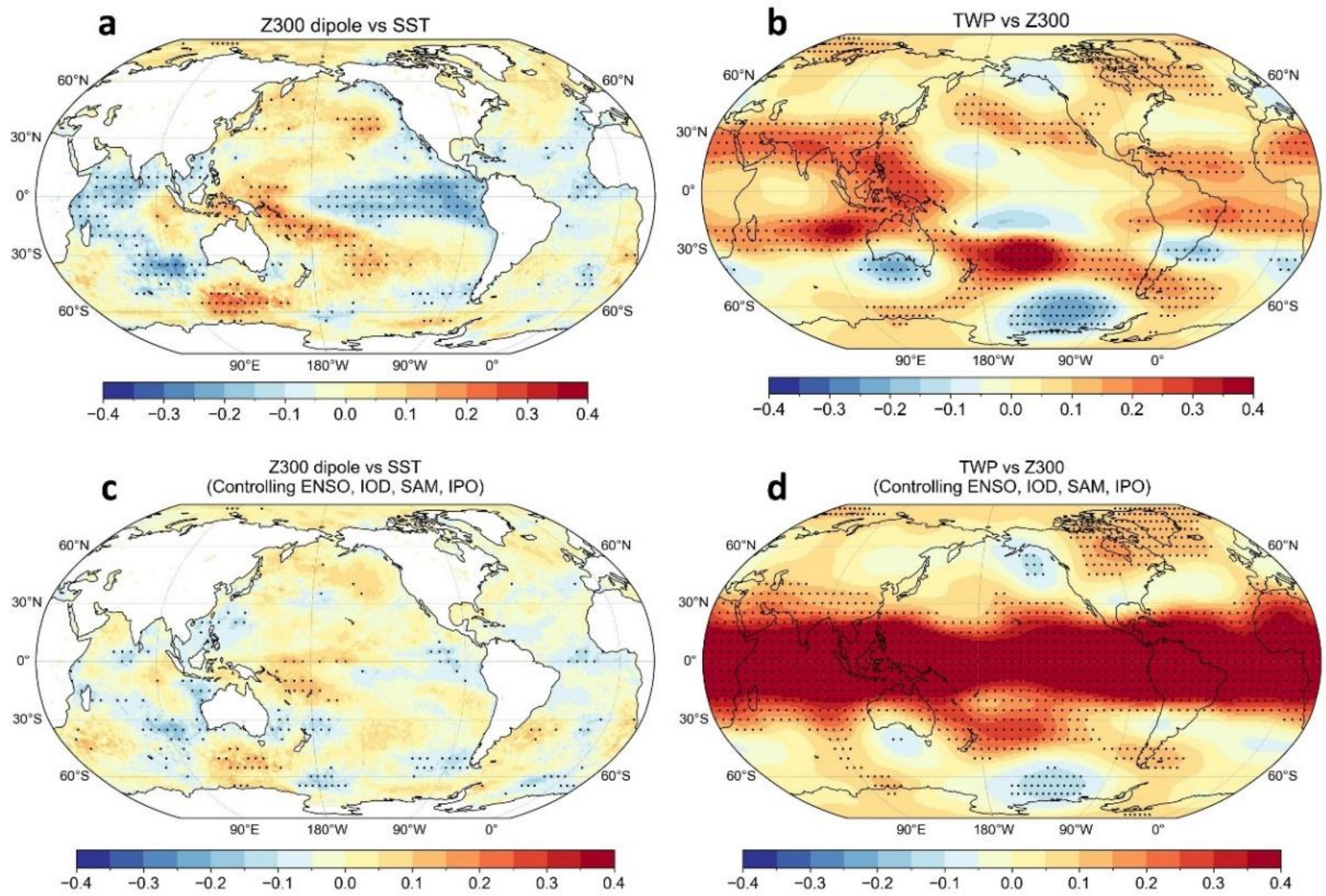

Resp Fig. 4.23. Relationships between the East Antarctic circulation dipole, TWP SST variability, and large-scale climate modes. ***a****, Correlation between the Z300-based East Antarctic dipole index and global SST anomalies. The dipole index is defined as the area-mean Z300 anomaly over the East Antarctic high-pressure center (55°–70°S, 120°–160°E) minus that over the low-pressure center south of Australia (30°–*

50°S, 80°–150°E). ***b****, Correlation between the TWP SST index and global Z300 anomalies.* ***c****, As in a, but after removing the linear influences of ENSO, IOD, SAM, and IPO.* ***d****, As in b, but after removing the linear influences of ENSO, IOD, SAM, and IPO. All variables were deseasonalized and detrended before the analysis. Stippling indicates regions significant at the 95% level.*

Line 239: Extended Data Fig. 6c, the Standardized TWP SST is even more positive around 2000. If the hypothesis of the authors is correct, should it induce even stronger precipitation anomalies?

Yes, the strong positive TWP SST index around 2000 corresponds to another jump in QW precipitation that we discuss in the manuscript. During 2000–2002, the cumulative MCA3 precipitation index increased, and the associated SST and Z300 anomaly patterns show a similar, but weaker, teleconnection structure to that during 2021–2023 (Resp Fig. 4.24 and 4.25). Therefore, the 2000–2002 period is consistent with our hypothesis, although the precipitation response was weaker and less persistent than the 2021–2023 event.

We do not suggest a simple one-to-one relationship in which a larger TWP SST index must always produce a proportionally larger QW precipitation anomaly. Prolonged TWP warming increases the likelihood of establishing the East Antarctic dipole, enhanced poleward moisture transport, and increased AR activity toward QW, but the ultimate precipitation response also depends on many coexisting factors and processes, including the Southern Hemisphere circulation variation in the background and synoptic moisture-transport processes. In particular, the positive phase around 1999 followed a strong negative phase, which was associated with an opposite tropical–high-latitude circulation configuration. This preceding circulation state may have made the East Antarctic dipole less efficiently coupled to moisture transport than during 2021–2023, leading to a weaker QW precipitation response despite the strong TWP SST forcing.

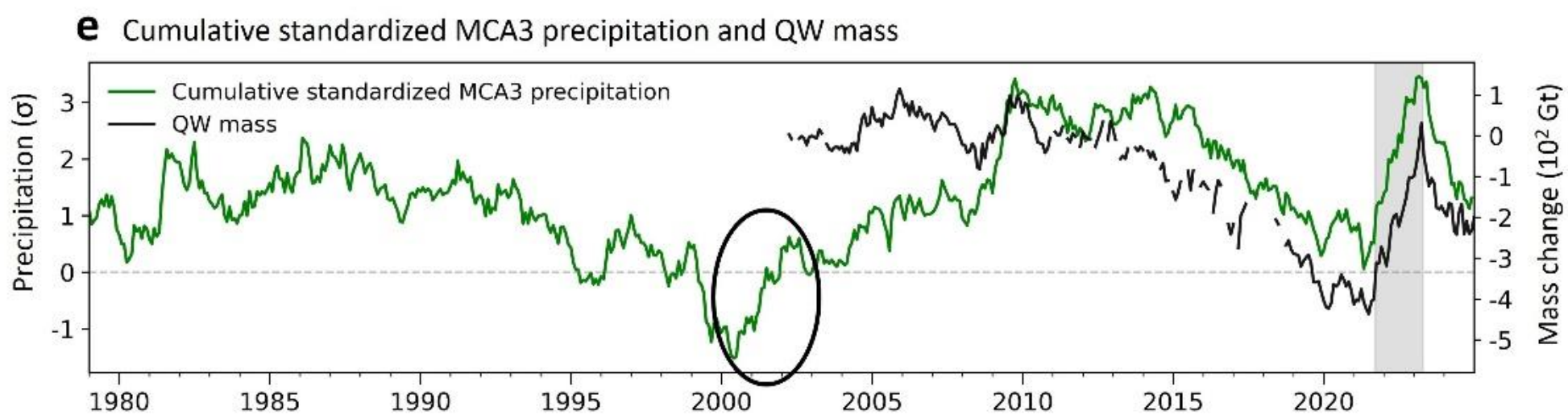

Resp Fig. 4.24 (also Fig. 2e in the main text). Comparison between the cumulative standardized MCA3 precipitation time series (green) and the QW mass change time series (black).

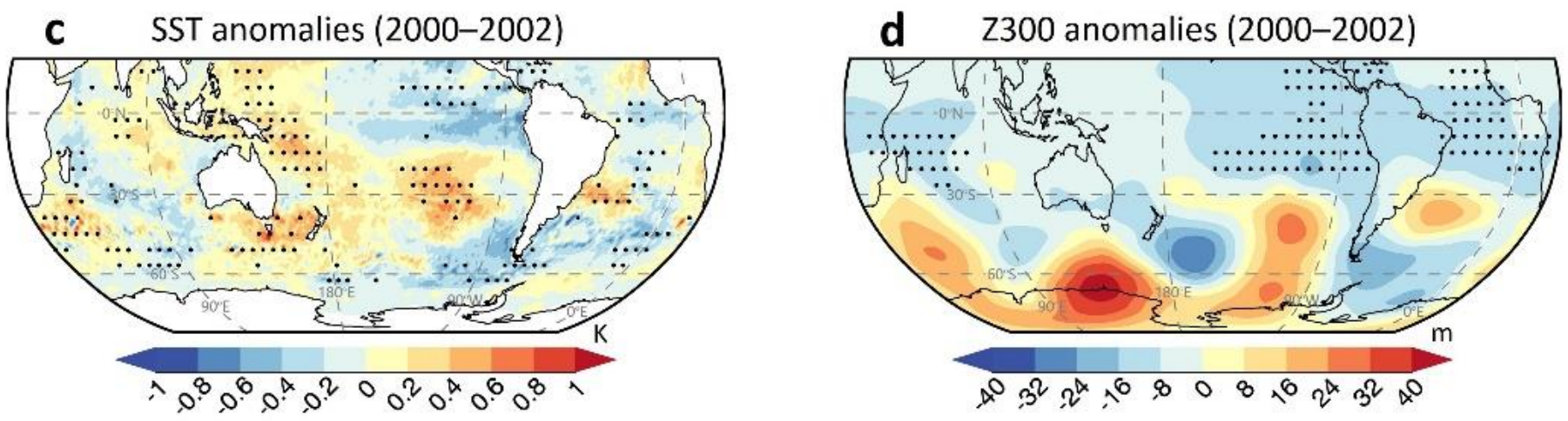

Resp Fig. 4.25 (also Extended Data Fig. 10c,d). Anomalies of SST and Z300 from ERA5 for the period from August 2000 to June 2002. ***c****, Mean SST anomalies.* ***d****, Mean Z300 anomalies. Stippling indicates regions significant at the 95% level based on an FDR-corrected Student's t test.*

Line 252: 'eddy-mean flow feedback': It is far beyond my expertise, but I consider it extremely interesting about potential positive feedbacks that maintain the dipole circulation patterns. Without referring to the references, it is very hard to follow the arguments of the authors about the slow down of westerly jet and its

consequences. Is it a hypothesis? Do you have more evidences in addition to the correlation patterns between Synoptic Eddy and MCA3 Z300 in Extended Data Fig. 7? As I asked before, are there temporal variations in TWP warming and the Rossby wave train during this event?

Our main evidence for the maintenance of the East Antarctic dipole comes from three parts. The WAF diagnosis shows Rossby wave activity propagating from the TWP toward high southern latitudes, supporting the role of remote tropical forcing in initiating the dipole circulation. The AGCM experiments show that TWP-only forcing can reproduce the low-pressure anomaly south of Australia and the downstream high-pressure anomaly over East Antarctica, whereas La Niña-like cooling-only and IOD-like cooling-only experiments do not produce a comparable response. The synoptic-eddy vorticity tendency is positively correlated with the MCA3 Z300 index near East Antarctica, suggesting that local eddy forcing is in phase with the dipole anomaly and helps reinforce it. This is a convenient approach, similar to those commonly used to demonstrate the role of eddy-mean flow feedback. If this mechanism is not important near East Antarctica, we would expect to see a noisy correlation pattern, similar to the very weak relationships between eddy forcing and the MCA3 Z300 time series in other regions.

Our interpretation is also consistent with Ding et al. (2012), which showed that the East Antarctic dipole can be driven by eddy–mean flow feedback, but did not link this dipole to tropical forcing. The novel contribution of our study is to show that this dipole can also be initiated by TWP forcing and then maintained locally by eddy-mean flow feedbacks. We have cited Ding et al. 2012 at L232 and L237 in the revised manuscript, where we discuss the role of this feedback in strengthening the dipole structure near EA.

Physically, we would like to elaborate on this process a bit more here. As we show in the manuscript, the EA dipole structure favors a weakening of the jet in the region between the two pressure cells. This weakened jet can reduce eddy activity and limit the eastward propagation of transient eddies over this region. As a result, the associated eddy vorticity forcing also changes. In the climatological mean state, transient eddies provide substantial vorticity forcing over EA. When slightly fewer eddies reach this region along the jet because of the weakened jet, a tendency toward higher pressure (anticyclonic tendency) is expected there. At the same time, more eddies may remain on the lower-latitude side of the jet, enhancing lower pressure (cyclonic tendency) in that region. This scenario is the classical idea of eddy-mean flow feedback, and our correlation pattern (MCA3 mode with eddy forcing) is consistent with this physical interpretation and also with previous literature.

We also compared the TWP SST index with a newly defined Z300-only dipole index (Resp Fig. 4.26). This index does not use Antarctic precipitation; it is defined as the area-mean Z300 anomaly over the East Antarctic high-pressure center minus that over the low-pressure center south of Australia. Both the TWP SST index and the Z300 dipole index show persistent positive anomalies during July 2021–April 2023, supporting the temporal co-evolution between prolonged TWP warming and the East Antarctic dipole during the event. We also note that the Z300 dipole index shows strong month-to-month variability, indicating that the East Antarctic high-latitude circulation is less persistent from month-to-month. We therefore interpret prolonged TWP warming as a persistent low-frequency forcing that favors the establishment of the East Antarctic dipole, while month-to-month circulation variations are also actively modulated by synoptic variability and local eddy–mean flow feedbacks. These processes can imprint strong short-term changes on top of the persistent dipole tendency driven by remote TWP forcing.

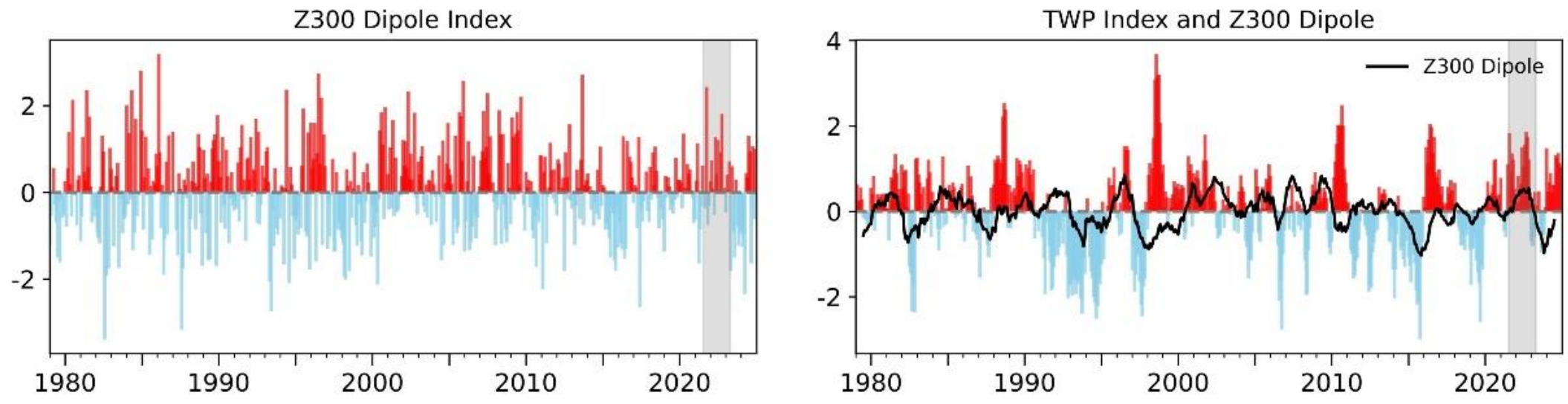

Resp Fig. 4.26. Temporal evolution of the East Antarctic Z300 dipole index and the TWP SST index from 1979 to 2024. The dipole index is defined as the area-averaged Z300 anomaly over the East Antarctic high-pressure center (55°–70°S, 120°–160°E) minus that over the low-pressure center south of Australia (30°–50°S, 80°–150°E). Red and blue bars indicate positive and negative anomalies, respectively. The black line in the right panel represents the 12-month running mean of the Z300 dipole index. The grey shading denotes the July 2021–April 2023 event period.

Line 264: The authors argue TWP warming recurs every ~10 years, whereas the high-latitude dipole circulation is more like an internal model of circulation patterns. Does the dipole circulation or MCA3 positive phase also recurs every 10 years following TWP warming?

Prolonged TWP warming events recur approximately once per decade, and the composite analysis shows that these events are statistically accompanied by a similar high-latitude dipole circulation and positive precipitation anomalies over QW. Using ERA5 over 1950–2025, we detect a mean frequency of 9.2 events per century. Composites based on these ERA5 TWP warming events show a TWP warming pattern in SST, together with a consistent East Antarctic high-pressure anomaly and enhanced precipitation over QW (Resp Fig. 4.6a,b). The 1,800-year CESM1 pre-industrial control simulation gives a similar event frequency and also produces significant composite Z300 dipole and precipitation anomalies over QW (Resp Fig. 4.6c,d). CESM1 historical simulations show comparable event frequency and similar composite patterns (Resp Fig. 4.6e,f). Thus, the composite results indicate that the TWP–East Antarctic teleconnection recurs on roughly decadal timescales in observations and model simulations.

To further address the concern that MCA3 may partly reflect an internal circulation mode, we use an Z300-based EA dipole index that does not contain any information on Antarctic precipitation. The correlation between this EA dipole index and global SST anomalies shows positive correlations over the TWP sector (Resp Fig. 4.23a). Conversely, the correlation between the TWP SST index and global Z300 anomalies shows a wave-like circulation pattern extending toward East Antarctica, including a dipole-like anomaly near the south-of-Australia/East Antarctic sector (Resp Fig. 4.23b). These results, which do not rely on MCA or precipitation-related calculations, support a recurrent linkage between TWP warming and the East Antarctic dipole circulation. Please see the revised manuscript at L217–219.

Line 271: If a sensitivity experiment is conducted with SST anomalies imposed over SPCZ (more align with observed anomalies and correlation patterns), would you also observe the dipole circulation anomalies? The authors argue ‘TWP warming is essential to producing the dipole circulation pattern over EA’. I am not yet fully convinced. It can produce does not mean it is the only way or a necessary requirement to produce.

Thank you for this comment. We have addressed this related issue in our earlier responses to your comments labeled Lines 36, 41, and 232 in your original review. Briefly, we conducted an additional ECHAM5

experiment with SPCZ SST forcing, but it does not reproduce the observed East Antarctic dipole circulation (Resp Fig. 4.10). In contrast, the TWP-only experiment reproduces the south-of-Australia low, the downstream East Antarctic high, and positive QW precipitation anomalies (Resp Fig. 4.2). The La Niña-only and IOD-only experiments do not produce a comparable response (Resp Fig. 4.3b,c), and the combined forcing experiment is broadly similar to, slightly stronger than, the TWP-only case (Resp Fig. 4.3a versus Resp Fig. 4.2).

Line 278: what does 'Gill-type' mean? In Fig. 3b, I am curious why the SST is increased more in the centre of the ellipse?

Thank you for pointing this out. "Gill-type" refers to the classical tropical atmospheric response to localized diabatic heating, with low-level convergence and upper-level divergence near the heating region, together with equatorial Kelvin- and Rossby-wave-like circulation responses. In this framework, local tropical heating can generate Rossby gyres to the west of the heating source. These Rossby gyres can then act as an effective source region for stationary Rossby wave propagation toward higher latitudes. Therefore, the fact that the diagnosed WAF appears to originate mainly from the Indian Ocean sector, rather than directly from the center of the TWP. Thus, it reflects the expected atmospheric response to localized TWP heating. We revised the text to define this term more clearly and cited Gill (1980). [L257–260]

The stronger SST anomaly in the center of the ellipse is part of an idealized forcing design. It represents a localized TWP warming pattern with the largest anomaly near the center of the warm pool and weaker anomalies toward the edge, rather than a direct reproduction of the observed SST anomalies. This smooth elliptical pattern was used to avoid unrealistically sharp SST gradients at the forcing boundary and to facilitate testing its role in a global warming scenario (Experiments 6 and 7), given that the future structure of the TWP in a warmer world remains uncertain. We also note that our results are not sensitive to the exact spatial shape of the imposed SST anomaly when the observed anomaly is imposed in the model. [L275–276]

Line 296: The sensitivity experiment does show that the La Nina-related cooling over the eastern Pacific alone cannot explain the dipole circulation, but it cannot differentiate TWP warming from a La Nina-related warming over the tropical western Pacific.

We do not intend to rule out the role of La Niña. Instead, our interpretation is that La Niña-related SST anomalies, IOD-related cooling, and TWP warming can interact with each other during the development, maintenance, and decay of this event. La Niña may contribute to the broader tropical background and may also enhance the SST warming impact over the TWP. Our sensitivity experiments were designed to test which part of the tropical SST anomaly more directly forces the East Antarctic dipole. The La Niña-like eastern-Pacific cooling-only experiment does not reproduce the observed East Antarctic dipole or the positive QW precipitation response, whereas the TWP warming experiment does (Resp Fig. 4.3b versus Resp Fig. 4.2). The combined experiment, including TWP warming, La Niña-like cooling, and IOD-like cooling, produces a response broadly similar to the TWP-only experiment (Resp Fig. 4.3a versus Resp Fig. 4.2). These results suggest that the direct forcing of the East Antarctic dipole is more closely tied to the localized TWP warming, while La Niña and IOD anomalies likely act as setting tropical background state.

This interpretation is dynamically consistent with the stationary Rossby wave pathway shown in our analysis. Local tropical heating, whether driven by SST anomalies or convection, can generate a Gill-type

response with a pair of Rossby gyres to the west of the heating source (Gill, 1980). These Rossby gyres can then act as an effective source region for stationary Rossby wave propagation toward higher latitudes. Therefore, the fact that the diagnosed WAF appears to originate mainly from the Indian Ocean sector (Resp Fig. 4.1 and Resp Fig. 4.2), rather than directly from the center of the TWP, does not contradict a TWP-driven mechanism. Instead, it reflects the expected atmospheric response to localized TWP heating. In addition, stationary Rossby wave dynamics indicate that tropical forcing tends to affect high-latitude circulation downstream of the forcing region. Therefore, eastern Pacific SST forcing more readily influences the Pacific sector and West Antarctica, whereas TWP forcing is more favorably positioned to influence the Indian Ocean sector, East Antarctica, and downstream regions. The observed TWP–EA teleconnection is therefore consistent with these physical constraints.

Line 303: 'lower latitudes' compared to what?

To shorten the manuscript, we have deleted this sentence in the revised manuscript.

Line 316: It seems to align with the findings of Gao et al., (2024) that Antarctic extreme precipitation is sourced from lower latitudes with smaller zonal transport compared to normal precipitation. Though they only publish data until 2022 (Gao et al., 2025), would it be worth to briefly check the overlapping period? Is it also worth using or noting the latest developments in process-oriented water tracing that could identify moisture source locations and environmental conditions associated with water phase change in iCAM6 (Fiorella et al., 2021), ECHAM6 (Gao et al., 2024), and UM (McLaren et al., 2025)?

Thank you for this helpful suggestion. Using the precipitation-weighted moisture source latitude and longitude data from Gao et al. (2025), we examined the QW sector during the overlapping period of July 2021 to December 2022. The results show that the mean moisture source shifted slightly equatorward during the event, while remaining within the Indian Ocean sector (Resp Figs. 4.27 and 4.28). In Resp Fig. 4.27, we first calculated monthly precipitation-weighted source latitude and longitude time series and then compared the event-period means with the 1979–2022 climatological means. In Resp Fig. 4.28, the mean source location was calculated directly using precipitation and area weights over all QW grid cells and months, giving a climatological mean source location of 82.26°E, 42.91°S and an event-period mean source location of 80.30°E, 41.61°S. Because the two calculations apply the weights in slightly different ways, the exact mean values differ slightly. However, both diagnostics consistently show that the moisture source for QW precipitation shifted modestly toward lower latitudes during the event. This overlapping-period comparison is consistent with Gao et al. (2024), who showed that Antarctic extreme precipitation was associated with moisture sources from lower latitudes. It also supports our interpretation based on the iCESM water-tagging results. We have added a brief note in the revised manuscript to mention this consistency. We have also modified Fig. 4b (Resp Figs. 4.29) to include the Gao et al. (2025) moisture-source locations. Please see the revised manuscript at L302–304 and Fig. 4b.

We also cited Gao et al. (2025) and recent process-oriented water-tracing studies, including Gao et al. (2024), Fiorella et al. (2021), and McLaren et al. (2025), as important recent developments along the same line of research. Please see the revised manuscript at L287–288.

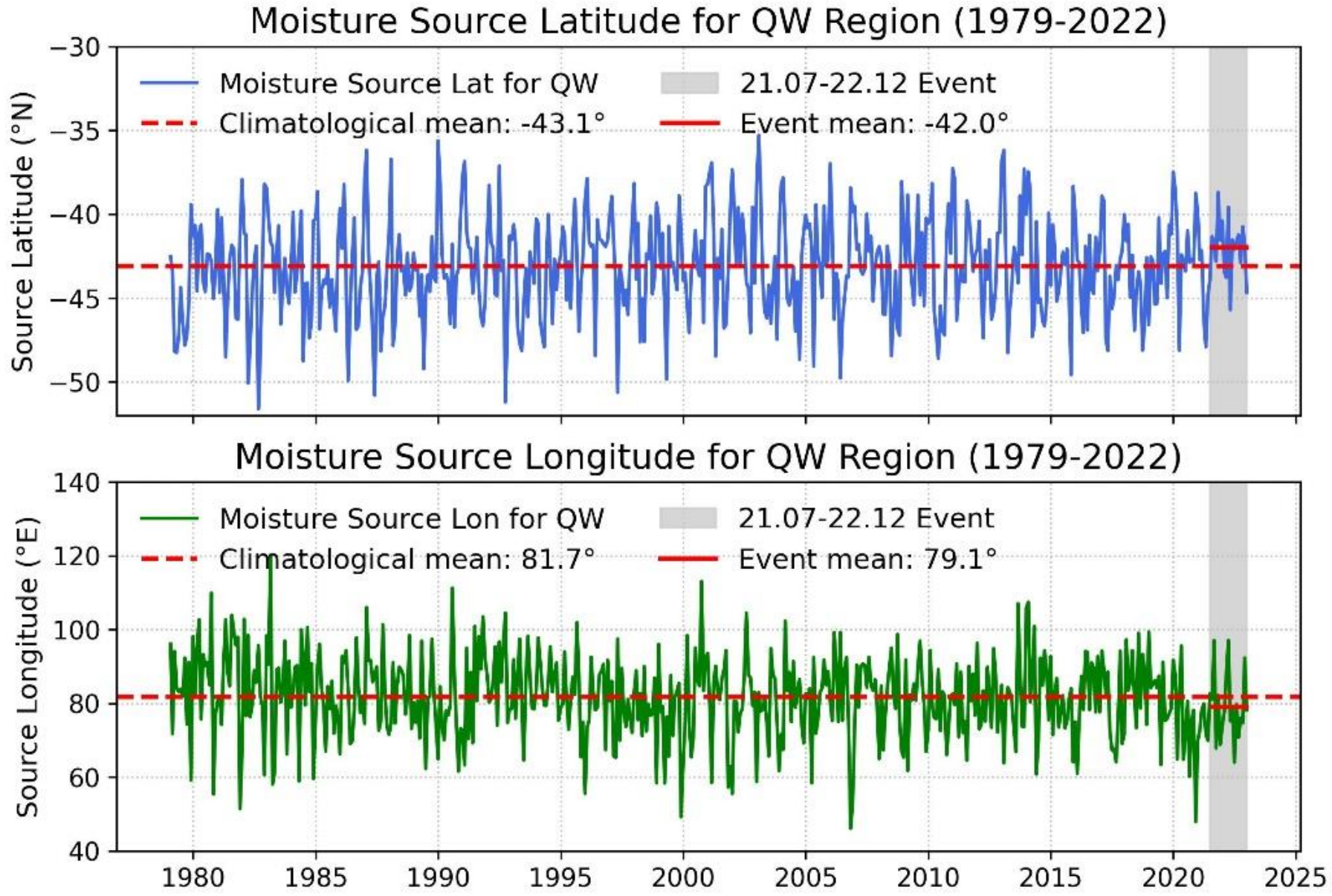

Resp Fig. 4.27. Time series of precipitation-weighted mean moisture source latitude and longitude for the QW region. The QW region (70–145°E, 65–75°S). The upper panel shows the monthly precipitation-weighted mean source latitude, and the lower panel shows the monthly precipitation-weighted mean source longitude during 1979–2022. Dashed red lines indicate the 1979–2022 climatological means. Grey shading indicates the extreme event period from July 2021 to December 2022. Solid red lines indicate the event-period means.

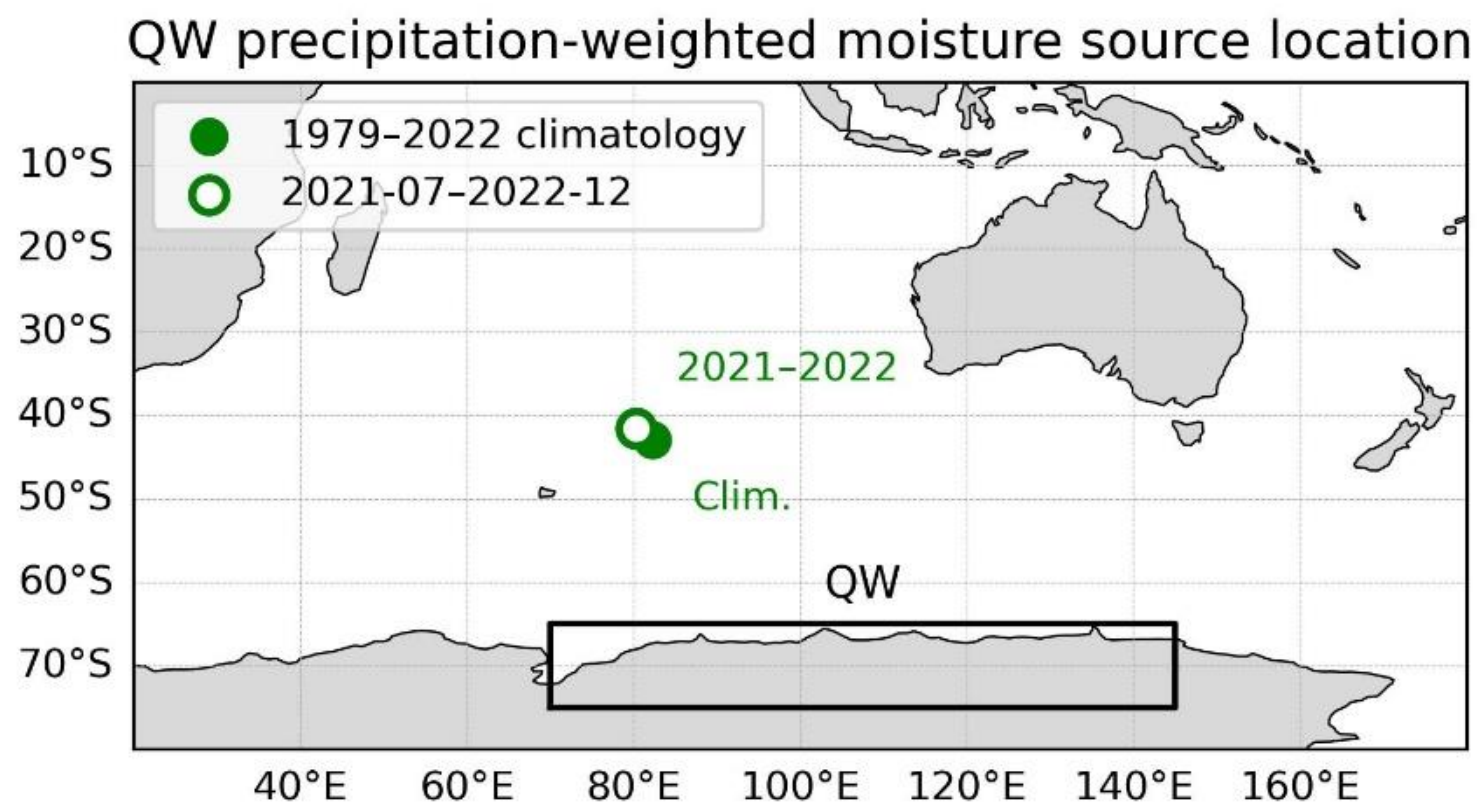

Resp Fig. 4.28. Precipitation-weighted mean moisture source location for the QW region. The black box indicates the QW region. The green filled circle represents the climatological mean moisture source location for 1979–2022 (82.26°E, 42.91°S), and the green open circle represents the mean moisture source location during the extreme event period from July 2021 to December 2022 (80.30°E, 41.61°S).

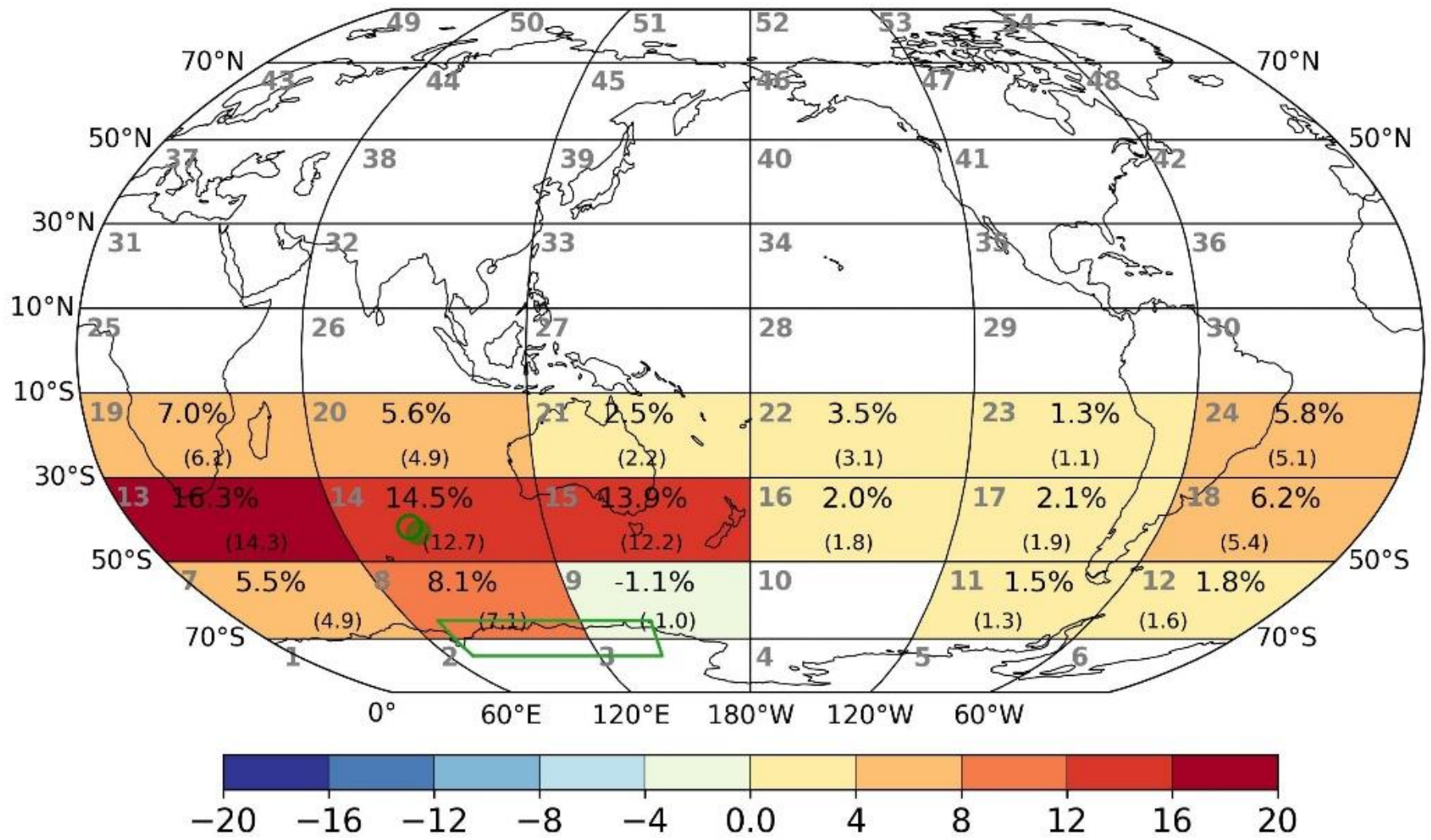

Resp Fig. 4.29 (also Fig. 4b in the main text). Moisture sources of QW precipitation anomalies during the 2021–2023 event. Contributions from each tagged moisture source region to the monthly QW precipitation anomaly during July 2021–April 2023. The value within each region denotes the percentage contribution to the total QW precipitation anomaly, with the absolute contribution shown below in parentheses in mm yr^{-1}. Percentages are calculated relative to the QW-domain total shown in the green box, which represents the area-weighted mean tagged precipitation anomaly summed across all 54 source regions. Only regions with a contribution exceeding 1% are shown. The green filled dot marks the climatological precipitation-weighted mean moisture-source location based on the water-tracer dataset of Gao et al. (2025) for 1979–2022, and the green open circle marks the corresponding mean moisture-source location during July 2021–December 2022.

Line 323: I am not really sure whether the Ferrel and polar cells are just patterns in the climatologically averaged fluxes or are really responsible for moisture and energy transport. Could the authors find a reference that documents their role in moisture and energy transport? I assume the authors mean 'the ascending branch' rather than 'the upper branch'? What are the relationships between 'storm-track activity and ARs' and is their role in moisture and energy transport quantified anywhere?

Thank you for the helpful comment. I appreciate the correction from 'the upper branch' to 'the ascending branch'. This term has been removed in the revised version. We would like to clarify that our intent was not to attribute the event solely to the zonal-mean Ferrel or polar cells. Rather, we intended to place the diagnosed moisture source regions within the broader climatological framework of high-latitude poleward moisture and energy transport. We have revised the text to make this clearer. Specifically, we describe the source regions as lying near the transition between the Ferrel and polar cells, where poleward moisture and energy transport is climatologically active. The anomalous transport during 2021–2023, however, is attributed to the TWP-related East Antarctic dipole circulation, which reorganizes the regional moisture pathway and favors moisture transport from the mid-latitude Indian Ocean into QW. This is consistent with our water-tagging results, which show that Zones 13–15 contribute about 45% of the positive QW precipitation anomaly, while evaporation over these key source regions is not significantly enhanced. ARs are narrow bands of strong moisture transport and are often associated with extratropical cyclones and frontal systems along storm tracks. In this sense, storm tracks provide the synoptic-scale circulation background, while ARs act as the strongest concentrated moisture transport pathways. The reference Li and

Ding (2024) showing the role of ARs in poleward moisture transport and Antarctic precipitation. To shorten the manuscript, we have deleted this sentence in the revised manuscript.

The analysis also shows that daily QW precipitation anomalies are strongly correlated with AR-frequency anomalies during July 2021–April 2023 ($r = 0.69$, $p < 0.01$; Resp Fig. 4.14), and the correlation remains strong after excluding the two most extreme AR days ($r = 0.67$). This supports the role of ARs as an important moisture transport pathway into QW during the event. Please see the revised manuscript at L226–229.

Li, Z. & Ding, Q. A global poleward shift of atmospheric rivers. Sci. Adv. 10, eadq0604 (2024).

Line 327: If the evaporation over the moisture source regions does not increase, it could be expected that precipitation along the transport pathways decreases, as can be seen in Fig. 3c. This may affect ocean surface salinity and thus deep water formation (if any). Could the authors briefly comment on this impact?

Thank you for this comment. We agree that moisture redistribution may affect precipitation and freshwater input along the transport pathway, with possible implications for sea-surface salinity, dense-water formation, and thermocline depth. However, our present experiments are atmosphere-only simulations with prescribed SST and sea ice, and our water-tagging analysis is also AMIP-type, with prescribed boundary forcing. These analyses are designed to diagnose moisture-source contributions rather than the ocean freshwater budget. Because the salinity response depends on the full freshwater balance, sea-ice processes, ocean mixing, advection, and ice-shelf freshwater input (Snow et al., 2016; Siahaan et al., 2024), we leave this issue as an open question for future studies.

Snow, K., Hogg, A. M., Sloyan, B. M., & Downes, S. M. (2016). Sensitivity of Antarctic Bottom Water to changes in surface buoyancy fluxes. Journal of Climate, 29, 313–330.
Siahaan, A., et al. (2024). Ice-shelf freshwater triggers for the Filchner–Ronne Ice Shelf melt tipping point in a global ocean–sea-ice model. The Cryosphere, 18(6), 2917-2936.

Line 333: Again, the cooling in Extended Data Fig. 11a is more significant over SPCZ.

We have addressed this related issue in our earlier responses to your comments labeled Lines 36, 41, and 232. Briefly, we conducted an additional ECHAM5 experiment with SPCZ SST forcing, but it does not reproduce the observed East Antarctic dipole circulation (Resp Fig. 4.10). In contrast, the TWP-only experiment reproduces the south-of-Australia low, the downstream East Antarctic high, and positive QW precipitation anomalies (Resp Fig. 4.2). The La Niña-only and IOD-only experiments do not produce a comparable response (Resp Fig. 4.3b,c), and the combined experiment is broadly similar to the TWP-only case (Resp Fig. 4.3a versus Resp Fig. 4.2). SST anomalies along the SPCZ extend into the subtropics, where the convective response and upper-level divergence are generally less effective at exciting a strong Rossby wave train toward East Antarctica than heating over the tropical warm pool. Thus, we interpret the SPCZ signal as part of the broader La Niña–TWP SST pattern extending into the subtropics, rather than as the main forcing of the East Antarctic teleconnection.

Line 369: Fig. 5a, what does x-axis mean? What is the average duration of these events?

Thank you for pointing this out. The x-axis represented the cumulative TWP SST anomaly during each detected event, with units of K, and the average event duration was 29.8 months. To shorten the manuscript, we removed Fig. 5 of the original manuscript. In the revised manuscript, the occurrence frequencies of TWP events derived from ERA5 and CESM1 simulations are now discussed in Extended Data Fig. 12.

Line 381: Extended Data Fig. 12a, the warming pattern is quite different from those in Fig. 2b and 5c. Although precipitation is lacking before 1979, ice core measurements from (Medley and Thomas, 2018) may still be more reliable than ERA-20C, which I assume is simply a model product without observational constraints before 1979? The precipitation anomalies in Extended Data Fig. 12b are small and insignificant over QW. Could you compare the difference with the 2021-23 event?

Thank you for this comment. We replaced this analysis with a new composite based entirely on ERA5 for 1950–2025 given the larger uncertainties in ERA20C. Specifically, we identified prolonged TWP warming events using the ERA5 SST index and then composited the corresponding ERA5 SST, Z300, and precipitation anomalies. The revised composite shows a clear TWP warming pattern (Resp Fig. 4.6a) that is much more consistent with the SST anomaly during 2021–2023 (Resp Fig. 4.9a). It also shows an East Antarctic high-pressure anomaly and positive precipitation anomalies over the QW sector (Resp Fig. 4.6b), consistent with the observed 2021–2023 event (Resp Fig. 4.13).

Because the Medley and Thomas (2019) regional reconstruction ends in 2000 and does not cover the GRACE era, we used the original annual Law Dome ice-core snow-accumulation record compiled by Thomas (2017), which extends to 2010. Since this ice-core record is annual, it cannot be composited using our monthly TWP warming-event definition. We therefore used a simpler annual approach based on the new East Antarctic dipole index constructed only from Z300 anomalies. Specifically, we converted the monthly Z300 dipole index to annual means over 1950–2010, and applied the same detrending and standardization to both the Z300 index and the Law Dome record. We then averaged the Law Dome accumulation anomalies for years when the standardized Z300 dipole index exceeded +1 standard deviation or was below −1 standard deviation. During positive Z300 dipole years, the mean standardized Law Dome accumulation anomaly is positive (+0.42 standard deviation), whereas during negative Z300 dipole years it is negative (−0.43 standard deviation). This supports our interpretation that East Antarctic high-pressure anomalies are associated with enhanced snow accumulation at Law Dome. Please see L348–349 in the revised manuscript and L245–249 in the Extended Data.

As described in our response to Major Comment 3, we also added a long-record check using EKF400v2 and LMR v2.1 paleo-reanalyses. The QW precipitation index is associated with a similar East Antarctic high-pressure anomaly and a low-pressure anomaly south of Australia in both datasets (Resp Fig. 4.7a,c). In addition, the corresponding EKF400v2 2-m air temperature and LMR v2.1 SST correlations show positive anomalies over the TWP sector (Resp Fig. 4.7b,d). These results further support that the QW precipitation-related circulation pattern is robust beyond the modern reanalysis period and is accompanied by tropical surface warming features consistent with the TWP–East Antarctic teleconnection. Please see Extended Data Fig. 13 in revised manuscript.

Line 391: Extended Data Fig. 12c&d, The circulation anomalies are displaced from EA and the precipitation anomalies seem small.

We agree that the circulation and precipitation responses in the CESM1 pre-industrial composite are weaker and less spatially confined to QW than the observed 2021–2023 event. Our intention in using Extended Data Fig. 12c,d was not to suggest that most TWP warming events produce the similar dipole circulation mode centered over East Antarctica. Rather, this figure provides a long-record check, showing that prolonged TWP warming events tend to be associated with a broadly consistent high-latitude circulation response and positive precipitation anomalies over East Antarctica. The weaker amplitude is expected because the composite is based upon the averages of 180 model-simulated events, each of which likely possesses a different peak seasons and duration, is likely to be embedded in different Southern Hemisphere background states and regimes, and is characterized by slightly different wave-train patterns. Such averaging may smooth and slightly displace the circulation centers and reduce the precipitation signal. In contrast, the observed 2021–2023 event represents one real-world realization with stronger persistence, a more favorable background state, and a sharper regional expression. The main evidence for the TWP–East Antarctic mechanism is derived from the diagnostics of the observed event in ERA5 and the AGCM sensitivity experiments. Extended Data Fig. 12c,d is used as supporting evidence for recurrence and statistical consistency, not as the primary proof of the exact spatial structure.

Line 447: 'poleward-shifted storm tracks': The author argued that 'This transport is further facilitated by storm-track activity and ARs' (Line 324), and argued here that a poleward shift of storm track is insufficient for the explanation of this event (Line 473). The poleward-shift is an average pattern and constitutes the behaviours of many storms. Since the storms or ARs here shift to the EA, it is more poleward than other SH storms or ARs. Could the author really argue it is unrelated and rule out the potential role of all other mechanisms proposed to explain the poleward shift of storm tracks?

We agree that poleward-shifted storm tracks and ARs are related to the event, and we did not intend to rule out any possible contribution from global warming or from the long-term poleward shift of SH storm tracks. Our point is that a zonal-mean, long-term poleward shift of storm tracks alone is insufficient to explain the 2021–2023 event. The observed event was highly regional, concentrated over QW, and accompanied by a clear dipole circulation pattern with a high-pressure anomaly over East Antarctica and a low-pressure anomaly to the north. This differs from a broad hemispheric storm-track displacement pattern favored by global warming effects across models. In the revised manuscript, we have shortened and simplified the Discussion. The sentence at Line 473 in the original manuscript has been removed.

We have also added a quantitative comparison argument. Based on the CESM1 ensemble-mean cumulative precipitation series (Resp Fig. 4.17), the forced precipitation increase over QW from July 2021 to April 2023 is only 32.3 ± 2.4 Gt, corresponding to about 9% of the observed ERA5 cumulative precipitation anomaly over the same region and period (351.2 Gt). This comparison suggests that forced thermodynamic moistening and storm track changes may have contributed to the global-warming-related background signal, but cannot explain the majority of the observed event magnitude. We therefore interpret the 2021 − 2023 event as being dominated by the dynamic component. This dynamic component is closely linked to the TWP-related teleconnection identified in our MCA and AGCM experiments. [L156-161]

Line 464: I do not see any discussions about sea ice. Could the record-low sea ice extent in 2022-23 contribute to the organization of the low/high pressure systems?

Thank you for this comment. We agree that the record-low Antarctic sea-ice extent in 2022–2023 may have affected near-surface heat and moisture fluxes. However, the circulation anomaly emphasized in our study is an upper-tropospheric, quasi-barotropic dipole, with a low-pressure anomaly south of Australia and a high-pressure anomaly over the East Antarctic coast. This structure is closely linked to the MCA3 circulation mode and to Rossby wave propagation from the TWP. In addition, the TWP-only AGCM experiments reproduce a similar dipole circulation and enhanced East Antarctic precipitation without imposing the observed 2022–2023 sea-ice anomaly, suggesting that sea-ice loss is not necessary to generate the large-scale circulation pattern. This interpretation is also consistent with Kolbe et al. (2026). They showed that recent Antarctic mass-balance reversal was linked to increased precipitation associated with more frequent ARs, and reduced sea-ice extent, but their sensitivity experiments indicate that the precipitation enhancement from sea-ice loss through increased evaporation accounts for only about 10% of the winter increase and is overall minor compared with remote large-scale processes. We cited Kolbe et al. (2026) in the revised manuscript.

Kolbe, M. et al. Atmospheric rivers and winter sea ice drive recent reversal in Antarctic ice mass loss. Commun. Earth Environ. 7, 255 (2026).

Benjamini, Y. and Hochberg, Y.: Controlling the False Discovery Rate: A Practical and Powerful Approach to Multiple Testing, Journal of the Royal Statistical Society: Series B (Methodological), 57, 289–300, https://doi.org/10.1111/J.2517-6161.1995.TB02031.X, 1995.
Fiorella, R. P., Siler, N., Nusbaumer, J., and Noone, D. C.: Enhancing Understanding of the Hydrological Cycle via Pairing of Process-Oriented and Isotope Ratio Tracers, J. Adv. Model. Earth Syst., 13, e2021MS002648, https://doi.org/10.1029/2021MS002648, 2021.
Gao, Q., Sime, L. C., McLaren, A. J., Bracegirdle, T. J., Capron, E., Rhodes, R. H., Steen-Larsen, H. C., Shi, X., and Werner, M.: Evaporative controls on Antarctic precipitation: an ECHAM6 model study using innovative water tracer diagnostics, Cryosphere, 18, 683–703, https://doi.org/10.5194/TC-18-683-2024, 2024.
Gao, Q., Sime, L. C., McLaren, A. J., and Werner, M.: Datasets for the article “Moisture source controls on water isotopes in Antarctic precipitation - insights from water tracers in ECHAM6-wiso” [Dataset], Zenodo, https://doi.org/10.5281/zenodo.15107251, 2025.
Goyal, R., Jucker, M., Gupta, A. Sen, England, M. H., Goyal, R., Jucker, M., Gupta, A. Sen, and England, M. H.: A New Zonal Wave-3 Index for the Southern Hemisphere, J. Clim., 35, 5137–5149, https://doi.org/10.1175/JCLI-D-21-0927.1, 2022.
Li, X., Cai, W., Meehl, G. A., Chen, D., Yuan, X., Raphael, M., Holland, D. M., Ding, Q., Fogt, R. L., Markle, B. R., Wang, G., Bromwich, D. H., Turner, J., Xie, S. P., Steig, E. J., Gille, S. T., Xiao, C., Wu, B., Lazzara, M. A., Chen, X., Stammerjohn, S., Holland, P. R., Holland, M. M., Cheng, X., Price, S. F., Wang, Z., Bitz, C. M., Shi, J., Gerber, E. P., Liang, X., Goosse, H., Yoo, C., Ding, M., Geng, L., Xin, M., Li, C., Dou, T., Liu, C., Sun, W., Wang, X., and Song, C.: Tropical teleconnection impacts on Antarctic climate changes, ， 2021 2:10, 2, 680–698, https://doi.org/10.1038/s43017-021-00204-5, 2021.
McLaren, A. J., Sime, L. C., Wilson, S., Ridley, J., Gao, Q., Gorguner, M., Line, G., Werner, M., and Valdes, P.: Implementation of water tracers in the Met Office Unified Model, Geosci. Model Dev., 18, 8129–8142, https://doi.org/10.5194/gmd-18-8129-2025, 2025.
Medley, B. and Thomas, E. R.: Increased snowfall over the Antarctic Ice Sheet mitigated twentieth-century sea-level rise, Nat. Clim. Chang., 9, 34–39, https://doi.org/10.1038/s41558-018-0356-x, 2018.
Palerme, C., Claud, C., Dufour, A., Genthon, C., Wood, N. B., and L’Ecuyer, T.: Evaluation of Antarctic

snowfall in global meteorological reanalyses, Atmos. Res., 190, 104–112, https://doi.org/10.1016/J.ATMOSRES.2017.02.015, 2017.
Wang, D., Ma, H., Li, X., Hu, Y., Hu, Z., An, C., Ding, M., Li, C., Jiang, S., Li, Y., Lu, S., Sun, B., Zeng, G., van den Broeke, M., and Shi, G.: Sustained decrease in inland East Antarctic surface mass balance between 2005 and 2020, Nature Geoscience 2025 18:6, 18, 462–470, https://doi.org/10.1038/s41561-025-01699-z, 2025a.
Wang, S., Liu, J., Cai, W., Yang, D., Kerzenmacher, T., Ding, S., and Cheng, X.: Strong impact of the rare three-year La Niña event on Antarctic surface climate changes in 2021–2023, npj Climate and Atmospheric Science 2025 8:1, 8, 173-, https://doi.org/10.1038/s41612-025-01066-0, 2025b.

We sincerely thank the editor and referees for their continued careful evaluation of our manuscript and for the positive assessment of the revised version. In this revision, we have focused on the remaining scientific and editorial issues, especially the distinction between TWP warming and La Niña forcing, as well as the production-ready formatting requirements.

The main changes are summarized below:

1. We revised the title by replacing “prolonged” with “multiyear,” which better reflects that the TWP warming discussed here is a recurring multi-year SST anomaly rather than a sustained long-term trend.

2. We added new analyses addressing the remaining concern about TWP warming versus La Niña. These include three new ECHAM5 AMIP-style experiments using the observed 2021–2023 SST anomaly pattern, and CESM1 pre-industrial composites comparing strong TWP warming, strong La Niña, and IOD-like cooling states. These results show that the EA high-pressure anomaly and enhanced QW precipitation are more closely tied to TWP warming than to La Niña or IOD-like cooling.

3. We further addressed the remaining comments from Referees 1–3 by clarifying the March 2022 AR case, the contribution from the Antarctic Peninsula, and the wording related to the spatial extent of our mechanism.

4. We completed the editorial and formatting revisions requested by the editor. These include moving the mechanism schematic into the main text, moving extended-text sections into the Methods, removing the Supplementary Information, limiting the main-text references to fewer than 50, making all legends self-contained, revising color schemes for readability, verifying area-weighted calculations, and updating the Code Availability statement and figshare repository.

Below, we provide point-by-point responses to the remaining referee comments. Reviewer comments are shown in black, and our responses are shown in blue.

Referees' comments:
Referee #1 (Remarks to the Author):

General comments

The authors have addressed most of my comments in a satisfactory manner. The manuscript does appear more robust with a cleaner narrative in this latest version. I feel this manuscript represents an important advancement in understanding tropical-Antarctic teleconnections and will serve as a good example in terms of methodology for future studies when making similar tropical-polar teleconnection studies. Overall, I support the publication of this manuscript after the following specific comment and minor comments are addressed.

We thank you for your positive assessment of the revised manuscript. Your comments in the previous round helped us greatly improve the presentation of the role of our new teleconnection in local AR activity, and we have further addressed your remaining points below. Reviewer comments are shown in black, and our responses are shown in blue.

Specific comments

1. There is one comment from my previous review that I would like to see further investigated. Regarding the March 2022 extreme East Antarctic AR, it was obvious that the event was accompanied by an increase in AR activity and precipitation in the Queen Mary Land and Wilkes Land. I was hoping instead that you could look at the tropical SST conditions and Rossby wave activity during March 2022 and see if there are any similarities with the TWP SST patterns identified in your analysis. I thought it could be nice for the reader to see a concrete example of a precipitation event during the 2021-2023 that fits the narrative of the TWP/QW precipitation teleconnection narrative. Of course, if word limits are a problem, then do not worry about trying to include this example as it is not a crucial component of the study.

We thank you for this suggestion. Following your comment, we examined the SST anomalies, Z300 anomalies, and Rossby wave activity flux (WAF) during March 2022. As shown in Response (Resp.) Fig. 1.1, March 2022 was characterized by positive SST anomalies over the TWP region. The monthly mean Z300 and WAF anomalies show a wave-train-like structure extending from the Indo-Pacific sector toward the South Pacific, with a high-pressure anomaly over East Antarctica (EA). Because a 30-day window may still contain fast processes, such as synoptic and intraseasonal variability, this pattern does not exactly match the longer-term dipole pattern identified in our MCA or model simulations. This is understandable, as our study focuses on a persistent 2–3-year process more directly linked to tropical SST forcing. In addition, because the extreme precipitation and AR anomalies were concentrated around mid-March, we also examined the circulation anomalies from 10 to 20 March, a key time window during which the extreme AR occurred. This shorter-period average shows a clearer EA high-pressure anomaly and associated WAF anomalies directed toward EA, bearing more resemblance to the TWP–EA teleconnection identified in the MCA analysis and our ECHAM5 experiments, such as Experiment 1.

This test supports the mechanism described in the manuscript: prolonged TWP warming from July 2021 to April 2023 increased the likelihood of AR intrusions into QW by generating a dipole teleconnection pattern. However, a one-month pattern may contain the tropically-forced teleconnection and the internal atmospheric

variability on synoptic and intraseasonal timescales. Such a short-period analysis is beyond the scope of this work, and we have therefore not added this case to the main text.

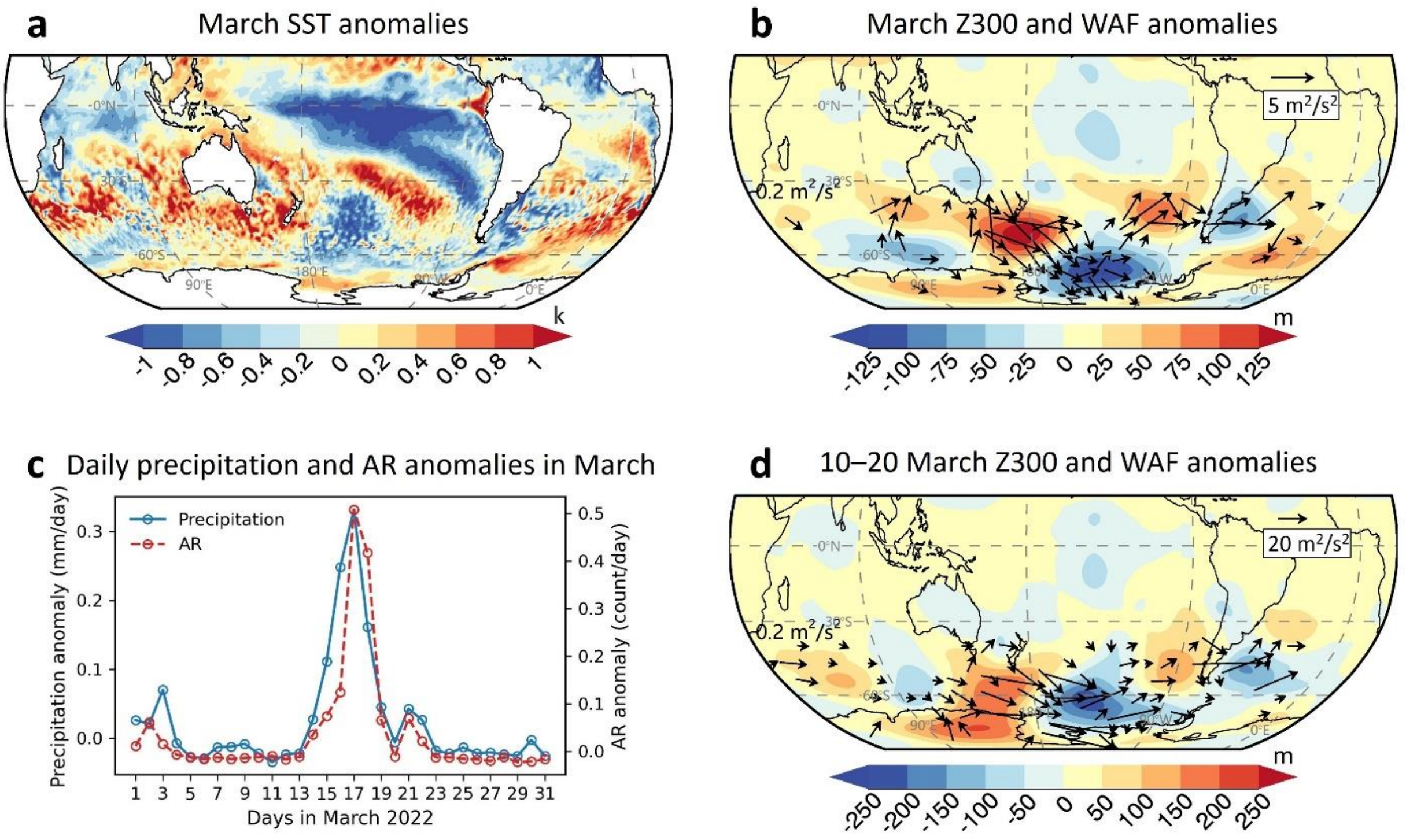

Resp. Fig. 1.1. SST, Z300, WAF, precipitation, and AR anomalies during March 2022. ***a****, Monthly mean SST anomalies in March 2022.* ***b****, Monthly mean 300 hPa geopotential height (Z300) anomalies and associated Rossby wave activity flux (WAF; black vectors) in March 2022.* ***c****, Daily precipitation and AR anomalies in March 2022.* ***d****, As in* ***b****, but averaged over 10–20 March 2022, spanning the peak precipitation and AR anomalies shown in c. Anomalies are calculated after removing the climatological seasonal cycle and linear trend over 1979–2024.*

Minor comments

Fig. 1e: The AR anomaly contours are a nice addition, but I find it difficult to follow the contours. In the current form, it is hard to tell if the red contours imply that there were positive AR anomalies around most of coastal Antarctica or just in the interior although I assume the answer is coastal Antarctica. I guess the red and blue contours naturally end at the coastline as you are only showing AR anomalies over land? Can you also color the coastline where relevant so that the contours represent enclosed regions?

We thank you for pointing this out. We have revised Fig. 1e by adding multiple positive and negative AR-frequency anomaly contours, which better separate coastal from interior anomalies and show that the positive AR anomalies are concentrated along coastal East Antarctica.

Line 72: "Rising height trend of the tropopause". Just to make it clearer for readers not familiar with the tropopause.

Thank you. We have revised this to "the rising trend in tropopause height." [L73]

Line 225: The phrasing makes it sound like the Rossby wave train is a continuous feature. Suggest

rephrasing it to “which increases the likelihood of a triggering a Rossby wave train that propagates towards the high southern latitudes and …”

We have revised this to “which favors a Rossby wave train propagating toward high southern latitudes.” [L192]

Line 282: I recommend adding a quick sentence or a few works pointing the reader towards the “Extended Text for Extended Data Fig. 8: Sensitivity Experiments 2–7” just so the reader is aware that a greater discussion on the sensitivity experiments is available.

We have revised this sentence to point readers to both Extended Data Figs. 8 and 9 and the Methods, where the sensitivity experiments are discussed. [L234]

Referee #2 (Remarks to the Author):
I co-reviewed this manuscript with one of the reviewers who provided the listed reports.

Referee #3 (Remarks to the Author):
The authors have presented a major revision to their paper. I am generally content with their revision and remain enthusiastic about their idea and the manuscript.

We thank you for your very positive assessment of the revised manuscript. We are glad that the revision has addressed your main concerns and that you are pleased with the main content of our work. Reviewer comments are shown in black, and our responses are shown in blue.

The one part of the revision I am still not entirely content with is related to the clear growth in the Antarctic Peninsula. This does not negate the current analysis but it clearly doesn't allow the perception to be given that WL is the only relevant area as it still is in L192 or L339. The argument that other modes are secondary modulators L342 requires that MCA1 and 2 are just a redistribution of mass with zero net effect. So I think a further refinement of language is required.

Thank you. Our manuscript notes the mass gain over the Antarctic Peninsula, while focusing on QW because it was the dominant contributor, accounting for about 68% of the continent-wide mass increase. We have revised the wording to clarify the scope of our focus. Specifically, we now describe the MCA3 precipitation pattern as having a spatially integrated positive effect over Antarctica, rather than implying positive anomalies everywhere [L171]. We have also specified that "secondary modulators" refers to the QW mass change. [L287]

L116 the law Dome ice core shown in Extended Figure 1b does not go past 2010. So there is a major error in "precipitation anomaly during July 2021–April 2023, which is evident in all three precipitation datasets and is also observed in the snow-accumulation record from the Law Dome ice core (Fig. 1f–h and Extended Data Fig. 1b)." If teh data somehow does go beyond 2010 but is not visibile in the plot then a new data reference is required as Thomas et al do not include data up to date.

We have corrected this sentence. We now state that the July 2021–April 2023 anomaly is evident in the three precipitation datasets, while the Law Dome record supports ERA5 precipitation variability over the overlapping period. [L117]

Minor comments:
Abstract: 'moistening of Antarctica' is a strange phrase and does not reflect what is happening. Antarctica is not becoming more moist! Maybe just 'Therefore, the recent Antarctic Ice Sheet mass gain is likely temporary and does not yet reflect a sustained, global warming-driven surface-mass shift.'

We thank you for raising this point. We retain "moistening" here to emphasize that the mass gain is not simply a consequence of global-warming-driven precipitation increases associated with the poleward shift of storm tracks. As we mentioned this poleward shift and moistening of Antarctica earlier in the Abstract, it is more natural to conclude the Abstract with the same concept. The idea of Antarctic moistening has been widely discussed in previous studies and IPCC AR6 (e.g., Frieler et al., 2015; Nicola et al., 2023).

Nicola, L., Notz, D. & Winkelmann, R. Revisiting temperature sensitivity: how does Antarctic precipitation change with temperature? Cryosphere 17, 2563-2583 (2023).
Frieler, K. et al. Consistent evidence of increasing Antarctic accumulation with warming. Nat. Clim. Change 5, 348-352 (2015).
IPCC. Regional fact sheet – Polar Regions. In Climate Change 2021: The Physical Science Basis. Contribution of Working Group I to the Sixth Assessment Report of the Intergovernmental Panel on Climate Change (2021).

L46 the uncertainty is over some timeframe. multidecadal or centennial or something "long term uncertainty". the uncertainty is not largest this decade.

We have revised this to refer to long-term sea-level projections. [L46]

L50: "In contrast, the current mass balance of the East Antarctic Ice Sheet (EAIS), which contains nearly 80% of Earth's land ice, *is more sensitive to large* precipitation-driven variability".

We have revised this to "is more sensitive to precipitation-driven variability." [L52]

L52 it is not clear why ice shelf melt is introduced here and what the link to snowfall variability is. If the desire is to link to ice dynamic changes, then I think invoking change in basal melt is required. I don't see the value of this sentence though and think the snowfall variability part can be merged with teh previous sentence

We have removed the ice-shelf basal-melt comparison and merged the snowfall-variability point into the preceding sentence. [L52]

L52 diverge *from zero and* beyond their uncertainties

We have revised this to "diverge from zero beyond their uncertainties." [L54]

L98 " halted between July 2021 and April 2023" - htese are very precise dates. approximately? Is the end confidently locked to mid 2023 or did it continue longer?

We have revised "halted between" to "interrupted by pronounced mass gain between July 2021 and April 2023" to avoid implying an exact termination date. [L99]

L101 delete "has"

We have deleted "has." [L102]

L262 check for circulation figure ref is correct as not on Fig 1

Fig. 1e shows the circulation anomalies for the 10 strongest QW precipitation months during the event. We have revised the reference to Extended Data Fig. 2a, which shows the mean Z300 anomaly for the full July 2021–April 2023 event. [L230]

Referee #4 (Remarks to the Author):

Thank you, I really appreciate your efforts in addressing my questions. I can see most questions are addressed in satisfactory ways. However, my major concern regarding the link between TWP and La Nina, as also pointed out by other reviewers, remains. I consider it sloppy to characterize La Nina as just cooling over eastern tropical Pacific, though the index is normally defined based only on SST in this region. My detailed comments are listed below.

The link between TWP and La Nina was suggested by a few reviewers for better clarification, and it is addressed by the authors through sensitivity experiments with La-Nina-like cooling over eastern tropical Pacific. I consider it an oversimplification to describe La Nina as just cooling in this region, though it is defined based on SST in this region (e.g. Nino 3.4). La Nina is a basin-wide phenomenon, characterised by a stronger Walker Circulation in the atmospheric part, warmer-than-usual SST in its ascending branch, and colder-than-usual SST in its descending branch (See Wikipedia 2020–2023 La Niña event, https://en.wikipedia.org/wiki/2020%E2%80%932023_La_Ni%C3%B1a_event). A focus on the cooling part while ignoring the warming part close to TWP is not satisfactory.

My null hypothesis is that TWP warming proposed by the authors is indeed part of the triple La Nina. I believe the author would agree that the real world will never exhibits TWP warming as in the ECHAM5 simulations, and a slight shift (north-south or east-west by a few degrees) or reshape (more circular or rectangle) of the TWP warming could still excites Rossby wave train and favours the dipole pattern near Antarctica. This simple fact raises concern that the impact of TWP warming may be hardly distinguishable from the impact of the warming associated with La Nina. And I speculate they are basically the same thing named differently when their spatial extent is slightly different. To test this hypothesis, I would suggest another set of ECHAM5 sensitivity experiments, where SST anomalies associated with La Nina (this triple event or a composite from multi-decades SST data) over the whole tropical regions (not just cooling over eastern tropical Pacific) and over the globe (just to test the role of extratropical regions, even if negligible) are superimposed on the climatological SST. If these experiments also show dipole pattern near Antarctica and precipitation anomalies over QW, then it suggests the teleconnection patterns may be better discussed within the framework of ENSO-Antarctica teleconnections. In this case, I would wonder how much scientific value this paper adds for our understanding of the global impacts of ENSO, and how innovative it is to detect a different teleconnection pathway that is just slightly shifted eastward or westward.

I would expect some discussions on the mechanisms regarding how TWP warming, or La Nina warming, is linked to intensified or more concentrated convection over the ascending branch of the Walker Circulation, and how these convection activities are linked to Rossby wave train. If the findings can be framed within the context of the impact of strengthened Walker Circulation on Antarctic climate, it may be helpful to incorporate the role of IOD.

It could be expected that La Niña-like cooling or IOD-like cooling are not conducive to convection and thus Rossby wave excitation. It is interesting that they help to produce a response slightly stronger than that in the TWP-only experiment. This seems to suggest that more concentrated convective activities in the ascending branch of the Walker circulation, which are favoured by warming over TWP and cooling over eastern tropical Pacific and tropical Indian Ocean, are more effective at excites Rossby wave train.
Resp Fig. 4.6a, Resp Fig. 4.7d, Resp Fig. 4.9b, and Resp Fig. 4.23a resemble what may be expected from La

Nina events in tropical Pacific. Resp Fig. 4.22. and Resp Fig. 4.23c suggests that while controlling ENSO and IOD influences, partial correlation between the MCA3 Z300 index and TWP SST is insignificant.

We thank you for raising this point. To distinguish the relative roles of tropical warm pool (TWP) SST warming and La Niña forcing in shaping the East Antarctic (EA) dipole, we added two further analyses. Reviewer comments are shown in black, and our responses are shown in blue.

Following your suggestion to test the response to an observed triple-La Niña-related SST pattern, we performed ECHAM5 AMIP-style experiments using the observed 2021–2023 SST anomaly pattern. In the first experiment, the observed tropical sea surface temperature (SST) anomalies from 2021 to 2023 are imposed in ECHAM5, with the same setting as Experiments 1–8 in the main text. In the second experiment, tropical and extratropical SST anomalies within 60°S–60°N during the same three-year period are prescribed. In the third experiment, we used the same setup as in new Experiment 1, except that the observed SST anomalies over the TWP region were reduced by 50%. In Experiment 1, imposing the full tropical SST anomaly during the three-year period reproduces the EA circulation dipole and positive Queen Mary Land and Wilkes Land (QW) precipitation anomalies (Resp. Fig. 4.1). Expanding the imposed SST anomaly from the tropics to 60°S–60°N produces a similar response, although the EA high-pressure anomaly shifts slightly westward compared with panel a, indicating that extratropical SST anomalies are not the primary driver of the observed dipole pattern over EA. Most importantly, when TWP warming within the boxed TWP region is reduced to half of its observed amplitude while the rest of the 2021–2023 SST anomaly pattern is retained, the EA high-pressure response becomes insignificant and negative QW precipitation anomalies increase. This experiment provides strong support that TWP warming plays a vital role in creating the tropical–EA teleconnection proposed in this study.

We also agree with you that the leading teleconnections in the Southern Hemisphere are still dominated by ENSO-related processes, as ENSO can effectively modulate the TWP region by influencing the Walker Circulation and Hadley Cell. We have emphasized this point in the main text. [L316–319]

As recommended by the editor, we also used the long CESM1 pre-industrial simulation to construct composites for three sets of climate states: strong TWP warming with weak La Niña, strong La Niña with weak TWP warming, and concurrent strong TWP warming and strong La Niña. Strong TWP warming (>1 standard deviation; see Resp. Fig. 4.2 caption for details) with weak La Niña (-1 < Niño3.4 < 0 standard deviation) produces an EA high-pressure anomaly and positive QW precipitation anomalies resembling the observed teleconnection, based on 1,729 monthly samples (Resp. Fig. 4.2). In contrast, weak TWP warming (0 < TWP < 1 standard deviation) with strong La Niña (Niño3.4 < -1 standard deviation) does not produce an EA high-pressure anomaly and is associated mainly with negative QW precipitation anomalies, based on 1,448 monthly samples. When strong TWP warming and strong La Niña occur together, the EA high-pressure and positive QW precipitation responses remain clear, based on 562 monthly samples. These composites show that the EA high-pressure anomaly and enhanced QW precipitation are more closely tied to strong TWP warming than to strong La Niña forcing. We have added this result to the main text [Please see Lines 310–315] and Extended Data Fig. 13.

Additional tests comparing the effects of TWP warming and Indian Ocean Dipole (IOD)-like cooling also show the dominant role of TWP warming: strong TWP warming can effectively generate the EA dipole pattern, whereas strong IOD-like cooling cannot (Resp. Fig. 4.3). These results also suggest that TWP

warming, IOD-like cooling, and La Niña may not always co-occur. In many months, they can occur independently of one another. This is very consistent with our understanding of SST variability over the TWP from previous research (Wang et al., 2005), which shows that SST variability in this region is strongly influenced by atmosphere–ocean interactions and Asian–Australian monsoon forcing, whereas SST variability in the El Niño/La Niña region is more strongly driven by ENSO related oceanic processes. Thus, TWP SST change may still have some independent variability that is not simply attributable to either ENSO or IOD forcing.

We appreciate your very constructive comments raised in this round and the previous round, all of which have helped us better understand our results and improve the presentation of the key mechanism behind the TWP–EA linkage.

Wang, B., Ding, Q., Fu, X., Kang, I.-S., Jin, K., Shukla, J. & Doblas-Reyes, F. (2005). Fundamental challenge in simulation and prediction of summer monsoon rainfall. Geophysical Research Letters, 32(15). https://doi.org/10.1029/2005GL022734

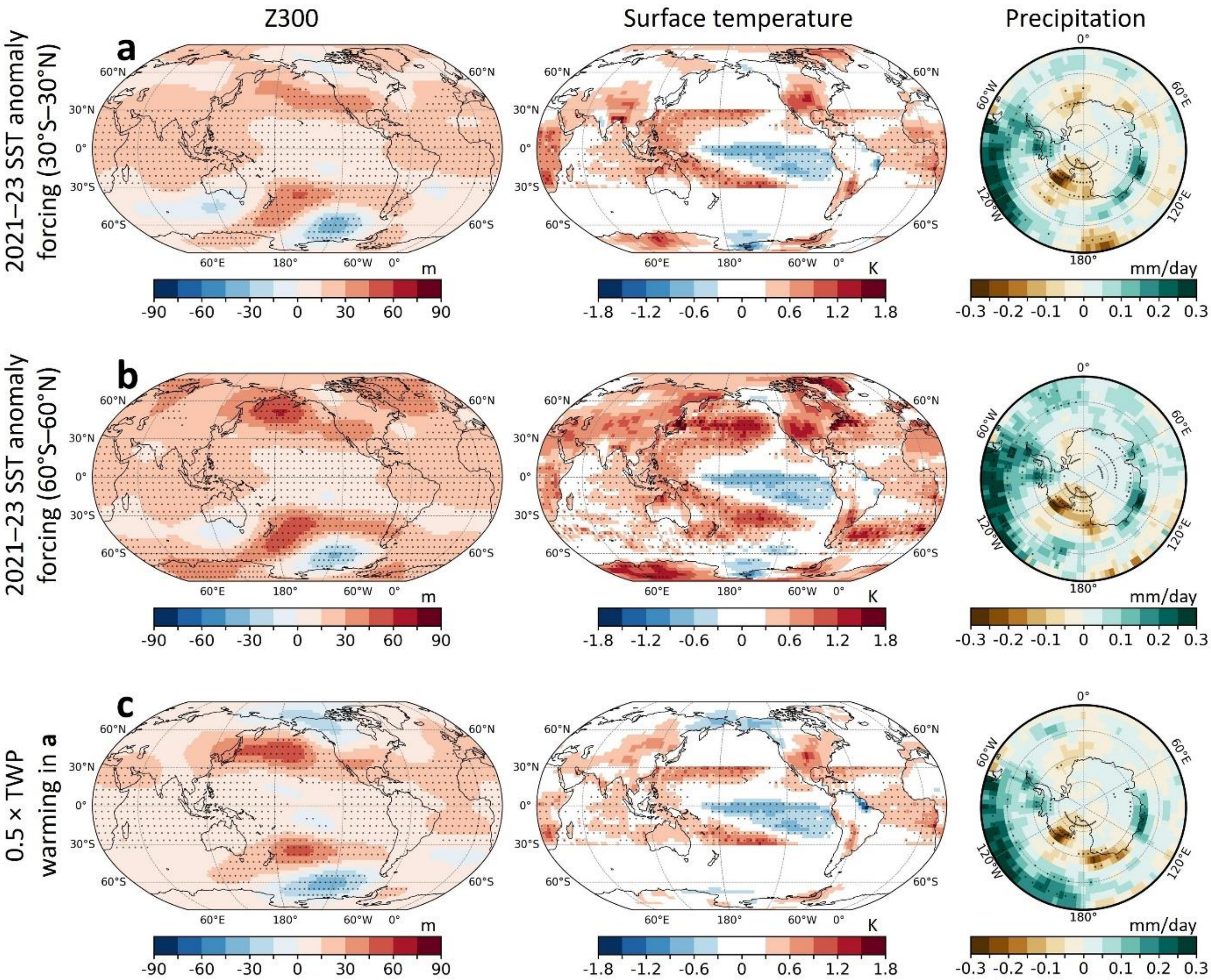

*Resp Fig. 4.1. ECHAM5-simulated atmospheric and surface responses. **a**, Responses of Z300 (left), surface temperature (middle), and precipitation (right) forced with the observed 2021–2023 SST anomaly over 30°S–30°N. **b**, As in **a**, but with the SST anomaly prescribed over 60°S–60°N. **c**, As in **a**, but with TWP warming reduced by half while retaining all other 2021–2023 SST anomalies. Stippling indicates regions significant at the 95% level based on a false-discovery rate (FDR)-corrected Student's t-test.*

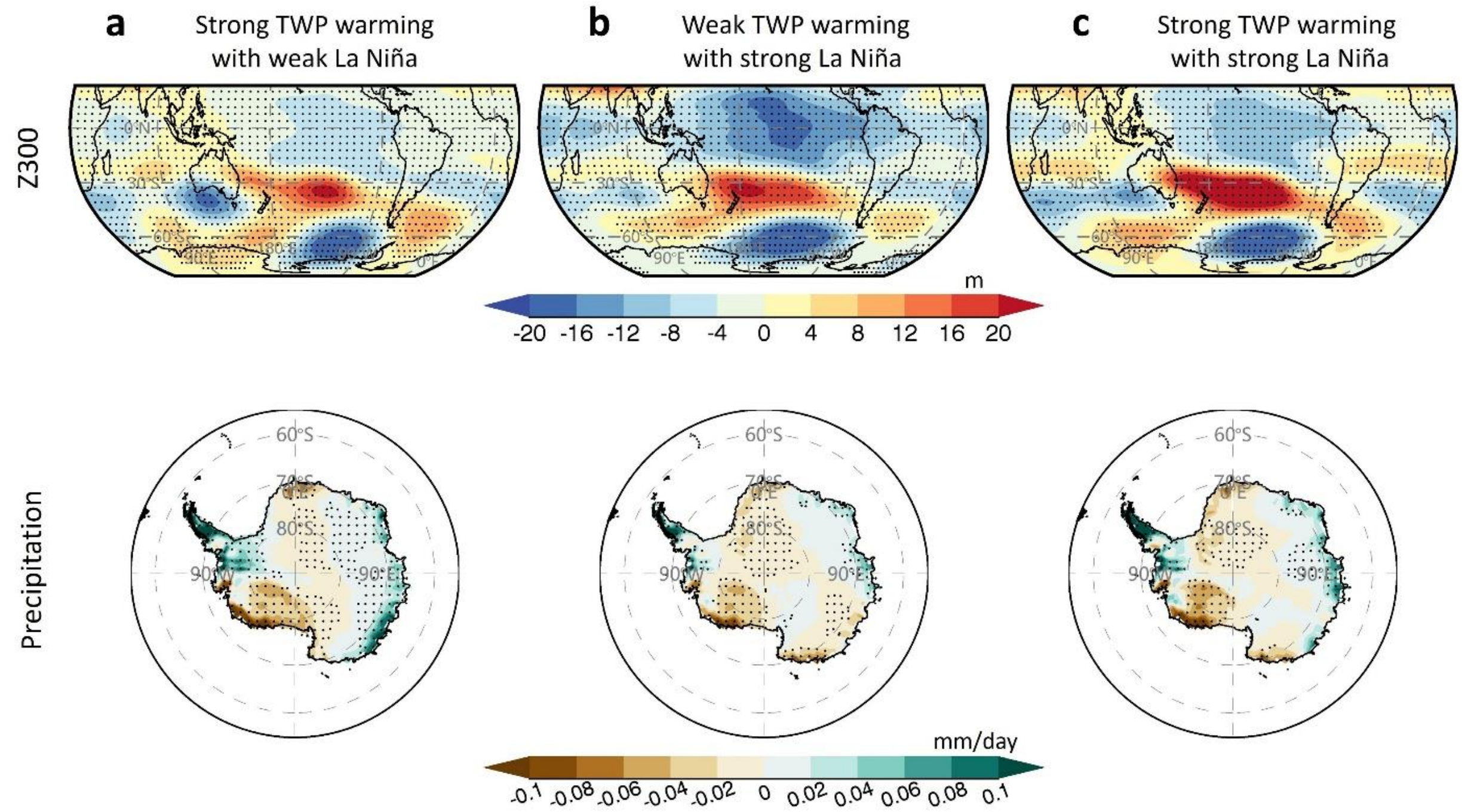

Resp Fig. 4.2. Composite Z300 and precipitation anomalies under different TWP and La Niña states in the CESM1 pre-industrial control simulation. ***a****, Strong TWP warming and weak La Niña.* ***b****, Weak TWP warming with strong La Niña.* ***c****, Concurrent strong TWP warming and strong La Niña. The composites in* ***a–c*** *are based on 1,729, 1,448, and 562 monthly samples, respectively. The TWP and Niño3.4 indices are derived from monthly SST. All anomalies are calculated relative to the full-period monthly climatology. Strong TWP warming is defined as TWP > 1 standard deviation, strong La Niña as Niño3.4 < −1 standard deviation, weak La Niña as −1 < Niño3.4 < 0 standard deviation, and weak TWP warming as 0 < TWP < 1 standard deviation. Stippling indicates regions significant at the 95% level based on an FDR-corrected Student's t-test.*

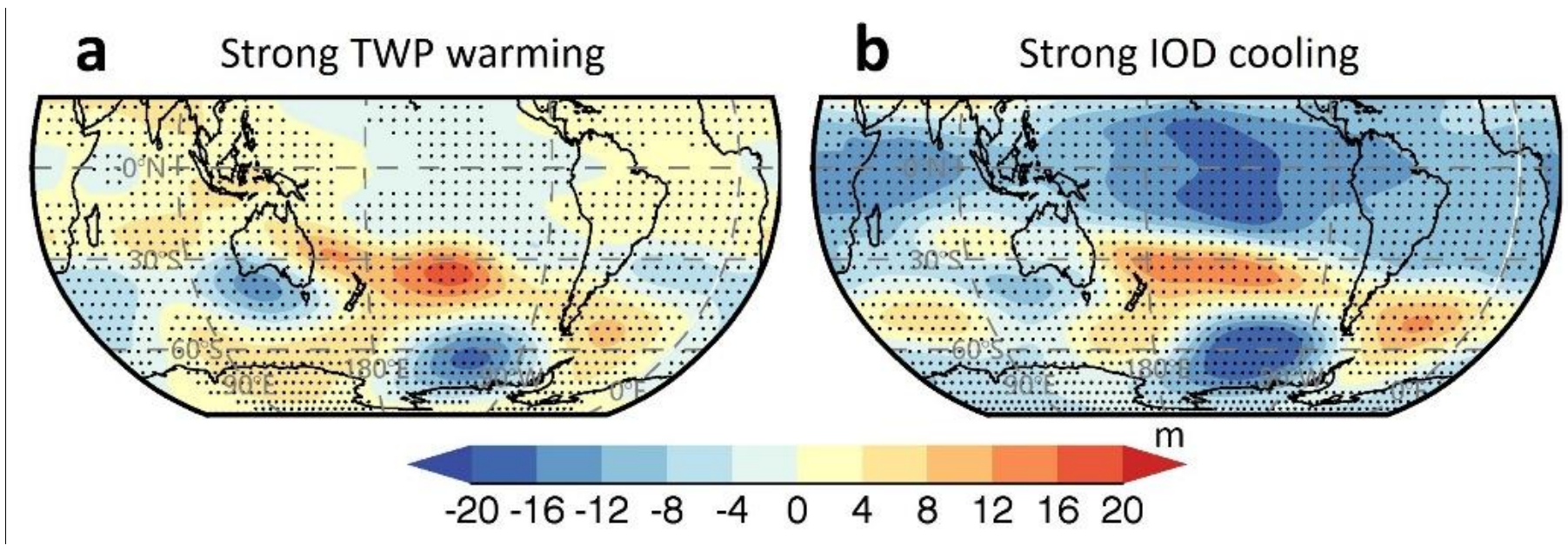

Resp Fig. 4.3. Composite Z300 anomalies under strong TWP warming and strong IOD-like cooling in the CESM1 pre-industrial control simulation. ***a****, Strong TWP warming.* ***b****, Strong IOD-like cooling. The composites in* ***a*** *and* ***b*** *are based on 3,099 and 3,498 monthly samples, respectively. The TWP and IOD-like indices are derived from monthly SST. The IOD-like index is defined as the area-mean SST over 50°–70°E, 10°S–10°N. All anomalies are calculated relative to the full-period monthly climatology. Strong TWP warming is defined as TWP > 1 standard deviation, and strong IOD-like cooling is defined as IOD-like index < −1 standard deviation. Stippling indicates regions significant at the 95% level based on an FDR-corrected Student's t-test.*
